# Supplementary material for: Rapid Mining of Novel α-Glucosidase and Lipase Inhibitors from Streptomyces sp. HO1518 Using UPLC-QTOF-MS/MS
Source: Mar Drugs. 2022 Mar 4;20(3):189. doi: 10.3390/md20030189 (PMC8955712; doi:10.3390/md20030189)
Supplement: Supplementary file 1 [file marinedrugs-20-00189-s001.zip › marinedrugs-1589284-supplementary.pdf]

Supplementary Information

Rapid Mining of Novel α-Glucosidase and Lipase Inhibitors from *Streptomyces* sp. HO1518 Using UPLC-QTOF-MS/MS

Jianlin Xu [1,2,3,†], Zhifeng Liu [2,3,†], Zhanguang Feng [2,3], Yuhong Ren [1], Haili Liu [2,*] and Yong Wang [1,2,*]

[1]*State Key Laboratory of Bioreactor Engineering, East China University of Science and Technology, Shanghai 200237, China*

[2]*CAS-Key Laboratory of Synthetic Biology, CAS Center for Excellence in Molecular Plant Sciences, Institute of Plant Physiology and Ecology, Chinese Academy of Sciences, Shanghai 200032, China*

[3]*University of Chinese Academy of Sciences, Beijing 100039, China*

*Corresponding authors. Tel./fax: +86 21 54924295.

E-mail addresses: yongwang@cemps.ac.cn (Yong Wang); hlliu@cemps.ac.cn (Haili Liu).

† These authors contributed equally to this work.

Table of Contents

Table S1 ^{1}H (500 MHz) and ^{13}C (125 MHz) NMR data of **10–13** (δppm) in D_2O.................. 8
Table S2 ^{1}H (500 MHz) and ^{13}C (125 MHz) NMR data of **14–16** (δppm) in D_2O. 10
Table S3 ^{1}H (500 MHz) and ^{13}C (125 MHz) NMR data of **10** (δppm) in D_2O...................... 12
Table S4 ^{1}H (500 MHz) and ^{13}C (125 MHz) NMR data of **11** (δppm) in D_2O...................... 14
Table S5 ^{1}H (500 MHz) and ^{13}C (125 MHz) NMR data of **12** (δppm) in D_2O...................... 16
Table S6 ^{1}H (500 MHz) and ^{13}C (125 MHz) NMR data of **13** (δppm) in D_2O...................... 18
Table S7 ^{1}H (500 MHz) and ^{13}C (125 MHz) NMR data of **14** (δppm) in D_2O...................... 20
Table S8 ^{1}H (500 MHz) and ^{13}C (125 MHz) NMR data of **15** (δppm) in D_2O...................... 22
Table S9 ^{1}H (500 MHz) and ^{13}C (125 MHz) NMR data of **16** (δppm) in D_2O...................... 24
Table S10 Acarviostatins with glucose(s) at the reducing terminus (Aca-glu) from *Streptomyces* sp. HO1518. 26
Table S11 Acarviostatins with glucose(s) at the reducing and nonreducing terminus (glu-Aca-glu) from *Streptomyces* sp. HO1518[a].. 28
Table S12 Acarviostatins with an incomplete pseudo-trisaccharide at the nonreducing terminus (incAca-glu) from *Streptomyces* sp. HO1518[a]. 29
Figure S1 HRESI-MS/MS spectra of compounds **1-9**... 31
Figure S2. Positive HRESIMS/MS fragmentation and spectra of **9** and **14-16**. (A) Positive-ion HRESIMS/MS fragmentation patterns of **9** and **14-16**; (B–E) HRESIMS/MS spectra of **9** and **14-16**.. 32
Figure S3 The inhibitory activities of **10-16** against three digestive enzymes. (A) The inhibitory activities of **10-16** against PPA. (B) The inhibitory activities of **10-16** against sucrase. (C) The inhibitory activities of **10-16** against PL. 33
Figure S4 HRESI-MS/MS spectra of compounds **17-20**... 34
Figure S5 The total ion chromatograms of fraction 1 and 2 derived from *Streptomyces* sp. HO1518. 34
Figure S6 The EIC of acarviostatins with glucose at the reducing terminus from *Streptomyces* sp. HO1518.. 36
Figure S7 The EIC of acarviostatins with glucose at the reducing and nonreducing terminus from *Streptomyces* sp. HO1518.. 36
Figure S8 The EIC of acarviostatins with an incomplete *pseudo*-tetrasaccharide at the nonreducing terminus from *Streptomyces* sp. HO1518... 37
Figure S9 Positive HRESIMS/MS fragmentation and spectra of Ac-Aca I12 and Ac-Aca I03. (A) Positive-ion HRESIMS/MS fragmentation patterns of Ac-Aca I12 and Ac-Aca I03; (B) The extracted ion chromatogram of aminooligosaccharides at *m*/*z* 1012; (C) HRESIMS/MS spectrum of Ac-Aca I12; (D) HRESIMS/MS spectrum of Ac-Aca I03. 38
Figure S10 ^{1}H NMR spectrum of compound **10** (500 MHz, D_2O).. 39
Figure S11 1D-selective TOCSY spectrum of compound **10** (500 MHz, D_2O, excitation at δ 5.23, H-**A**1α). 40
Figure S12 1D-selective TOCSY spectrum of compound **10** (500 MHz, D_2O, excitation at δ 4.65, H-**A**1β)... 41
Figure S13 1D-selective TOCSY spectrum of compound **10** (500 MHz, D_2O, excitation at δ 5.41, H-**B**1, and H-**C**1). 42

Figure S14 1D-selective TOCSY spectrum of compound **10** (500 MHz, D_2O, excitation at δ 4.22, H-**C**6)....... 43
Figure S15 1D-selective TOCSY spectrum of compound **10** (500 MHz, D_2O, excitation at δ 5.33, H-**D**1 and H-**G**1). 44
Figure S16 1D-selective TOCSY spectrum of compound **10** (500 MHz, D_2O, excitation at δ 5.98, H-**E**7)....... 45
Figure S17 1D-selective TOCSY spectrum of compound **10** (500 MHz, D_2O, excitation at δ 5.38, H-**F**1)....... 46
Figure S18 1D-selective TOCSY spectrum of compound **10** (500 MHz, D_2O, excitation at δ 5.90, H-**H**7). 47
Figure S19 ^{13}C NMR spectrum of compound **10** (125 MHz, D_2O)....... 48
Figure S20 DEPT-135 spectrum of compound **10** (125 MHz, D_2O)....... 49
Figure S21 HSQC spectrum of compound **10** (500 MHz, D_2O). 50
Figure S22 ^{1}H-^{1}H COSY spectrum of compound **10** (500 MHz, D_2O)....... 51
Figure S23 2D-TOCSY spectrum of compound **10** (500 MHz, D_2O)....... 52
Figure S24 HSQC-TOCSY spectrum of compound **10** (500 MHz, D_2O)....... 53
Figure S25 HMBC spectrum of compound **10** (500 MHz, D_2O). 54
Figure S26 NOESY spectrum of compound **10** (500 MHz, D_2O)....... 55
Figure S27 HRESIMS spectrum of compound **10**. 56
Figure S28 UV spectrum of compound **10**....... 56
Figure S29 IR spectrum of compound **10**....... 57
Figure S30 ^{1}H NMR spectrum of compound **11** (500 MHz, D_2O)....... 58
Figure S31 1D-selective TOCSY spectrum of compound **11** (500 MHz, D_2O, excitation at δ 4.66, H-**A**1β)....... 59
Figure S32 1D-selective TOCSY spectrum of compound **11** (500 MHz, D_2O, excitation at δ 5.41, H-**B**1 and H-**C**1)....... 60
Figure S33 1D-selective TOCSY spectrum of compound **11** (500 MHz, D_2O, excitation at δ 4.44, H-**C**6a)....... 61
Figure S34 1D-selective TOCSY spectrum of compound **11** (500 MHz, D_2O, excitation at δ 5.29, H-**D**1)....... 62
Figure S35 1D-selective TOCSY spectrum of compound **11** (500 MHz, D_2O, excitation at δ 5.98, H-**E**7)....... 63
Figure S36 1D-selective TOCSY spectrum of compound **11** (500 MHz, D_2O, excitation at δ 5.38, H-**F**1)....... 64
Figure S37 1D-selective TOCSY spectrum of compound **11** (500 MHz, D_2O, excitation at δ 5.32, H-**G**1). 65
Figure S38 1D-selective TOCSY spectrum of compound **11** (500 MHz, D_2O, excitation at δ 5.90, H-**H**7). 66
Figure S39 ^{13}C NMR spectrum of compound **11** (125 MHz, D_2O)....... 67
Figure S40 DEPT-135 spectrum of compound **11** (125 MHz, D_2O)....... 68
Figure S41 HSQC spectrum of compound **11** (500 MHz, D_2O). 69
Figure S42 ^{1}H-^{1}H COSY spectrum of compound **11** (500 MHz, D_2O)....... 70
Figure S43 2D-TOCSY spectrum of compound **11** (500 MHz, D_2O)....... 71
Figure S44 HSQC-TOCSY spectrum of compound **11** (500 MHz, D_2O)....... 72

Figure S45 HMBC spectrum of compound **11** (500 MHz, D_2O). 73
Figure S46 NOESY spectrum of compound **11** (500 MHz, D_2O). 74
Figure S47 HRESIMS spectrum of compound **11**. 75
Figure S48 UV spectrum of compound **11**. 75
Figure S49 IR spectrum of compound **11**. 76
Figure S50 ^{1}H NMR spectrum of compound **12** (500 MHz, D_2O). 77
Figure S51 1D-selective TOCSY spectrum of compound **12** (500 MHz, D_2O, excitation at δ 5.24, H-**A**1α). 78
Figure S52 1D-selective TOCSY spectrum of compound **12** (500 MHz, D_2O, excitation at δ 4.66, H-**A**1β). 79
Figure S53 1D-selective TOCSY spectrum of compound **12** (500 MHz, D_2O, excitation at δ 5.38, H-**B**1 and H-**C**1). 80
Figure S54 1D-selective TOCSY spectrum of compound **12** (500 MHz, D_2O, excitation at δ 4.44, H-**C**6a). 81
Figure S55 1D-selective TOCSY spectrum of compound **12** (500 MHz, D_2O, excitation at δ 5.28, H-**D**1). 82
Figure S56 1D-selective TOCSY spectrum of compound **12** (500 MHz, D_2O, excitation at δ 5.98, H-**E**1). 83
Figure S57 1D-selective TOCSY spectrum of compound **12** (500 MHz, D_2O, excitation at δ 5.42, H-**F**1). 84
Figure S58 1D-selective TOCSY spectrum of compound **12** (500 MHz, D_2O, excitation at δ 5.33, H-**G**1). 85
Figure S59 1D-selective TOCSY spectrum of compound **12** (500 MHz, D_2O, excitation at δ 5.91, H-**H**1). 86
Figure S60 ^{13}C NMR spectrum of compound **12** (125 MHz, D_2O). 87
Figure S61 DEPT-135 spectrum of compound **12** (125 MHz, D_2O). 88
Figure S62 HSQC spectrum of compound **12** (500 MHz, D_2O). 89
Figure S63 ^{1}H-^{1}H COSY spectrum of compound **12** (500 MHz, D_2O). 90
Figure S64 2D-TOCSY spectrum of compound **12** (500 MHz, D_2O). 91
Figure S65 HSQC-TOCSY spectrum of compound **12** (500 MHz, D_2O). 92
Figure S66 HMBC spectrum of compound **12** (500 MHz, D_2O). 93
Figure S67 NOESY spectrum of compound **12** (500 MHz, D_2O). 94
Figure S68 HRESIMS spectrum of compound **12**. 95
Figure S69 UV spectrum of compound **12**. 95
Figure S70 IR spectrum of compound **12**. 96
Figure S71 ^{1}H NMR spectrum of compound **13** (500 MHz, D_2O). 97
Figure S72 1D-selective TOCSY spectrum of compound **13** (500 MHz, D_2O, excitation at δ 5.24, H-**A**1α). 98
Figure S73 1D-selective TOCSY spectrum of compound **13** (500 MHz, D_2O, excitation at δ 4.66, H-**A**1β). 99
Figure S74 1D-selective TOCSY spectrum of compound **13** (500 MHz, D_2O, excitation at δ 5.41, H-**B**1 and H-**C**1). 100
Figure S75 1D-selective TOCSY spectrum of compound **13** (500 MHz, D_2O, excitation at δ 4.49, H-**C**6). 101

Figure S76 1D-selective TOCSY spectrum of compound **13** (500 MHz, D_2O, excitation at δ 5.29, H-**D**1)........ 102
Figure S77 1D-selective TOCSY spectrum of compound **13** (500 MHz, D_2O, excitation at δ 5.99, H-**E**7)........ 103
Figure S78 1D-selective TOCSY spectrum of compound **13** (500 MHz, D_2O, excitation at δ 5.38, H-**F**1)........ 104
Figure S79 1D-selective TOCSY spectrum of compound **13** (500 MHz, D_2O, excitation at δ 5.33, H-**G**1)........ 105
Figure S80 1D-selective TOCSY spectrum of compound **13** (500 MHz, D_2O, excitation at δ 5.91, H-**H**7)........ 106
Figure S81 ^{13}C NMR spectrum of compound **13** (125 MHz, D_2O)........ 107
Figure S82 DEPT-135 spectrum of compound **13** (125 MHz, D_2O)........ 108
Figure S83 HSQC spectrum of compound **13** (500 MHz, D_2O)........ 109
Figure S84 ^{1}H-^{1}H COSY spectrum of compound **13** (500 MHz, D_2O)........ 110
Figure S85 2D-TOCSY spectrum of compound **13** (500 MHz, D_2O)........ 111
Figure S86 HSQC-TOCSY spectrum of compound **13** (500 MHz, D_2O)........ 112
Figure S87 HMBC spectrum of compound **13** (500 MHz, D_2O)........ 113
Figure S88 NOESY spectrum of compound **13** (500 MHz, D_2O)........ 114
Figure S89 HRESIMS spectrum of compound **13**........ 115
Figure S90 UV spectrum of compound **13**........ 115
Figure S91 IR spectrum of compound **13**........ 116
Figure S92 ^{1}H NMR spectrum of compound **14** (500 MHz, D_2O)........ 117
Figure S93 1D-selective TOCSY spectrum of compound **14** (500 MHz, D_2O, excitation at δ 5.20, H-**A**1α)........ 118
Figure S94 1D-selective TOCSY spectrum of compound **14** (500 MHz, D_2O, excitation at δ 4.62, H-**A**1β)........ 119
Figure S95 1D-selective TOCSY spectrum of compound **14** (500 MHz, D_2O, excitation at δ 5.38, H-**B**1, H-**C**1, and H-**D**1)........ 120
Figure S96 1D-selective TOCSY spectrum of compound **14** (500 MHz, D_2O, excitation at δ 4.40, H-**D**6a)........ 121
Figure S97 1D-selective TOCSY spectrum of compound **14** (500 MHz, D_2O, excitation at δ 5.25, H-**E**1)........ 122
Figure S98 1D-selective TOCSY spectrum of compound **14** (500 MHz, D_2O, excitation at δ 5.94, H-**F**7)........ 123
Figure S99 1D-selective TOCSY spectrum of compound **14** (500 MHz, D_2O, excitation at δ 5.34, H-**G1**)........ 124
Figure S100 1D-selective TOCSY spectrum of compound **14** (500 MHz, D_2O, excitation at δ 5.29, H-**H1**)........ 125
Figure S101 1D-selective TOCSY spectrum of compound **14** (500 MHz, D_2O, excitation at δ 5.86, H-**I1**)........ 126
Figure S102 ^{13}C NMR spectrum of compound **14** (125 MHz, D_2O)........ 127
Figure S103 DEPT-135 spectrum of compound **14** (125 MHz, D_2O)........ 128
Figure S104 HSQC spectrum of compound **14** (500 MHz, D_2O)........ 129
Figure S105 ^{1}H-^{1}H COSY spectrum of compound **14** (500 MHz, D_2O)........ 130

Figure S106 2D-TOCSY spectrum of compound **14** (500 MHz, D_2O)............................... 131
Figure S107 HSQC-TOCSY spectrum of compound **14** (500 MHz, D_2O)......................... 132
Figure S108 HMBC spectrum of compound **14** (500 MHz, D_2O)...................................... 133
Figure S109 NOESY spectrum of compound **14** (500 MHz, D_2O)..................................... 134
Figure S110 HRESIMS spectrum of compound **14**. 135
Figure S111 UV spectrum of compound **14**.. 135
Figure S112 IR spectrum of compound **14**... 136
Figure S113 ^{1}H NMR spectrum of compound **15** (500 MHz, D_2O).................................... 137
Figure S114 1D-selective TOCSY spectrum of compound **15** (500 MHz, D_2O, excitation at δ 5.23, H-A1α). 138
Figure S115 1D-selective TOCSY spectrum of compound **15** (500 MHz, D_2O, excitation at δ 4.65, H-A1β).. 139
Figure S116 1D-selective TOCSY spectrum of compound **15** (500 MHz, D_2O, excitation at δ 5.40, H-**B**1, **C**1, and **D**1). 140
Figure S117 1D-selective TOCSY spectrum of compound **15** (500 MHz, D_2O, excitation at δ 4.45, H-**D**6a)... 141
Figure S118 1D-selective TOCSY spectrum of compound **15** (500 MHz, D_2O, excitation at δ 5.28, H-**E**1)... 142
Figure S119 1D-selective TOCSY spectrum of compound **15** (500 MHz, D_2O, excitation at δ 5.97, H-**F**1)... 143
Figure S120 1D-selective TOCSY spectrum of compound **15** (500 MHz, D_2O, excitation at δ 5.37, H-**G**1). 144
Figure S121 1D-selective TOCSY spectrum of compound **15** (500 MHz, D_2O, excitation at δ 5.32, H-**H**1). 145
Figure S122 1D-selective TOCSY spectrum of compound **15** (500 MHz, D_2O, excitation at δ 5.90, H-**I**1).. 146
Figure S123 ^{13}C NMR spectrum of compound **15** (125 MHz, D_2O).................................. 147
Figure S124 DEPT-135 spectrum of compound **15** (125 MHz, D_2O)................................ 148
Figure S125 HSQC spectrum of compound **15** (500 MHz, D_2O). 149
Figure S126 ^{1}H-^{1}H COSY spectrum of compound **15** (500 MHz, D_2O)............................ 150
Figure S127 2D-TOCSY spectrum of compound **15** (500 MHz, D_2O)............................... 151
Figure S128 HSQC-TOCSY spectrum of compound **15** (500 MHz, D_2O)......................... 152
Figure S129 HMBC spectrum of compound **15** (500 MHz, D_2O)...................................... 153
Figure S130 NOESY spectrum of compound **15** (500 MHz, D_2O)..................................... 154
Figure S131 HRESIMS spectrum of compound **15**. 155
Figure S132 UV spectrum of compound **15**.. 155
Figure S133 IR spectrum of compound **15**... 156
Figure S134 ^{1}H NMR spectrum of compound **16** (500 MHz, D_2O).................................... 157
Figure S135 1D-selective TOCSY spectrum of compound **16** (500 MHz, D_2O, excitation at δ 5.20, H-**A**1α). 158
Figure S136 1D-selective TOCSY spectrum of compound **16** (500 MHz, D_2O, excitation at δ 4.63, H-**A**1β).. 159
Figure S137 1D-selective TOCSY spectrum of compound **16** (500 MHz, D_2O, excitation at δ 5.38, H-**B**1, H-**C**1, H-**D**1)... 160

Figure S138 1D-selective TOCSY spectrum of compound **16** (500 MHz, D_2O, excitation at δ 4.43, H-**D**6a). 161
Figure S139 1D-selective TOCSY spectrum of compound **16** (500 MHz, D_2O, excitation at δ 5.24, H-**E**1). 162
Figure S140 1D-selective TOCSY spectrum of compound **16** (500 MHz, D_2O, excitation at δ 5.94, H-**F**7). 163
Figure S141 1D-selective TOCSY spectrum of compound **16** (500 MHz, D_2O, excitation at δ 5.34, H-**G**1). 164
Figure S142 1D-selective TOCSY spectrum of compound **16** (500 MHz, D_2O, excitation at δ 5.30, H-**H**1). 165
Figure S143 1D-selective TOCSY spectrum of compound **16** (500 MHz, D_2O, excitation at δ 5.87, H-**I**7). 166
Figure S144 ^{13}C NMR spectrum of compound **16** (125 MHz, D_2O). 167
Figure S145 DEPT-135 spectrum of compound **16** (125 MHz, D_2O). 168
Figure S146 HSQC spectrum of compound **16** (500 MHz, D_2O). 169
Figure S147 ^1H-^1H COSY spectrum of compound **16** (500 MHz, D_2O). 170
Figure S148 2D-TOCSY spectrum of compound **16** (500 MHz, D_2O). 171
Figure S149 HSQC-TOCSY spectrum of compound **16** (500 MHz, D_2O). 172
Figure S150 HMBC spectrum of compound **16** (500 MHz, D_2O). 173
Figure S151 NOESY spectrum of compound **16** (500 MHz, D_2O). 174
Figure S152 HRESIMS spectrum of compound **16**. 175
Figure S153 UV spectrum of compound **16**. 175
Figure S154 IR spectrum of compound **16**. 176

Table S1. ^{1}H (500 MHz) and ^{13}C (125 MHz) NMR data of **10–13**δ(ppm) in D_2O.

No.	**10**		**11**		**12**		**13**	
	δ_C	δ_H	δ_C	δ_H	δ_C	δ_H	δ_C	δ_H
A1α	93.4, CH	5.23, d (3.5, 1H)	95.3, CH	5.23, d (3.5, 1H)	91.9, CH	5.24, d (3.4, 1H)	92.0, CH	5.24, d (3.5, 1H)
A2α	71.2, CH	3.57, m (1H)	74.8, CH	3.58, m (1H)	71.3, CH	3.52, m (1H)	71.4, CH	3.51, m (1H)
A3α	73.2, CH	4.09, m (1H)	76.7, CH	3.97, m (1H)	73.3, CH	4.16, m (1H)	73.4, CH	3.92, m (1H)
A4α	76.8, CH	3.68, m (1H)	80.2, CH	3.65, m (1H)	76.8, CH	3.69, m (1H)	76.9, CH	3.69, m (1H)
A5α	70.0, CH	3.97, m (1H)	73.3, CH	3.87, m (1H)	70.0, CH	3.90, m (1H)	70.0, CH	3.82, m (1H)
A6α	60.5, CH_2	3.85, m (2H)	63.8, CH_2	3.87, m (2H)	60.5, CH_2	3.74, m (2H)	60.5, CH_2	3.79, m (2H)
A1β	95.8, CH	4.65, d (8.0, 1H)	99.2, CH	4.66, d (8.0, 1H)	95.8, CH	4.66, d (8.0, 1H)	95.8, CH	4.66, d (7.8, 1H)
A2β	74.0, CH	3.27, t (9.0, 1H)	77.4, CH	3.28, t (9.0, 1H)	74.0, CH	3.29, m (1H)	74.0, CH	3.28, m (1H)
A3β	76.2, CH	3.76, m (1H)	79.7, CH	3.77, m (1H)	76.3, CH	3.78, m (1H)	76.2, CH	3.77, m (1H)
A4β	76.9, CH	3.65, m (1H)	80.2, CH	3.66, m (1H)	76.8, CH	3.67, m (1H)	76.8, CH	3.66, m (1H)
A5β	74.6, CH	3.60, m (1H)	77.9, CH	3.62, m (1H)	74.6, CH	3.62, m (1H)	74.6, CH	3.60, m (1H)
A6β	60.5, CH_2	3.90, m (2H)	64.1, CH_2	3.91, m (2H)	60.6, CH_2	3.92, m (2H)	60.5, CH_2	3.90, m (2H)
B1	99.5, CH	5.41, d (3.5, 1H)	102.8, CH	5.41, d (3.5, 1H)	99.6, CH	5.38, d (3.5, 1H)	99.4, CH	5.41, d (3.5, 1H)
B2	71.5, CH	3.62, m (1H)	74.9, CH	3.64, m (1H)	71.6, CH	3.58, m (1H)	71.6, CH	3.62, m (1H)
B3	73.4, CH	3.95, m (1H)	76.7, CH	3.96, m (1H)	73.4, CH	3.94, m (1H)	73.2, CH	3.96, m (1H)
B4	77.0, CH	3.67, m (1H)	80.4, CH	3.67, m (1H)	77.1, CH	3.63, m (1H)	77.1, CH	3.64, m (1H)
B5	71.2, CH	3.85, m (1H)	74.6, CH	3.85, m (1H)	71.2, CH	3.80, m (1H)	71.3, CH	3.85, m (1H)
B6	60.7, CH_2	3.85, m (2H)	63.9, CH_2	3.85, m (2H)	60.7, CH_2	3.80, m (2H)	60.7, CH_2	3.85, m (2H)
C1	99.4, CH	5.41, d (3.5, 1H)	103.0, CH	5.41, d (3.5, 1H)	99.4, CH	5.38, d (3.5, 1H)	99.6, CH	5.41, d (3.5, 1H)
C2	71.0, CH	3.66, m (1H)	74.5, CH	3.67, m (1H)	71.1, CH	3.62, m (1H)	71.2, CH	3.62, m (1H)
C3	73.3, CH	3.83, m (1H)	76.6, CH	3.95, m (1H)	73.2, CH	3.92, m (1H)	73.2, CH	3.96, m (1H)
C4	77.0, CH	3.68, m (1H)	81.3, CH	3.67, m (1H)	78.2, CH	3.62, m (1H)	78.0, CH	3.64, m (1H)
C5	69.4, CH	4.04, d (12.0, 1H)	72.3, CH	4.05, d (12.0, 1H)	69.0, CH	4.03, d (12.0, 1H)	69.0, CH	4.05, d (12.0, 1H)
C6a	63.2, CH_2	4.15, m (1H)	63.8, CH_2	4.22, m (1H)	63.5, CH_2	4.20, m (1H)	63.4, CH_2	4.23, m (1H)
C6b		4.23, d (12.0, 1H)		4.44, d (12.0, 1H)		4.45, d (12.0, 1H)		4.49, d (12.0, 1H)
D1	99.9, CH	5.33, d (3.5, 1H)	104.0, CH	5.29, d (3.5, 1H)	100.7, CH	5.28, d (3.5, 1H)	100.6, CH	5.29, d (3.5, 1H)
D2	71.2, CH	3.56, m (1H)	74.6, CH	3.56, m (1H)	71.2, CH	3.54, m (1H)	71.3, CH	3.58, m (1H)
D3	72.7, CH	3.59, m (1H)	76.3, CH	3.59, m (1H)	73.0, CH	3.56, m (1H)	73.0, CH	3.60, m (1H)
D4	64.2, CH	2.48, t (9.0, 1H)	67.7, CH	2.48, t (9.0, 1H)	64.3, CH	2.47, t (8.8, 1H)	64.3, CH	2.47, t (9.0, 1H)
D5	69.7, CH	3.76, m (1H)	73.2, CH	3.73, m (1H)	69.8, CH	3.71, m (1H)	69.8, CH	3.75, m (1H)
D6	17.4, CH_3	1.34, d (5.7, 3H)	20.7, CH_3	1.30, d (5.7, 3H)	17.4, CH_3	1.31, d (5.6, 3H)	17.4, CH_3	1.32, d (5.6, 3H)
E1	55.0, CH	3.54, m (1H)	58.4, CH	3.53, m (1H)	55.0, CH	3.53, m (1H)	55.0, CH	3.48, m (1H)
E2	70.7, CH	3.82, m (1H)	73.0, CH	3.80, m (1H)	70.8, CH	3.80, m (1H)	69.7, CH	3.79, m (1H)
E3	70.8, CH	4.13, m (1H)	74.1, CH	4.13, m (1H)	70.9, CH	4.14, m (1H)	70.8, CH	4.14, m (1H)
E4	76.1, CH	4.22, m (1H)	79.6, CH	4.22, m (1H)	76.2, CH	4.23, m (1H)	76.4, CH	4.23, m (1H)
E5	136.5, C		139.9, C		136.5, C		136.5, C	
E6a	62.0, CH_2	4.13, m (1H)	65.4, CH_2	4.13, m (1H)	62.0, CH_2	4.14, m (1H)	62.0, CH_2	4.14, m (1H)
E6b		4.22, m (1H)		4.22, m (1H)		4.23, m (1H)		4.23, m (1H)

E7	126.3, CH	5.98, d (3.2, 1H)	129.7, CH	5.98, d (3.2, 1H)	126.3, CH	5.98, d (3.1, 1H)	126.4, CH	5.99, d (3.2, 1H)
F1	97.5, CH	5.38, d (3.4, 1H)	101.0, CH	5.38, d (3.4, 1H)	97.8, CH	5.42, d (3.5, 1H)	97.6, CH	5.38, d (3.5, 1H)
F2	72.9, CH	3.58, m (1H)	74.9, CH	3.62, m (1H)	73.2, CH	3.68, m (1H)	72.7, CH	3.61, m (1H)
F3	73.5, CH	3.93, m (1H)	76.9, CH	3.93, m (1H)	73.5, CH	3.94, m (1H)	73.5, CH	3.93, m (1H)
F4	70.8, CH	3.60, m (1H)	74.2, CH	3.64, m (1H)	70.9, CH	3.66, m (1H)	70.9, CH	3.61, m (1H)
F5	71.1, CH	3.95, m (1H)	74.5, CH	3.93, m (1H)	71.2, CH	3.96, m (1H)	71.2, CH	3.93, m (1H)
F6	60.4, CH_2	3.81, m (2H)	66.9, CH_2	3.81, m (2H)	60.4, CH_2	3.82, m (2H)	60.7, CH_2	3.82, m (2H)
G1	99.6, CH	5.33, d (3.4, 1H)	103.3, CH	5.32, d (3.4, 1H)	99.9, CH	5.33, d (3.4, 1H)	100.0, CH	5.33, d (3.4, 1H)
G2	71.3, CH	3.56, m (1H)	74.7, CH	3.58, m (1H)	71.2, CH	3.61, m (1H)	71.5, CH	3.58, m (1H)
G3	72.2, CH	3.59, m (1H)	75.9, CH	3.62, m (1H)	72.6, CH	3.61, m (1H)	72.6, CH	3.60, m (1H)
G4	64.9, CH	2.48, t (9.0, 1H)	68.3, CH	2.48, t (9.0, 1H)	64.9, CH	2.47, t (8.8, 1H)	65.0, CH	2.47, t (9.0, 1H)
G5	69.6, CH	3.86, m (1H)	73.0, CH	3.76, m (1H)	69.6, CH	3.77, m (1H)	69.6, CH	3.75, m (1H)
G6	17.3, CH_3	1.34, d (5.7, 3H)	20.8, CH_3	1.34, d (5.7, 3H)	17.3, CH_3	1.35, d (5.6, 3H)	17.4, CH_3	1.35, d (5.6, 3H)
H1	56.0, CH	3.52, m (1H)	59.4, CH	3.54, m (1H)	56.0, CH	3.54, m (1H)	56.0, CH	3.48, m (1H)
H2	72.6, CH	3.72, m (1H)	76.1, CH	3.68, m (1H)	72.7, CH	3.69, m (1H)	72.8, CH	3.67, m (1H)
H3	72.6, CH	3.75, m (1H)	76.3, CH	3.77, m (1H)	72.9, CH	3.77, m (1H)	73.0, CH	3.76, m (1H)
H4	70.9, CH	4.07, d (12.0, 1H)	74.3, CH	4.04, d (12.0, 1H)	71.0, CH	4.06, d (12.0, 1H)	71.0, CH	4.05, d (12.0, 1H)
H5	139.0, C		142.3, C		139.0, C		139.0, C	
H6a<br>H6b	61.6, CH_2	4.12, m (2H)	65.0 CH_2	4.12, m (1H)<br>4.22, m (1H)	61.6, CH_2	4.13, m (1H)<br>4.25, m (1H)	61.6, CH_2	4.12, m (1H)<br>4.23, m (1H)
H7	123.7, CH	5.90, d (3.5, 1H)	127.2, CH	5.90, d (3.5, 1H)	123.8, CH	5.91, d (3.4, 1H)	123.8, CH	5.91, d (3.4, 1H)
1’			180.8, C=O		180.1, C=O		176.2, C=O	
2’			30.6, CH_2	2.48, m (2H)	33.9, CH	2.72, m (2H)	42.9, CH_2	2.36, m (2H)
3’			11.7, CH_3	1.13, d (7.2, 3H)	18.3, CH_3	1.19, d (3.0, 3H)	25.5, CH	2.08, m (1H)
4’					18.2, CH_3	1.19, d (3.0, 3H)	21.7, CH_3	0.96, d (6.6, 3H)
5’							21.7, CH_3	0.96, d (6.6, 3H)

Table S2. ^{1}H (500 MHz) and ^{13}C (125 MHz) NMR data of **14–16** (δ ppm) in D_2O.

No.	14		15		16	
	δ_C	δ_H	δ_C	δ_H	δ_C	δ_H
A1α	94.7, CH	5.20, d (3.5, 1H)	91.9, CH	5.22, d (3.5, 1H)	91.9, CH	5.21, d (3.5, 1H)
A2α	74.2, CH	3.53, m (1H)	71.4, CH	3.52, m (1H)	71.3, CH	3.50, m (1H)
A3α	76.1, CH	3.93, m (1H)	73.3, CH	3.93, m (1H)	73.3, CH	3.92, m (1H)
A4α	79.6, CH	3.61, m (1H)	76.8, CH	3.62, m (1H)	76.8, CH	3.64, m (1H)
A5α	72.8, CH	3.85, m (1H)	69.9, CH	3.85, m (1H)	69.9, CH	3.84, m (1H)
A6α	63.2, CH_2	3.78, m (2H)	60.4, CH_2	3.79, m (2H)	60.4, CH_2	3.78, m (2H)
A1β	98.6, CH	4.62, d (8.0, 1H)	95.8, CH	4.65, d (8.0, 1H)	95.8, CH	4.63, d (8.0, 1H)
A2β	76.8, CH	3.24, t (9.0, 1H)	74.0, CH	3.27, m (1H)	74.0, CH	3.25, m (1H)
A3β	79.1, CH	3.74, m (1H)	76.3, CH	3.77, m (1H)	76.2, CH	3.73, m (1H)
A4β	79.7, CH	3.63, m (1H)	76.8, CH	3.65, m (1H)	76.8, CH	3.64, m (1H)
A5β	77.4, CH	3.61, m (1H)	74.5, CH	3.60, m (1H)	74.5, CH	3.61, m (1H)
A6β	63.3, CH_2	3.88, m (2H)	60.5, CH_2	3.89, m (2H)	60.4, CH_2	3.88, m (2H)
B1	102.2, CH	5.38, d (3.5, 1H)	99.4, CH	5.40, d (3.5, 1H)	99.4, CH	5.38, d (3.5, 1H)
B2	74.4, CH	3.61, m (1H)	71.5, CH	3.60, m (1H)	71.6, CH	3.59, m (1H)
B3	76.2, CH	3.93, m (1H)	73.3, CH	3.95, m (1H)	73.2, CH	3.94, m (1H)
B4	79.9, CH	3.63, m (1H)	77.0, CH	3.64, m (1H)	77.0, CH	3.64, m (1H)
B5	74.0, CH	3.82, m (1H)	71.2, CH	3.82, m (1H)	71.2, CH	3.82, m (1H)
B6	63.4, CH_2	3.82, m (2H)	60.7, CH_2	3.84, m (2H)	60.5, CH_2	3.82, m (2H)
C1	102.3, CH	5.38, d (3.5, 1H)	99.5, CH	5.40, d (3.5, 1H)	99.5, CH	5.38, d (3.5, 1H)
C2	74.3, CH	3.61, m (1H)	71.5, CH	3.60, m (1H)	71.5, CH	3.59, m (1H)
C3	76.1, CH	3.93, m (1H)	73.2, CH	3.95, m (1H)	73.3, CH	3.94, m (1H)
C4	79.9, CH	3.63, m (1H)	77.0, CH	3.64, m (1H)	76.9, CH	3.64, m (1H)
C5	74.1, CH	3.82, m (1H)	71.2, CH	3.82, m (1H)	71.2, CH	3.82, m (1H)
C6	63.5, CH_2	3.82, m (2H)	60.8, CH_2	3.84, m (2H)	60.7, CH_2	3.82, m (2H)
D1	102.4, CH	5.38, d (3.5, 1H)	99.6, CH	5.40, d (3.5, 1H)	99.6, CH	5.38, d (3.5, 1H)
D2	73.9, CH	3.63, m (1H)	71.1, CH	3.63, m (1H)	71.1, CH	3.64, m (1H)
D3	76.0, CH	3.93, m (1H)	73.2, CH	3.94, m (1H)	73.1, CH	3.93, m (1H)
D4	80.7, CH	3.63, m (1H)	77.9, CH	3.63, m (1H)	78.4, CH	3.64, m (1H)
D5	71.7, CH	4.01, d (12.0, 1H)	68.9, CH	4.04, d (12.0, 1H)	69.0, CH	4.01, d (12.0, 1H)
D6a	67.1, CH_2	4.19, m (1H)	64.3, CH_2	4.22, m (1H)	63.4, CH_2	4.19, m (1H)
D6b		4.44, d (12.0, 1H)		4.45, dd (12.0, 3.0, 1H)		4.43, d (12.0, 1H)
E1	103.4, CH	5.25, d (3.5, 1H)	100.5, CH	5.28, d (3.4, 1H)	100.7, CH	5.24, d (3.5, 1H)
E2	74.1, CH	3.57, m (1H)	71.3, CH	3.53, m (1H)	71.3, CH	3.50, m (1H)
E3	75.8, CH	3.61, m (1H)	72.9, CH	3.58, m (1H)	72.9, CH	3.52, m (1H)
E4	67.8, CH	2.43, t (9.0, 1H)	64.9, CH	2.45, t (9.0, 1H)	64.3, CH	2.43, t (9.0, 1H)
E5	72.6, CH	3.73, m (1H)	69.8, CH	3.73, m (1H)	69.7, CH	3.71, m (1H)
E6	20.2, CH_3	1.27, d (5.6, 3H)	17.4, CH_3	1.30, d (6.0, 3H)	17.4, CH_3	1.29, d (5.6, 3H)
F1	57.8, CH	3.49, m (1H)	55.0, CH	3.53, m (1H)	55.0, CH	3.45, m (1H)

F2	72.5, CH	3.77, m (1H)	69.7, CH	3.79, m (1H)	69.7, CH	3.76, m (1H)
F3	73.6, CH	4.09, m (1H)	70.8, CH	4.13, m (1H)	70.8, CH	4.09, m (1H)
F4	79.0, CH	4.19, m (1H)	76.2, CH	4.22, m (1H)	76.3, CH	4.19, m (1H)
F5	139.3, C		136.5, C		136.5, C	
F6a<br>F6b	64.8, CH_2	4.09, m (1H)<br>4.19, m (1H)	62.0, CH_2	4.13, m (1H)<br>4.22, m (1H)	62.0, CH_2	4.09, m (1H)<br>4.19, m (1H)
F7	129.1, CH	5.94, d (3.2, 1H)	126.3, CH	5.97, d (3.2, 1H)	126.6, CH	5.94, d (3.1, 1H)
G1	100.4, CH	5.34, d (3.4, 1H)	97.6, CH	5.37, d (3.6, 1H)	97.5, CH	5.34, d (3.5, 1H)
G2	74.4, CH	3.61, m (1H)	71.5, CH	3.63, m (1H)	71.7, CH	3.58, m (1H)
G3	76.3, CH	3.91, m (1H)	73.5, CH	3.92, m (1H)	73.5, CH	3.89, m (1H)
G4	73.7, CH	3.61, m (1H)	70.8, CH	3.63, m (1H)	70.8, CH	3.59, m (1H)
G5	74.0, CH	3.91, m (1H)	71.1, CH	3.92, m (1H)	71.1, CH	3.89, m (1H)
G6	66.3, CH_2	3.86, m (2H)	63.4, CH_2	3.86, m (2H)	62.5, CH_2	3.83, m (2H)
H1	102.7, CH	5.29, d (3.4, 1H)	99.9, CH	5.32, d (3.4, 1H)	99.9, CH	5.30, d (3.4, 1H)
H2	74.4, CH	3.57, m (1H)	71.6, CH	3.61, m (1H)	71.5, CH	3.57, m (1H)
H3	75.4, CH	3.61, m (1H)	72.5, CH	3.65, m (1H)	72.6, CH	3.57, m (1H)
H4	67.8, CH	2.43, t (9.0, 1H)	64.9, CH	2.45, t (9.0, 1H)	64.9, CH	2.43, t (9.0, 1H)
H5	72.4, CH	3.74, m (1H)	69.6, CH	3.78, m (1H)	69.6, CH	3.73, m (1H)
H6	20.2, CH_3	1.31, d (5.6, 3H)	17.3, CH_3	1.34, d (6.0, 3H)	17.3, CH_3	1.30, d (5.6, 3H)
I1	58.8, CH	3.49, m (1H)	56.0, CH	3.53, m (1H)	56.0, CH	3.45, m (1H)
I2	75.6, CH	3.67, m (1H)	72.7, CH	3.66, m (1H)	72.7, CH	3.63, m (1H)
I3	75.8, CH	3.77, m (1H)	72.9, CH	3.78, m (1H)	72.9, CH	3.73, m (1H)
I4	73.8, CH	4.01, d (12.0, 1H)	70.9, CH	4.04, d (12.0, 1H)	70.9, CH	4.01, d (12.0, 1H)
I5	141.8, C		138.9, C		138.9, C	
I6a<br>I6b	64.4, CH_2	4.09, m (1H)<br>4.19, m (1H)	61.6, CH_2	4.13, m (1H)<br>4.22, m (1H)	61.6, CH_2	4.09, m (1H)<br>4.19, m (1H)
I7	126.6, CH	5.86, d (3.3, 1H)	123.8, CH	5.90, d (3.4, 1H)	123.8, CH	5.87, d (3.5, 1H)
1’	180.2, C=O		176.7, C=O		179.8, C=O	
2’	30.0, CH_2	2.43, m (2H)	35.6, CH_2	2.45, m (2H)	40.9, CH	2.52, m (1H)
3’	11.1, CH_3	1.09, d (7.0, 3H)	17.9, CH_2	1.18, m (1H)<br>1.65, m (1H)	26.4, CH_2	1.51, m (1H)<br>1.63, m (1H)
4’			12.9, CH_3	0.94, t (7.2, 3H)	10.9, CH_3	0.88, t (7.0, 3H)
5’					15.7, CH_3	1.13, d (7.0, 3H)

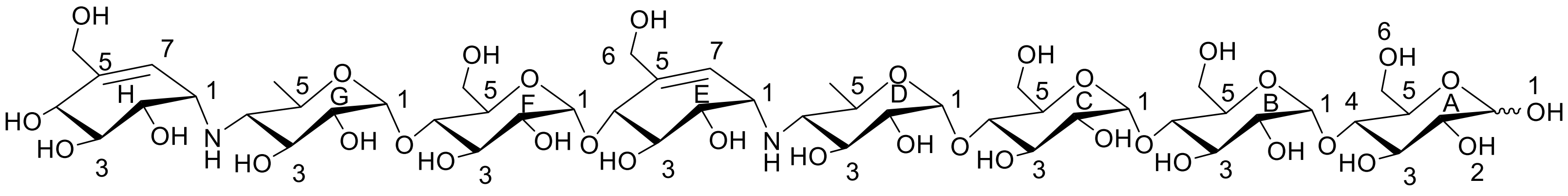

10

Table S3. ^{1}H (500 MHz) and ^{13}C (125 MHz) NMR data of **10** (δ ppm) in D_2O.

No.	δ_C	δ_H	HMBC ($^1H\rightarrow^{13}C$)	COSY ($^1H\rightarrow^1H$)	TOCSY ($^1H\rightarrow^1H$)
A1α	93.4, CH	5.23, d (3.5, 1H)	A2α, A5α	A2α	A2α, A5α
A2α	71.2, CH	3.57, m (1H)		A1α, A3α	A1α, A3α
A3α	73.2, CH	4.09, m (1H)		A2α, A4α	A2α, A4α
A4α	76.8, CH	3.68, m (1H)	B1	A3α, A5α	A3α, A5α
A5α	70.0, CH	3.97, m (1H)		A4α, A6α	A4α, A6α
A6α	60.5, CH_2	3.85, m (2H)		A5α	A5α
A1β	95.8, CH	4.65, d (8.0, 1H)	A2β, A3β, A5β	A2β	A2β, A3β, A5β
A2β	74.0, CH	3.27, t (9.0, 1H)		A1β, A3β	A1β, A3β
A3β	76.2, CH	3.76, m (1H)		A2β, A4β	A2β, A4β
A4β	76.9, CH	3.65, m (1H)	B1	A3β	A3β, A5β
A5β	74.6, CH	3.60, m (1H)		A4β, A6β	A4β, A6β
A6β	60.5, CH_2	3.90, m (2H)		A5β	A5β
B1	99.5, CH	5.41, d (3.5, 1H)	B2, B3	B2	B2, B3, B5
B2	71.5, CH	3.62, m (1H)		B1, B3	B1, B3
B3	73.4, CH	3.95, m (1H)		B2, B4	B2, B4
B4	77.0, CH	3.67, m (1H)	C1	B3, B5	B3, B5
B5	71.2, CH	3.85, m (1H)		B4	B4
B6	60.7, CH_2	3.85, m (2H)		overlapped	overlapped
C1	99.4, CH	5.41, d (3.5, 1H)	C2, C3	C2	C2, C3
C2	71.0, CH	3.66, m (1H)		C1, C3	C1, C3
C3	73.3, CH	3.83, m (1H)		C2, C4	C2, C4
C4	77.0, CH	3.68, m (1H)	D1	C3, C5	C3, C5
C5	69.4, CH	4.04, d (12.0, 1H)		C4, C6a, C6b	C4, C6a, C6b
C6a	63.2, CH_2	4.15, m (1H)		C5, C6b	C5, C6b
C6b		4.23, d (12.0, 1H)		C5, C6a	C5, C6a
D1	99.9, CH	5.33, d (3.5, 1H)	D2, D5	D2	D2, D3, D5
D2	71.2, CH	3.56, m (1H)		D1, D3	D1, D3
D3	72.7, CH	3.59, m (1H)		D2, D4	D2, D4
D4	64.2, CH	2.48, t (9.0, 1H)	D1, D3, D5, E1	D3, D5	D3, D5, D6

D5	69.7, CH	3.76, m (1H)		D4, D6	D4, D6
D6	17.4, CH_3	1.34, d (5.7, 3H)	D4, D5	D5	D3, D4, D5
E1	55.0, CH	3.54, m (1H)	E6	E2, E7	E2, E7
E2	70.7, CH	3.82, m (1H)		E1, E3	E1, E3
E3	70.8, CH	4.13, m (1H)		E2, E4	E2, E4
E4	76.1, CH	4.22, m (1H)	F1	E3	E3
E5	136.5, C				
E6a<br>E6b	62.0, CH_2	4.13, m (1H)<br>4.22, m (1H)		E6b<br>E6a	E6b<br>E6a
E7	126.3, CH	5.98, d (3.2, 1H)	E1, E2, E6	E1	E1, E2, E6b
F1	97.5, CH	5.38, d (3.4, 1H)		F2	F2
F2	72.9, CH	3.58, m (1H)		F1, F3	F1, F3
F3	73.5, CH	3.93, m (1H)		F2, F4	F2, F4
F4	70.8, CH	3.60, m (1H)	G1	F3, F5	F3, F5
F5	71.1, CH	3.95, m (1H)		F4, F6	F4, F6
F6	60.4, CH_2	3.81, m (2H)		F5	F5
G1	99.6, CH	5.33, d (3.4, 1H)	G2, G3	G2	G2, G3, G5
G2	71.3, CH	3.56, m (1H)		G1	G1, G3
G3	72.2, CH	3.59, m (1H)		G4	G4, G3
G4	64.9, CH	2.48, t (9.0, 1H)	H1	G3, G5	G3, G5, G6
G5	69.6, CH	3.86, m (1H)		G4, G6	G4, G6
G6	17.3, CH_3	1.34, d (5.7, 3H)	G4, G5	G5	G3, G4, G5
H1	56.0, CH	3.52, m (1H)	H6	H2, H7	H2, H7
H2	72.6, CH	3.72, m (1H)		H1, H3	H1, H3
H3	72.6, CH	3.75, m (1H)		H2, H4	H2, H4
H4	70.9, CH	4.07, d (12.0, 1H)		H3	H3
H5	139.0, C				
H6a<br>H6b	61.6, CH_2	4.12, m (2H)			
H7	123.7, CH	5.90, d (3.5, 1H)	H1, H2, H6	H1	H1, H2, H6a, H6b

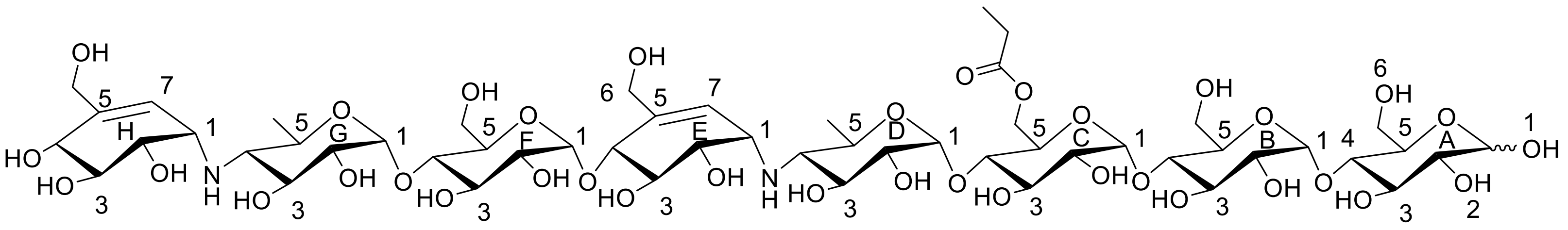

11

Table S4. ^{1}H (500 MHz) and ^{13}C (125 MHz) NMR data of **11** (δ ppm) in D_2O.

No.	δ_C	δ_H	HMBC ($^1H \rightarrow ^{13}C$)	COSY ($^1H \rightarrow ^1H$)	TOCSY ($^1H \rightarrow ^1H$)
A1α	95.3, CH	5.23, d (3.5, 1H)	A2α, A5α	A2α	A2α, A5α
A2α	74.8, CH	3.58, m (1H)		A1α, A3α	A1α, A3α
A3α	76.7, CH	3.97, m (1H)		A2α, A4α	A2α, A4α
A4α	80.2, CH	3.65, m (1H)	B1	A3α, A5α	A3α, A5α
A5α	73.3, CH	3.87, m (1H)		A4α	A4α, A6α
A6α	63.8, CH_2	3.87, m (2H)		overlapped	A5α
A1β	99.2, CH	4.66, d (8.0, 1H)	A2β, A5β	A2β	A2β, A3β, A5β
A2β	77.4, CH	3.28, t (9.0, 1H)		A1β, A3β	A1β, A3β
A3β	79.7, CH	3.77, m (1H)		A2β, A4β	A2β, A4β
A4β	80.2, CH	3.66, m (1H)	B1	A3β, A5β	A3β, A5β
A5β	77.9, CH	3.62, m (1H)		A4β, A6β	A4β, A6β
A6β	64.1, CH_2	3.91, m (2H)		A5β	A5β
B1	102.8, CH	5.41, d (3.5, 1H)	B3, B4, B5	B2	B2, B3, B5
B2	74.9, CH	3.64, m (1H)		B1, B3	B1, B3
B3	76.7, CH	3.96, m (1H)		B2, B4	B2, B4
B4	80.4, CH	3.67, m (1H)	C1	B3, B5	B3, B5
B5	74.6, CH	3.85, m (1H)		B4	B4
B6	63.9, CH_2	3.85, m (2H)		overlapped	overlapped
C1	103.0, CH	5.41, d (3.5, 1H)	C3, C5	C2	C2, C3
C2	74.5, CH	3.67, m (1H)		C1, C3	C1, C3
C3	76.6, CH	3.95, m (1H)		C2, C4	C2, C4
C4	81.3, CH	3.67, m (1H)	D1	C3, C5	C3, C5
C5	72.3, CH	4.05, d (12.0, 1H)		C4, C6a, C6b	C4, C6a, C6b
C6a	63.8, CH_2	4.22, m (1H)		C5, C6b	C5, C6b
C6b		4.44, d (12.0, 1H)		C5, C6a	C5, C6a
D1	104.0, CH	5.29, d (3.5, 1H)	D2, D5	D2	D2, D3, D5
D2	74.6, CH	3.56, m (1H)		D1, D3	D1, D3
D3	76.3, CH	3.59, m (1H)		D2, D4	D2, D4
D4	67.7, CH	2.48, t (9.0, 1H)	D3, D5, D6, E1	D3, D5	D2, D3, D5, D6

D5	73.2, CH	3.73, m (1H)		D4, D6	D4, D6
D6	20.7, CH_3	1.30, d (5.7, 3H)	D4, D5	D5	D3, D4, D5
E1	58.4, CH	3.53, m (1H)	E6	E2, E7	E2, E7
E2	73.0, CH	3.80, m (1H)		E1, E3	E1, E3
E3	74.1, CH	4.13, m (1H)		E2, E4	E2, E4
E4	79.6, CH	4.22, m (1H)	F1	E3	E3
E5	139.9, C				
E6a	65.4, CH_2	4.13, m (1H)		E6b	E6b
E6b		4.22, m (1H)		E6a	E6a
E7	129.7, CH	5.98, d (3.2, 1H)	E1, E2, E4, E6	E1	E1, E2, E6b
F1	101.0, CH	5.38, d (3.4, 1H)		F2	F2
F2	74.9, CH	3.62, m (1H)		F1, F3	F1, F3
F3	76.9, CH	3.93, m (1H)		F2, F4	F2, F4
F4	74.2, CH	3.64, m (1H)	G1	F3	F3, F5
F5	74.5, CH	3.93, m (1H)		F4, F6	F4, F6
F6	66.9, CH_2	3.81, m (2H)		F5	F5
G1	103.3, CH	5.32, d (3.4, 1H)	G2, G3	G2	G2, G4, G5
G2	74.7, CH	3.58, m (1H)		G1, G3	G1, G3
G3	75.9, CH	3.62, m (1H)		G2, G4	G2, G4
G4	68.3, CH	2.48, t (9.0, 1H)	H1	G3, G5	G2, G3, G5, G6
G5	73.0, CH	3.76, m (1H)		G4, G6	G4, G6
G6	20.8, CH_3	1.34, d (5.7, 3H)	G4, G5	G5	G3, G4, G5
H1	59.4, CH	3.54, m (1H)	H6	H2, H7	H2, H7
H2	76.1, CH	3.68, m (1H)		H1, H3	H1, H3
H3	76.3, CH	3.77, m (1H)		H2, H4	H2, H4
H4	74.3, CH	4.04, d (12.0, 1H)		H3	H3
H5	142.3, C				
H6a	65.0 CH_2	4.12, m (1H)		H6b	
H6b		4.22, m (1H)		H6a	
H7	127.2, CH	5.90, d (3.5, 1H)	H1, H2, H6	H1	H1, H2, H6a, H6b
1’	180.8, C=O				
2’	30.6, CH_2	2.48, m (2H)	1’, 3’	3’	3’
3’	11.7, CH_3	1.13, d (7.2, 3H)	2’, 3’	2’	2’

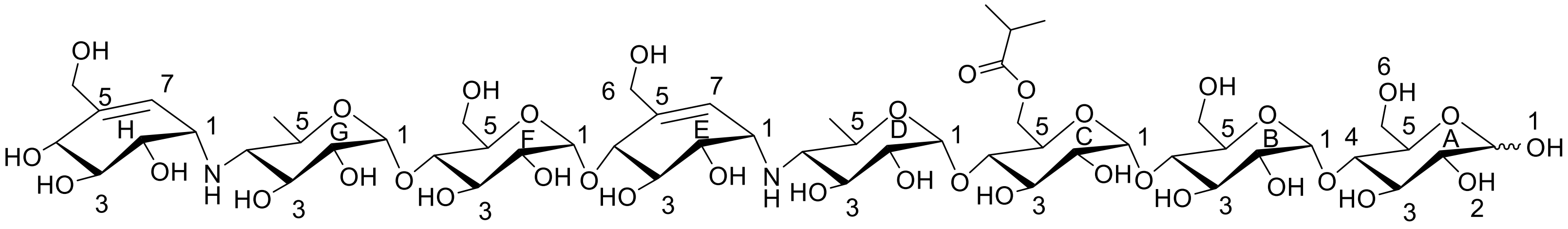

12

Table S5. ^{1}H (500 MHz) and ^{13}C (125 MHz) NMR data of **12** (δ ppm) in D_2O.

No.	δ_C	δ_H	HMBC ($^1H\rightarrow^{13}C$)	COSY ($^1H\rightarrow^1H$)	TOCSY ($^1H\rightarrow^1H$)
A1α	91.9, CH	5.24, d (3.4, 1H)	A2α, A5α	A2α	A2α, A5α
A2α	71.3, CH	3.52, m (1H)		A1α, A3α	A1α, A3α
A3α	73.3, CH	4.16, m (1H)		A2α, A4α	A2α, A4α
A4α	76.8, CH	3.69, m (1H)	B1	A3α, A5α	A3α, A5α
A5α	70.0, CH	3.90, m (1H)		A4α, A6α,	A4α, A6α
A6α	60.5, CH_2	3.74, m (2H)		A5α	A5α
A1β	95.8, CH	4.66, d (8.0, 1H)	A2β, A5β	A2β	A2β, A3β, A5β
A2β	74.0, CH	3.29, m (1H)		A1β, A3β	A1β, A3β
A3β	76.3, CH	3.78, m (1H)		A2β, A4β	A2β, A4β
A4β	76.8, CH	3.67, m (1H)	B1	A3β, A5β	A3β, A5β
A5β	74.6, CH	3.62, m (1H)		A4β, A6β	A4β, A6β
A6β	60.6, CH_2	3.92, m (2H)		A5β	A5β
B1	99.6, CH	5.38, d (3.5, 1H)	B3, B5	B2	B3, B4
B2	71.6, CH	3.58, m (1H)		B1, B3	B1, B3
B3	73.4, CH	3.94, m (1H)		B2, B4	B2, B4
B4	77.1, CH	3.63, m (1H)	C1	B3, B5	B3, B5
B5	71.2, CH	3.80, m (1H)		B4	B4
B6	60.7, CH_2	3.80, m (2H)		overlapped	overlapped
C1	99.4, CH	5.38, d (3.5, 1H)	C3, C5	C2	C2, C3
C2	71.1, CH	3.62, m (1H)		C1, C3	C1, C3
C3	73.2, CH	3.92, m (1H)		C2, C4	C2, C4
C4	78.2, CH	3.62, m (1H)	D1	C3, C5	C3, C5
C5	69.0, CH	4.03, d (12.0, 1H)		C4, C6a, C6b	C4, C6a, C6b
C6a	63.5, CH_2	4.20, m (1H)		C5, C6b	C5, C6b
C6b		4.45, d (12.0, 1H)		C5, C6a	C5, C6a
D1	100.7, CH	5.28, d (3.5, 1H)	D2, D5	D2	D2, D3, D4
D2	71.2, CH	3.54, m (1H)		D1	D1, D3
D3	73.0, CH	3.56, m (1H)		D4	D2, D4
D4	64.3, CH	2.47, t (8.8, 1H)	D3, D5, E1	D3, D5	D1, D2, D3, D5

D5	69.8, CH	3.71, m (1H)		D4, D6	D4, D6
D6	17.4, CH_3	1.31, d (5.6, 3H)	D4, D5	D5	D3, D4, D5
E1	55.0, CH	3.53, m (1H)	E6	E2, E7	E2, E7
E2	70.8, CH	3.80, m (1H)		E1, E3	E1, E3
E3	70.9, CH	4.14, m (1H)		E2, E4	E2, E4
E4	76.2, CH	4.23, m (1H)	F1	E3	E3
E5	136.5, C				
E6a	62.0, CH_2	4.14, m (1H)		E6b	E6b
E6b		4.23, m (1H)		E6a	E6a
E7	126.3, CH	5.98, d (3.1, 1H)	E2, E6	E1	E1, E2, E6b
F1	97.8, CH	5.42, d (3.5, 1H)		F2	F2
F2	73.2, CH	3.68, m (1H)		F1, F3	F1, F3
F3	73.5, CH	3.94, m (1H)		F2, F4	F2, F4
F4	70.9, CH	3.66, m (1H)	G1	F3, F5	F3, F5
F5	71.2, CH	3.96, m (1H)		F4, F6	F4, F6
F6	60.4, CH_2	3.82, m (2H)		F5	F5
G1	99.9, CH	5.33, d (3.4, 1H)	G2, G3	G2	G2, G4
G2	71.2, CH	3.61, m (1H)		G1	G1, G3
G3	72.6, CH	3.61, m (1H)		G4	G2, G4
G4	64.9, CH	2.47, t (8.8, 1H)	G3, G5, H1	G3, G5	G2, G3, G5
G5	69.6, CH	3.77, m (1H)		G4, G6	G4, G6
G6	17.3, CH_3	1.35, d (5.6, 3H)	G4, G5	G5	G3, G4, G5
H1	56.0, CH	3.54, m (1H)	H6	H2, H7	H2, H7
H2	72.7, CH	3.69, m (1H)		H1, H3	H1, H3
H3	72.9, CH	3.77, m (1H)		H2, H4	H2, H4
H4	71.0, CH	4.06, d (12.0, 1H)		H3	H3
H5	139.0, C				
H6a	61.6, CH_2	4.13, m (1H)		H6b	
H6b		4.25, m (1H)		H6a	
H7	123.8, CH	5.91, d (3.4, 1H)	H1, H2, H6	H1	H1, H2, H6a, H6b
1’	180.1, C=O				
2’	33.9, CH	2.72, m (2H)	1’, 3’, 4’	3’	3’, 4’
3’	18.3, CH_3	1.19, d (3.0, 3H)	1, 2’, 4’	2’	2’
4’	18.2, CH_3	1.19, d (3.0, 3H)	1, 2’, 3’	Overlapped	2’

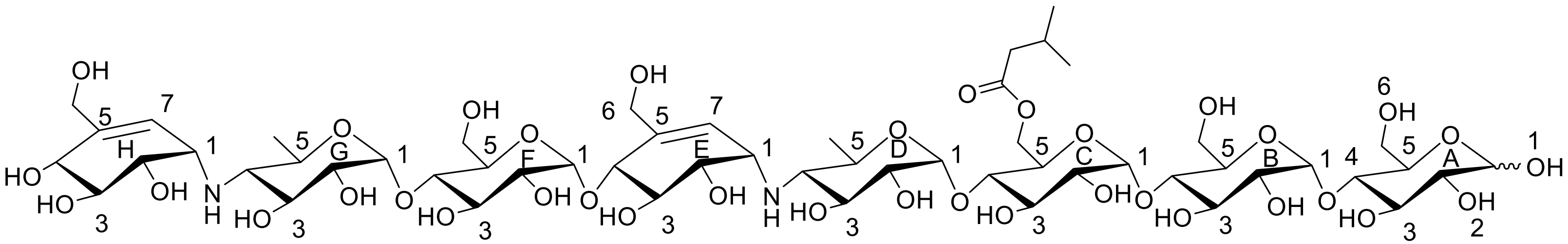

13

Table S6. ^{1}H (500 MHz) and ^{13}C (125 MHz) NMR data of **13** (δ ppm) in D_2O.

No.	δ_C	δ_H	HMBC ($^1H \rightarrow ^{13}C$)	COSY ($^1H \rightarrow ^1H$)	TOCSY ($^1H \rightarrow ^1H$)
A1α	92.0, CH	5.24, d (3.5, 1H)	A2α, A5α	A2α	A2α, A5α
A2α	71.4, CH	3.51, m (1H)		A1α, A3α	A1α, A3α
A3α	73.4, CH	3.92, m (1H)		A2α, A4α	A2α, A4α
A4α	76.9, CH	3.69, m (1H)	B1	A3α, A5α	A3α, A5α
A5α	70.0, CH	3.82, m (1H)		A4α, A6α,	A4α, A6α
A6α	60.5, CH_2	3.79, m (2H)		A5α	A5α
A1β	95.8, CH	4.66, d (7.8, 1H)	A2β, A5β	A2β	A2β, A4β, A5β
A2β	74.0, CH	3.28, m (1H)		A1β, A3β	A1β, A3β
A3β	76.2, CH	3.77, m (1H)		A2β, A4β	A2β, A4β
A4β	76.8, CH	3.66, m (1H)	B1	A3β, A5β	A3β, A5β
A5β	74.6, CH	3.60, m (1H)		A4β, A6β	A4β, A6β
A6β	60.5, CH_2	3.90, m (2H)		A5β	A5β
B1	99.4, CH	5.41, d (3.5, 1H)	B2, B3, B5	B2	B2, B3, B5
B2	71.6, CH	3.62, m (1H)		B1, B3	B1, B3
B3	73.2, CH	3.96, m (1H)		B2, B4	B2, B4
B4	77.1, CH	3.64, m (1H)	C1	B3, B5	B3, B5
B5	71.3, CH	3.85, m (1H)		B4	B4
B6	60.7, CH_2	3.85, m (2H)		overlapped	overlapped
C1	99.6, CH	5.41, d (3.5, 1H)	C2, C3, C5	C2	C2, C3
C2	71.2, CH	3.62, m (1H)		C1, C3	C1, C3
C3	73.2, CH	3.96, m (1H)		C2, C4	C2, C4
C4	78.0, CH	3.64, m (1H)	D1	C3, C5	C3, C5
C5	69.0, CH	4.05, d (12.0, 1H)		C4, C6a, C6b	C4, C6a, C6b
C6a	63.4, CH_2	4.23, m (1H)		C5, C6b	C5, C6b
C6b		4.49, d (12.0, 1H)		C5, C6a	C5, C6a
D1	100.6, CH	5.29, d (3.5, 1H)	D2, D5	D2	D2, D4, D5
D2	71.3, CH	3.58, m (1H)		D1	D1
D3	73.0, CH	3.60, m (1H)		D4	D4

D4	64.3, CH	2.47, t (9.0, 1H)	D3, D5, D6, E1	D3, D5	D1, D3, D5, D6
D5	69.8, CH	3.75, m (1H)		D4, D6	D4, D6
D6	17.4, CH_3	1.32, d (5.6, 3H)	D4, D5	D5	D3, D4, D5
E1	55.0, CH	3.48, m (1H)	E6	E2, E7	E2, E7
E2	69.7, CH	3.79, m (1H)		E1, E3	E1, E3
E3	70.8, CH	4.14, m (1H)		E2, E4	E2, E4
E4	76.4, CH	4.23, m (1H)	F1	E3	E3
E5	136.5, C				
E6a	62.0, CH_2	4.14, m (1H)		E6b	E6b
E6b		4.23, m (1H)		E6a	E6a
E7	126.4, CH	5.99, d (3.2, 1H)	E2, E6	E1	E1, E2, E6b
F1	97.6, CH	5.38, d (3.5, 1H)		F2	F2, F5
F2	72.7, CH	3.61, m (1H)		F1, F3	F1, F3
F3	73.5, CH	3.93, m (1H)		F2	F2, F4
F4	70.9, CH	3.61, m (1H)	G1	F3	F3, F5
F5	71.2, CH	3.93, m (1H)		F4, F6	F4, F6
F6	60.7, CH_2	3.82, m (2H)		F5	F5
G1	100.0, CH	5.33, d (3.4, 1H)	G2, G3	G2	G2, G4
G2	71.5, CH	3.58, m (1H)		G1	G1, G3
G3	72.6, CH	3.60, m (1H)		G4	G2, G4
G4	65.0, CH	2.47, t (9.0, 1H)	G3, G5 ,H1	G3, G5	G1, G2, G5, G6
G5	69.6, CH	3.75, m (1H)		G4, G6	G4, G6
G6	17.4, CH_3	1.35, d (5.6, 3H)	G4, G5	G5	G3, G4, G5
H1	56.0, CH	3.48, m (1H)	H6	H2, H7	H2, H7
H2	72.8, CH	3.67, m (1H)		H1, H3	H1, H3
H3	73.0, CH	3.76, m (1H)		H2, H4	H2, H4
H4	71.0, CH	4.05, d (12.0, 1H)		H3	H3
H5	139.0, C				
H6a	61.6, CH_2	4.12, m (1H)		H6b	
H6b		4.23, m (1H)		H6a	
H7	123.8, CH	5.91, d (3.4, 1H)	H1, H2, H6	H1	H1, H2, H6a, H6b
1’	176.2, C=O				
2’	42.9, CH_2	2.36, m (2H)	1’, 3’, 4’	3’	3’, 4’
3’	25.5, CH	2.08, m (1H)	1, 2’, 4’	2’, 4’	2’, 4’
4’	21.7, CH_3	0.96, d (6.6, 3H)	2’, 3’	3’	3’
5’	21.7, CH_3	0.96, d (6.6, 3H)		overlapped	overlapped

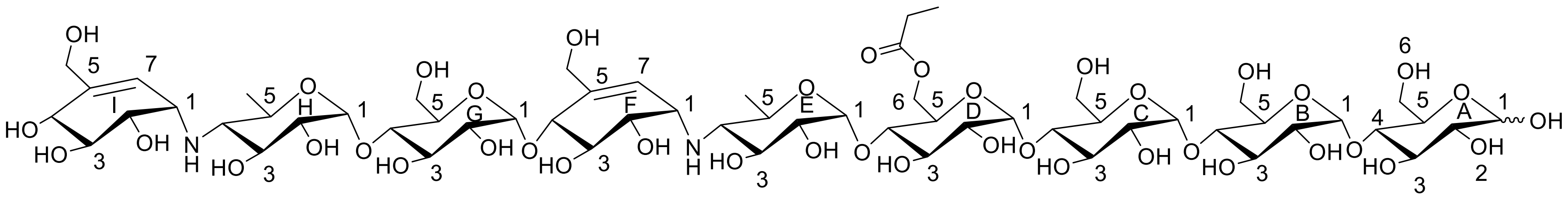

14

Table S7. ^{1}H (500 MHz) and ^{13}C (125 MHz) NMR data of **14** (δ ppm) in D_2O.

No.	δ_C	δ_H	HMBC ($^1H \rightarrow ^{13}C$)	COSY ($^1H \rightarrow ^1H$)	TOCSY ($^1H \rightarrow ^1H$)
A1α	94.7, CH	5.20, d (3.5, 1H)	A2α, A5α	A2α	A2α, A5α
A2α	74.2, CH	3.53, m (1H)		A1α, A3α	A1α, A3α
A3α	76.1, CH	3.93, m (1H)		A2α, A4α	A2α, A4α
A4α	79.6, CH	3.61, m (1H)	B1	A3α, A5α	A3α, A5α
A5α	72.8, CH	3.85, m (1H)		A4α, A6α,	A4α, A6α,
A6α	63.2, CH_2	3.78, m (2H)		A5α	A5α
A1β	98.6, CH	4.62, d (8.0, 1H)	A5β	A2β	A2β, A3β, A5β
A2β	76.8, CH	3.24, t (9.0, 1H)		A1β, A3β	A1β, A3β
A3β	79.1, CH	3.74, m (1H)		A2β, A4β	A2β, A4β
A4β	79.7, CH	3.63, m (1H)	B1	A3β	A3β,
A5β	77.4, CH	3.61, m (1H)		A6β	A6β
A6β	63.3, CH_2	3.88, m (2H)		A5β	A5β
B1	102.2, CH	5.38, d (3.5, 1H)	B2, B3, B5	B2	B2, B3
B2	74.4, CH	3.61, m (1H)		B1, B3	B1, B3
B3	76.2, CH	3.93, m (1H)		B2, B4	B2, B4
B4	79.9, CH	3.63, m (1H)	C1	B3, B5	B3, B5
B5	74.0, CH	3.82, m (1H)		B4	B4
B6	63.4, CH_2	3.82, m (2H)		overlapped	overlapped
C1	102.3, CH	5.38, d (3.5, 1H)	C2, C3, C5	C2	C2, C3
C2	74.3, CH	3.61, m (1H)		C1, C3	C1, C3
C3	76.1, CH	3.93, m (1H)		C2, C4	C2, C4
C4	79.9, CH	3.63, m (1H)	D1	C3, C5	C3, C5
C5	74.1, CH	3.82, m (1H)		C4	C4
C6	63.5, CH_2	3.82, m (2H)		overlapped	overlapped
D1	102.4, CH	5.38, d (3.5, 1H)	D2, D3, D5	D2	D2, D3
D2	73.9, CH	3.63, m (1H)		D1, D3	D1, D3
D3	76.0, CH	3.93, m (1H)		D2	D2
D4	80.7, CH	3.63, m (1H)	E1	D3, D5	D3, D5
D5	71.7, CH	4.01, d (12.0, 1H)		D4, D6a, D6b	D4, D6a, D6b

D6a	67.1, CH_2	4.19, m (1H)		D5, D6b	D5, D6b
D6b		4.44, d (12.0, 1H)		D5, D6a	D5, D6a
E1	103.4, CH	5.25, d (3.5, 1H)	E3, E5	E2	E2, E4
E2	74.1, CH	3.57, m (1H)		E1, E3	E1, E3, E4, E6
E3	75.8, CH	3.61, m (1H)		E2, E4	E2, E4
E4	67.8, CH	2.43, t (9.0, 1H)	E2, E5, E6, F1	E3, E5	E3, E5, E6
E5	72.6, CH	3.73, m (1H)		E4, E6	E4, E6
E6	20.2, CH_3	1.27, d (5.6, 3H)	E4, E5	E5	E4, E5
F1	57.8, CH	3.49, m (1H)	F2, F6	F2, F7	F2, F3, F7
F2	72.5, CH	3.77, m (1H)		F1, F3	F1, F3
F3	73.6, CH	4.09, m (1H)		F2, F4	F2, F4
F4	79.0, CH	4.19, m (1H)	G1	F3	F3
F5	139.3, C				
F6a	64.8, CH_2	4.09, m (1H)		F6b	F6b
F6b		4.19, m (1H)		F6a	F6a
F7	129.1, CH	5.94, d (3.2, 1H)	F2, F6	F1	F1, F2, F4
G1	100.4, CH	5.34, d (3.4, 1H)	G3, G5	G2	G2, G5
G2	74.4, CH	3.61, m (1H)		G1, G3	G1, G3
G3	76.3, CH	3.91, m (1H)		G2	G2
G4	73.7, CH	3.61, m (1H)	H1	G3	G3
G5	74.0, CH	3.91, m (1H)		G4, G6	G4, G6
G6	66.3, CH_2	3.86, m (2H)		G5	G5
H1	102.7, CH	5.29, d (3.4, 1H)	H3, H5	H2	H2
H2	74.4, CH	3.57, m (1H)		H1, H3	H1, H3, H4, H6
H3	75.4, CH	3.61, m (1H)		H2, H4	H2, H4
H4	67.8, CH	2.43, t (9.0, 1H)	H1, H2, H5, H6	H3, H5	H3, H5, H6
H5	72.4, CH	3.74, m (1H)		H4, H6	H4, H6
H6	20.2, CH_3	1.31, d (5.6, 3H)	H4, H5	H5	H4, H5
I1	58.8, CH	3.49, m (1H)	I2, I6	I2, I7	I2, I3, I7
I2	75.6, CH	3.67, m (1H)		I1, I3	I1, I3
I3	75.8, CH	3.77, m (1H)		I2, I4	I2, I4
I4	73.8, CH	4.01, d (12.0, 1H)		I3	I3
I5	141.8, C				
I6a	64.4, CH_2	4.09, m (1H)		I6b	I6b
I6b		4.19, m (1H)		I6a	I6a
I7	126.6, CH	5.86, d (3.3, 1H)	I1, I2, I6	I1	I1
1’	180.2, C=O				
2’	30.0, CH_2	2.43, m (2H)	1’, 3’	3’	3’
3’	11.1, CH_3	1.09, d (7.0, 3H)	1’, 2’	2’	2’

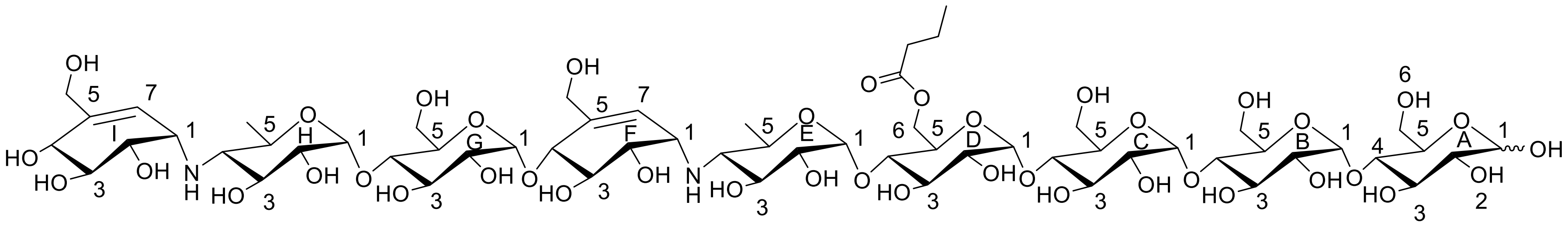

15

Table S8. ^{1}H (500 MHz) and ^{13}C (125 MHz) NMR data of **15** (δ ppm) in D_2O.

No.	δ_C	δ_H	HMBC ($^1H \rightarrow ^{13}C$)	COSY ($^1H \rightarrow ^1H$)	TOCSY ($^1H \rightarrow ^1H$)
A1α	91.9, CH	5.22, d (3.5, 1H)	A2α, A5α	A2α	A2α, A5α
A2α	71.4, CH	3.52, m (1H)		A1α, A3α	A1α, A3α
A3α	73.3, CH	3.93, m (1H)		A2α, A4α	A2α, A4α
A4α	76.8, CH	3.62, m (1H)	B1	A3α, A5α	A3α, A5α
A5α	69.9, CH	3.85, m (1H)		A4α, A6α,	A4α, A6α,
A6α	60.4, CH_2	3.79, m (2H)		A5α	A5α
A1β	95.8, CH	4.65, d (8.0, 1H)	A2β, A5β	A2β	A2β, A3β, A5β
A2β	74.0, CH	3.27, m (1H)		A1β, A3β	A1β, A3β
A3β	76.3, CH	3.77, m (1H)		A2β, A4β	A2β, A4β
A4β	76.8, CH	3.65, m (1H)	B1	A3β, A5β	A3β, A5β
A5β	74.5, CH	3.60, m (1H)		A4β, A6β	A4β, A6β
A6β	60.5, CH_2	3.89, m (2H)		A5β	A5β
B1	99.4, CH	5.40, d (3.5, 1H)	B2, B3, B5	B2	B2, B3, B5
B2	71.5, CH	3.60, m (1H)		B1, B3	B1, B3
B3	73.3, CH	3.95, m (1H)		B2, B4	B2, B4
B4	77.0, CH	3.64, m (1H)	C1	B3, B5	B3, B5
B5	71.2, CH	3.82, m (1H)		B4	B4
B6	60.7, CH_2	3.84, m (2H)		overlapped	overlapped
C1	99.5, CH	5.40, d (3.5, 1H)	C2, C3, C5	C2	C2, C3, C5
C2	71.5, CH	3.60, m (1H)		C1, C3	C1, C3
C3	73.2, CH	3.95, m (1H)		C2, C4	C2, C4
C4	77.0, CH	3.64, m (1H)	D1	C3, C5	C3, C5
C5	71.2, CH	3.82, m (1H)		C4	C4
C6	60.8, CH_2	3.84, m (2H)		overlapped	overlapped
D1	99.6, CH	5.40, d (3.5, 1H)	D2, D3, D5	D2	D2, D3, D5
D2	71.1, CH	3.63, m (1H)		D1, D3	D1, D3
D3	73.2, CH	3.94, m (1H)		D2	D2
D4	77.9, CH	3.63, m (1H)	E1	D3, D5	D3, D5

D5	68.9, CH	4.04, d (12.0, 1H)		D4, D6a, D6b	D4, D6a, D6b
D6a	64.3, CH_2	4.22, m (1H)		D5, D6b	D5, D6b
D6b		4.45, dd (12.0, 3.0, 1H)		D5, D6a	D5, D6a
E1	100.5, CH	5.28, d (3.4, 1H)	E3, E5	E2	E2, E3, E4
E2	71.3, CH	3.53, m (1H)		E1, E3	E1, E3
E3	72.9, CH	3.58, m (1H)		E2, E4	E1, E2, E4, E6
E4	64.9, CH	2.45, t (9.0, 1H)	E3, E5, E6, F1	E3, E5	E3, E5, E6
E5	69.8, CH	3.73, m (1H)		E4, E6	E4, E6
E6	17.4, CH_3	1.30, d (6.0, 3H)	E4, E5	E5	E4, E5
F1	55.0, CH	3.53, m (1H)	F2, F5	F2, F7	F2, F7
F2	69.7, CH	3.79, m (1H)		F1, F3	F1, F3
F3	70.8, CH	4.13, m (1H)		F2, F4	F2, F4
F4	76.2, CH	4.22, m (1H)	G1	F3	F3
F5	136.5, C				
F6a	62.0, CH_2	4.13, m (1H)		F6b	F6b
F6b		4.22, m (1H)		F6a	F6a
F7	126.3, CH	5.97, d (3.2, 1H)	F2, F6	F1	F1, F2, F6b
G1	97.6, CH	5.37, d (3.6, 1H)	G3, G5	G2	G2, G5
G2	71.5, CH	3.63, m (1H)		G1, G3	G1, G3
G3	73.5, CH	3.92, m (1H)		G2	G2
G4	70.8, CH	3.63, m (1H)	H1	G3	G3
G5	71.1, CH	3.92, m (1H)		G4, G6	G4, G6
G6	63.4, CH_2	3.86, m (2H)		G5	G5
H1	99.9, CH	5.32, d (3.4, 1H)	H3, H5	H2	H2, H4, H5
H2	71.6, CH	3.61, m (1H)		H1, H3	H1, H3
H3	72.5, CH	3.65, m (1H)		H2, H4	H1, H2, H4, H6
H4	64.9, CH	2.45, t (9.0, 1H)	H3, H5, H6	H3, H5	H3, H5, H6
H5	69.6, CH	3.78, m (1H)		H4, H6	H4, H6
H6	17.3, CH_3	1.34, d (6.0, 3H)	H4, H5	H5	H2, H4, H5
I1	56.0, CH	3.53, m (1H)	I2, I6	I2, I7	I2, I7
I2	72.7, CH	3.66, m (1H)		I1, I3	I1, I3
I3	72.9, CH	3.78, m (1H)		I2, I4	I2, I4
I4	70.9, CH	4.04, d (12.0, 1H)		I3	I3
I5	138.9, C				
I6a	61.6, CH_2	4.13, m (1H)		I6b	I6b
I6b		4.22, m (1H)		I6a	I6a
I7	123.8, CH	5.90, d (3.4, 1H)	I1, I2, I6	I1	I1, I6a, I6b
1’	176.7, C=O				
2’	35.6, CH_2	2.45, m (2H)	1’, 3’, 4’	3’b	3’b, 4’
3’a	17.9, CH_2	1.18, m (1H)	2’		2’
3’b		1.65, m (1H)	1’, 2’, 4’	2’, 4’	2’, 4’
4’	12.9, CH_3	0.94, t (7.2, 3H)	2’, 3’	3’b	2’, 3’b

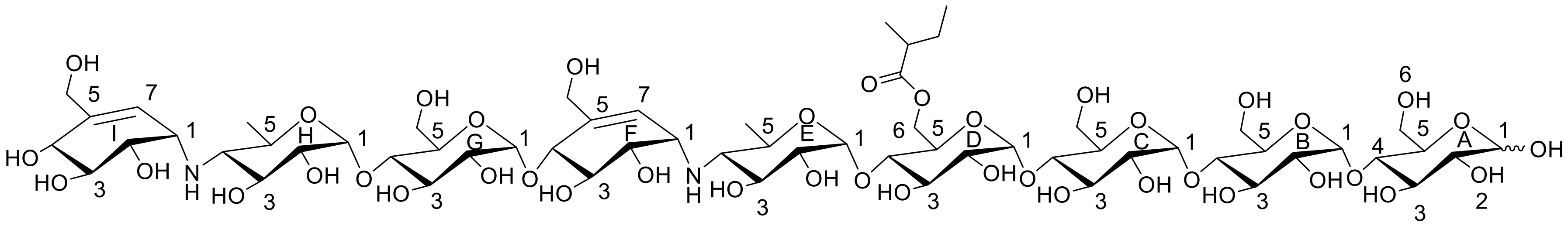

16

Table S9. ^{1}H (500 MHz) and ^{13}C (125 MHz) NMR data of **16** (δ ppm) in D_2O.

No.	δ_C	δ_H	HMBC ($^1H \rightarrow ^{13}C$)	COSY ($^1H \rightarrow ^1H$)	TOCSY ($^1H \rightarrow ^1H$)
A1α	91.9, CH	5.21, d (3.5, 1H)	A2α, A5α	A2α	A2α, A5α
A2α	71.3, CH	3.50, m (1H)		A1α, A3α	A1α, A3α
A3α	73.3, CH	3.92, m (1H)		A2α, A4α	A2α, A4α
A4α	76.8, CH	3.64, m (1H)	B1	A3α, A5α	A3α, A5α
A5α	69.9, CH	3.84, m (1H)		A4α, A6α,	A4α, A6α,
A6α	60.4, CH_2	3.78, m (2H)		A5α	A5α
A1β	95.8, CH	4.63, d (8.0, 1H)	A2β, A3β	A2β	A3β, A5β
A2β	74.0, CH	3.25, m (1H)		A1β, A3β	A1β, A3β
A3β	76.2, CH	3.73, m (1H)		A2β, A4β	A2β, A4β
A4β	76.8, CH	3.64, m (1H)	B1	A3β, A5β	A3β, A5β
A5β	74.5, CH	3.61, m (1H)		A4β, A6β	A4β, A6β
A6β	60.4, CH_2	3.88, m (2H)		A5β	A5β
B1	99.4, CH	5.38, d (3.5, 1H)	B2, B4, B5	B2	B2, B3, B5
B2	71.6, CH	3.59, m (1H)		B1, B3	B1, B3
B3	73.2, CH	3.94, m (1H)		B2, B4	B2, B4
B4	77.0, CH	3.64, m (1H)	C1	B3, B5	B3, B5
B5	71.2, CH	3.82, m (1H)		B4	B4
B6	60.5, CH_2	3.82, m (2H)		overlapped	overlapped
C1	99.5, CH	5.38, d (3.5, 1H)	C2, C4, C5	C2	C2, C3, C5
C2	71.5, CH	3.59, m (1H)		C1, C3	C1, C3
C3	73.3, CH	3.94, m (1H)		C2, C4	C2, C4
C4	76.9, CH	3.64, m (1H)	D1	C3, C5	C3, C5
C5	71.2, CH	3.82, m (1H)		C4	C4
C6	60.7, CH_2	3.82, m (2H)		overlapped	overlapped
D1	99.6, CH	5.38, d (3.5, 1H)	D2, D4, D5	D2	D2, D3, D5
D2	71.1, CH	3.64, m (1H)		D1, D3	D1, D3
D3	73.1, CH	3.93, m (1H)		D2	D2
D4	78.4, CH	3.64, m (1H)	E1	D3, D5	D3, D5

D5	69.0, CH	4.01, d (12.0, 1H)		D4, D6a, D6b	D4, D6a, D6b
D6a	63.4, CH_2	4.19, m (1H)		D5, D6b	D5, D6b
D6b		4.43, d (12.0, 1H)		D5, D6a	D5, D6a
E1	100.7, CH	5.24, d (3.5, 1H)	E3, E5	E2	E2, E3, E4
E2	71.3, CH	3.50, m (1H)		E1, E3	E1, E3
E3	72.9, CH	3.52, m (1H)		E2, E4	E1, E2, E4
E4	64.3, CH	2.43, t (9.0, 1H)	E3, E5, E6, F1	E3, E5	E3, E5, E6
E5	69.7, CH	3.71, m (1H)		E4, E6	E4, E6
E6	17.4, CH_3	1.29, d (5.6, 3H)	E4, E5	E5	E4, E5
F1	55.0, CH	3.45, m (1H)	F2, F5	F2, F7	F2, F7
F2	69.7, CH	3.76, m (1H)		F1, F3	F1, F3
F3	70.8, CH	4.09, m (1H)		F2, F4	F2, F4
F4	76.3, CH	4.19, m (1H)	G1	F3	F3
F5	136.5, C				
F6a	62.0, CH_2	4.09, m (1H)		F6b	F6b
F6b		4.19, m (1H)		F6a	F6a
F7	126.6, CH	5.94, d (3.1, 1H)	F2, F6	F1	F1, F2, F6b
G1	97.5, CH	5.34, d (3.5, 1H)	G3, G5	G2	G2, G5
G2	71.7, CH	3.58, m (1H)		G1, G3	G1, G3
G3	73.5, CH	3.89, m (1H)		G2	G2
G4	70.8, CH	3.59, m (1H)	H1	G3	G3
G5	71.1, CH	3.89, m (1H)		G4, G6	G4, G6
G6	62.5, CH_2	3.83, m (2H)		G5	G5
H1	99.9, CH	5.30, d (3.4, 1H)	H3, H5	H2	H2, H4, H5
H2	71.5, CH	3.57, m (1H)		H1	H1
H3	72.6, CH	3.57, m (1H)		H4	H1, H4, H6
H4	64.9, CH	2.43, t (9.0, 1H)	H3, H5, H6	H3, H5	H3, H5, H6
H5	69.6, CH	3.73, m (1H)		H4, H6	H4, H6
H6	17.3, CH_3	1.30, d (5.6, 3H)	H4, H5	H5	H2, H4, H5
I1	56.0, CH	3.45, m (1H)	I2, I6	I2, I7	I2, I7
I2	72.7, CH	3.63, m (1H)		I1, I3	I1, I3
I3	72.9, CH	3.73, m (1H)		I2, I4	I2, I4
I4	70.9, CH	4.01, d (12.0, 1H)		I3	I3
I5	138.9, C				
I6a	61.6, CH_2	4.09, m (1H)		I6b	I6b
I6b		4.19, m (1H)		I6a	I6a
I7	123.8, CH	5.87, d (3.5, 1H)	I1, I2, I6	I1	I1, I6a, I6b
1’	179.8, C=O				
2’	40.9, CH	2.52, m (1H)	3’, 4’, 5’	3’b, 5’	3’a, 3’b, 4’, 5’
3’a	26.4, CH_2	1.51, m (1H)	2’, 4’, 5’	4’	2’, 3’b, 4’, 5’
3’b		1.63, m (1H)	2’, 4’, 5’	2’, 4’	2’, 3’a, 4’, 5’
4’	10.9, CH_3	0.88, t (7.0, 3H)	2’, 3’	3’a, 3’b	3’a, 3’b, 5’
5’	15.7, CH_3	1.13, d (7.0, 3H)	2’, 3’	2’	2’, 4’

Table S10. Acarviostatins with glucose(s) at the reducing terminus (Aca-glu) from *Streptomyces* sp. HO1518.

No.	Compounds	Formula	Δ (ppm)	t_R (min)	$[M + H]^+$	Characterized fragment ions	Ref.
1	Aca I01	$C_{25}H_{43}NO_{18}$	0.2	6.00	646.2553	304.1392, 646.2554	1
2	Aca I02	$C_{31}H_{53}NO_{23}$	1.7	6.14	808.3081	304.1395, 808.3095	2
3	Aca I03 (**17**)	$C_{37}H_{63}NO_{28}$	0.1	6.55	970.3609	304.1397, 970.3610	3
4	Aca II00	$C_{38}H_{64}N_2O_{25}$	1.4	6.82	949.3871	304.1398, 769.3251, 949.3884	4
5	Aca II01	$C_{44}H_{74}N_2O_{30}$	–0.4	6.93	1111.4399	304.1401, 769.3245, 808.3090, 1111.4395	5
6	Aca II02 (**10**)	$C_{50}H_{84}N_2O_{35}$	0.2	7.81	1273.4927	304.1394, 769.3244, 970.3609, 1273.4929	–
7	Aca II03 (**9**)	$C_{56}H_{94}N_2O_{40}$	–2.0	9.27	1435.5456	304.1386, 769.3219, 1132.4094, 1435.5427	3
8	Aca II04	$C_{62}H_{104}N_2O_{45}$	0.1	7.74	1597.5984	304.1389, 769.3233, 1294.4610, 1597.5986	–
9	Aca II05	$C_{68}H_{114}N_2O_{50}$	–1.2	9.18	1759.6512	304.1389, 769.3233, 1759.6491	–
10	Ac-Aca I01	$C_{27}H_{45}NO_{19}$	–0.1	10.71	688.2659	304.1396, 688.2658	–
11	Ac-Aca I02	$C_{33}H_{55}NO_{24}$	–1.9	10.93	850.3187	304.1393, 850.3171	–
12	Ac-Aca I03 (**1**)	$C_{39}H_{65}NO_{29}$	–1.2	10.99	1012.3715	304.1395, 1012.3703	6
13	Ac-Aca II01	$C_{46}H_{76}N_2O_{31}$	–0.5	10.31	1153.4505	304.1392, 769.3233, 850.3168, 1153.4499	–
14	Ac-Aca II02	$C_{52}H_{86}N_2O_{36}$	–1.8	10.80	1315.5033	304.1390, 769.3231, 1012.3683, 1315.5009	–
15	Ac-Aca II03 (**7**)	$C_{58}H_{97}N_2O_{41}$	–1.2	11.11	1477.5561	304.1389, 769.3231, 1174.4232, 1477.5543	7
16	Hac-Aca I03	$C_{39}H_{65}NO_{30}$	–1.0	10.29	1028.3664	304.1391, 1028.3654	–
17	Hac-Aca II03	$C_{58}H_{96}N_2O_{42}$	2.4	11.80	1493.5510	304.1392, 769.3219, 1493.5546	–
18	Pr-Aca I01	$C_{28}H_{47}NO_{19}$	–3.4	12.39	702.2815	304.1385, 702.2791	–
19	Pr-Aca I02	$C_{34}H_{57}NO_{24}$	–2.0	12.59	864.3343	304.1379, 864.3326	–
20	Pr-Aca I03 (**2**)	$C_{40}H_{67}NO_{29}$	–1.0	13.10	1026.3872	304.1395, 1026.3862	6
21	Pr-Aca II01	$C_{47}H_{78}N_2O_{31}$	–0.6	11.63	1167.4661	304.1394, 769.3236, 864.3338, 1167.4654	–
22	Pr-Aca II02 (**11**)	$C_{53}H_{88}N_2O_{36}$	–0.7	11.86	1329.5190	304.1395, 769.3233, 1026.3870, 1329.5181	–
23	Pr-Aca II03 (**14**)	$C_{59}H_{98}N_2O_{41}$	–0.2	12.40	1491.5718	304.1377, 769.3239, 1188.4393, 1491.5715	–
24	Pr-Aca II04	$C_{65}H_{108}N_2O_{46}$	–4.0	12.15	1653.6246	304.1394, 769.3229, 1653.6180	–
25	Pr-Aca III03	$C_{78}H_{129}N_3O_{53}$	–2.8	11.95	1956.7564	304.1382, 769.3221, 1188.4364, 1956.7510	–
26	Hpr-Aca I02	$C_{34}H_{57}NO_{25}$	–1.4	12.07	880.3292	304.1389, 880.3280	–
27	Hpr-Aca I03	$C_{40}H_{67}NO_{30}$	–2.9	12.81	1042.3821	304.1381, 769.3215, 1042.3791	–
28	Hpr-Aca II03	$C_{59}H_{98}N_2O_{42}$	1.8	13.61	1507.5667	304.1387, 769.3211, 1204.4598, 1507.5694	–
29	isoBu-Aca I01	$C_{29}H_{49}NO_{19}$	–0.4	14.14	716.2972	304.1383, 716.2969	–

30	isoBu-Aca I02	$C_{35}H_{59}NO_{24}$	–1.1	14.56	878.3500	304.1390, 878.3490	–
31	isoBu-Aca I03 (**3**)	$C_{41}H_{69}NO_{29}$	0.6	15.43	1040.4028	304.1481, 1040.4034	7
32	Bu-Aca I03 (**18**)	$C_{41}H_{69}NO_{29}$	–0.1	15.53	1040.4028	304.1391, 1040.4027	8
33	Bu-Aca II01	$C_{48}H_{80}N_2O_{31}$	–1.1	13.55	1181.4818	304.1380, 769.3232, 878.3489, 1181.4805	8
34	isoBu-Aca II02 (**12**)	$C_{54}H_{90}N_2O_{36}$	–2.9	13.75	1343.5346	304.1386, 769.3255, 1040.4000, 1343.5307	–
35	isoBu-Aca II03 (**8**)	$C_{60}H_{100}N_2O_{41}$	0.2	14.40	1505.5874	304.1391, 769.3228, 1202.4547, 1505.5877	7
36	Bu-Aca II03 (**15**)	$C_{60}H_{100}N_2O_{41}$	2.2	14.48	1505.5874	304.1392, 769.3238, 1202.4570, 1505.5907	–
37	isoBu-Aca II04	$C_{66}H_{110}N_2O_{46}$	–3.8	15.76	1667.6403	304.1392, 769.3230, 1667.6339	–
38	Hbu-Aca I01	$C_{29}H_{49}NO_{20}$	–2.4	9.71	732.2921	304.1383, 732.2903	–
39	Hbu-Aca I02	$C_{35}H_{59}NO_{25}$	–0.3	10.15	894.3449	304.1397, 894.3446	–
40	Hbu-Aca I03 (**4**)	$C_{41}H_{70}NO_{30}$	–3.2	11.31	1056.3977	304.1386, 1056.3943	6
41	Hbu-Aca II01	$C_{48}H_{80}N_2O_{32}$	–2.4	10.35	1197.4767	304.1392, 769.3224, 894.3440, 1197.4738	–
42	Hbu-Aca II02	$C_{54}H_{90}N_2O_{37}$	–1.2	10.49	1359.5295	304.1382, 769.3230, 1056.3944, 1359.5279	–
43	Hbu-Aca II03 (**19**)	$C_{60}H_{100}N_2O_{42}$	–0.8	10.85	1521.5823	304.1383, 769.3228, 1218.4477, 1521.5810	6
44	isoVa-Aca I01	$C_{30}H_{51}NO_{19}$	–1.6	15.83	730.3128	304.1385, 730.3116	–
45	isoVa-Aca I02	$C_{36}H_{61}NO_{24}$	–0.4	16.78	892.3656	304.1408, 892.3660	–
46	Mbu-Aca I03 (**5**)	$C_{42}H_{71}NO_{29}$	–1.1	17.93`	1054.4184	304.1388, 1054.4172	7
47	isoVa-Aca I03 (**6**)	$C_{42}H_{71}NO_{29}$	1.5	18.04	1054.4184	304.1388, 1054.4200	9
48	isoVa-Aca II01	$C_{49}H_{82}N_2O_{30}$	–2.4	14.91	1195.4974	304.1300, 769.3226, 1195.4945	–
49	isoVa-Aca II02 (**13**)	$C_{55}H_{92}N_2O_{36}$	0.1	16.06	1357.5503	304.1390, 769.3219, 1054.4195, 1357.5505	–
50	Mbu-Aca II03 (**16**)	$C_{61}H_{102}N_2O_{41}$	0.6	16.65	1519.6031	304.1391, 769.3234, 1216.4712, 1519.6040	–
51	isoVa-Aca II03 (**20**)	$C_{61}H_{102}N_2O_{41}$	0.6	16.79	1519.6031	304.1389, 769.3226, 1216.4704, 1519.6041	10
52	Hva-Aca I01	$C_{30}H_{51}NO_{20}$	1.6	10.90	746.3077	304.1378, 746.3089	–
53	Hva-Aca I02	$C_{36}H_{61}NO_{25}$	–4.5	11.93	908.3605	304.1369, 908.3564	–
54	Hva-Aca I03	$C_{42}H_{71}NO_{30}$	–1.8	13.08	1070.4134	304.1372, 1070.4115	–
55	He-Aca I01	$C_{31}H_{53}NO_{19}$	–0.7	17.50	744.3285	304.1377, 744.3280	–
56	He-Aca I02	$C_{37}H_{63}NO_{24}$	–1.3	19.34	906.3813	304.1379, 906.3801	–
57	He-Aca I03	$C_{43}H_{73}NO_{29}$	–1.1	21.06	1068.4341	304.1378, 1068.4329	–
58	He-Aca II02	$C_{56}H_{94}N_2O_{36}$	–3.4	18.04	1371.5659	304.1392, 769.3182, 1371.5612	–
59	He-Aca II03	$C_{62}H_{104}N_2O_{41}$	–3.9	19.55	1533.6187	304.1379, 769.3215, 1230.4826, 1533.6127	–
60	Hhe-Aca I03	$C_{43}H_{73}NO_{30}$	–1.3	18.97	1084.4290	304.1396, 1084.4276	–
61	Hhe-Aca II01	$C_{50}H_{84}N_2O_{32}$	0.7	17.80	1225.5080	304.1394, 769.3238, 922.3765, 1225.5089	–

62	Hhe-Aca II02	$C_{56}H_{94}N_2O_{37}$	–0.2	17.93	1387.5608	304.1392, 769.3233, 1084.4272, 1387.5605	–
63	diHva-Aca I03	$C_{42}H_{71}NO_{31}$	–3.4	8.96	1086.4083	304.1390, 1086.4046	–

Aca, acarviostatin; Ac, acetyl; Hac, hydroxyacetyl; Pr, propionyl; Hpr, hydroxypropionyl; Bu, butyryl; isoBu, isobutyryl; Hbu, hydroxybutyryl; Mbu, 2-methyl-butyryl; isoVa, isovaleryl; Hva, hydroxyvaleryl; He, hexanoyl; Hhe, hydroxyhexanoyl; diHva, dihydroxyvaleryl. Acarviostatins, marked with "–" in Ref. column, are potential new compounds.

Table S11. Acarviostatins with glucose(s) at the reducing and nonreducing terminus (glu-Aca-glu) from *Streptomyces* sp. HO1518[a].

No.	Compounds	Formula	Δ (ppm)	t_R (min)	$[M + H]^+$	Characterized fragment ions
1	Aca I13	$C_{43}H_{73}NO_{33}$	–0.7	6.80	1132.4138	466.1933, 1132.4130
2	Ac-Aca I10	$C_{27}H_{45}NO_{19}$	1.6	10.34	688.2659	466.1900, 688.2670
3	Ac-Aca I12	$C_{39}H_{65}NO_{29}$	–1.7	10.19	1012.3715	466.1918, 1012.3698
4	Ac-Aca I13	$C_{45}H_{75}NO_{34}$	–1.1	10.50	1174.4243	466.1921, 1174.4230
5	Ac-Aca II11	$C_{52}H_{86}N_2O_{36}$	0.2	10.30	1315.5033	466.1922, 850.3170, 931.3730, 1315.5035
6	Pr-Aca I10	$C_{28}H_{47}NO_{19}$	1.1	12.09	702.2815	466.1920, 702.2823
7	Pr-Aca I13	$C_{46}H_{77}NO_{34}$	–1.3	12.20	1188.4400	466.1915, 1188.4384
8	Hpr-Aca II12	$C_{59}H_{98}N_2O_{42}$	–1.6	11.83	1507.5667	466.1921, 1042.3769, 931.3743, 1507.5642
9	Bu-Aca I13	$C_{47}H_{79}NO_{34}$	–1.7	13.44	1202.4556	466.1910, 1202.4536
10	Hbu-Aca I10	$C_{29}H_{49}NO_{20}$	–1.8	9.45	732.2921	466.1920, 732.2908
11	Hbu-Aca I12	$C_{41}H_{70}NO_{30}$	–0.5	10.13	1056.3977	466.1916, 1056.3972
12	Hbu-Aca I13	$C_{47}H_{79}NO_{35}$	–0.6	10.02	1218.4505	466.1920, 1218.4498
13	Hbu-Aca II10	$C_{48}H_{80}N_2O_{32}$	–2.0	9.97	1197.4767	466.1919, 931.3626, 1197.4743
14	isoVa-Aca I13	$C_{48}H_{81}NO_{34}$	–2.0	15.10	1216.4713	466.1908, 1216.4688
15	Hva-Aca I11	$C_{36}H_{61}NO_{25}$	–2.3	11.34	908.3605	466.1902, 908.3584

Aca, acarviostatin; Ac, acetyl; Pr, propionyl; Hpr, hydroxypropionyl; Hbu, hydroxybutyryl; Hva, hydroxyvaleryl. [a] all isolates are potential new compounds.

Table S12. Acarviostatins with an incomplete pseudo-trisaccharide at the nonreducing terminus (incAca-glu) from *Streptomyces* sp. HO1518[a].

No.	Compounds	Formula	Δ (ppm)	t_R (min)	$[M+H]^+$	Characterized fragment ions
1	Aca I(–1)2	$C_{30}H_{53}NO_{24}$	0.1	5.90	812.3030	146.0813, 812.3031
2	Aca II(–1)2	$C_{43}H_{74}N_2O_{31}$	–1.5	7.56	1115.4348	146.0811, 611.2658, 1115.4331
3	Aca II(–1)3	$C_{49}H_{84}N_2O_{36}$	–2.0	8.94	1277.4877	146.0804, 611.2647, 1277.4851
4	Ac-Aca I(–1)3	$C_{32}H_{55}NO_{25}$	–2.2	10.44	854.3136	146.0810, 854.3117
5	Ac-Aca II(–1)2	$C_{45}H_{76}N_2O_{32}$	2.5	10.23	1157.4454	146.0810, 611.2639, 1157.4483
6	Ac-Aca II(–1)3	$C_{51}H_{86}N_2O_{37}$	–2.2	10.60	1319.4982	146.0811, 611.2655, 1174.4167, 1319.4953
7	Hac-Aca I(–1)3	$C_{32}H_{55}NO_{26}$	–2.0	9.74	870.3085	146.0809, 870.3067
8	Pr-Aca I(–1)3	$C_{34}H_{57}NO_{24}$	–1.3	12.42	868.3292	146.0811, 868.3281
9	Pr-Aca II(–1)2	$C_{46}H_{78}N_2O_{32}$	0.8	11.89	1171.4610	146.0809, 611.2623, 1026.3845, 1171.4600
10	Pr-Aca II(–1)3	$C_{52}H_{88}N_2O_{37}$	–4.4	12.37	1333.5139	146.0810, 611.2648, 1188.4208, 1333.5080
11	Hpr-Aca II(–1)3	$C_{52}H_{88}N_2O_{38}$	–1.6	12.50	1349.5088	146.0810, 611.2655, 1204.4326, 1349.5066
12	Bu-Aca II(–1)2	$C_{47}H_{80}N_2O_{32}$	3.8	13.72	1185.4767	146.0800, 611.2651, 1185.4812
13	Bu-Aca II(–1)3	$C_{53}H_{90}N_2O_{37}$	–3.8	14.10	1347.5295	146.0799, 611.2649, 1202.4555, 1347.5245
14	Hbu-Aca I(–1)3	$C_{34}H_{59}NO_{26}$	–2.4	9.64	898.3398	146.0803, 898.3376
15	Hbu-Aca II(–1)2	$C_{47}H_{80}N_2O_{33}$	–3.4	10.34	1201.4716	146.0812, 611.2656, 1056.3941, 1201.4675
16	isoVa-Aca I(–1)3	$C_{35}H_{61}NO_{25}$	–2.4	15.90	896.3605	146.0805, 896.3583
17	isoVa-Aca II(–1)2	$C_{48}H_{82}N_2O_{32}$	–1.8	15.72	1199.4923	146.0810, 611.2651, 1054.4174, 1199.4901
18	isoVa-Aca II(–1)3	$C_{54}H_{92}N_2O_{37}$	1.0	16.13	1361.5452	146.0810, 611.2637, 1216.4712, 1361.5465
19	Hva-Aca II(–1)2	$C_{48}H_{82}N_2O_{33}$	–2.4	11.20	1215.4873	146.0797, 611.2645, 1215.4844
20	Hva-Aca II(–1)3	$C_{54}H_{92}N_2O_{38}$	–3.0	11.41	1377.5401	146.0797, 611.2644, 1218.4834, 1377.5360

Aca, acarviostatin; Ac, acetyl; Hac, hydroxyacetyl; Pr, propionyl; Hpr, hydroxypropionyl; Bu, butyryl; Hbu, hydroxybutyryl; isoVa, isovaleryl; Hva, hydroxyvaleryl.

[a] all isolates are potential new compounds.

References

1. Schmidt, D.D.; Frommer, W.; Junge, B.; Müller, L.; Wingender, W.; Truscheit, E.; Schäfer, D. α-Glucosidase inhibitors. New complex oligosaccharides of microbial origin. *Naturwissenschaften* **1977**, *64*, 535–536.

2. Fukuhara, K.; Murai, H.; Murao, S. Isolation and structure-activity relationship of some amylostatins (F-lb Fraction) produced by *Streptomyces diastaticus* subsp *amylostaticus* No. 9410. *Agric. Biol. Chem.* **1982**, *46*, 1941–1945.
3. Geng, P.; Qiu, F.; Zhu, Y.Y.; Bai, G. Four acarviosin-containing oligosaccharides identified from *Streptomyces coelicoflavus* ZG0656 are potent inhibitors of *α*-amylase. *Carbohydr. Res.* **2008**, *343*, 882–892.
4. Qian, M.X.; Nahoum, V.; Bonicel, J.; Bischoff, H.; Henrissat, B.; Payan, F. Enzyme-catalyzed condensation reaction in a mammalian *α*-amylase. High resolution structural analysis of an enzymeinhibitor complex. *Biochemistry* **2001**, *40*, 7700–7709.
5. Weiss, S.C.; Skerra, A.; Schiefner, A. Structural basis for the interconversion of maltodextrins by MalQ, the amylomaltase of *Escherichia coli*. *J. Biol. Chem.* **2015**, *290*, 21352–21364.
6. Liu, H.L.; E, H.C.; Xie, D.A.; Cheng, W.B.; Tao, W.Q.; Wang, Y. Acylated aminooligosaccharides with inhibitory effects against α-amylase from *Streptomyces* sp. HO1518. *Mar. Drugs* **2018**, *16*, 403.
7. Xu, J.L.; Liu, H.L.; Liu, Z.F.; Ren, Y.H.; Wang, Y. Acylated aminooligosaccharides from the yellow sea *Streptomyces* sp. HO1518 as both α-glucosidase and lipase inhibitors. *Mar. Drugs* **2020**, *18*, 576.
8. Si, D.Y.; Zhong, D.F.; Xu, Q.M. Two butylated aminooligosaccharides isolated from the culture filtrate of *Streptomyces luteogriseus*. *Carbohydr. Res.* **2001**, *335*, 127–132.
9. Zhong, D.F.; Si, D.Y.; He, W.Y.; Zhao, L.M.; Xu, Q.M. Structural revision of isovalertatins M03, M13, and M23 isolated from the culture of *Streptomyces luteogriseus*. *Carbohydr. Res.* **2001**, *331*, 69–75.
10. Si, D.Y.; Zhong, D.F.; He, W.Y.; Zhao, L.M. Structural revision of isovalertatins D03 and D23 isolated from the culture filtrate of *Streptomyces luteogriseus*. *Chin. Chem. Lett.* **2001**, *12*, 327–330.

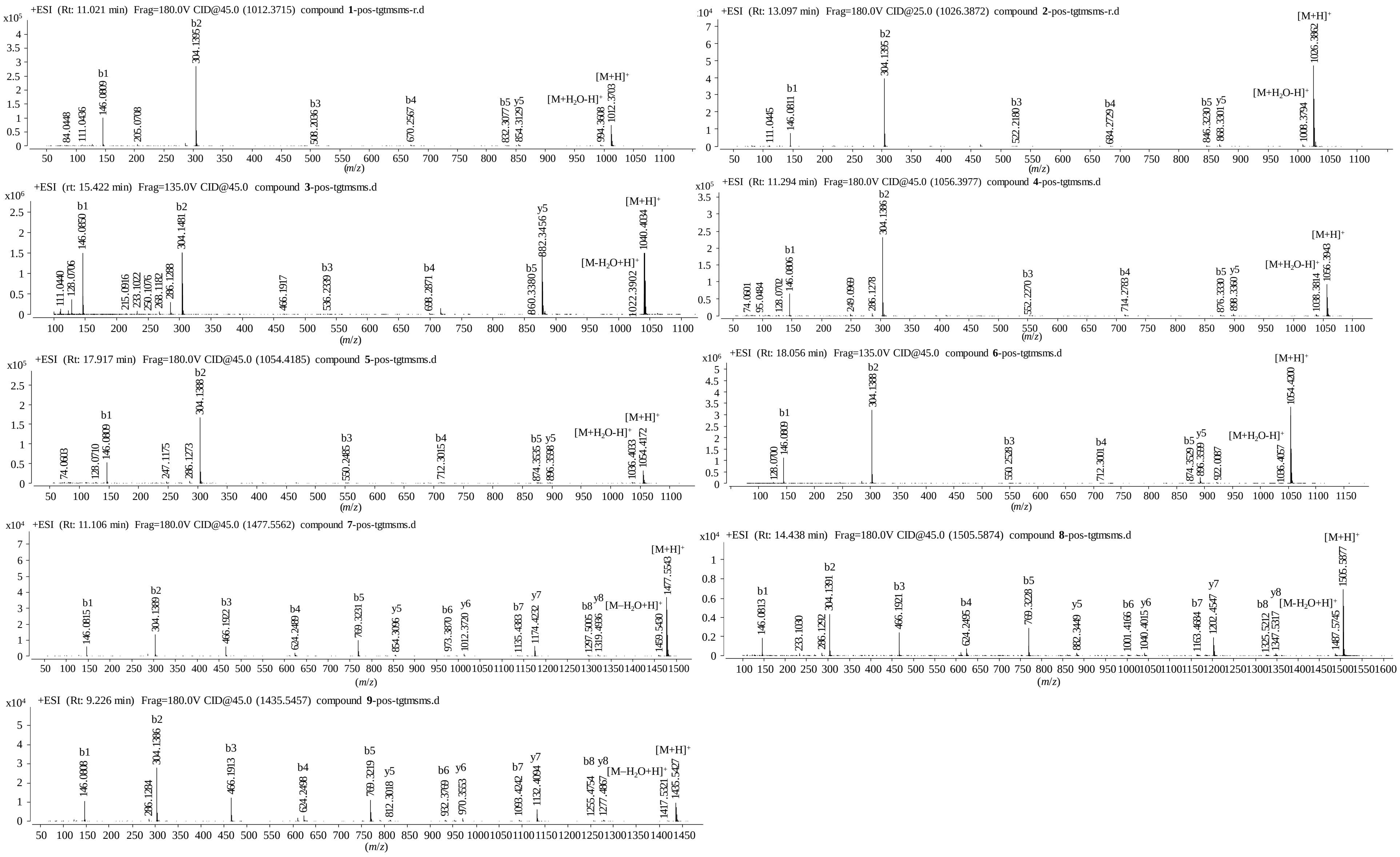

Figure S1. HRESI-MS/MS spectra of compounds **1-9**.

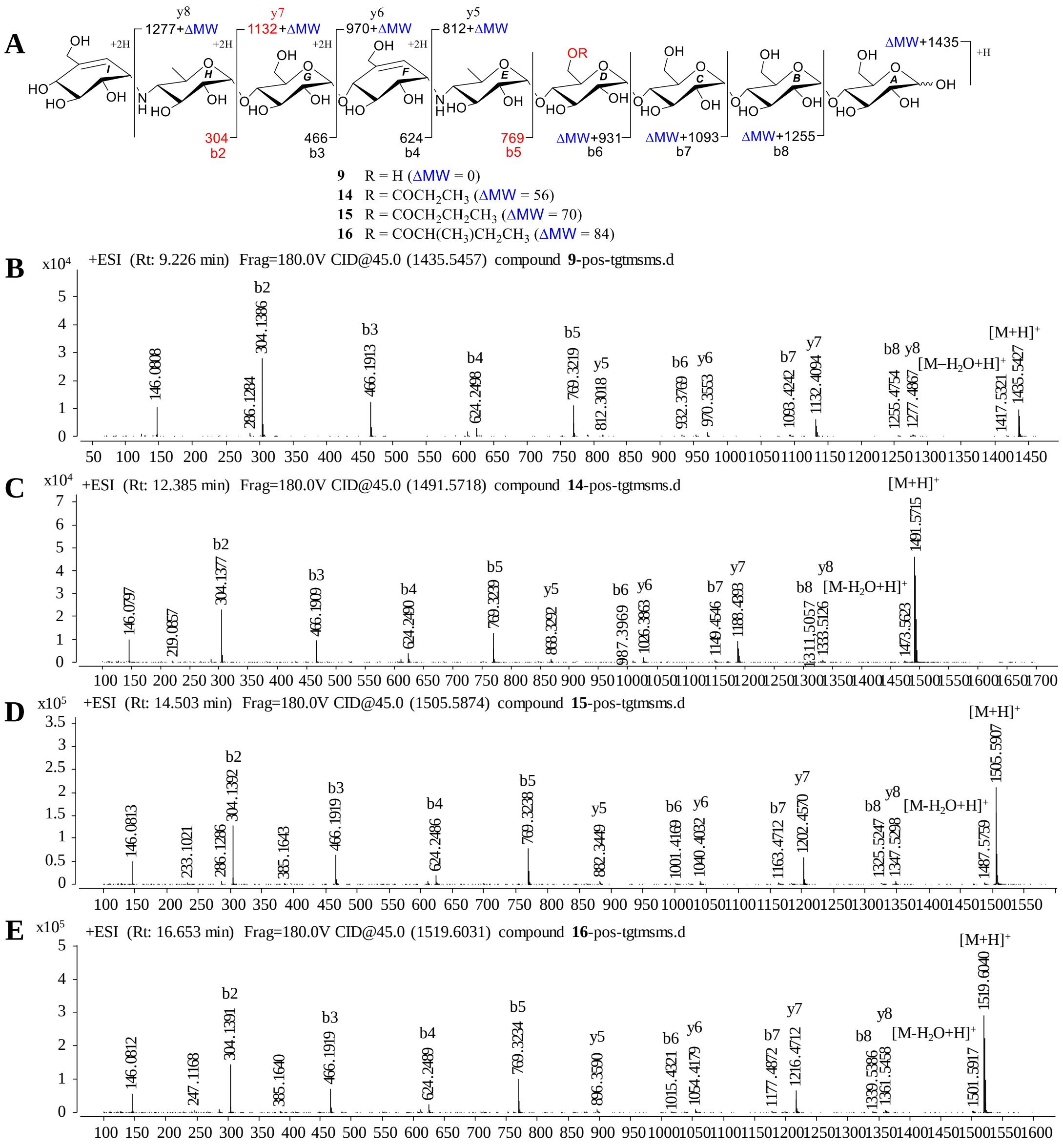

Figure S2. Positive HRESIMS/MS fragmentation and spectra of **9** and **14-16**. (A) Positive-ion HRESIMS/MS fragmentation patterns of **9** and **14-16**; (B–E) HRESIMS/MS spectra of **9** and **14-16**.

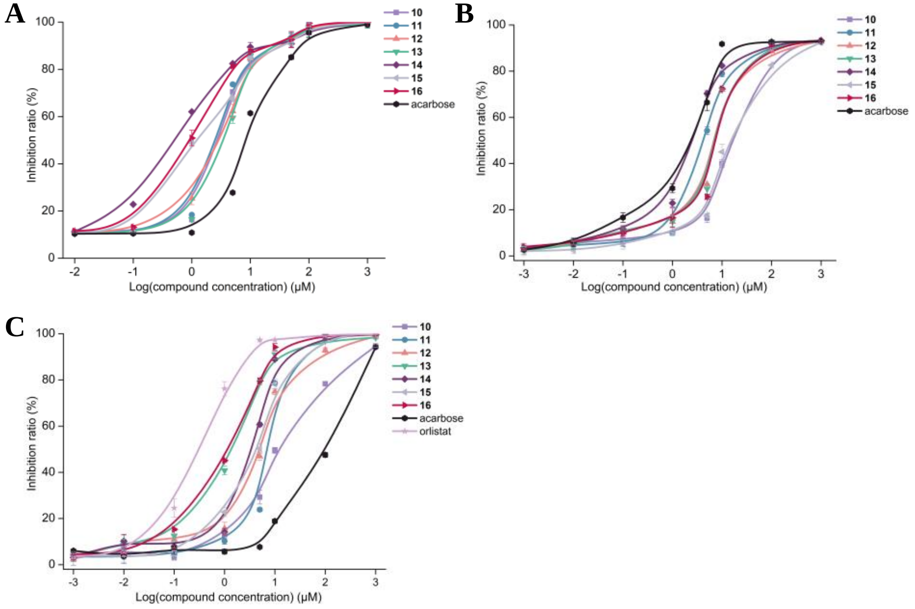

Figure S3. The inhibitory activities of **10-16** against three digestive enzymes. (A) The inhibitory activities of **10-16** against PPA. (B) The inhibitory activities of **10-16** against sucrase. (C) The inhibitory activities of **10-16** against PL.

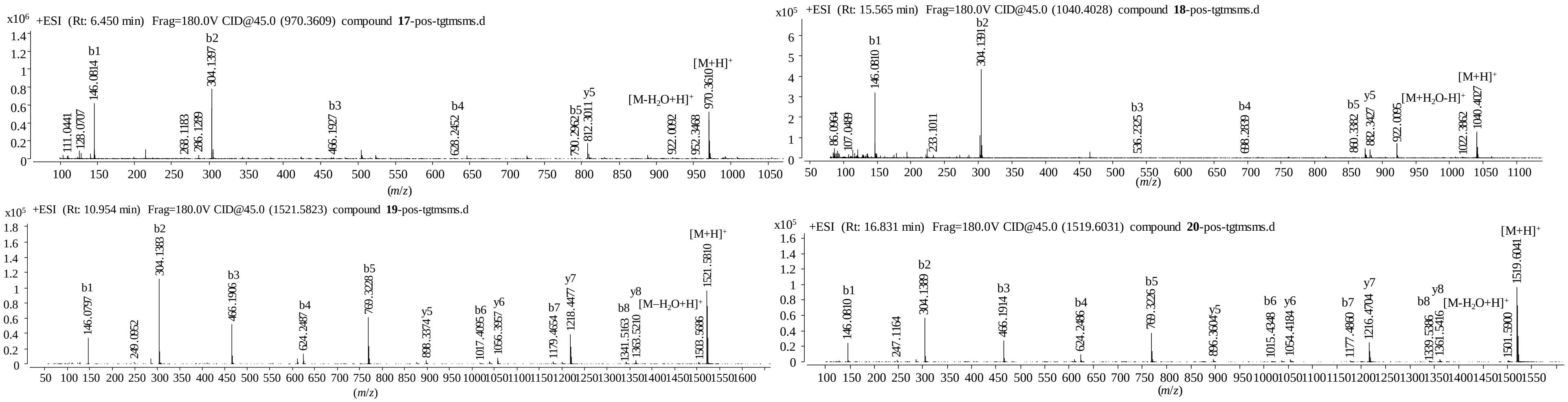

Figure S4. HRESI-MS/MS spectra of compounds **17-20**.

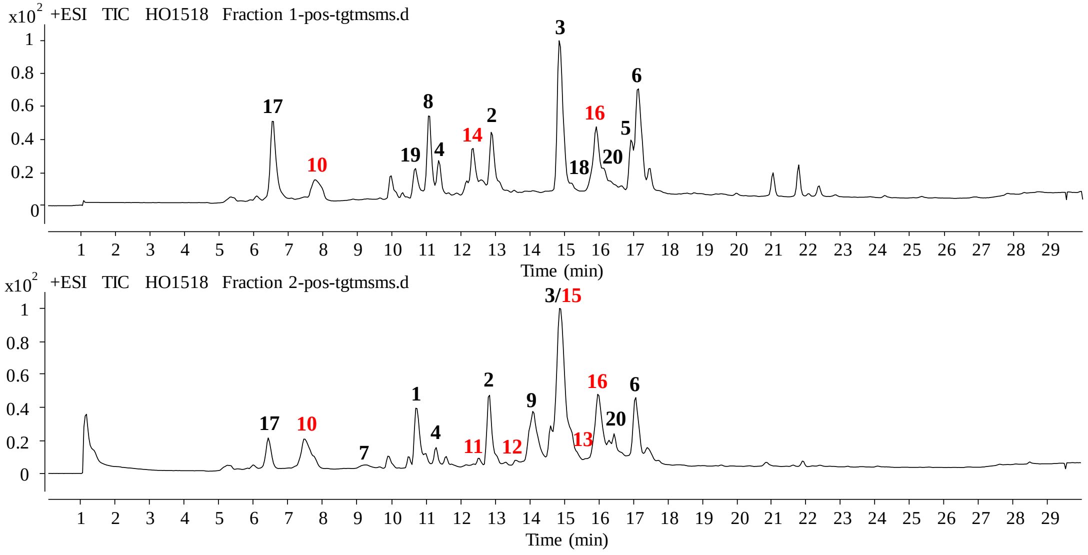

Figure S5. The total ion chromatograms of fraction 1 and 2 derived from *Streptomyces* sp. HO1518.

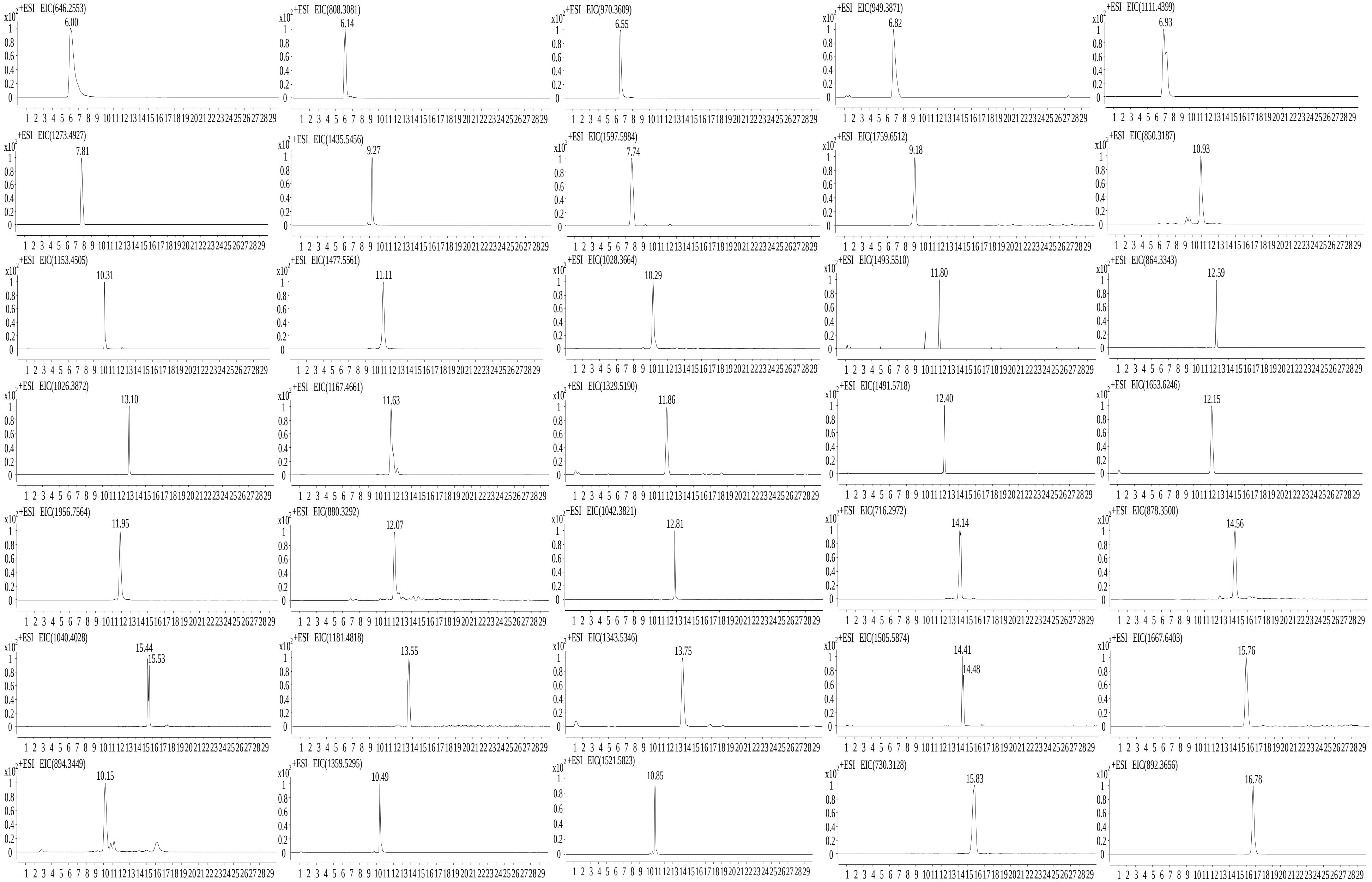
+ESI EIC(646.2553)
6.00
+ESI EIC(808.3081)
6.14
+ESI EIC(970.3609)
6.55
+ESI EIC(949.3871)
6.82
+ESI EIC(1111.4399)
6.93
+ESI EIC(1273.4927)
7.81
+ESI EIC(1435.5456)
9.27
+ESI EIC(1597.5984)
7.74
+ESI EIC(1759.6512)
9.18
+ESI EIC(850.3187)
10.93
+ESI EIC(1153.4505)
10.31
+ESI EIC(1477.5561)
11.11
+ESI EIC(1028.3664)
10.29
+ESI EIC(1493.5510)
11.80
+ESI EIC(864.3343)
12.59
+ESI EIC(1026.3872)
13.10
+ESI EIC(1167.4661)
11.63
+ESI EIC(1329.5190)
11.86
+ESI EIC(1491.5718)
12.40
+ESI EIC(1653.6246)
12.15
+ESI EIC(1956.7564)
11.95
+ESI EIC(880.3292)
12.07
+ESI EIC(1042.3821)
12.81
+ESI EIC(716.2972)
14.14
+ESI EIC(878.3500)
14.56
+ESI EIC(1040.4028)
15.44
15.53
+ESI EIC(1181.4818)
13.55
+ESI EIC(1343.5346)
13.75
+ESI EIC(1505.5874)
14.41
14.48
+ESI EIC(1667.6403)
15.76
+ESI EIC(894.3449)
10.15
+ESI EIC(1359.5295)
10.49
+ESI EIC(1521.5823)
10.85
+ESI EIC(730.3128)
15.83
+ESI EIC(892.3656)
16.78

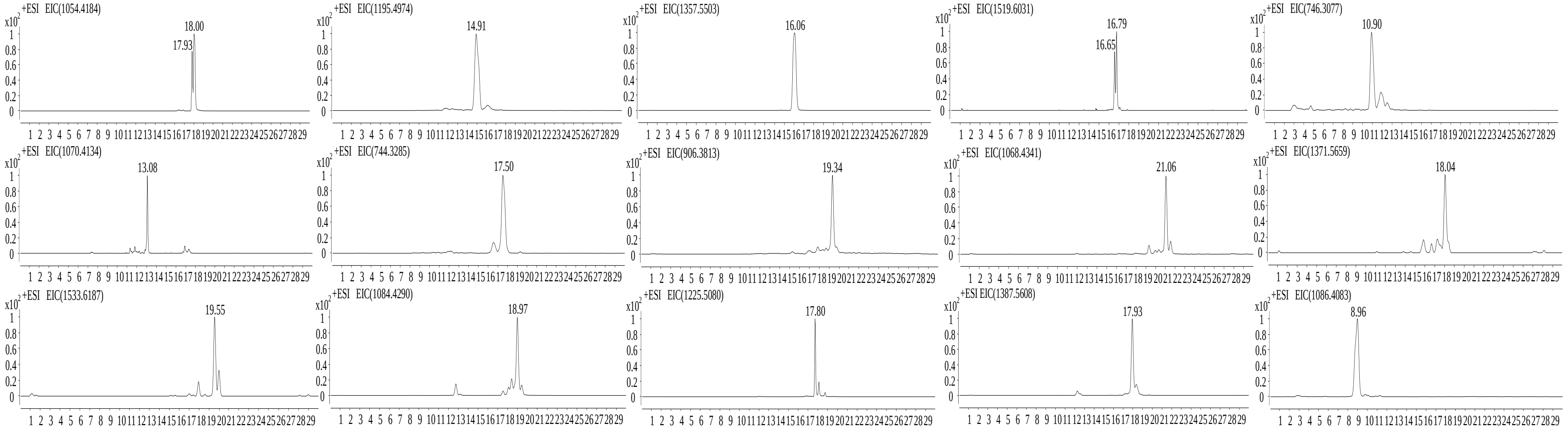

Figure S6. The EIC of acarviostatins with glucose at the reducing terminus from *Streptomyces* sp. HO1518.

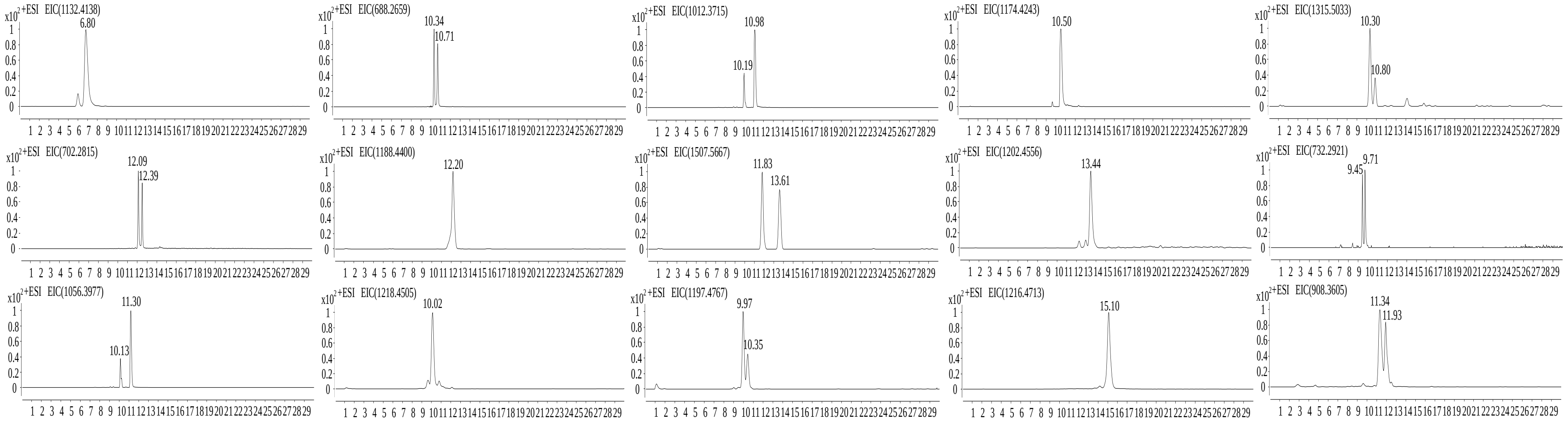

Figure S7. The EIC of acarviostatins with glucose at the reducing and nonreducing terminus from *Streptomyces* sp. HO1518.

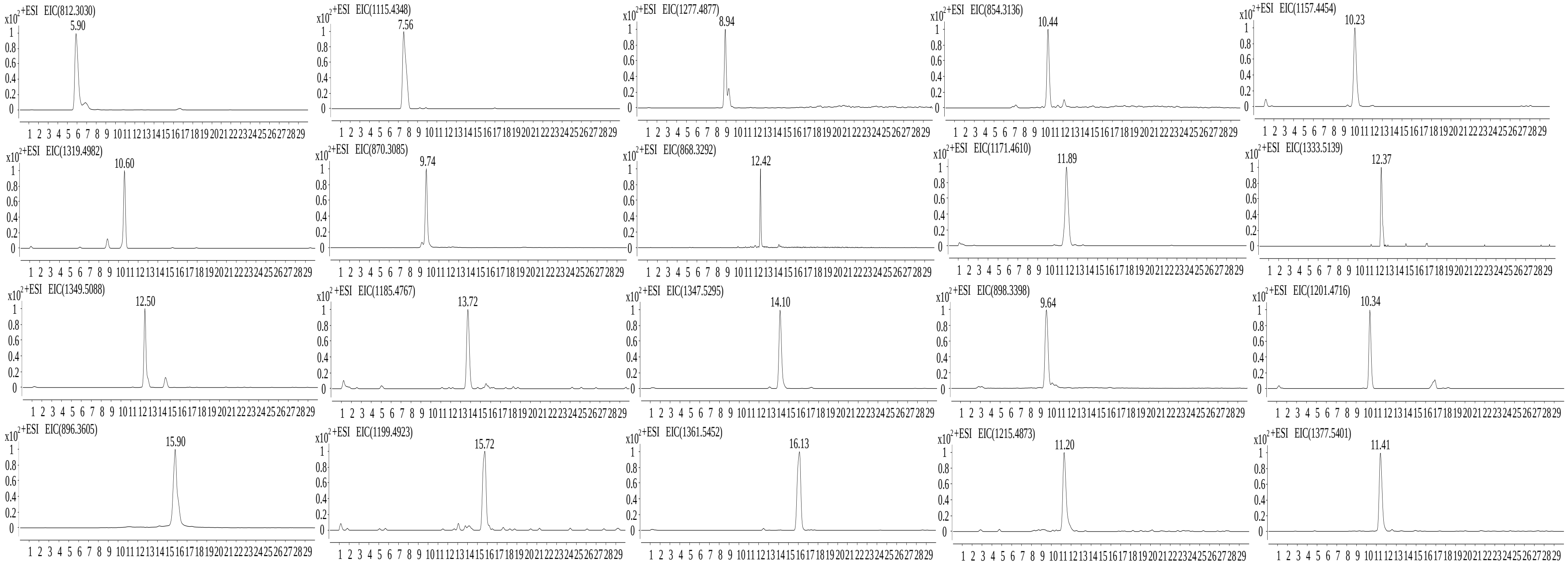

Figure S8. The EIC of acarviostatins with an incomplete *pseudo*-tetrasaccharide at the nonreducing terminus from *Streptomyces* sp. HO1518.

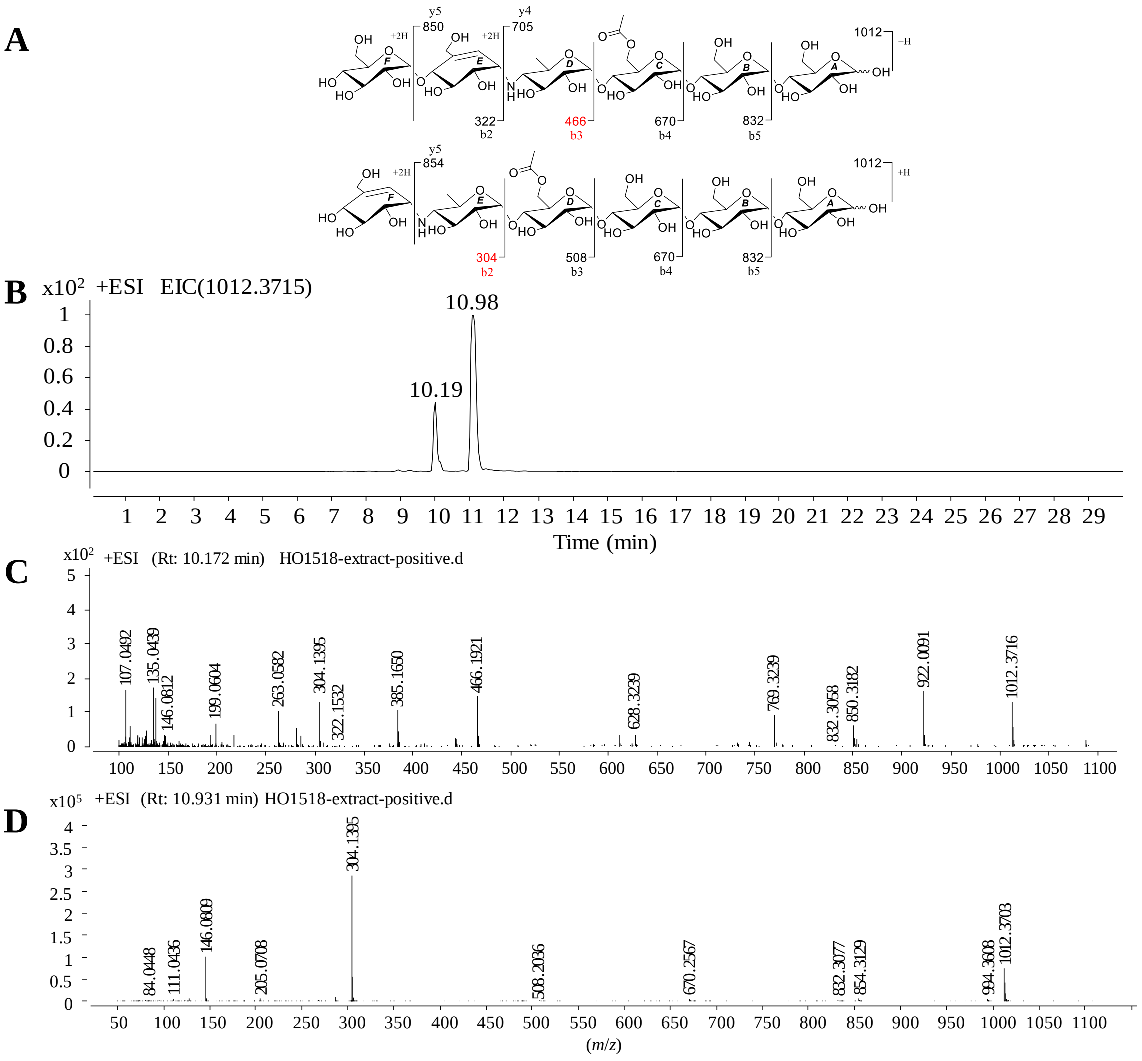

Figure S9. Positive HRESIMS/MS fragmentation and spectra of Ac-Aca I12 and Ac-Aca I03. (A) Positive-ion HRESIMS/MS fragmentation patterns of Ac-Aca I12 and Ac-Aca I03; (B) The extracted ion chromatogram of aminooligosaccharides at *m/z* 1012; (C) HRESIMS/MS spectrum of Ac-Aca I12; (D) HRESIMS/MS spectrum of Ac-Aca I03.

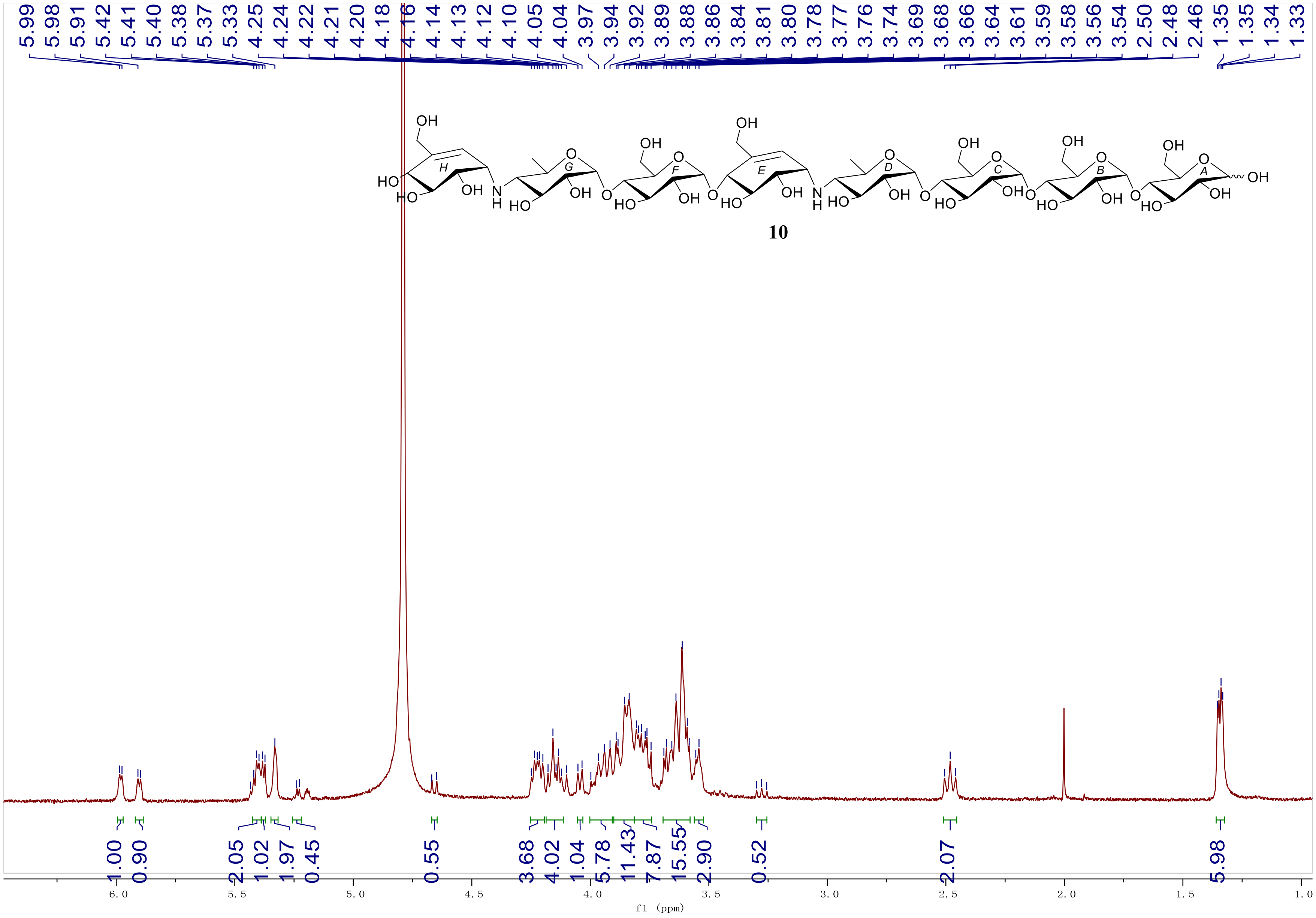

Figure S10. ^{1}H NMR spectrum of compound **10** (500 MHz, D_2O).

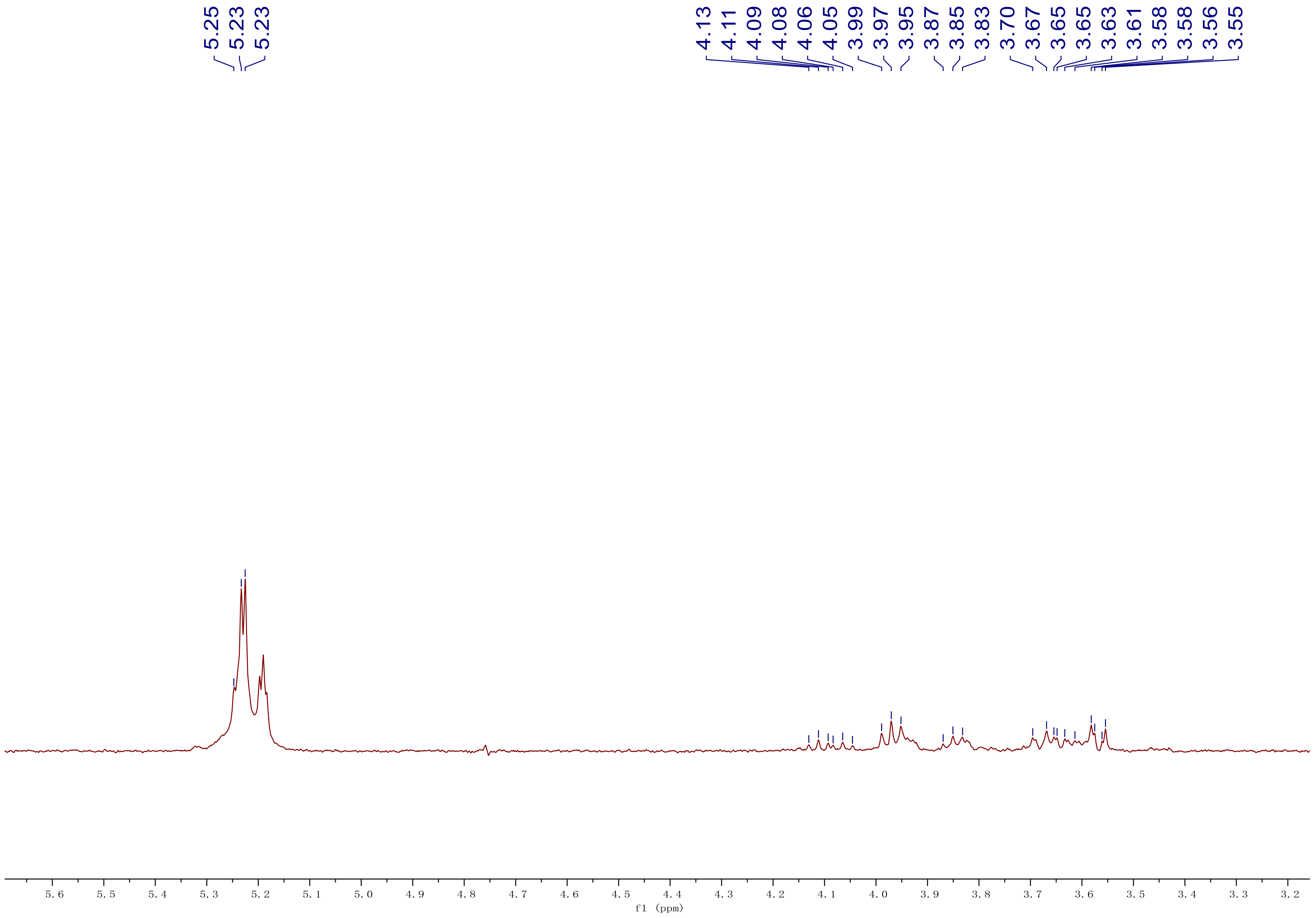

Figure S11. 1D-selective TOCSY spectrum of compound **10** (500 MHz, D_2O, excitation at δ 5.23, H-**A**1α).

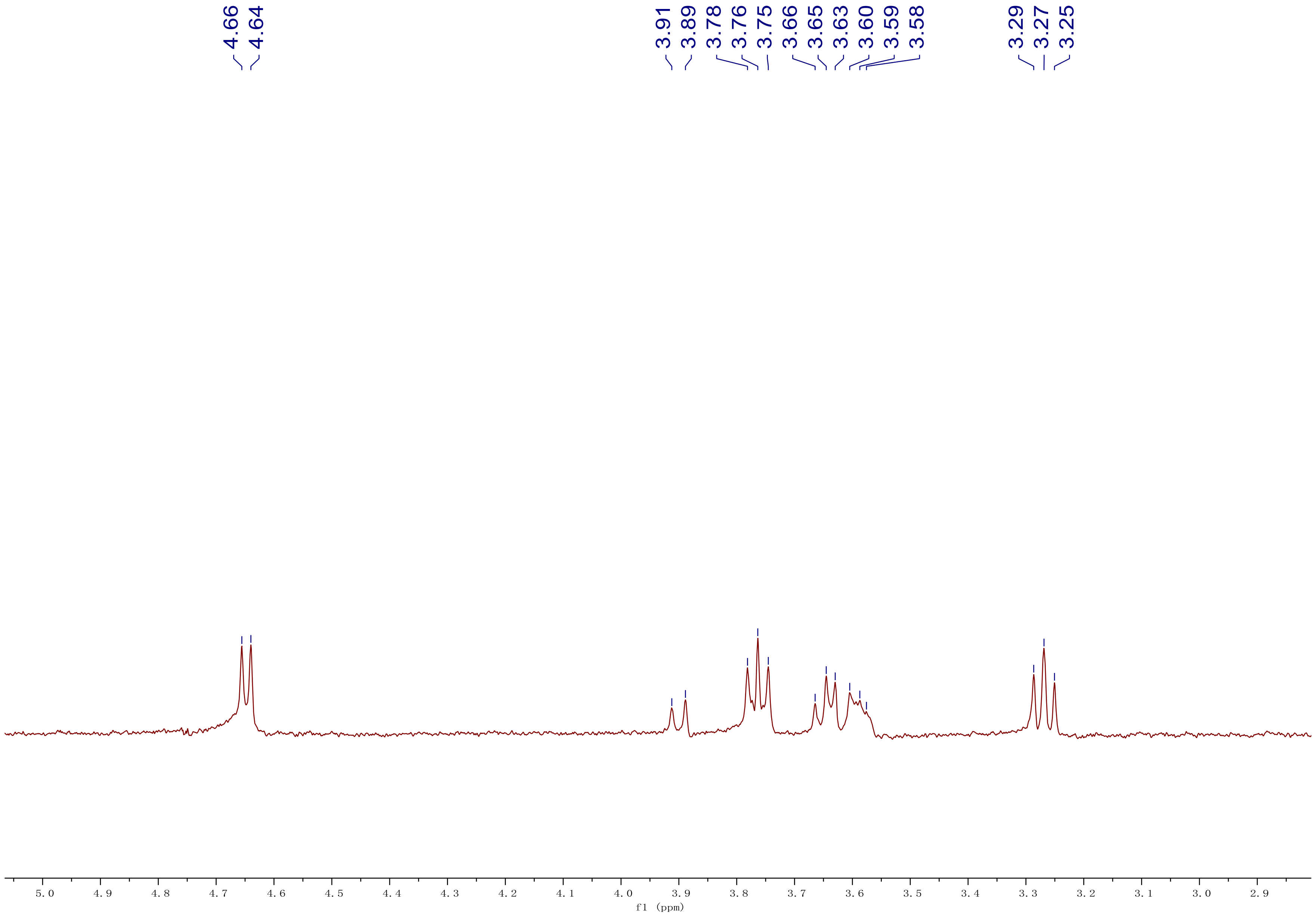

Figure S12. 1D-selective TOCSY spectrum of compound **10** (500 MHz, D_2O, excitation at δ 4.65, H-**A**1β).

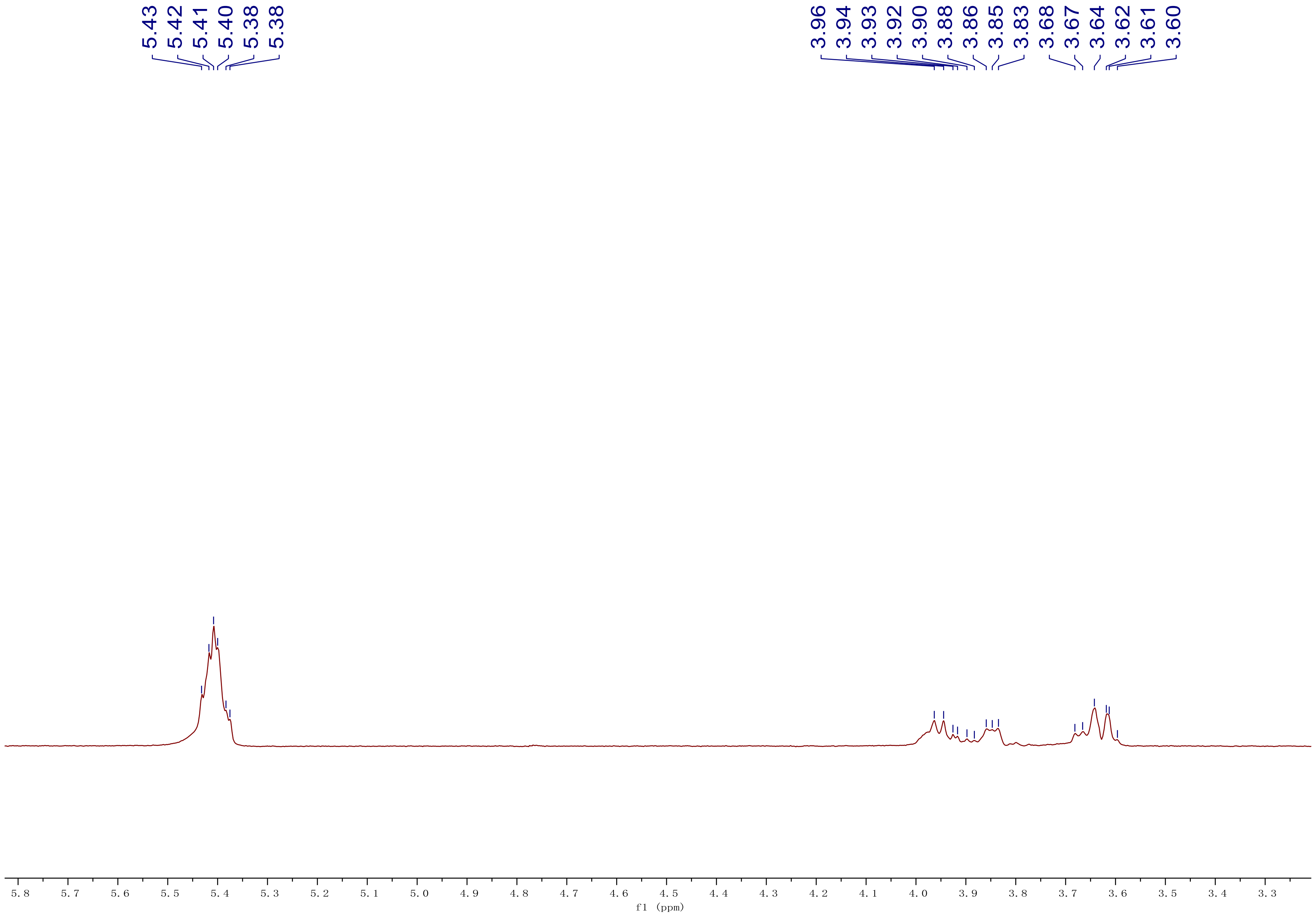

Figure S13. 1D-selective TOCSY spectrum of compound **10** (500 MHz, D_2O, excitation at δ 5.41, H-**B**1, and H-**C**1).

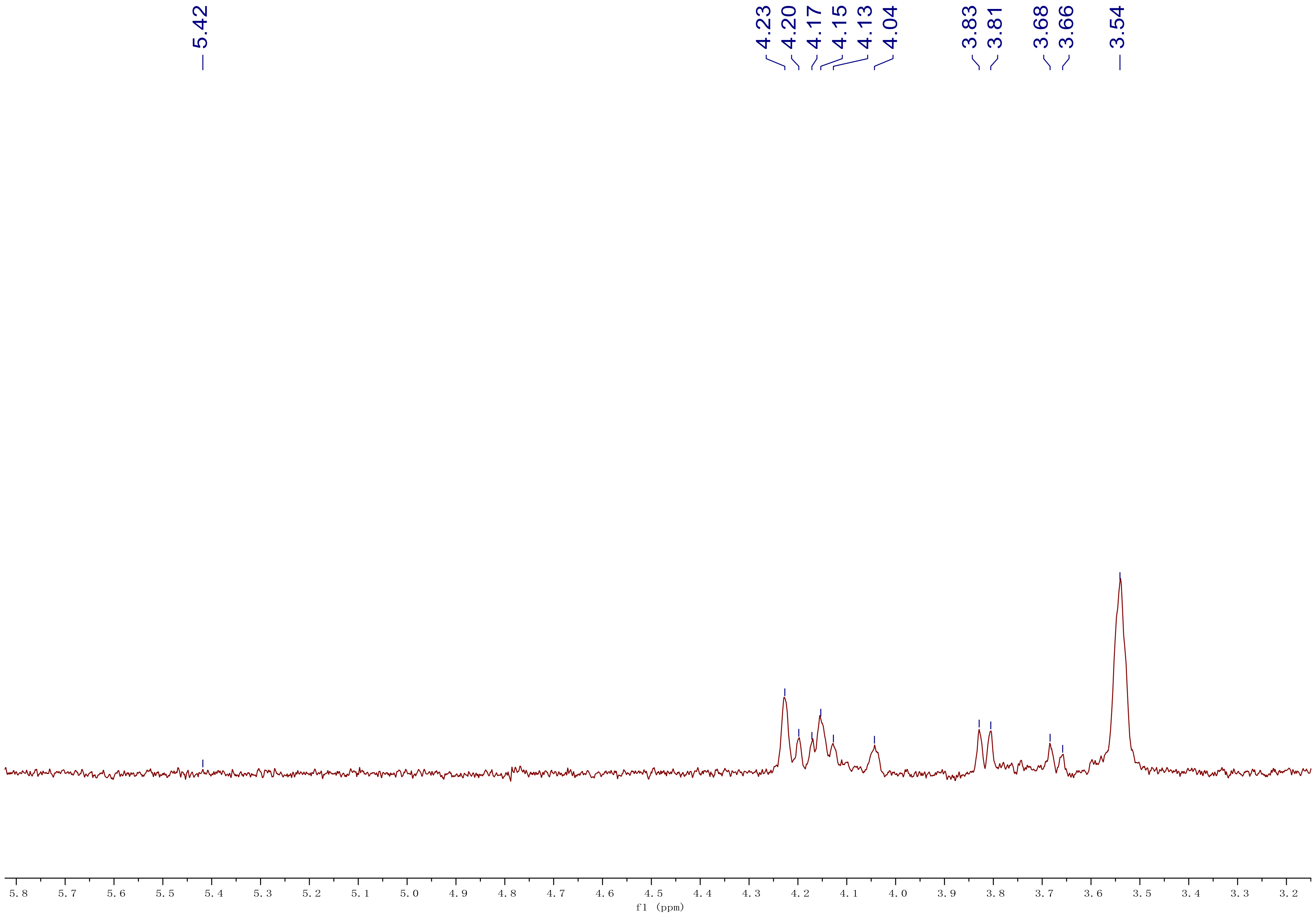

Figure S14. 1D-selective TOCSY spectrum of compound **10** (500 MHz, D_2O, excitation at δ 4.22, H-**C**6).

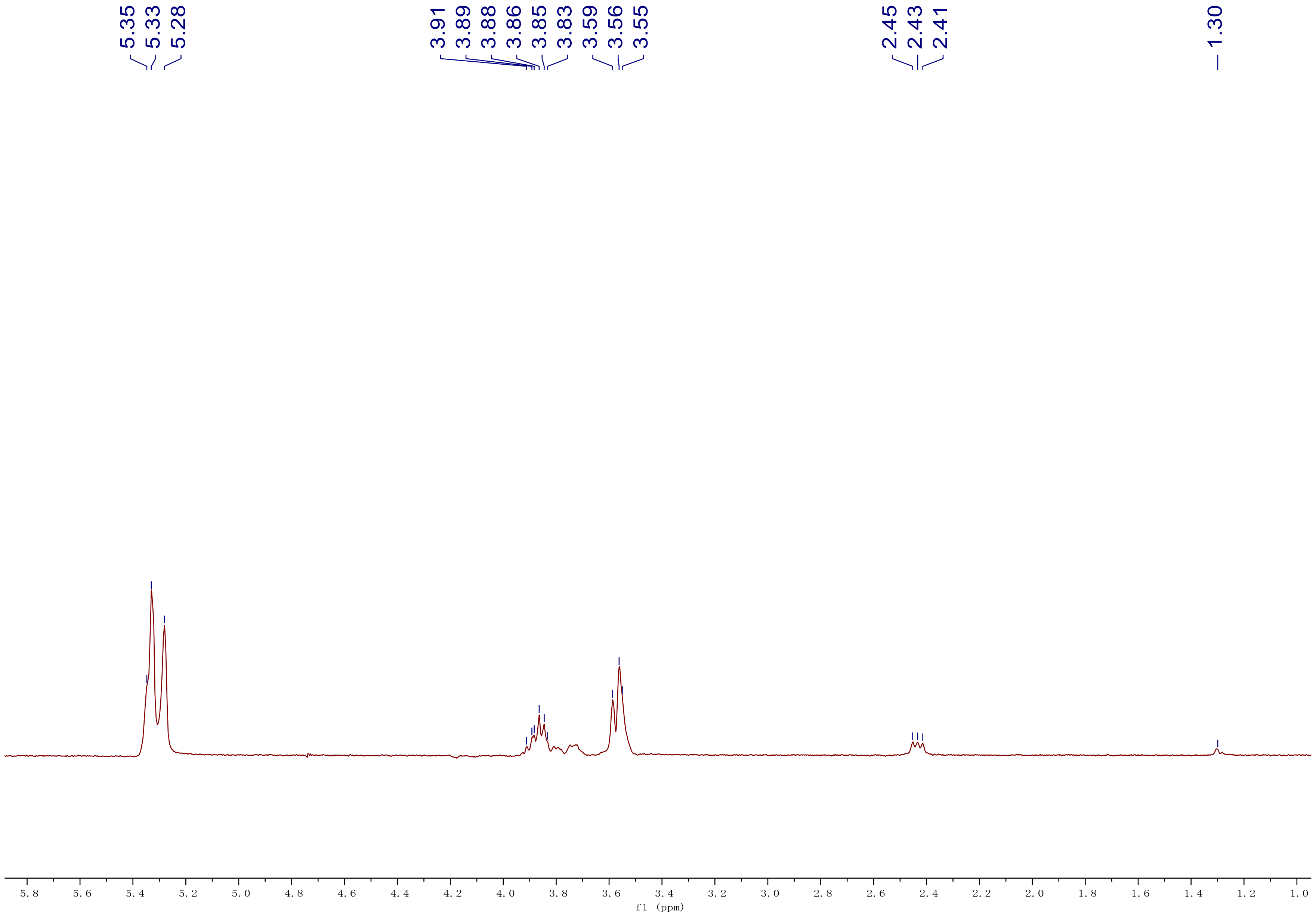

Figure S15. 1D-selective TOCSY spectrum of compound **10** (500 MHz, D_2O, excitation at δ 5.33, H-**D**1 and H-**G**1).

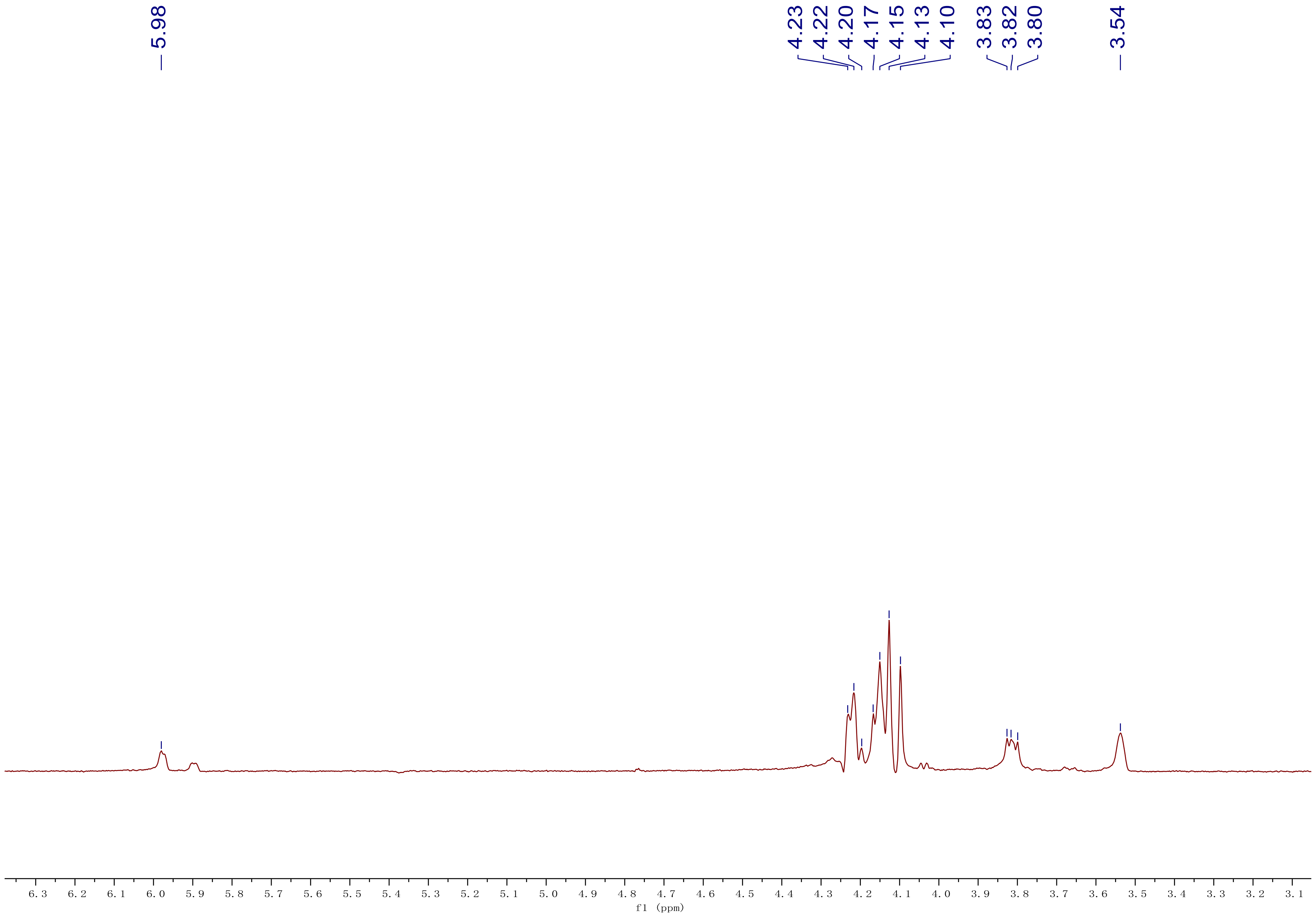

Figure S16. 1D-selective TOCSY spectrum of compound **10** (500 MHz, D_2O, excitation at δ 5.98, H-**E**7).

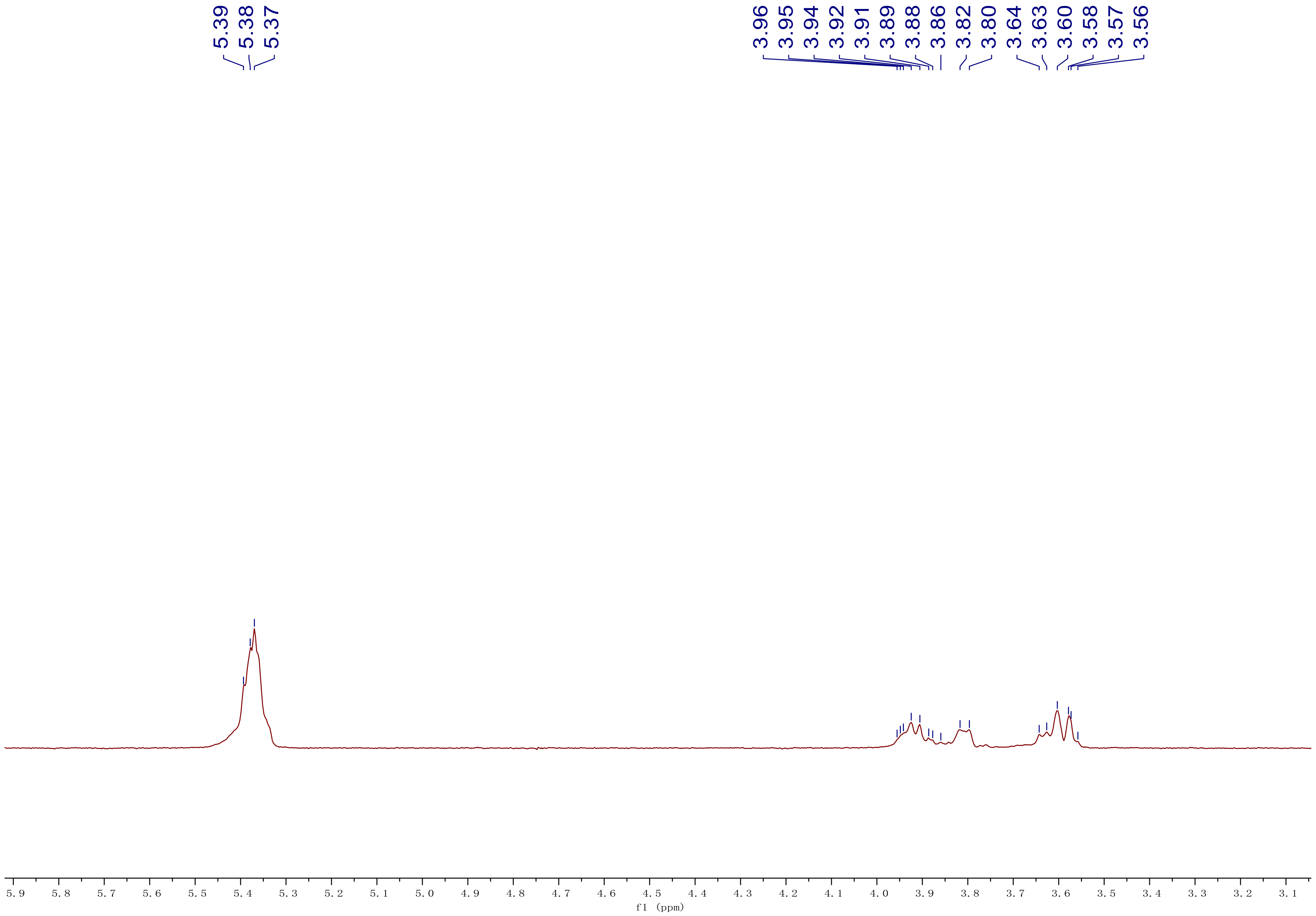

Figure S17. 1D-selective TOCSY spectrum of compound **10** (500 MHz, D_2O, excitation at δ 5.38, H-**F**1).

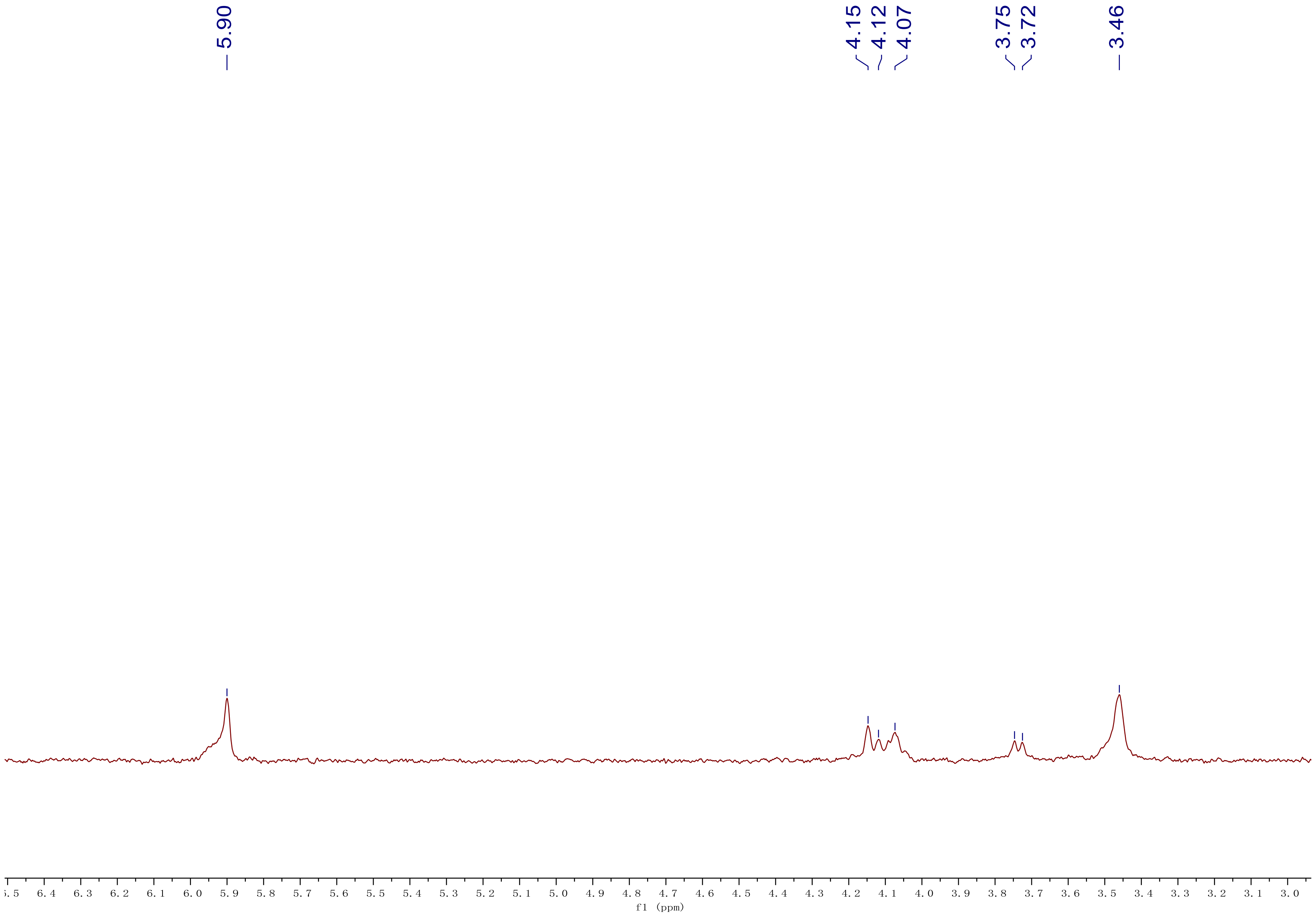

Figure S18. 1D-selective TOCSY spectrum of compound **10** (500 MHz, D_2O, excitation at δ 5.90, H-**H**7).

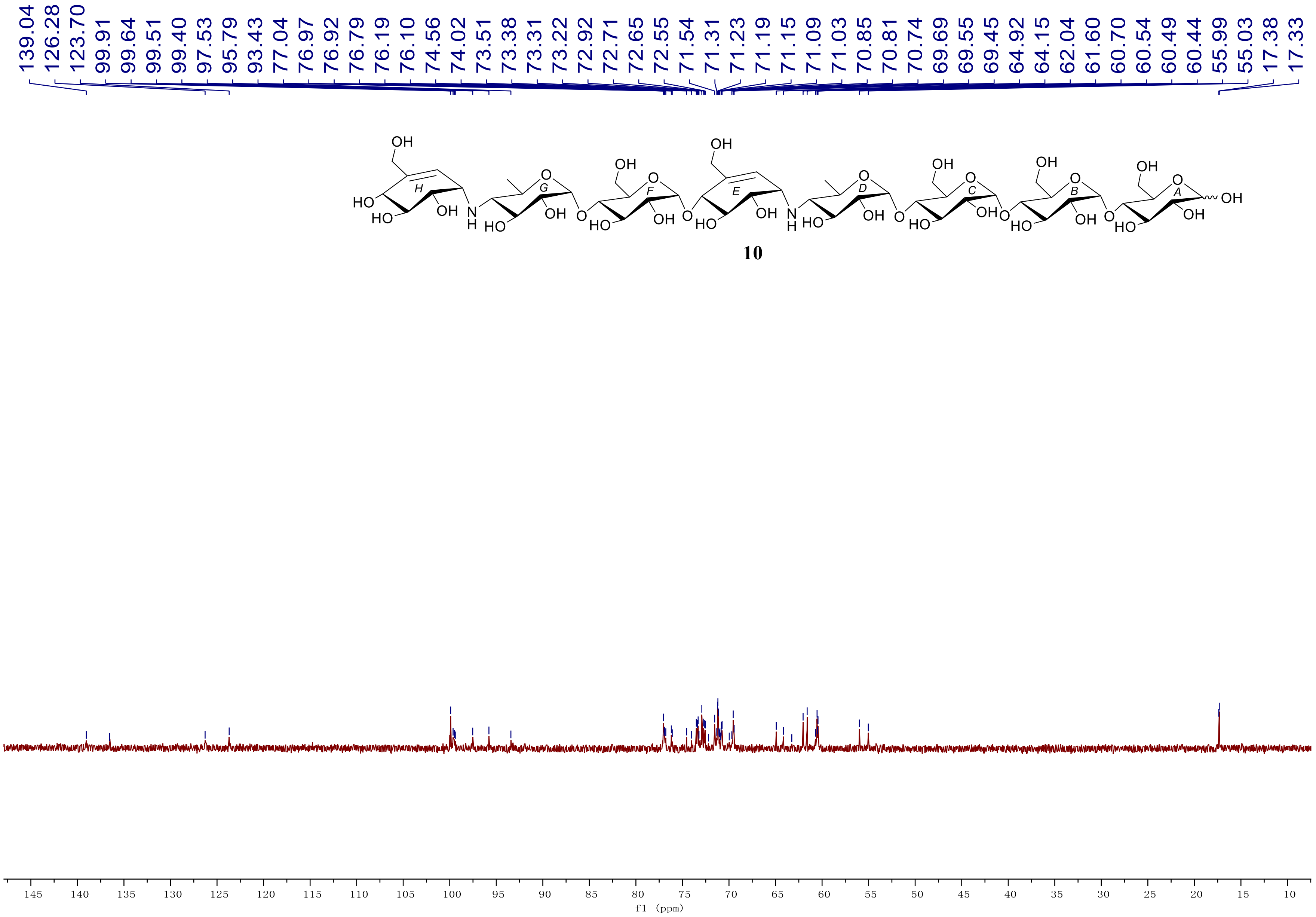

Figure S19. ^{13}C NMR spectrum of compound **10** (125 MHz, D_2O).

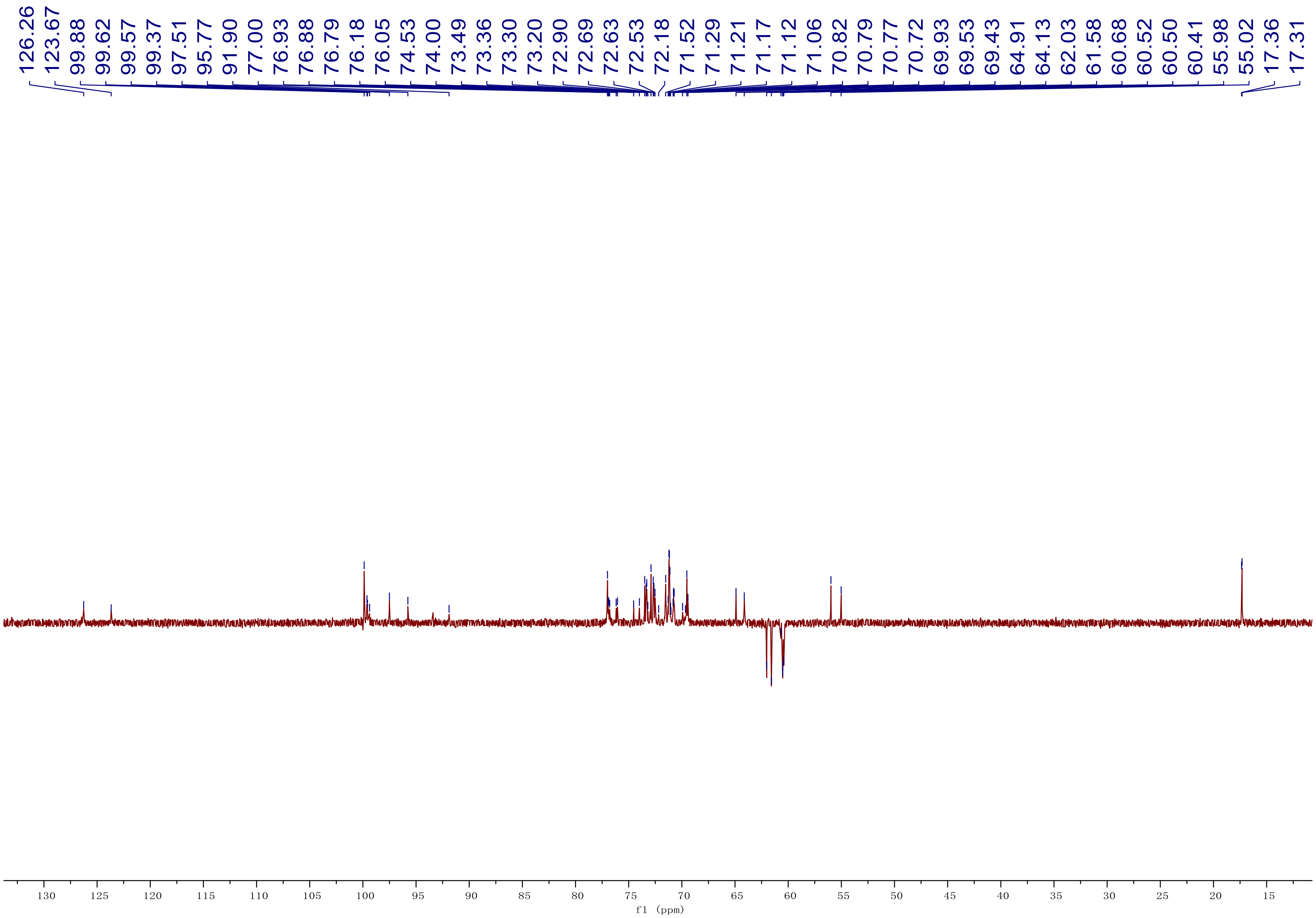

Figure S20. DEPT-135 spectrum of compound **10** (125 MHz, D_2O).

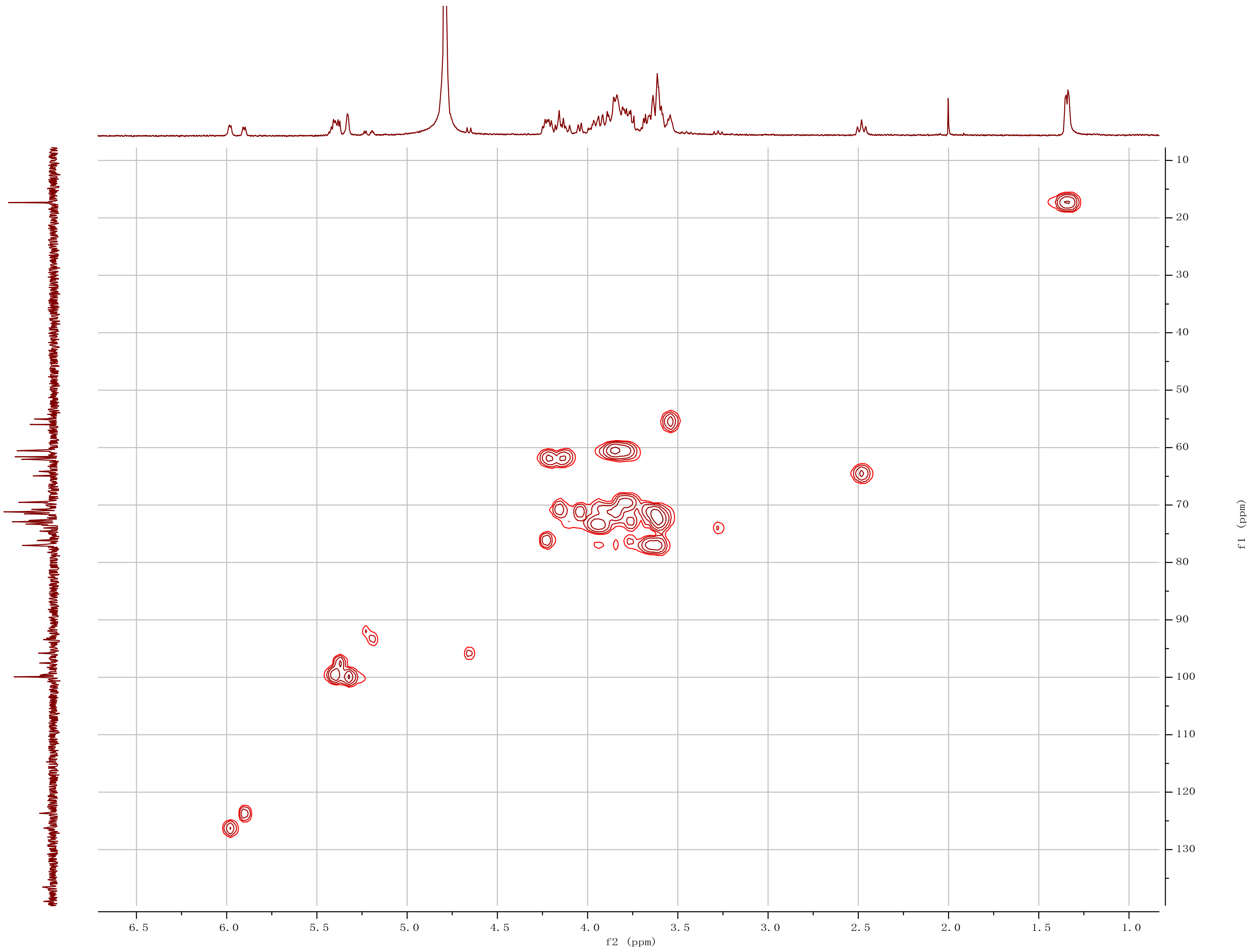

Figure S21. HSQC spectrum of compound **10** (500 MHz, D_2O).

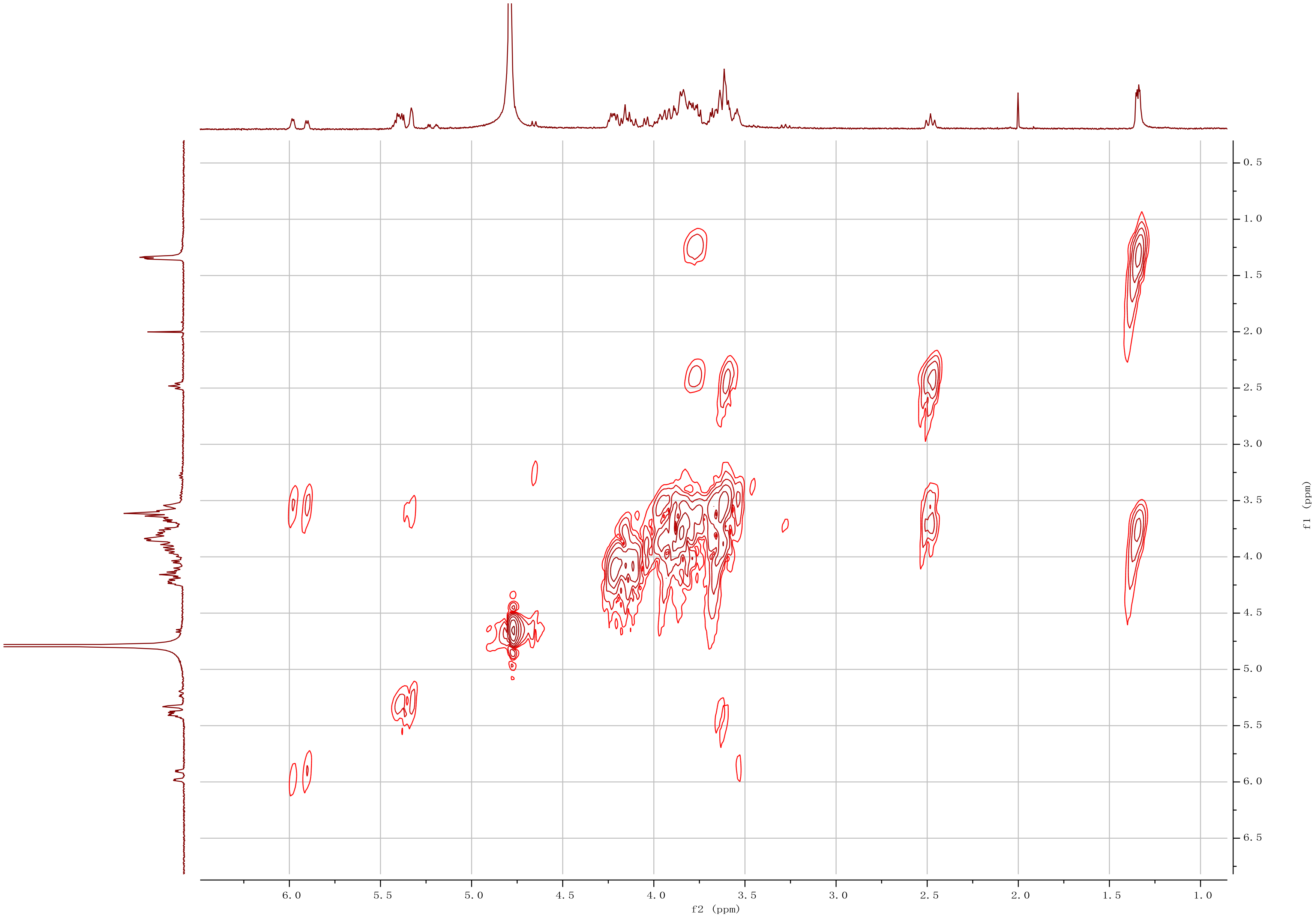

Figure S22. ^{1}H-^{1}H COSY spectrum of compound **10** (500 MHz, D_2O).

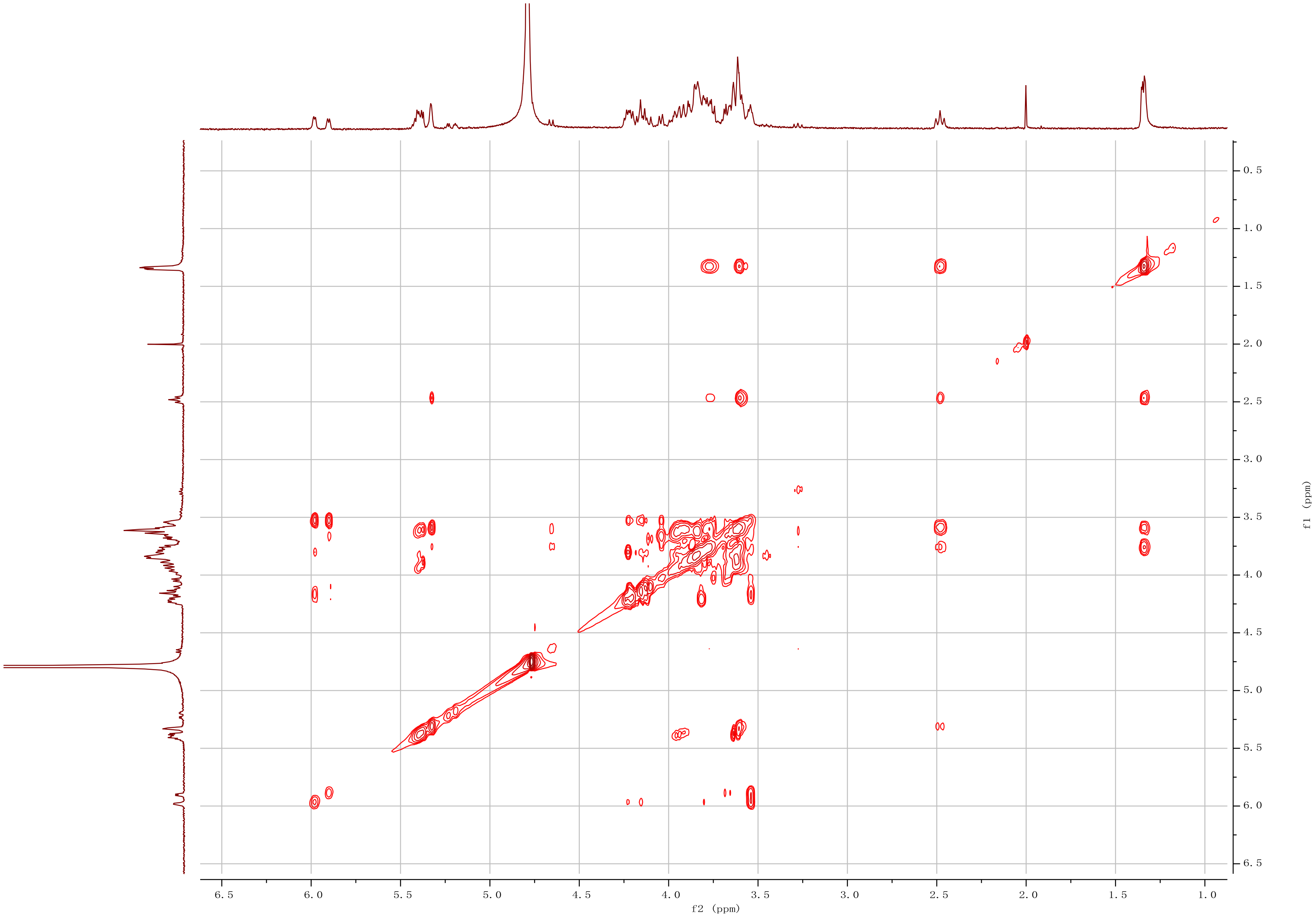

Figure S23. 2D-TOCSY spectrum of compound **10** (500 MHz, D_2O).

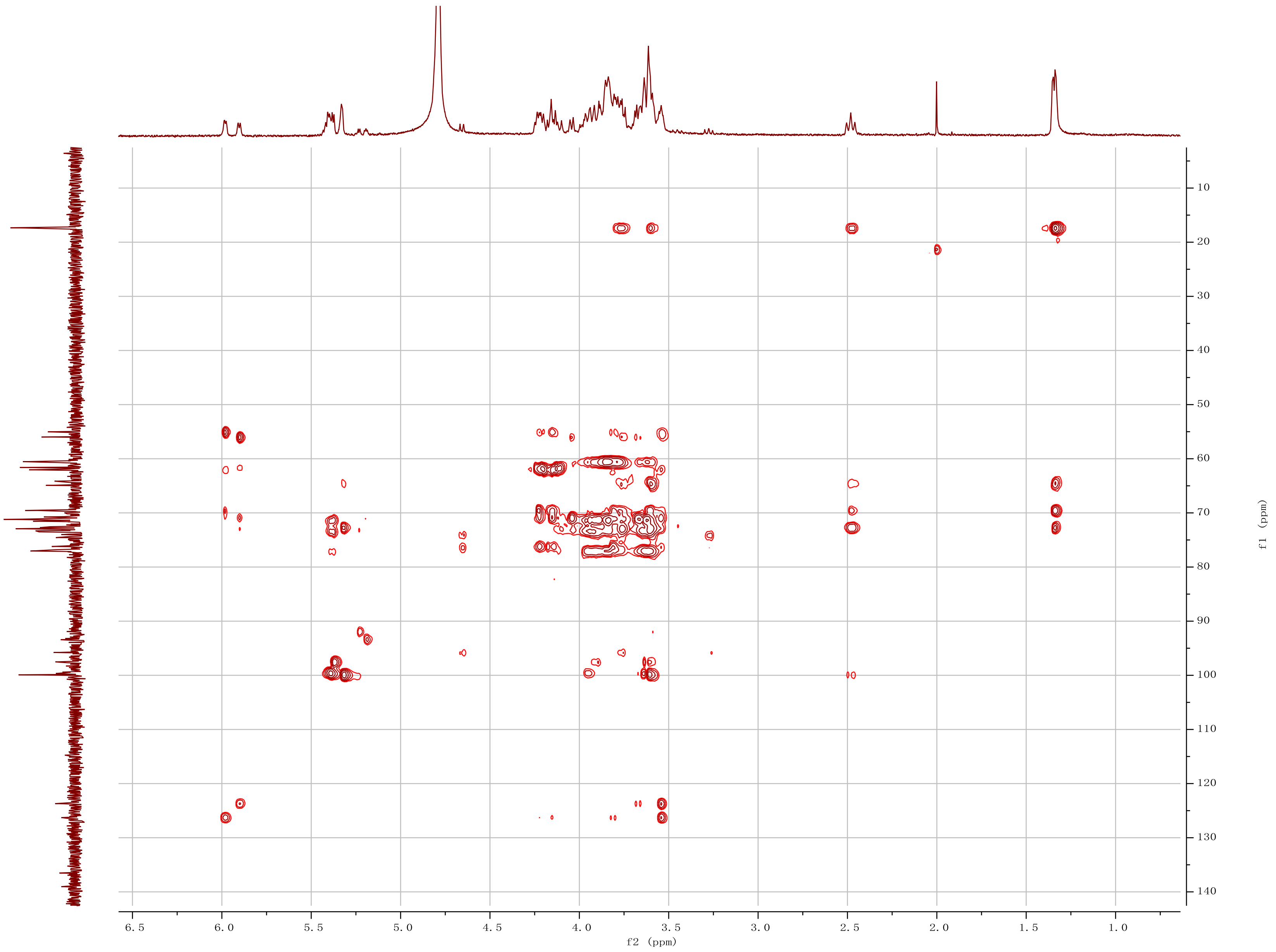

Figure S24. HSQC-TOCSY spectrum of compound **10** (500 MHz, D_2O).

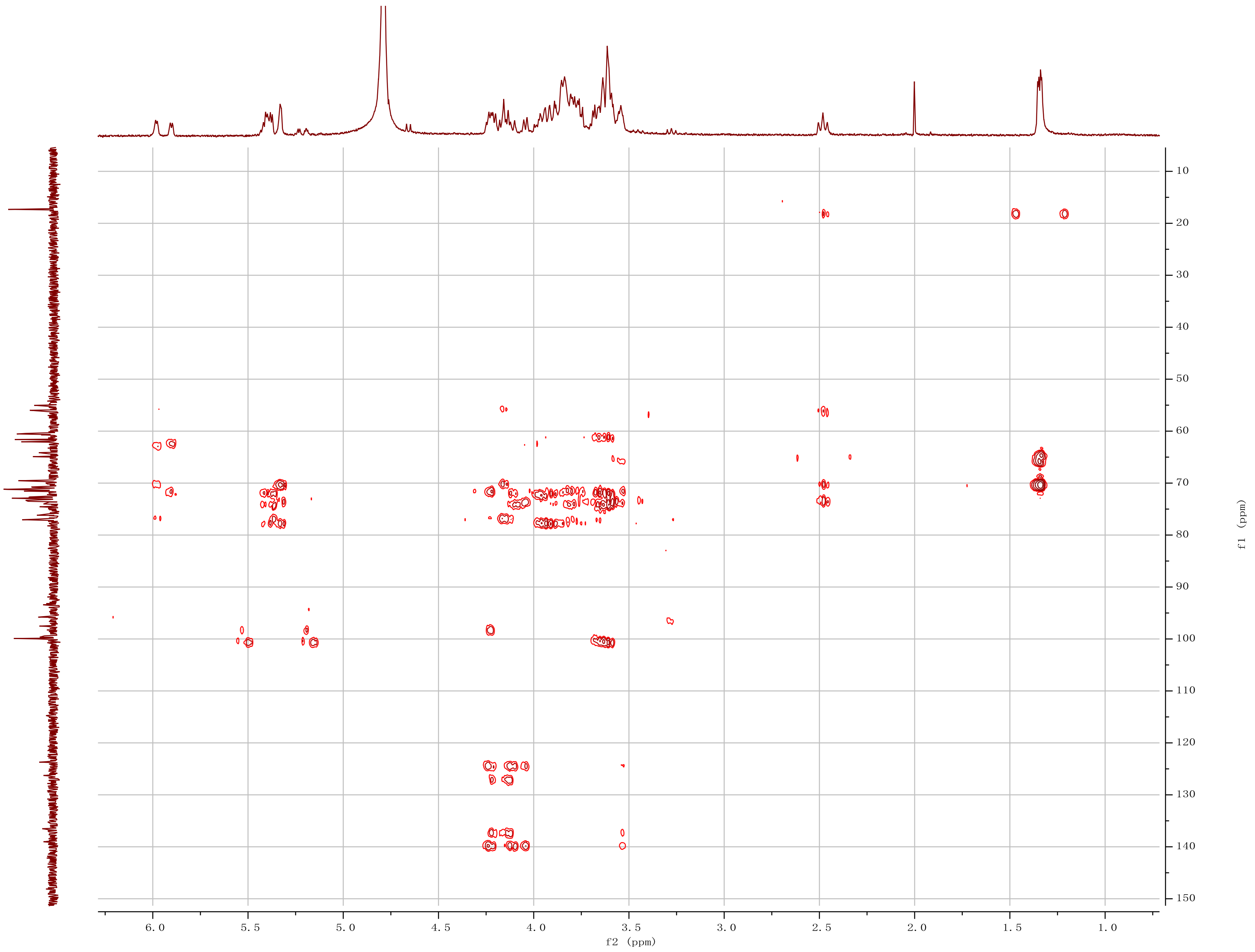

Figure S25. HMBC spectrum of compound **10** (500 MHz, D_2O).

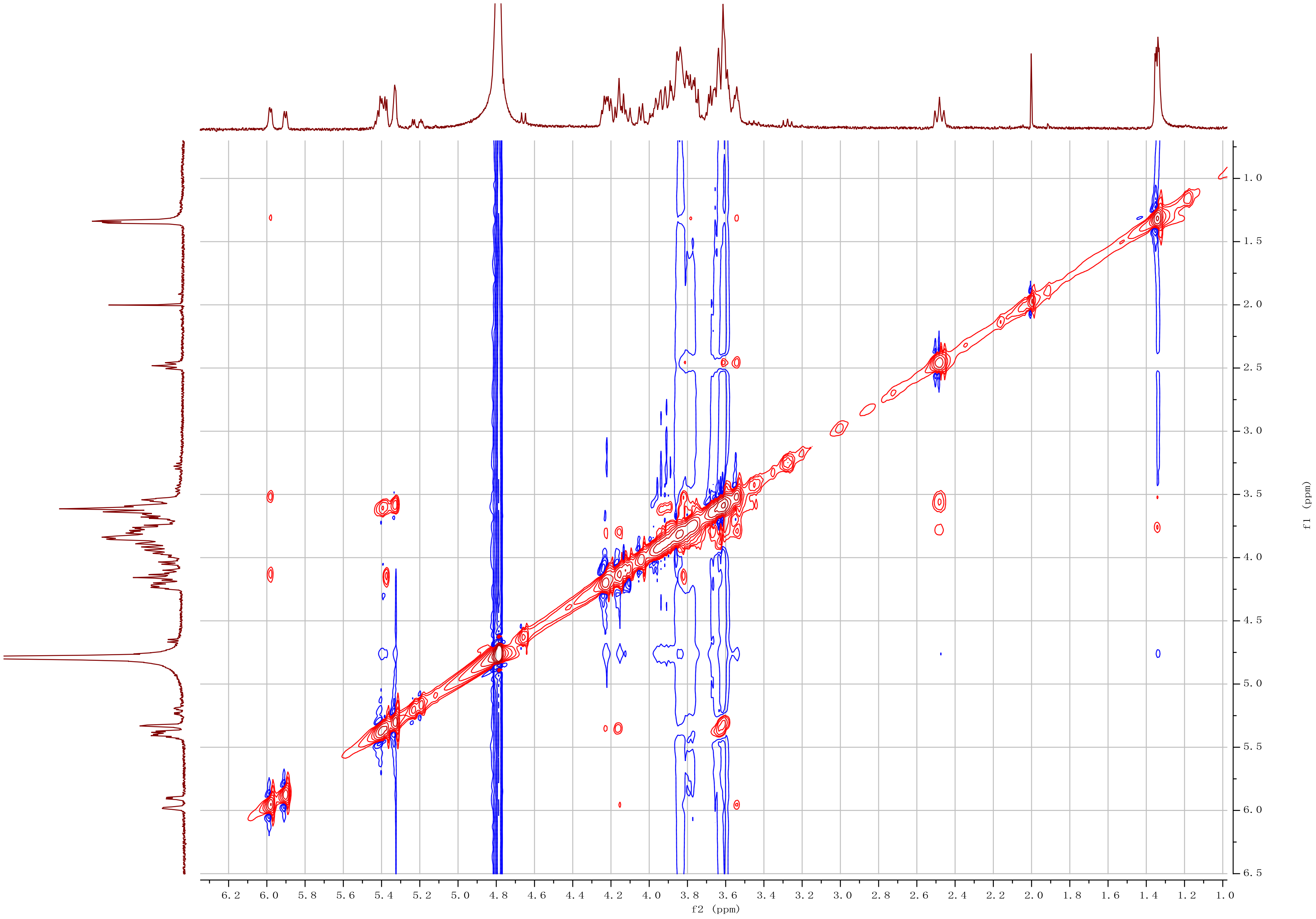

Figure S26. NOESY spectrum of compound **10** (500 MHz, D_2O).

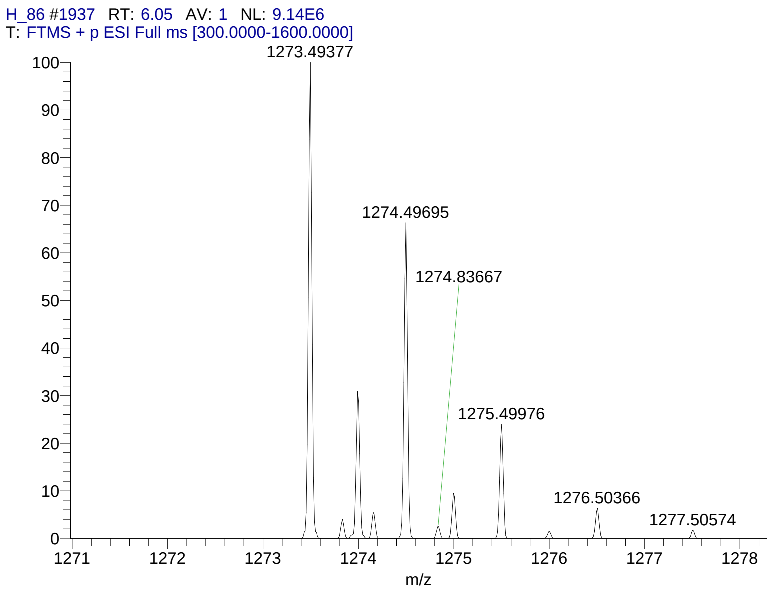

Figure S27. HRESIMS spectrum of compound **10**.

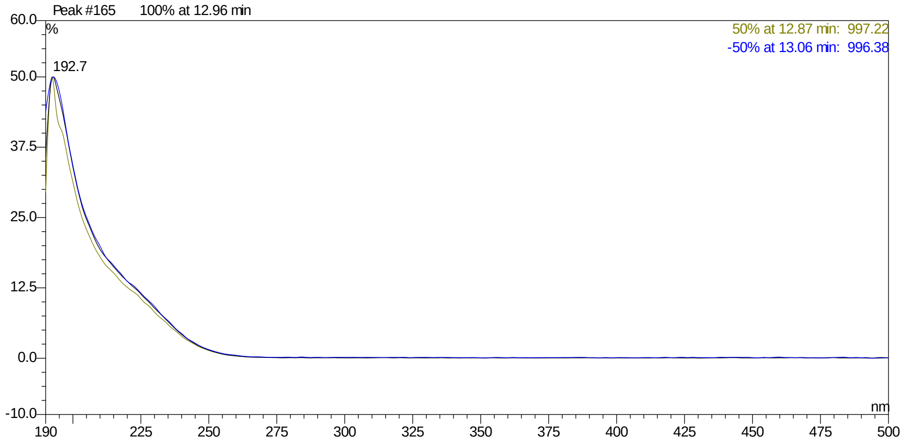

Figure S28. UV spectrum of compound **10**.

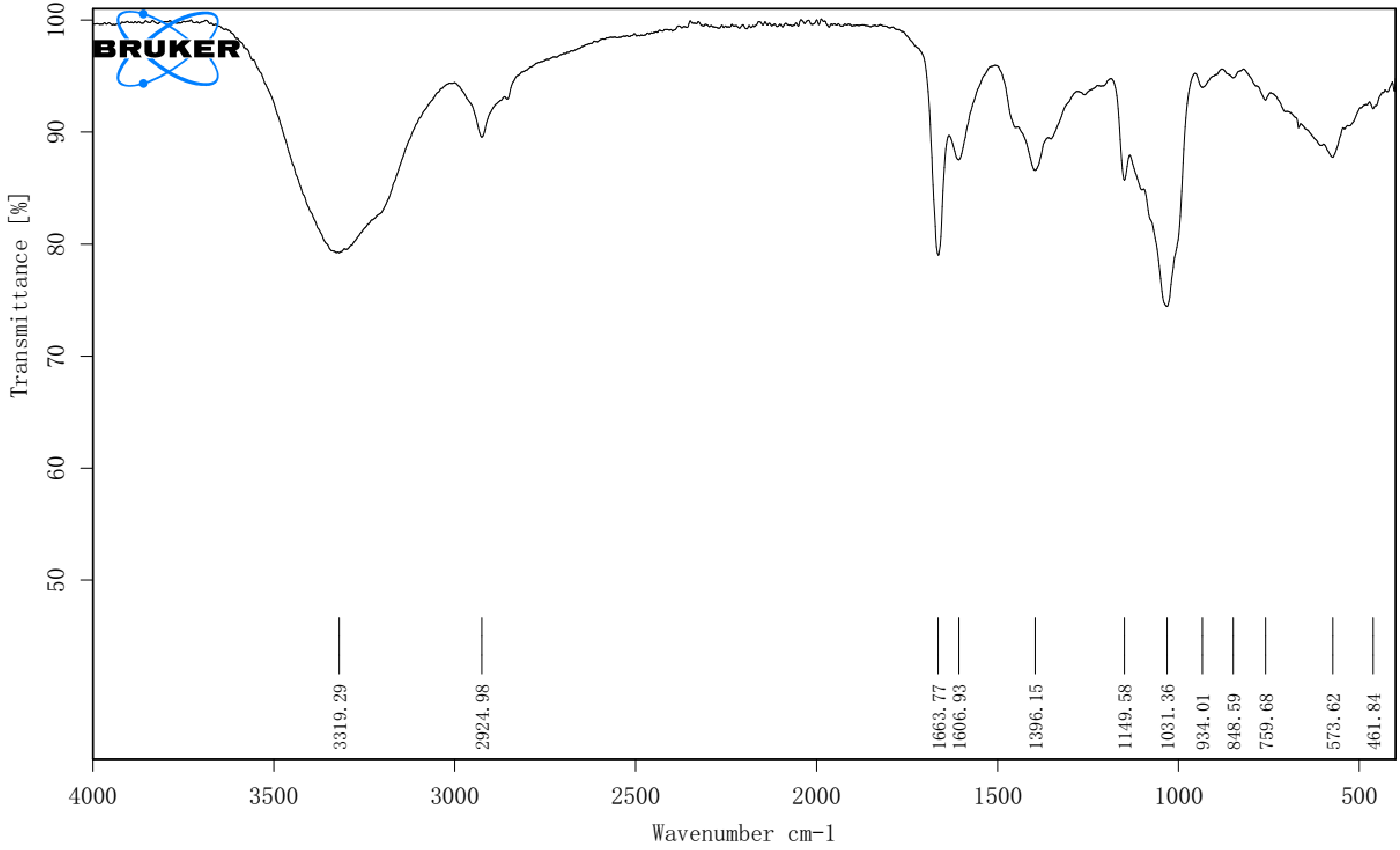

Figure S29. IR spectrum of compound **10**.

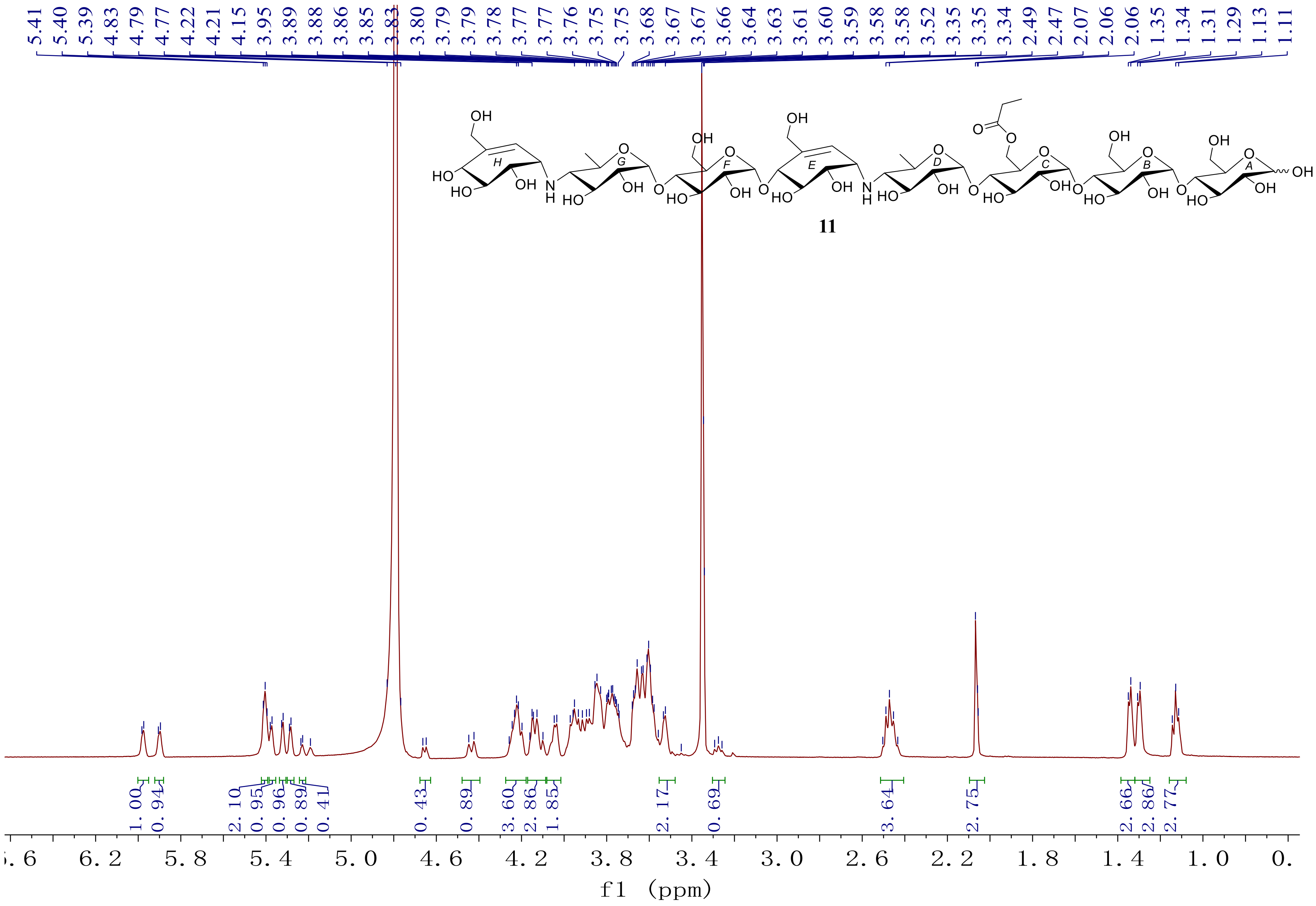

Figure S30. ^{1}H NMR spectrum of compound **11** (500 MHz, D_2O).

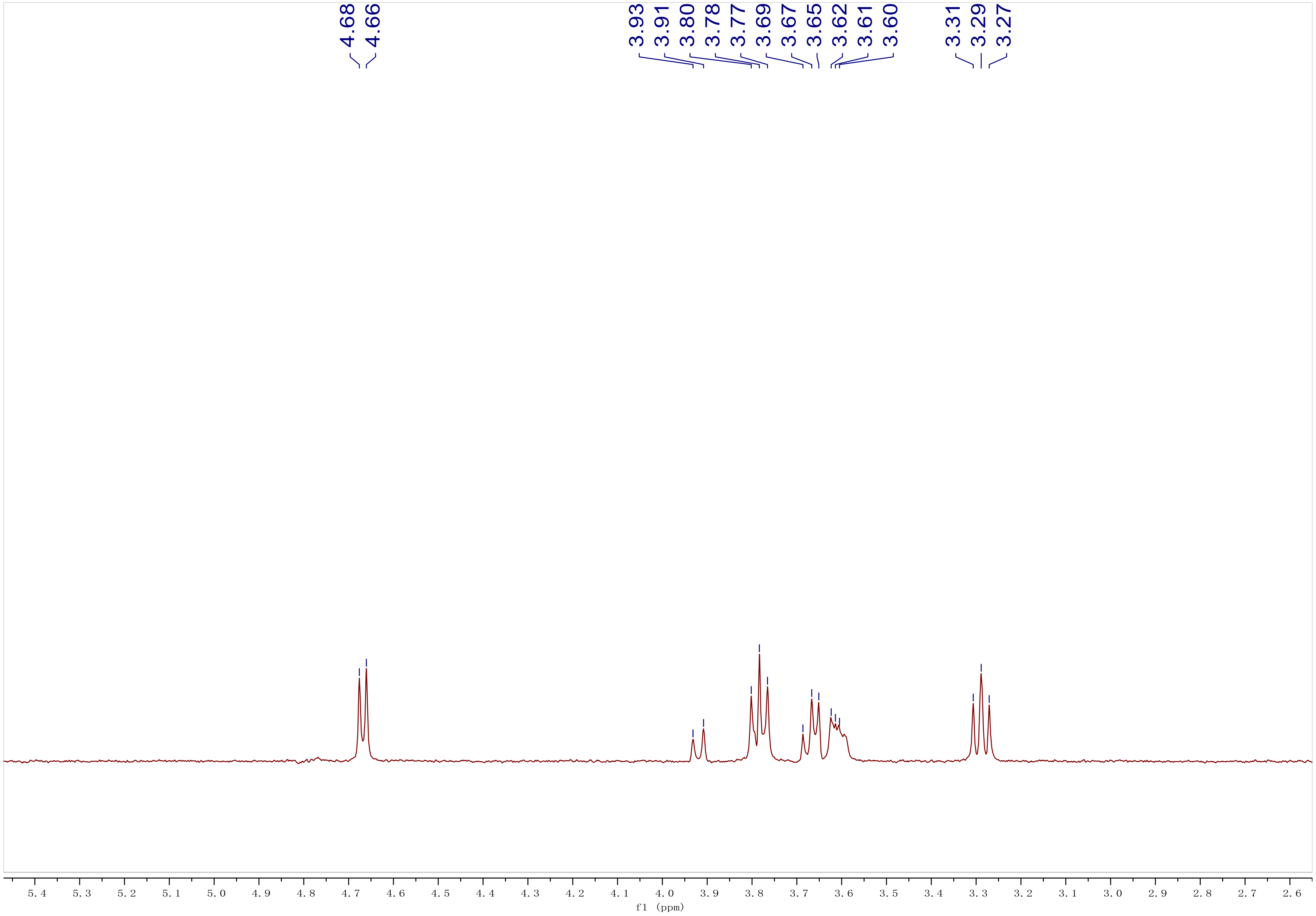

Figure S31. 1D-selective TOCSY spectrum of compound **11** (500 MHz, D_2O, excitation at δ 4.66, H-**A**1β).

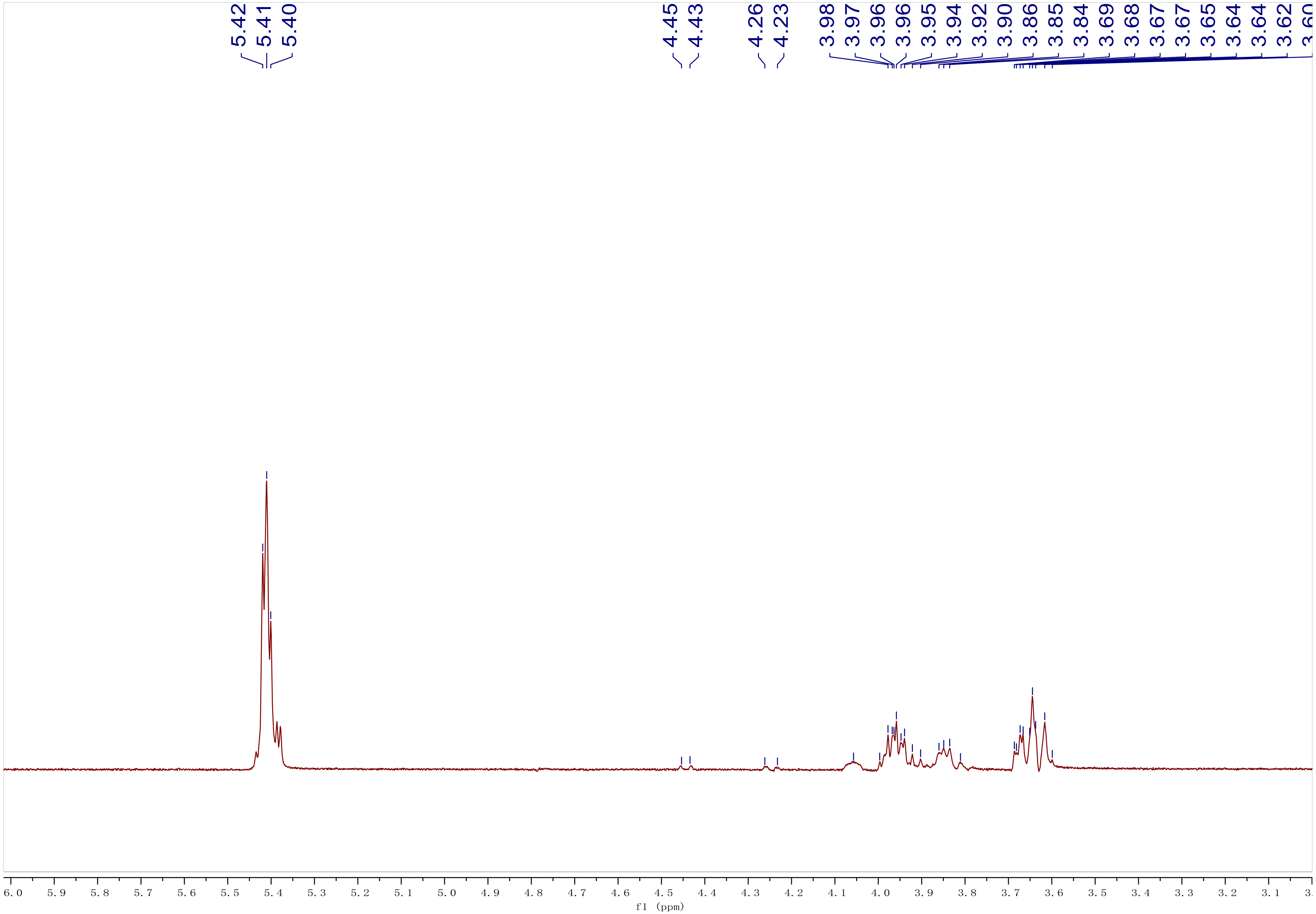

Figure S32. 1D-selective TOCSY spectrum of compound **11** (500 MHz, D_2O, excitation at δ 5.41, H-**B**1 and H-**C**1).

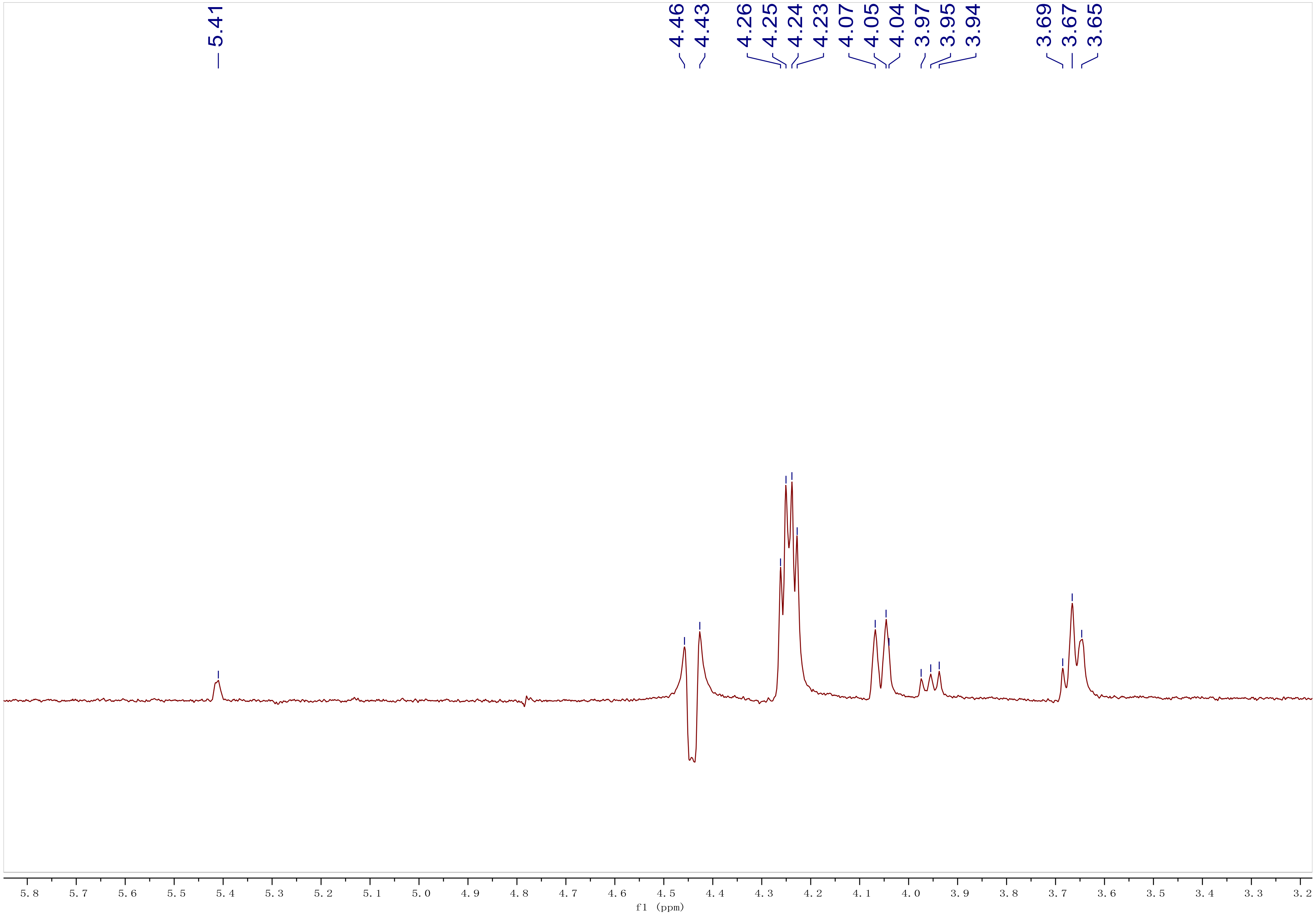

Figure S33. 1D-selective TOCSY spectrum of compound **11** (500 MHz, D_2O, excitation at δ 4.44, H-**C**6a).

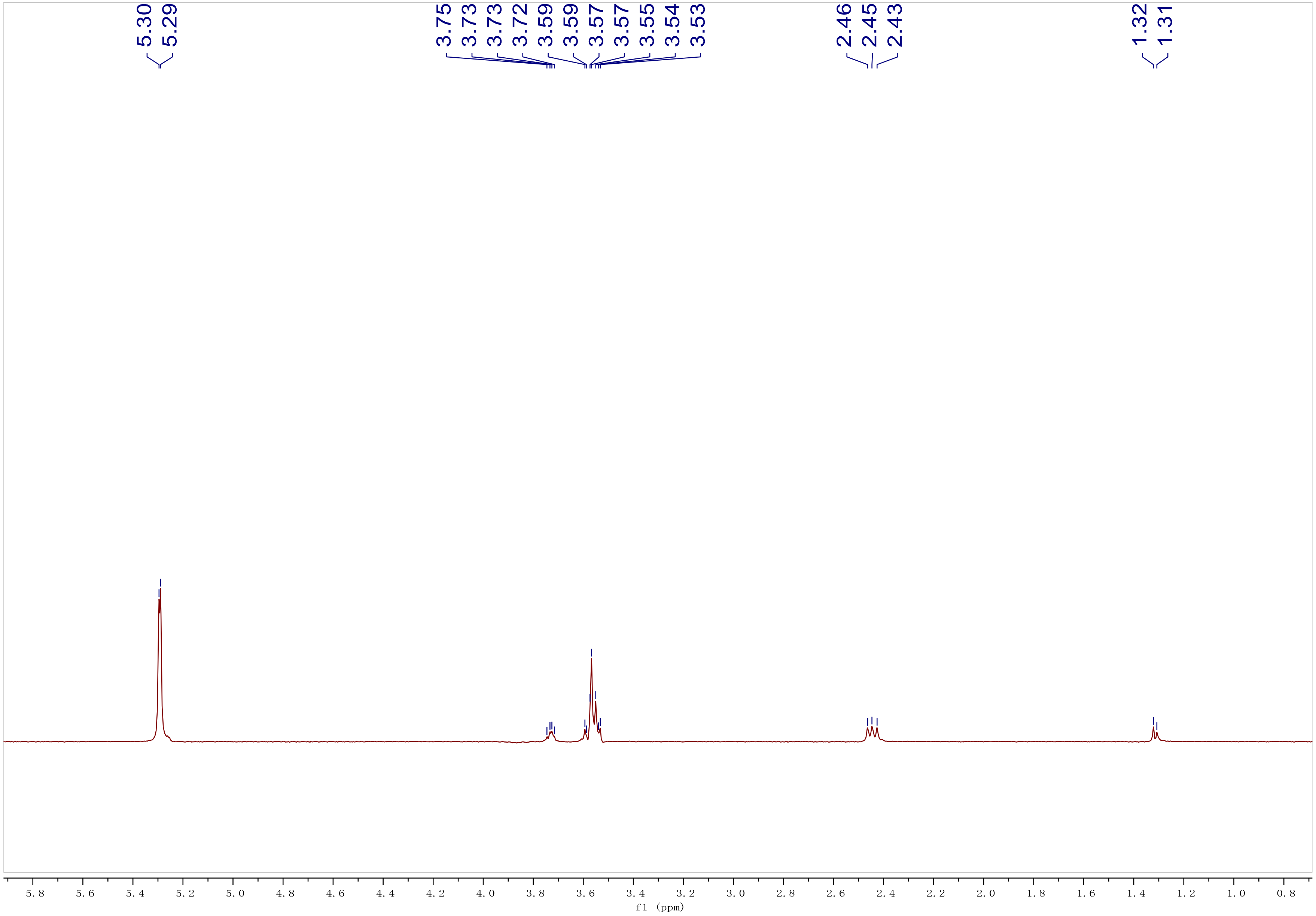

Figure S34. 1D-selective TOCSY spectrum of compound **11** (500 MHz, D_2O, excitation at δ 5.29, H-**D**1).

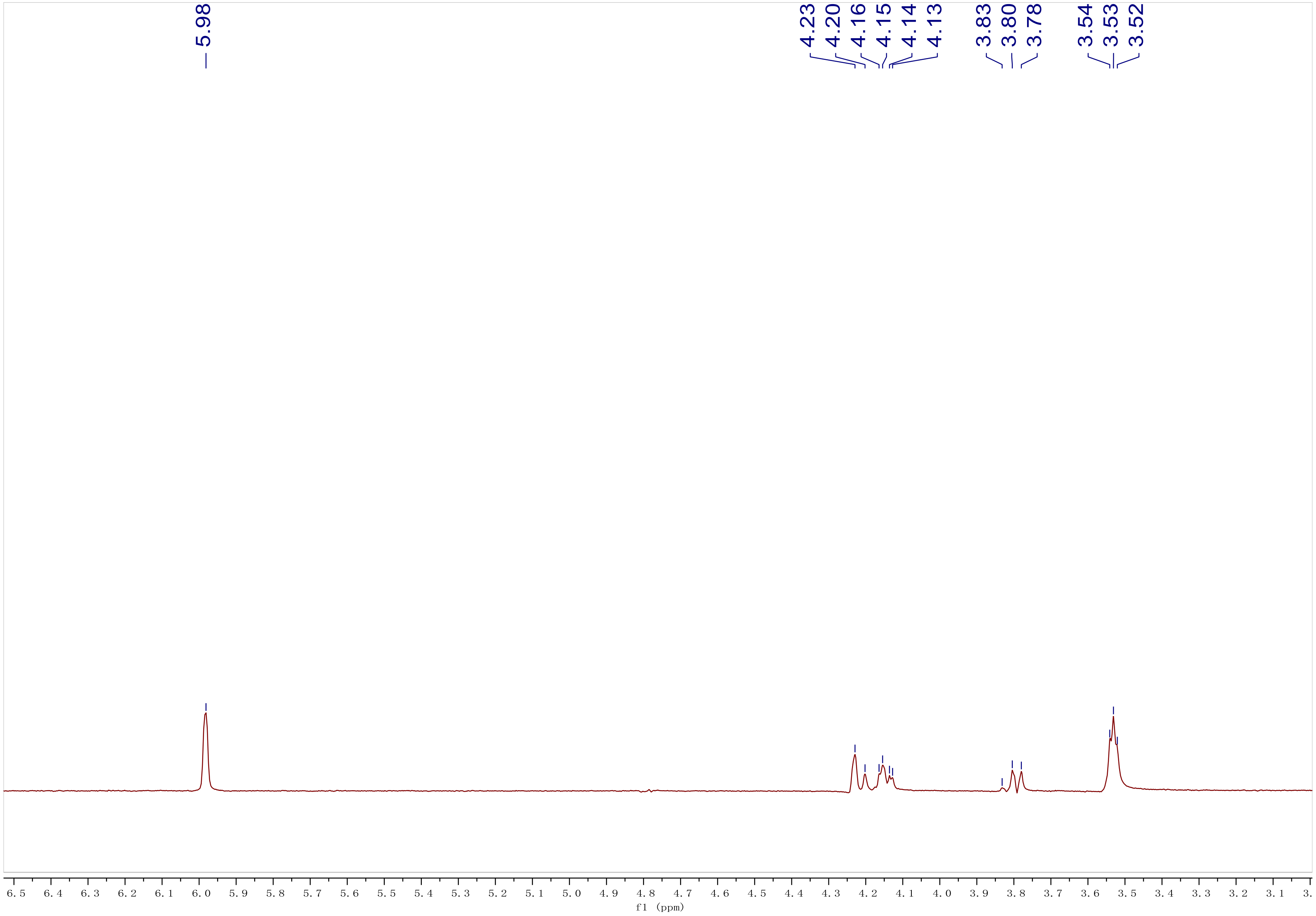

Figure S35. 1D-selective TOCSY spectrum of compound **11** (500 MHz, D_2O, excitation at δ 5.98, H-**E**7).

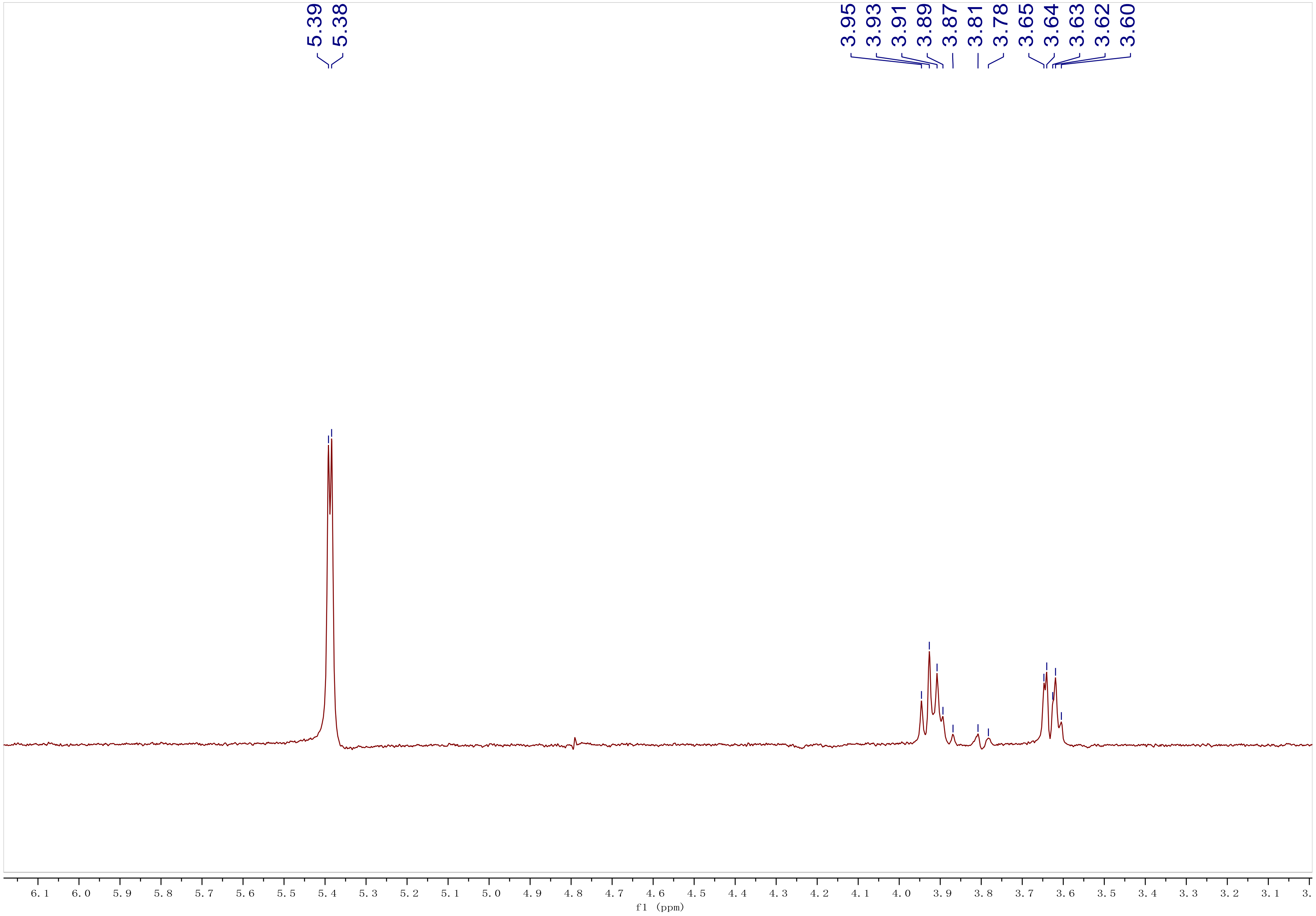

Figure S36. 1D-selective TOCSY spectrum of compound **11** (500 MHz, D_2O, excitation at δ 5.38, H-**F**1).

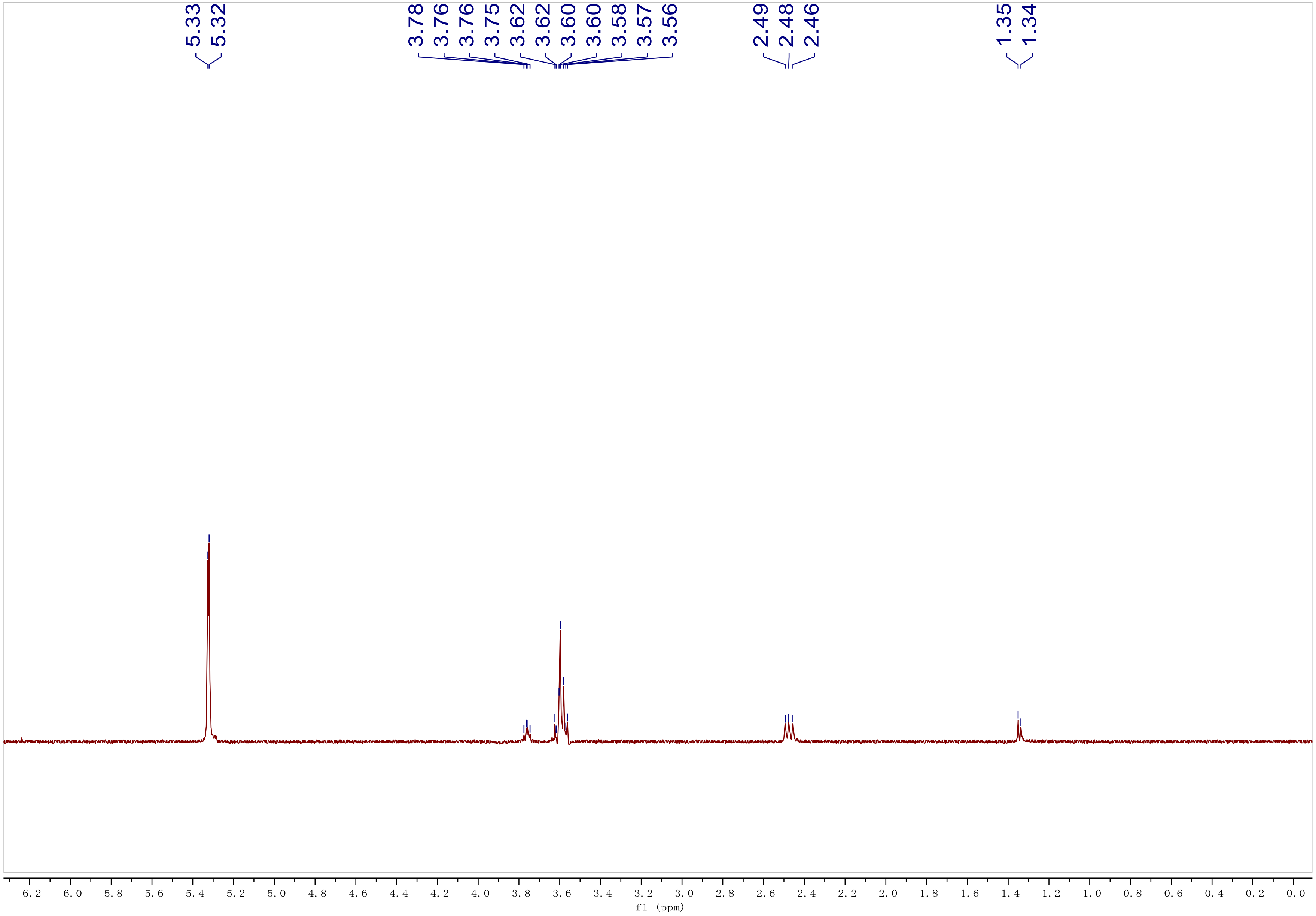

Figure S37. 1D-selective TOCSY spectrum of compound **11** (500 MHz, D_2O, excitation at δ 5.32, H-**G**1).

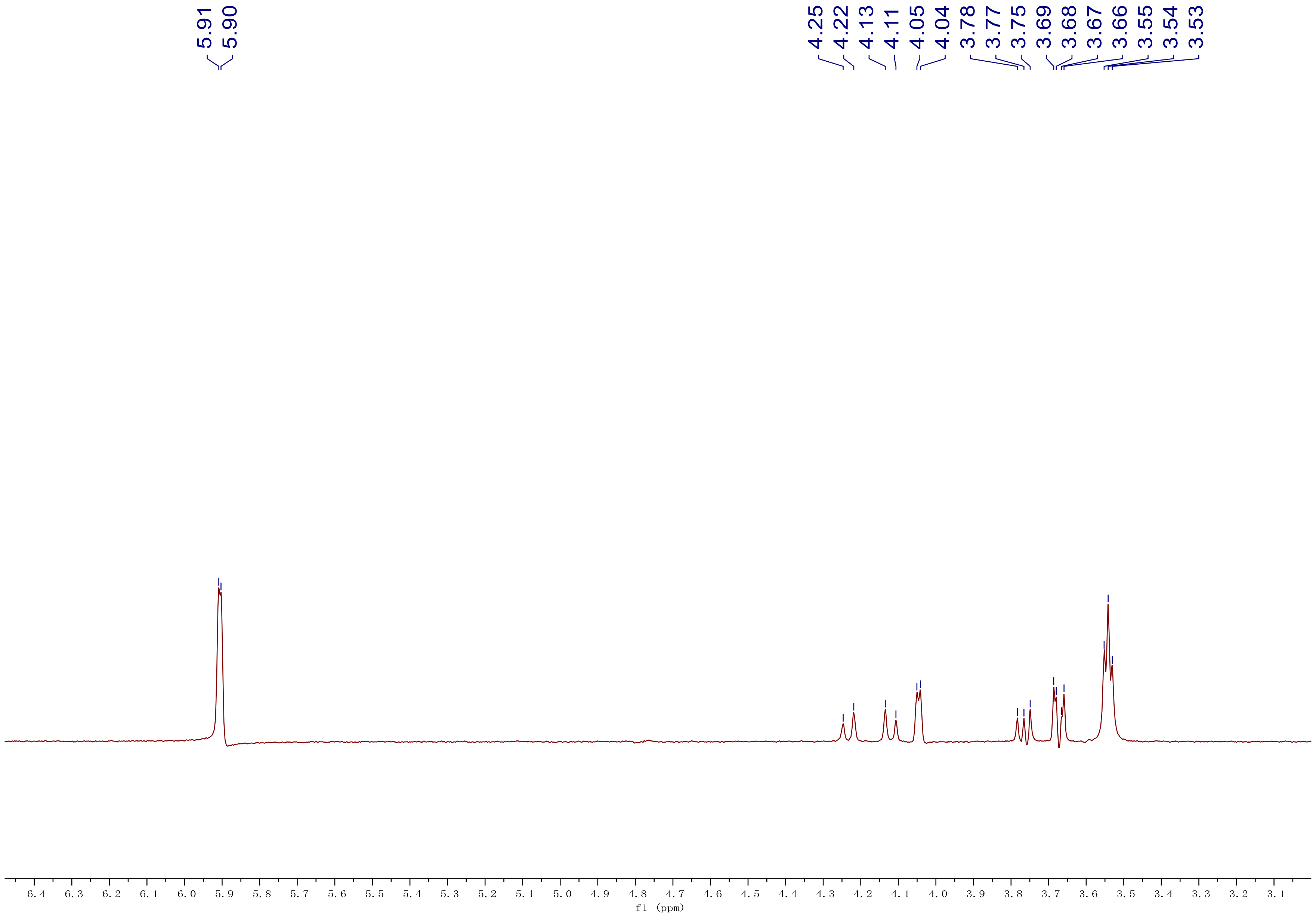

Figure S38. 1D-selective TOCSY spectrum of compound **11** (500 MHz, D_2O, excitation at δ 5.90, H-**H**7).

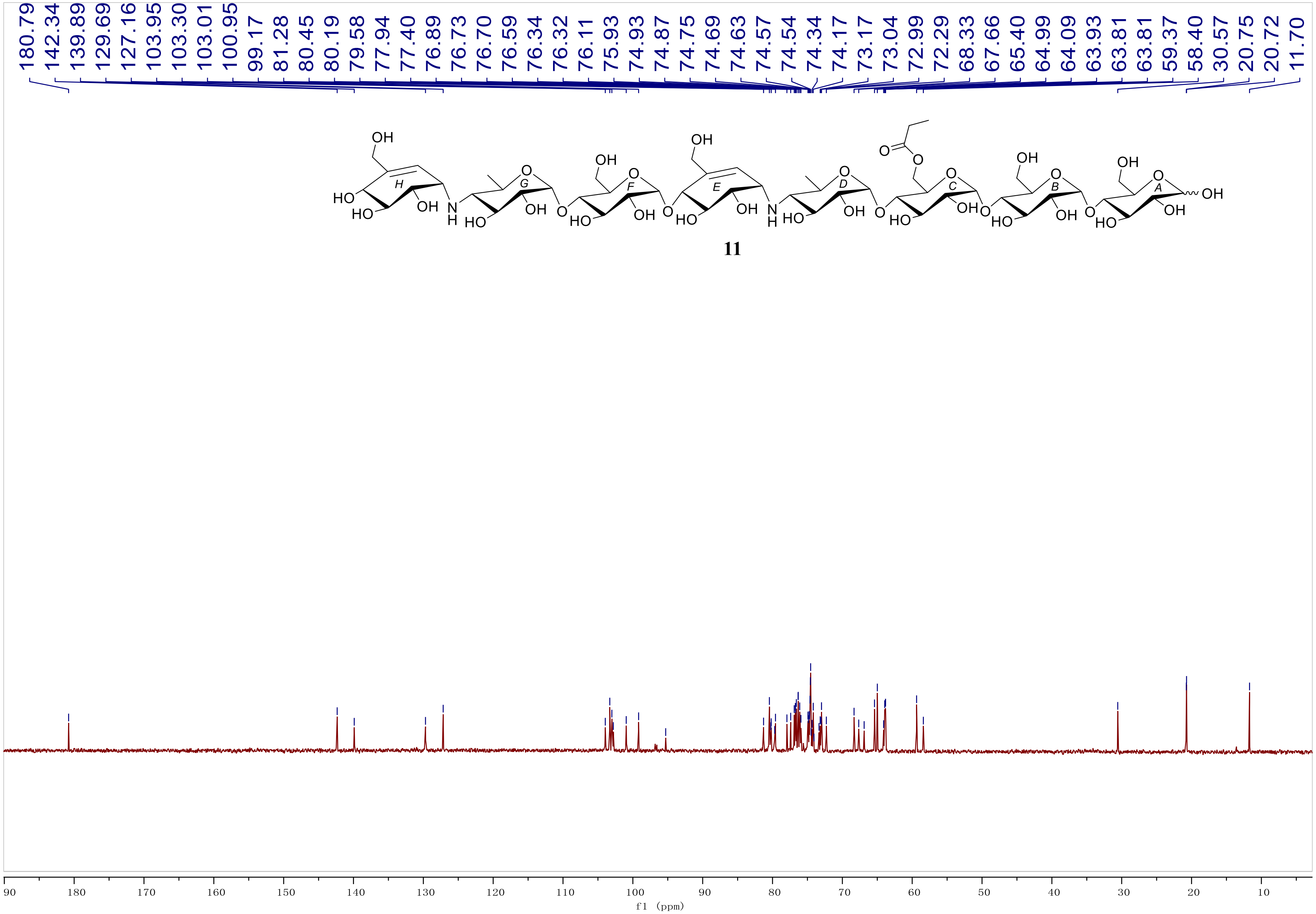

Figure S39. ^{13}C NMR spectrum of compound **11** (125 MHz, D_2O).

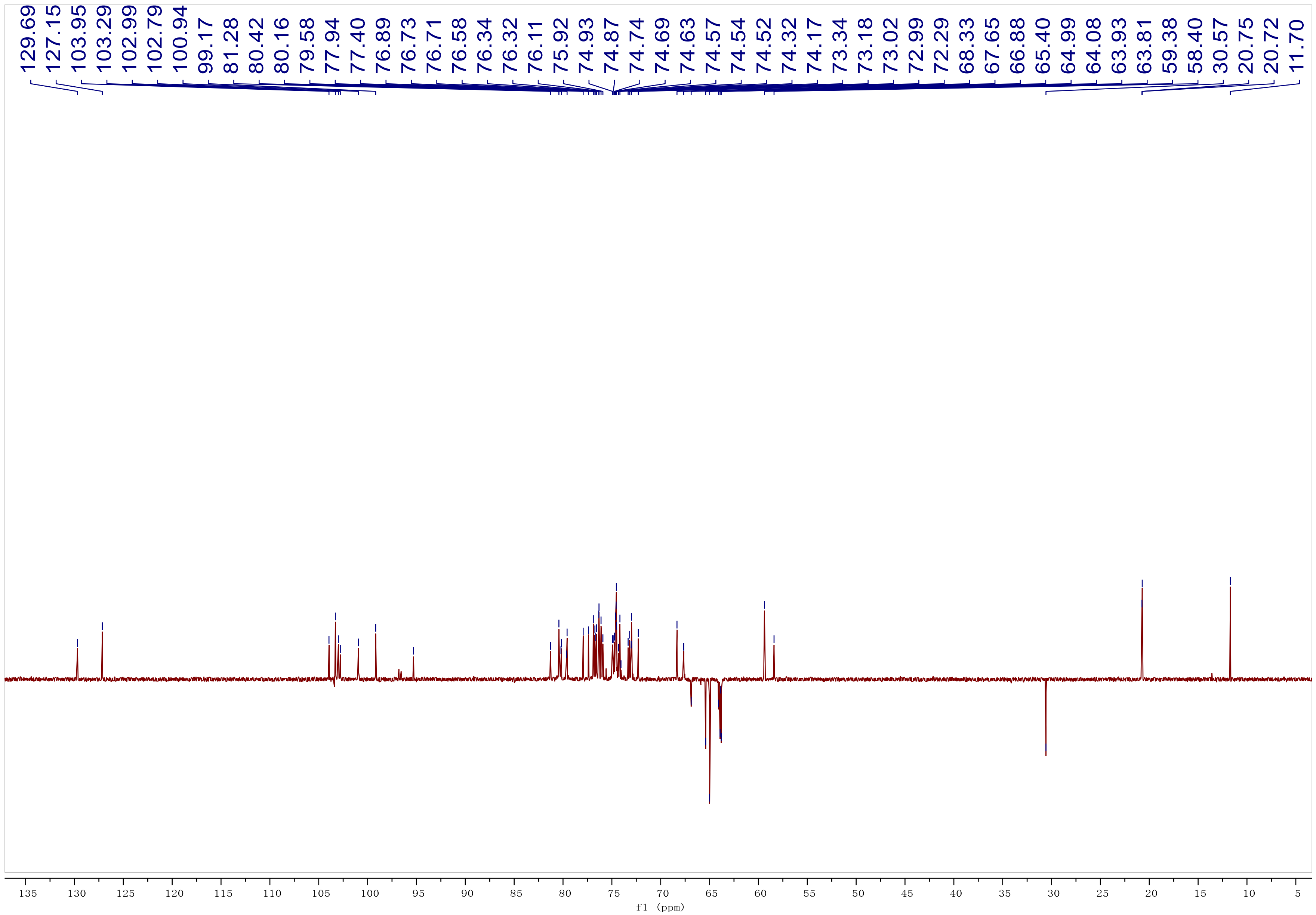

Figure S40. DEPT-135 spectrum of compound **11** (125 MHz, D_2O).

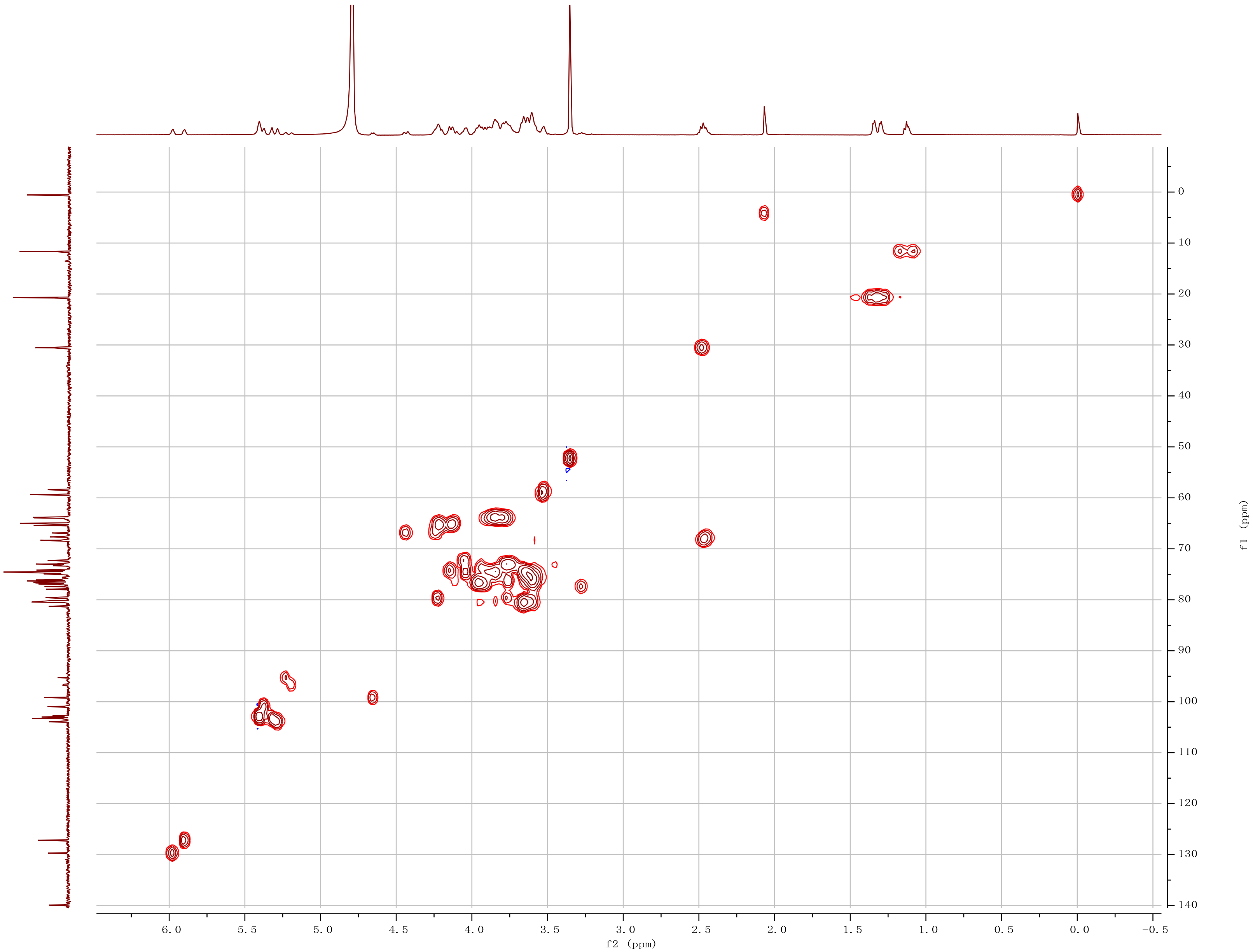

Figure S41. HSQC spectrum of compound **11** (500 MHz, D_2O).

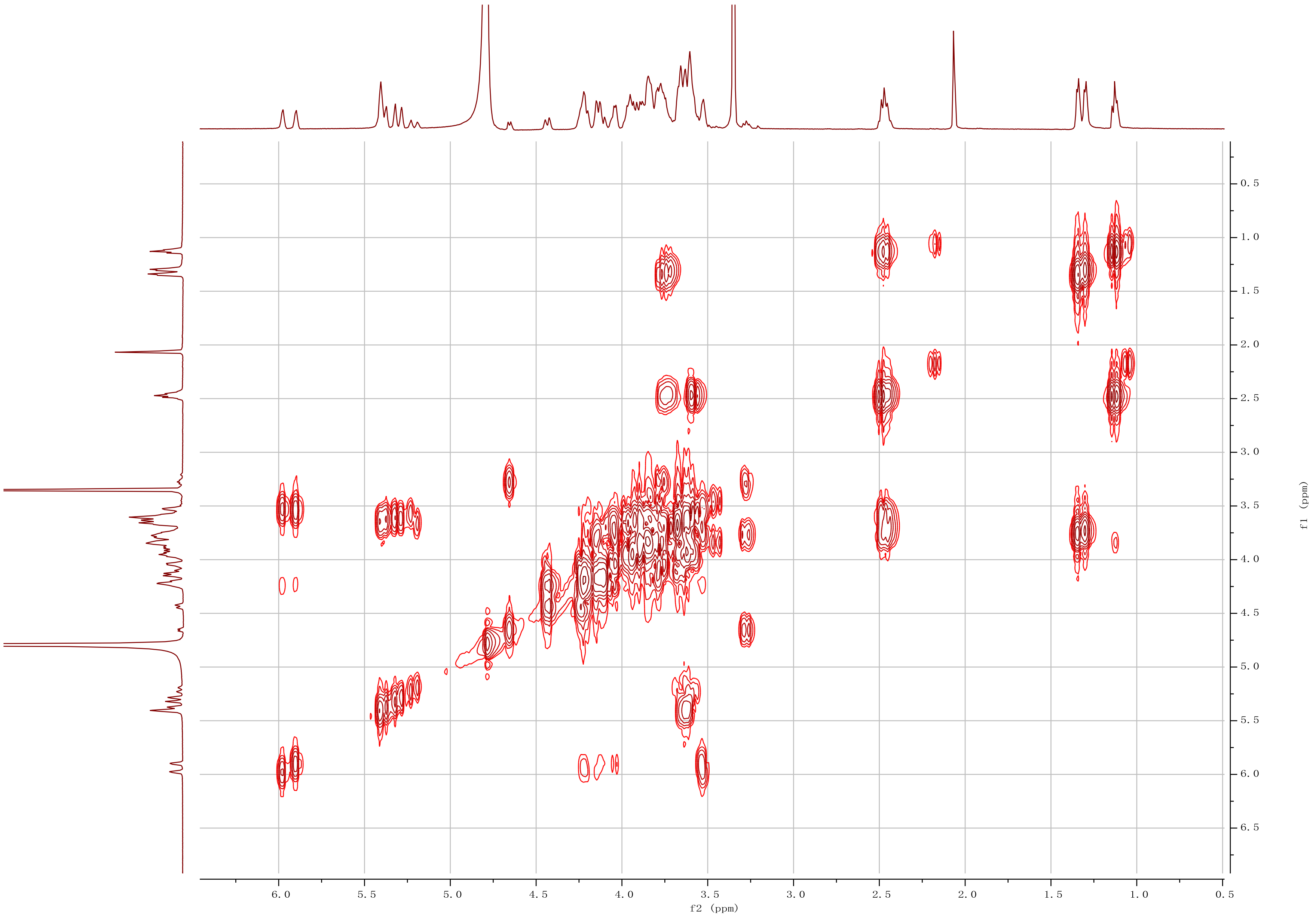

Figure S42. ^{1}H-^{1}H COSY spectrum of compound **11** (500 MHz, D_2O).

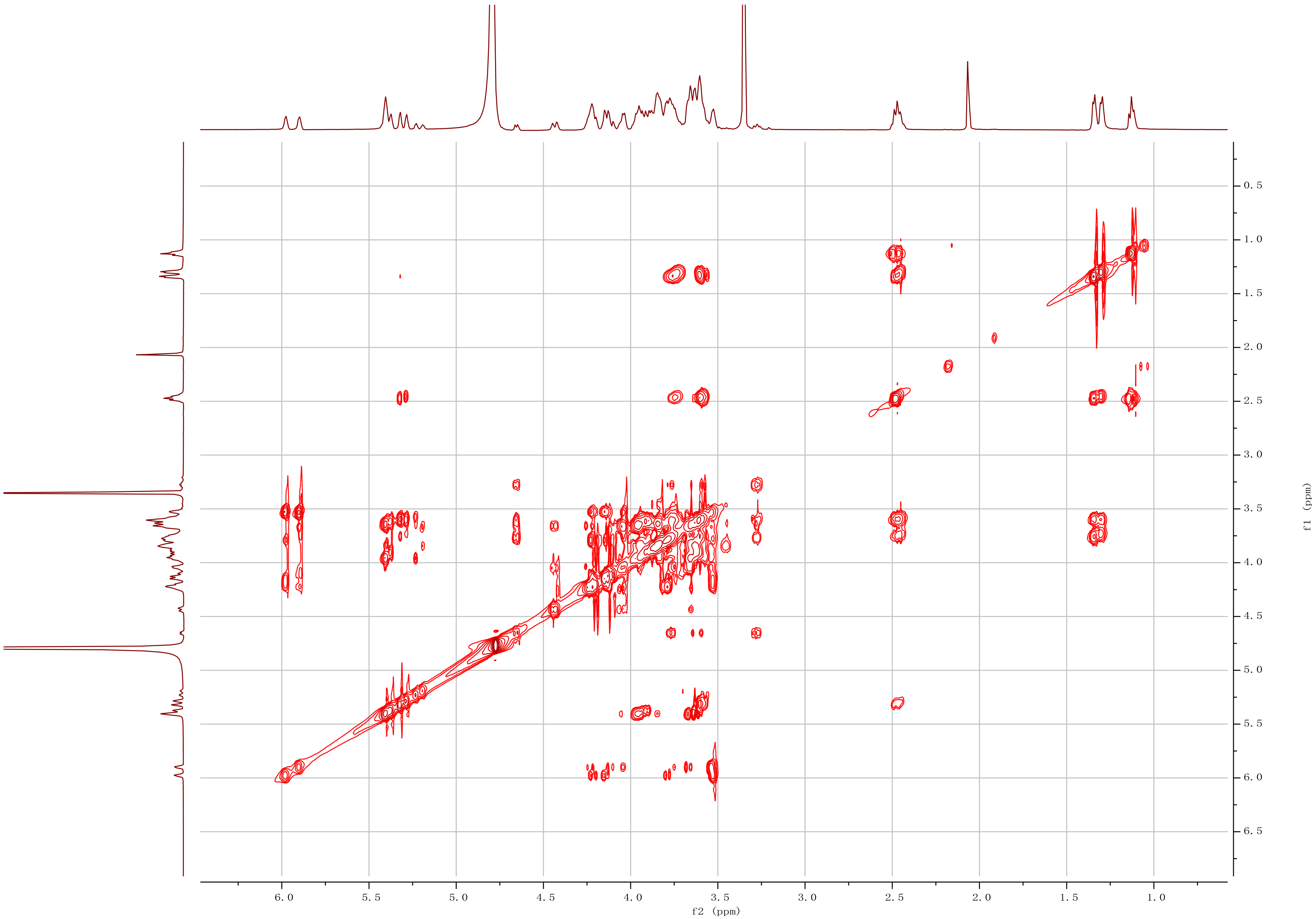

Figure S43. 2D-TOCSY spectrum of compound **11** (500 MHz, D_2O).

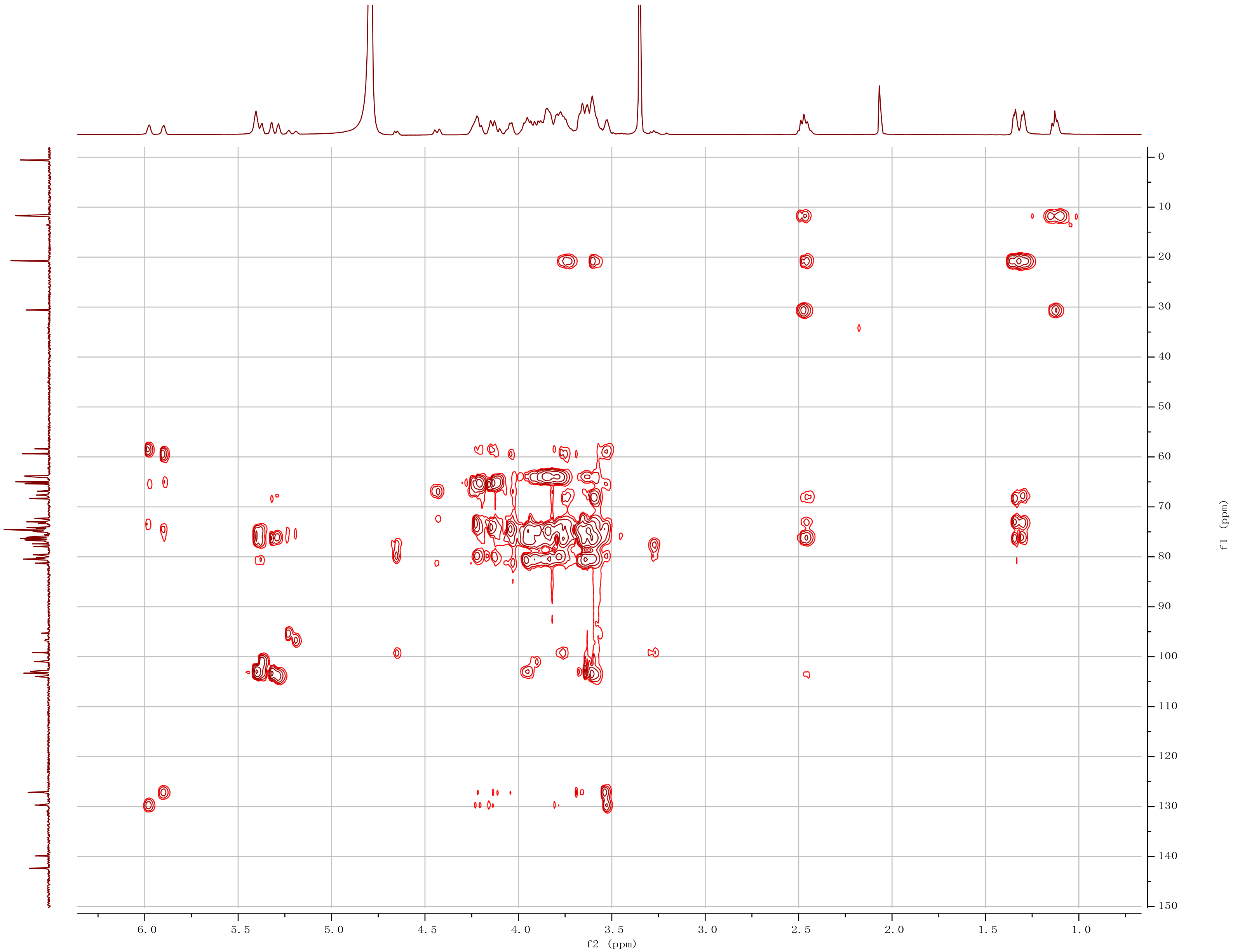

Figure S44. HSQC-TOCSY spectrum of compound **11** (500 MHz, D_2O).

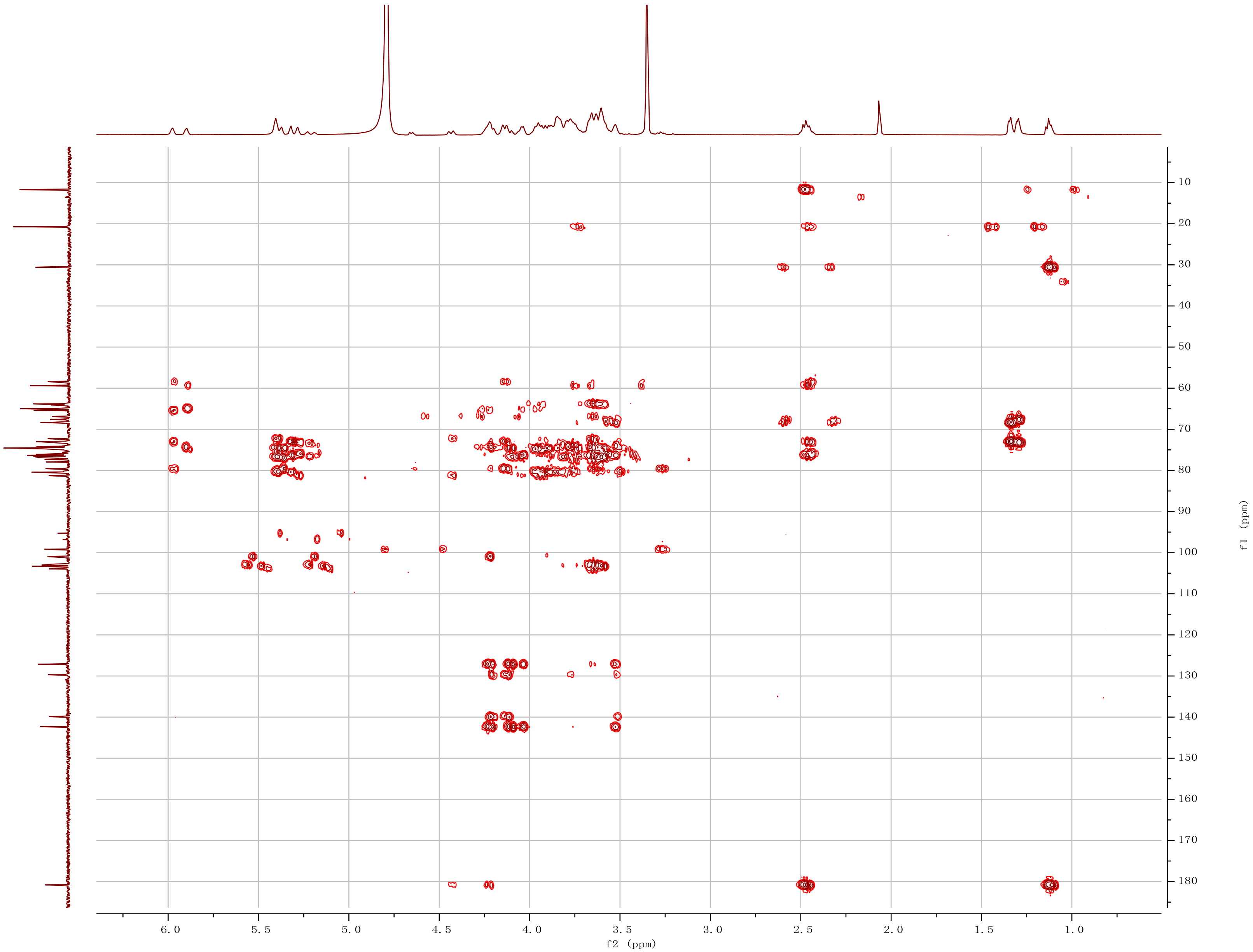

Figure S45. HMBC spectrum of compound **11** (500 MHz, D_2O).

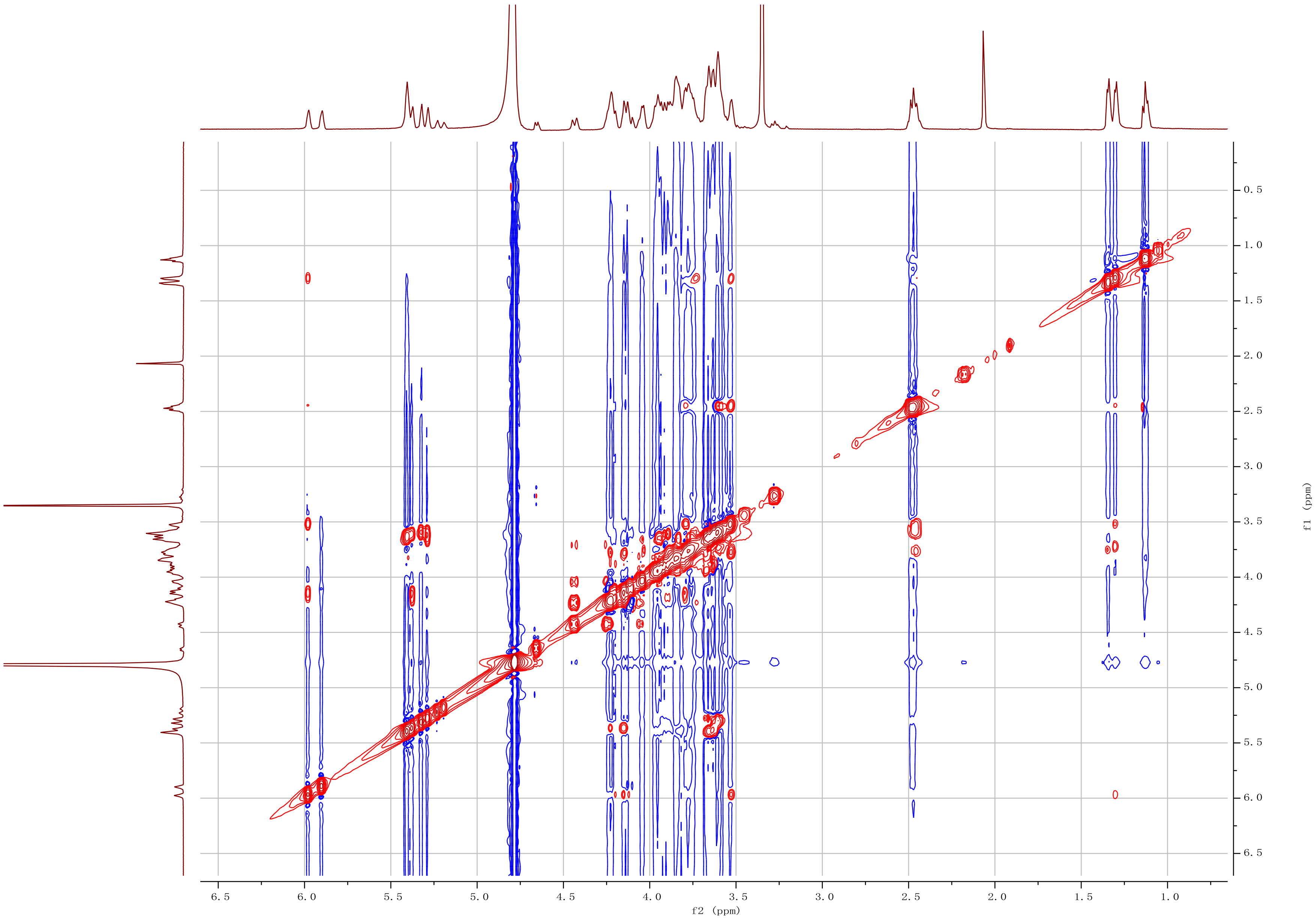

Figure S46. NOESY spectrum of compound **11** (500 MHz, D_2O).

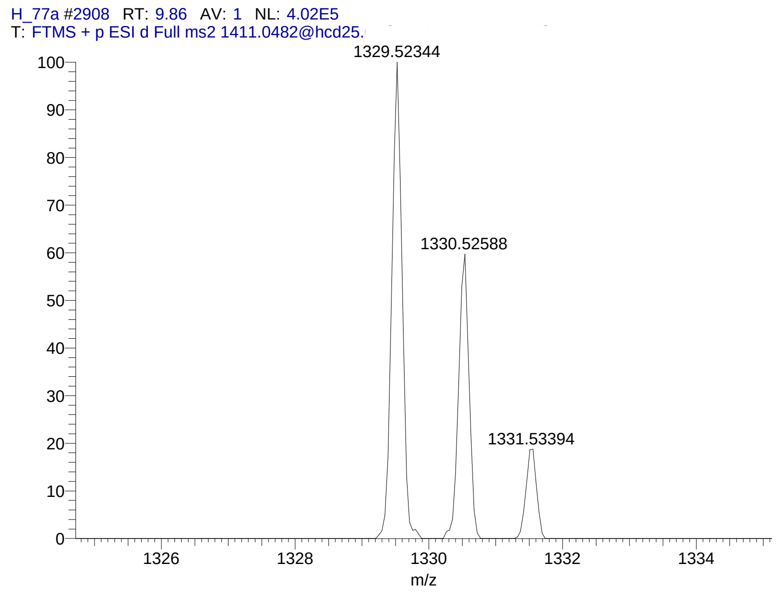

Figure S47. HRESIMS spectrum of compound **11**.

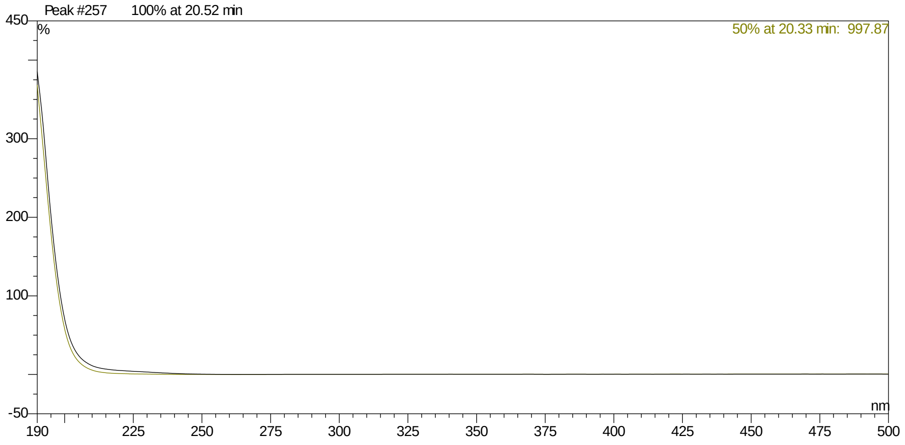

Figure S48. UV spectrum of compound **11**.

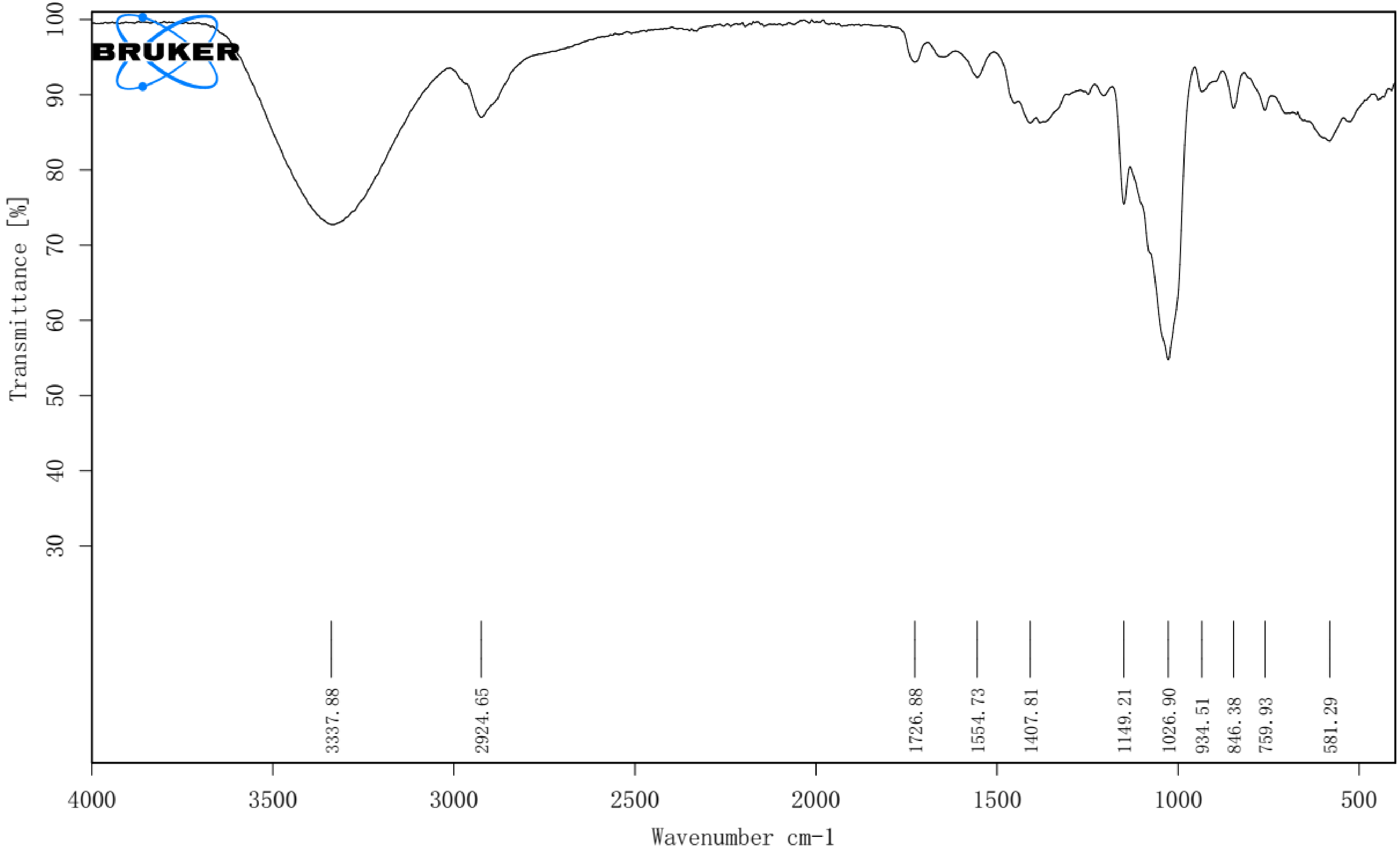

Figure S49. IR spectrum of compound **11**.

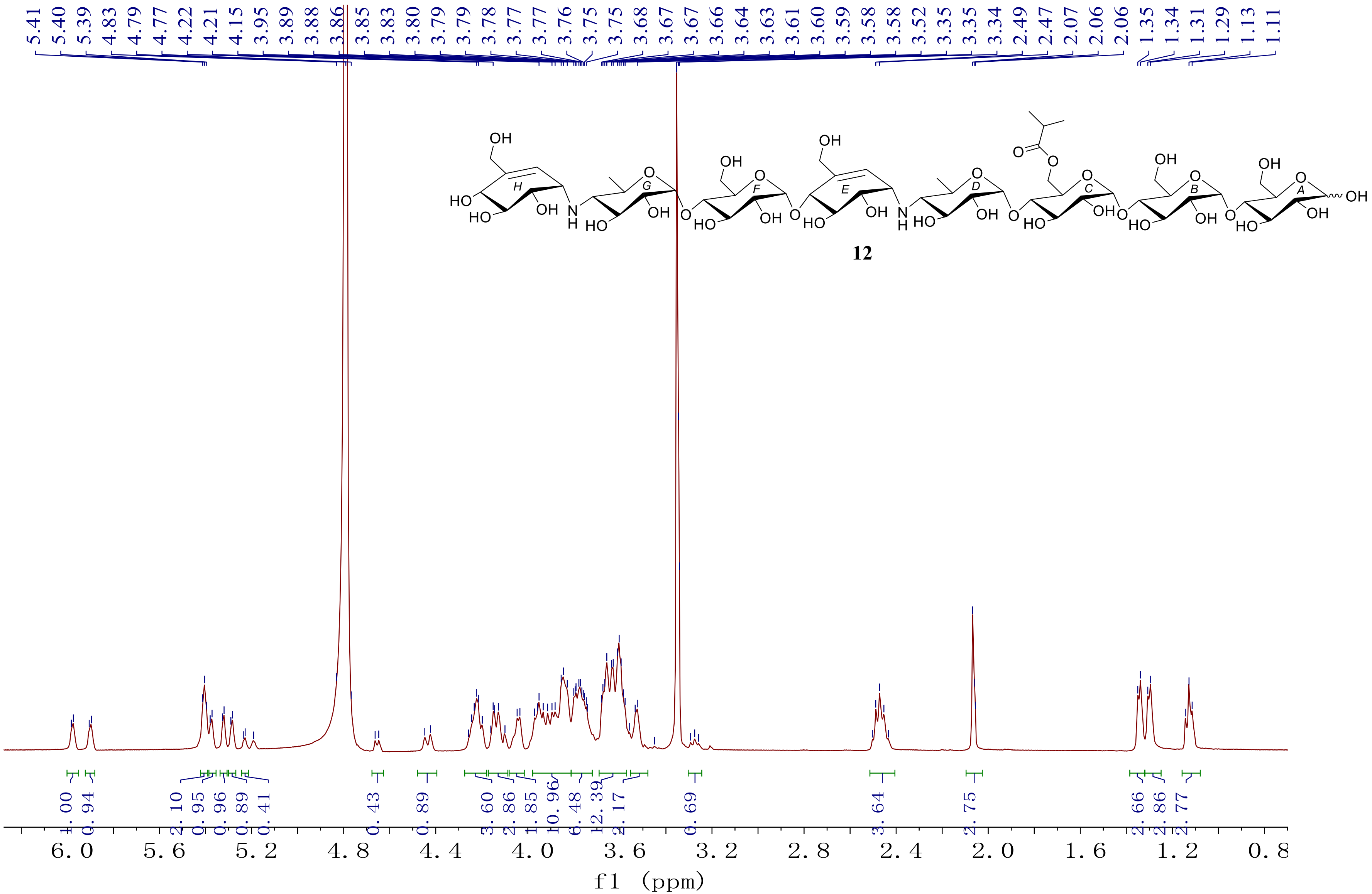

Figure S50. ^{1}H NMR spectrum of compound **12** (500 MHz, D_2O).

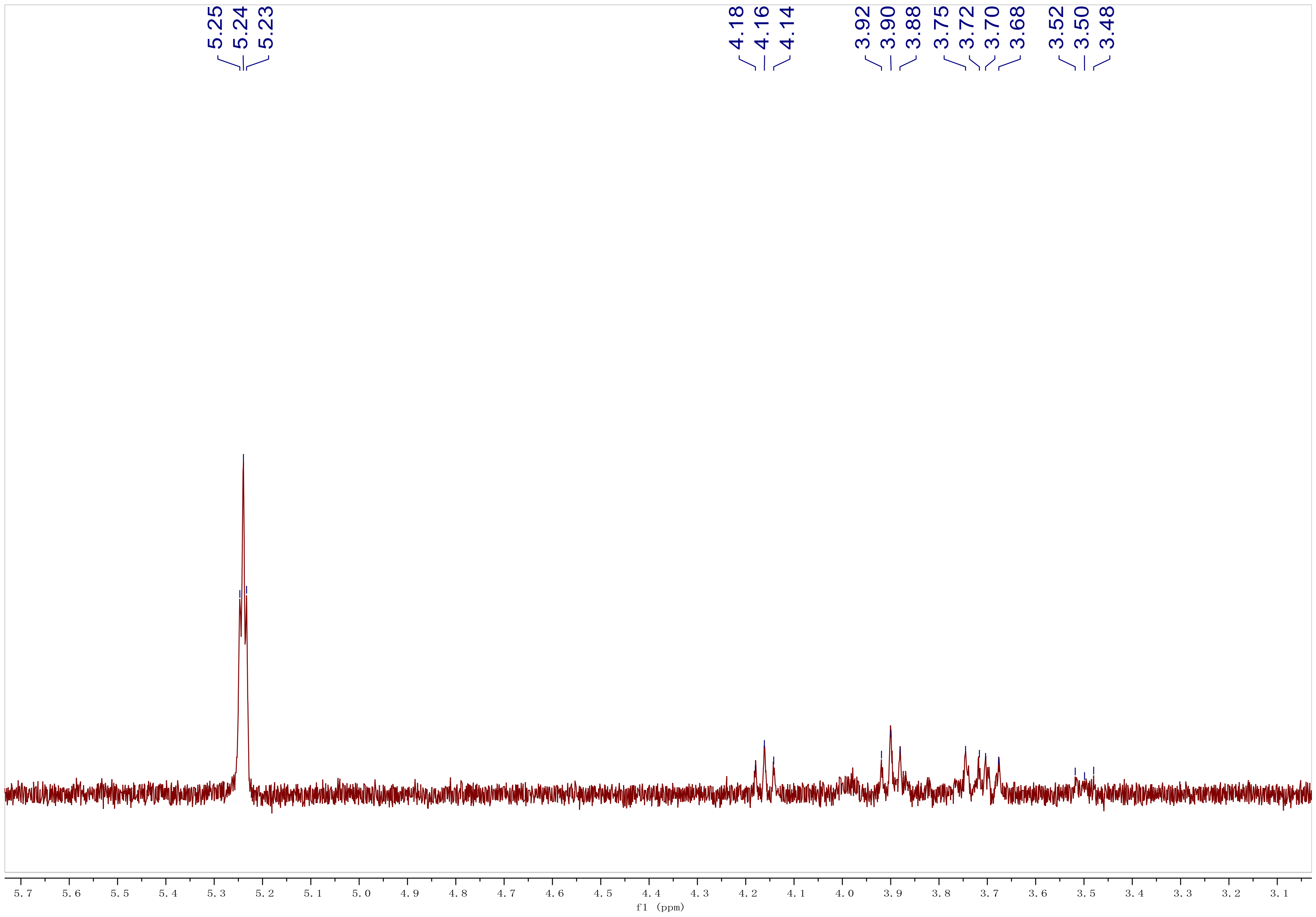

Figure S51. 1D-selective TOCSY spectrum of compound **12** (500 MHz, D_2O, excitation at δ 5.24, H-**A**1α).

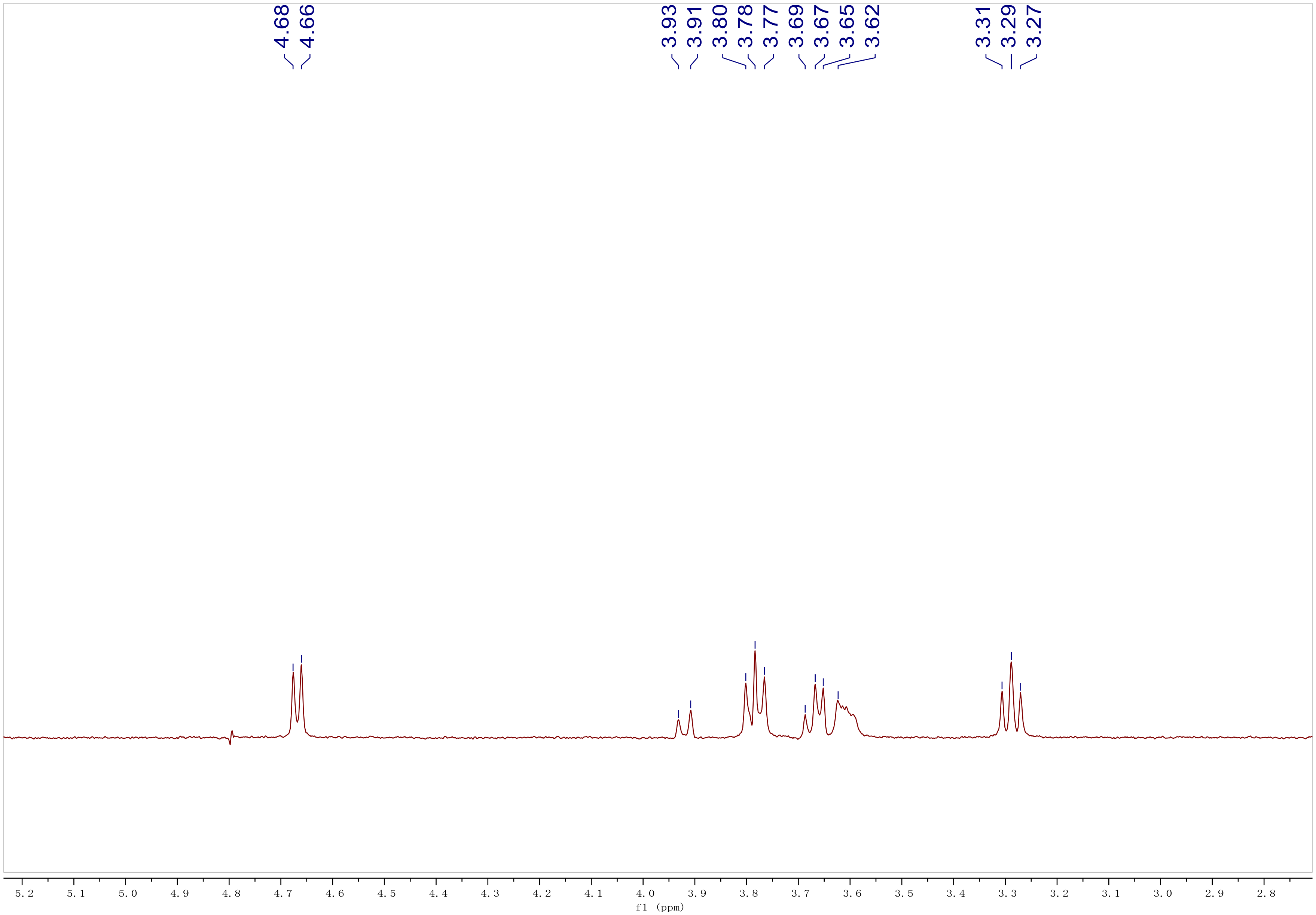

Figure S52. 1D-selective TOCSY spectrum of compound **12** (500 MHz, D_2O, excitation at δ 4.66, H-**A**1β).

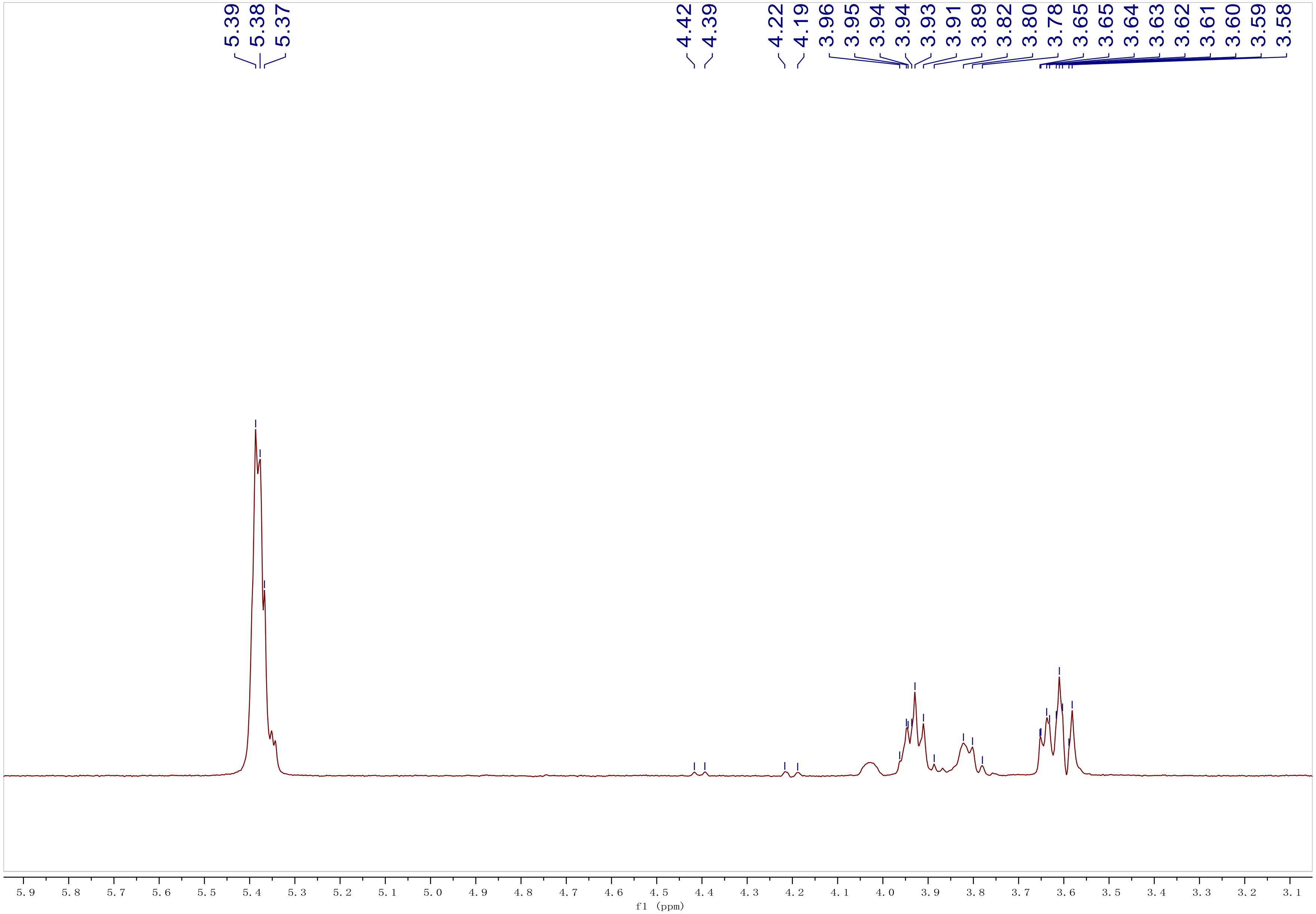

Figure S53. 1D-selective TOCSY spectrum of compound **12** (500 MHz, D_2O, excitation at δ 5.38, H-**B**1 and H-**C**1).

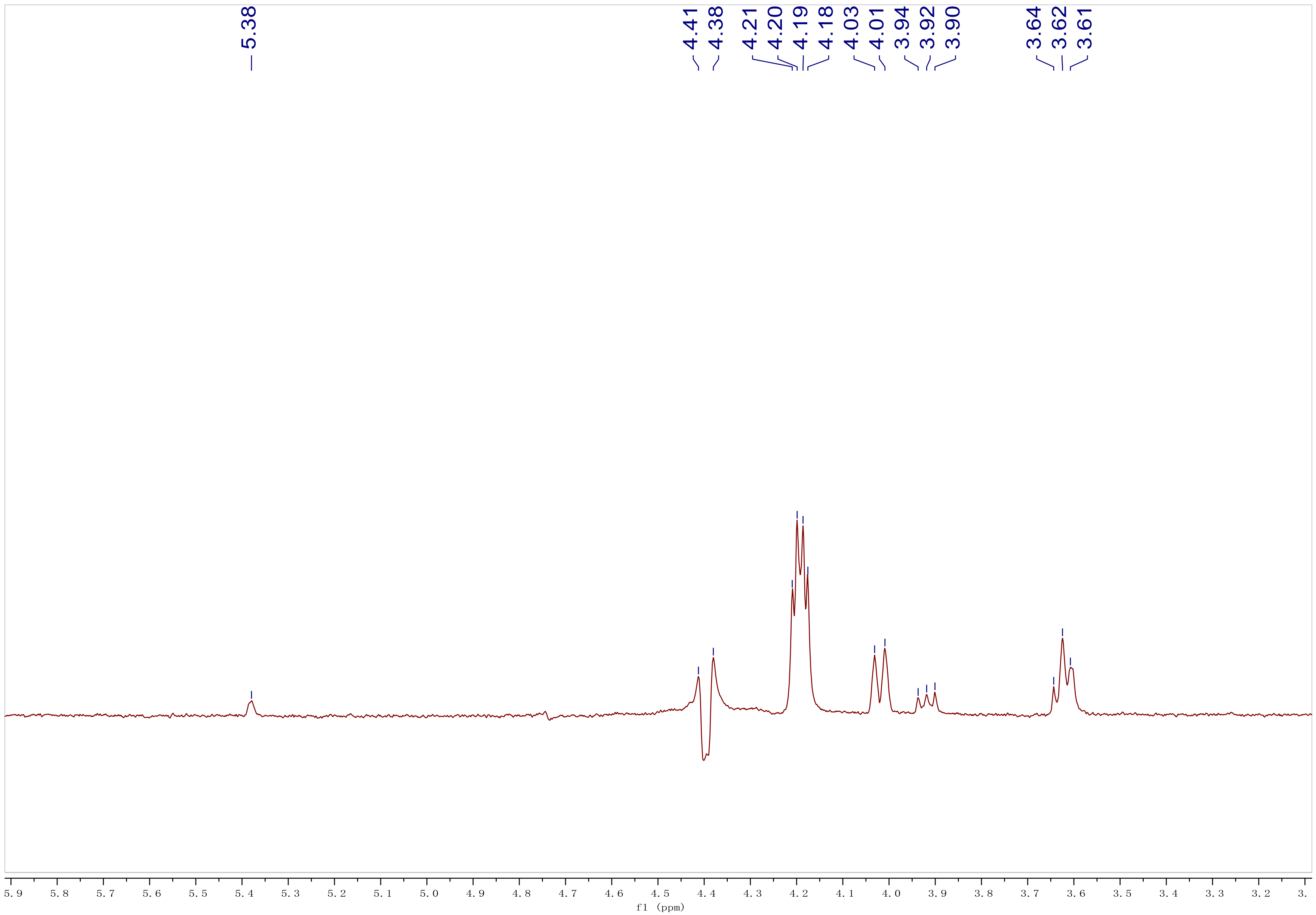

Figure S54. 1D-selective TOCSY spectrum of compound **12** (500 MHz, D_2O, excitation at δ 4.44, H-**C**6a).

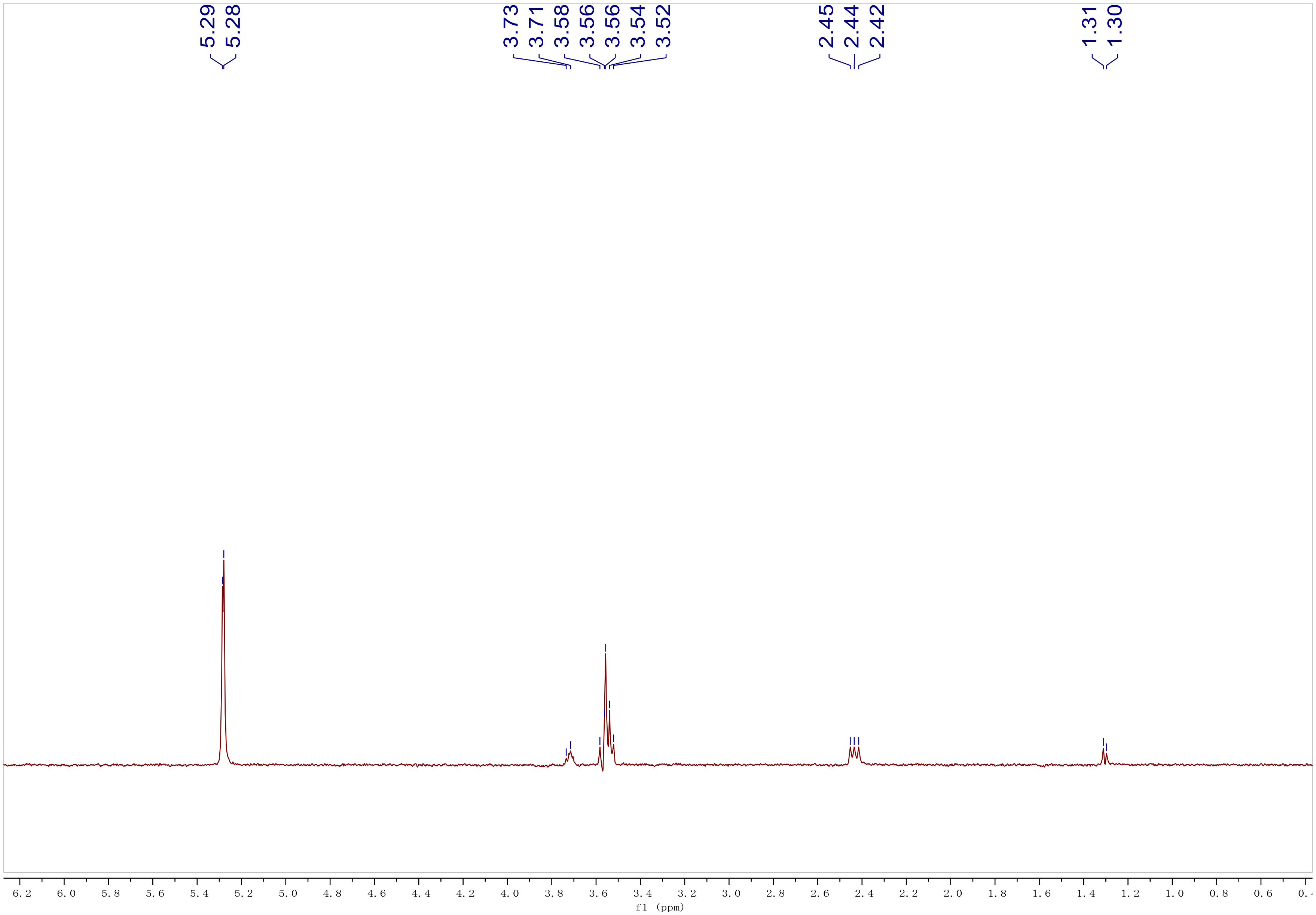

Figure S55. 1D-selective TOCSY spectrum of compound **12** (500 MHz, D_2O, excitation at δ 5.28, H-**D**1).

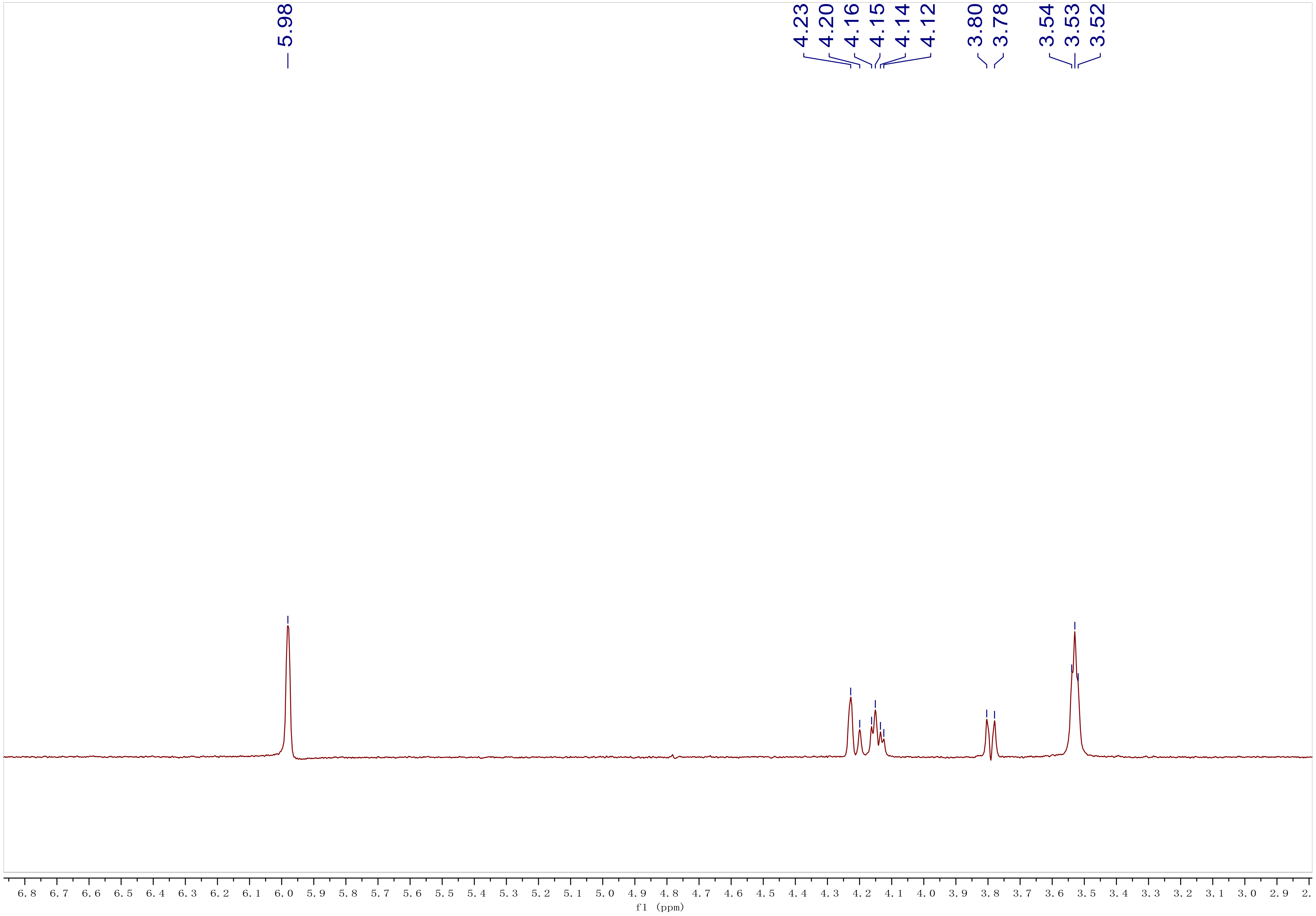

Figure S56. 1D-selective TOCSY spectrum of compound **12** (500 MHz, D_2O, excitation at δ 5.98, H-**E**1).

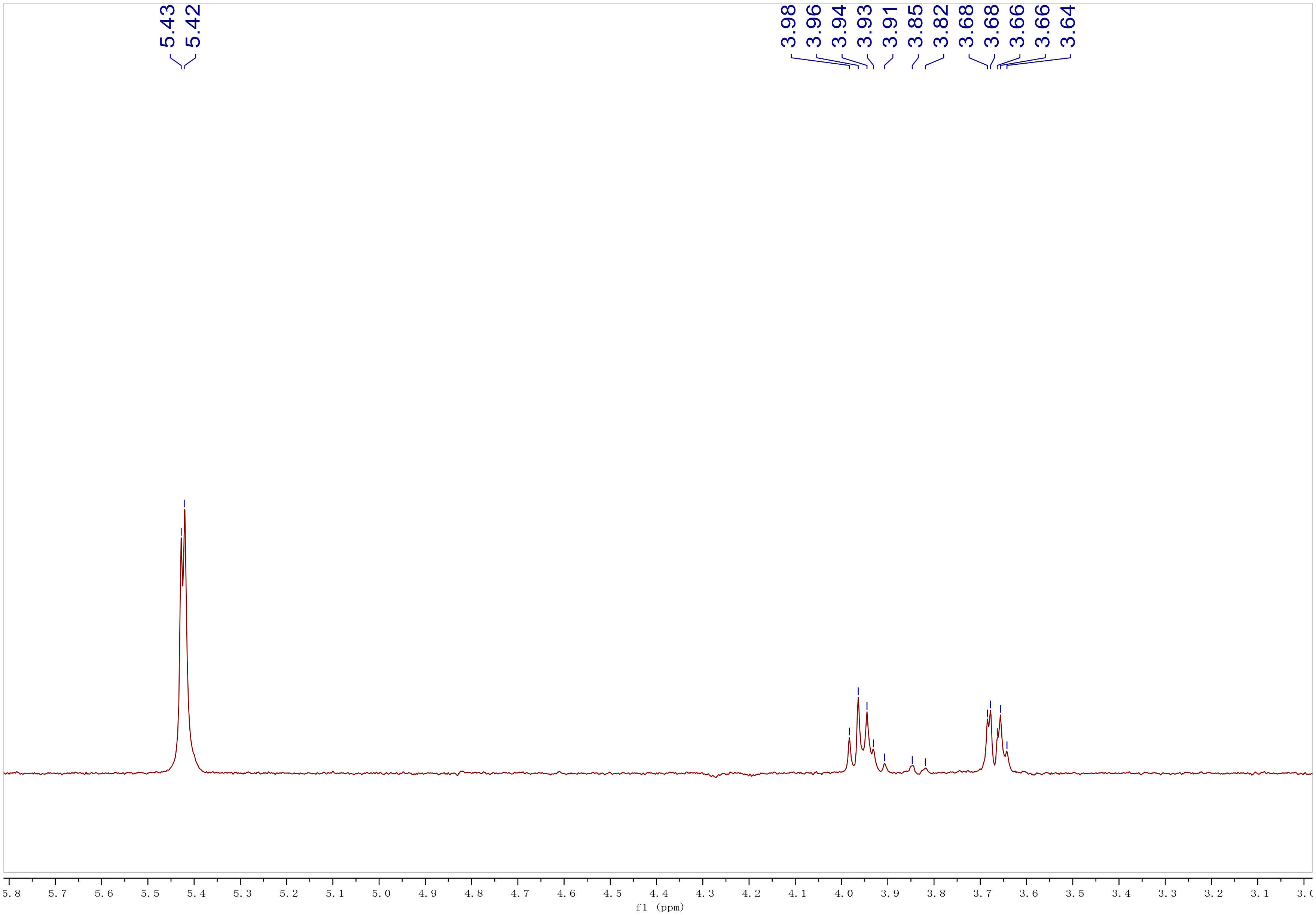

Figure S57. 1D-selective TOCSY spectrum of compound **12** (500 MHz, D_2O, excitation at δ 5.42, H-**F**1).

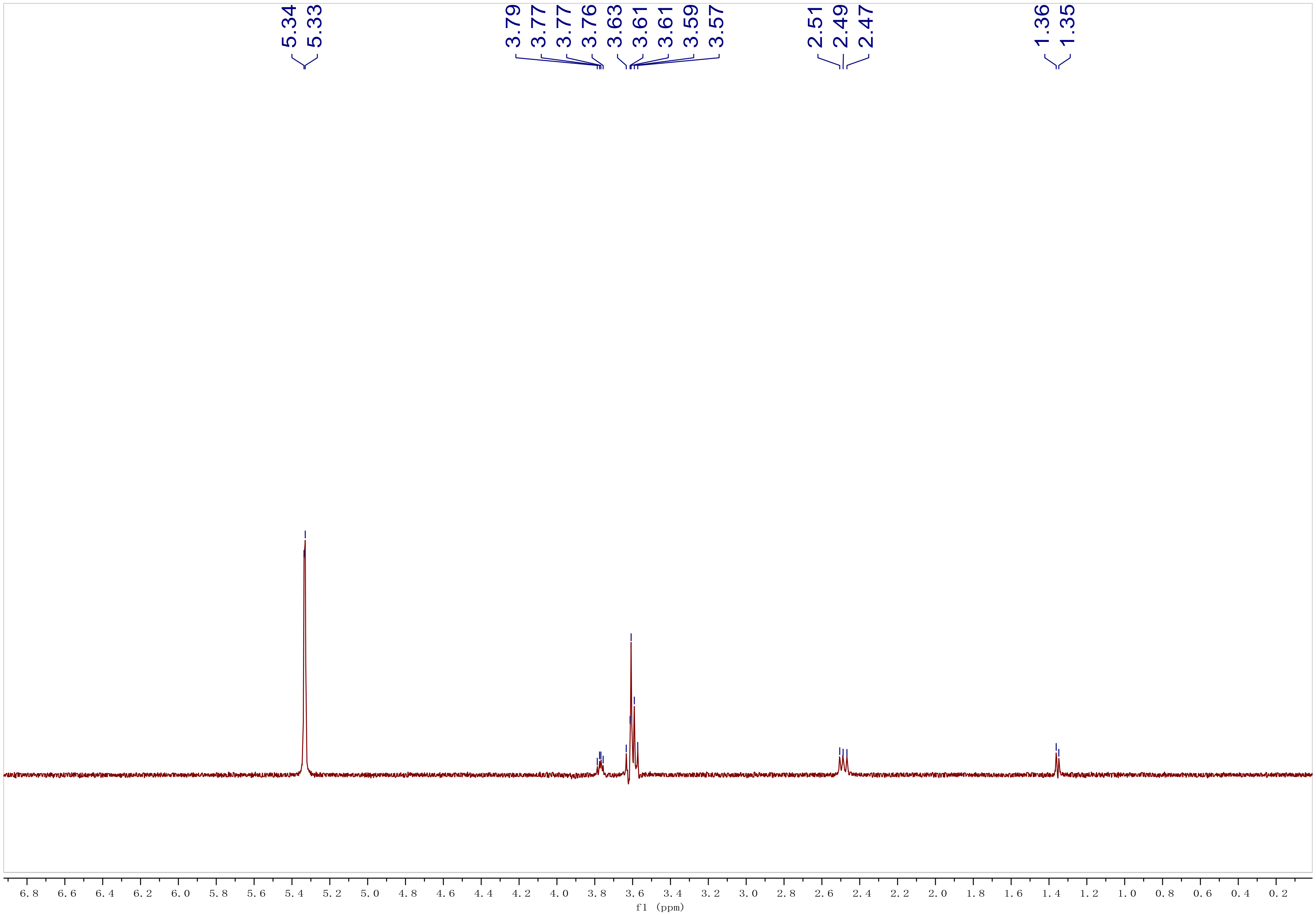

Figure S58. 1D-selective TOCSY spectrum of compound **12** (500 MHz, D_2O, excitation at δ 5.33, H-**G**1).

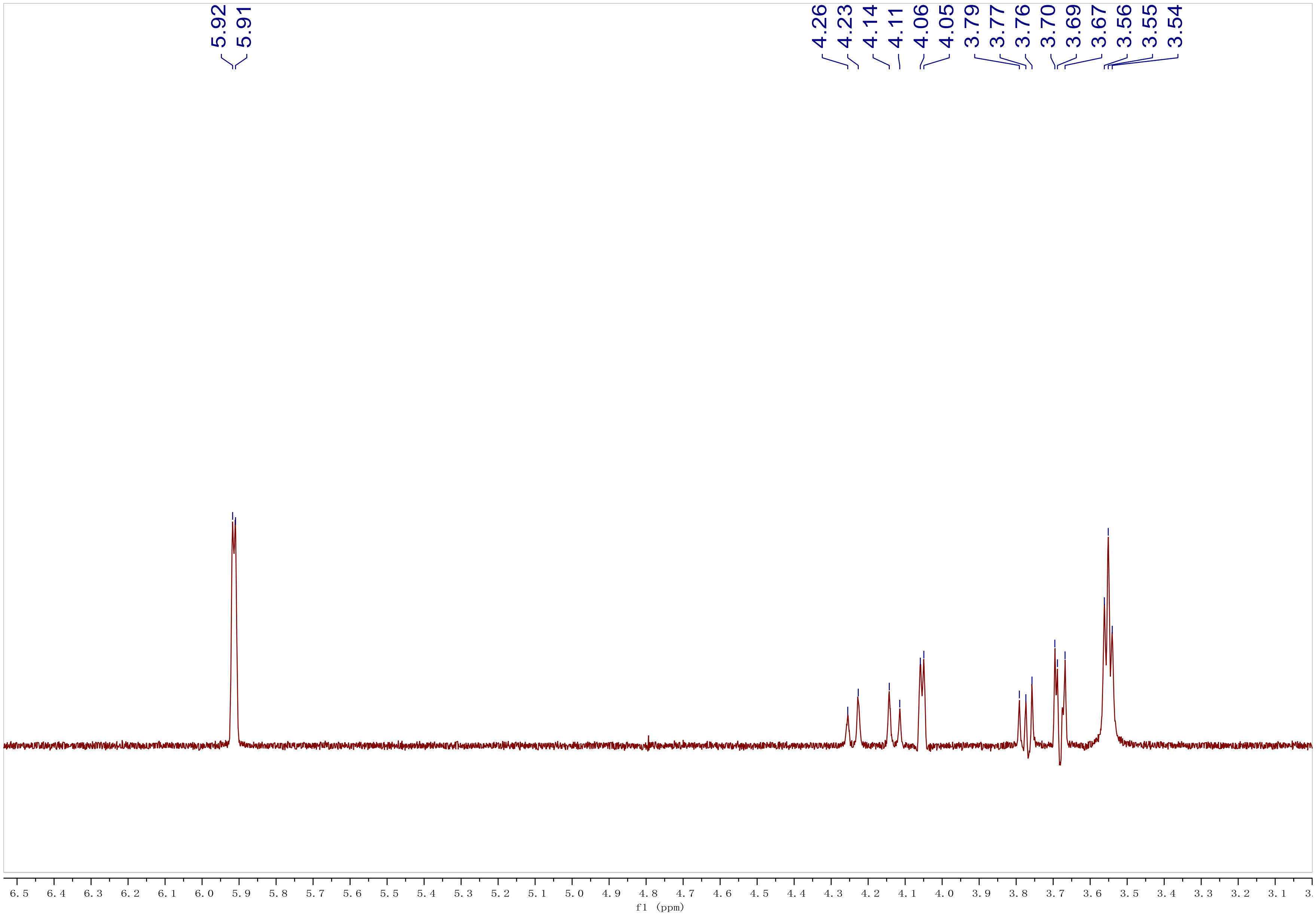

Figure S59. 1D-selective TOCSY spectrum of compound **12** (500 MHz, D_2O, excitation at δ 5.91, H-**H**1).

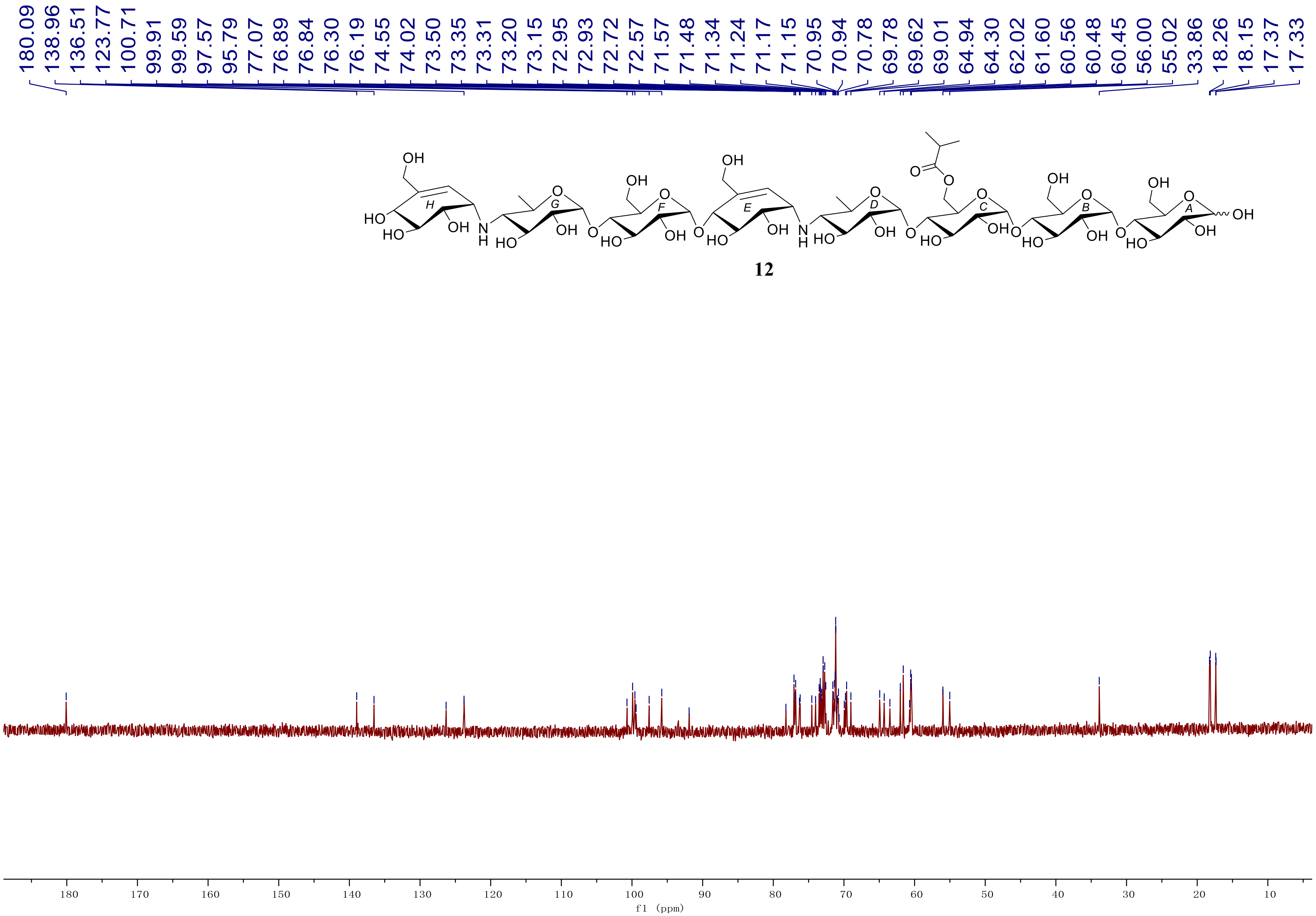

Figure S60. ^{13}C NMR spectrum of compound **12** (125 MHz, D_2O).

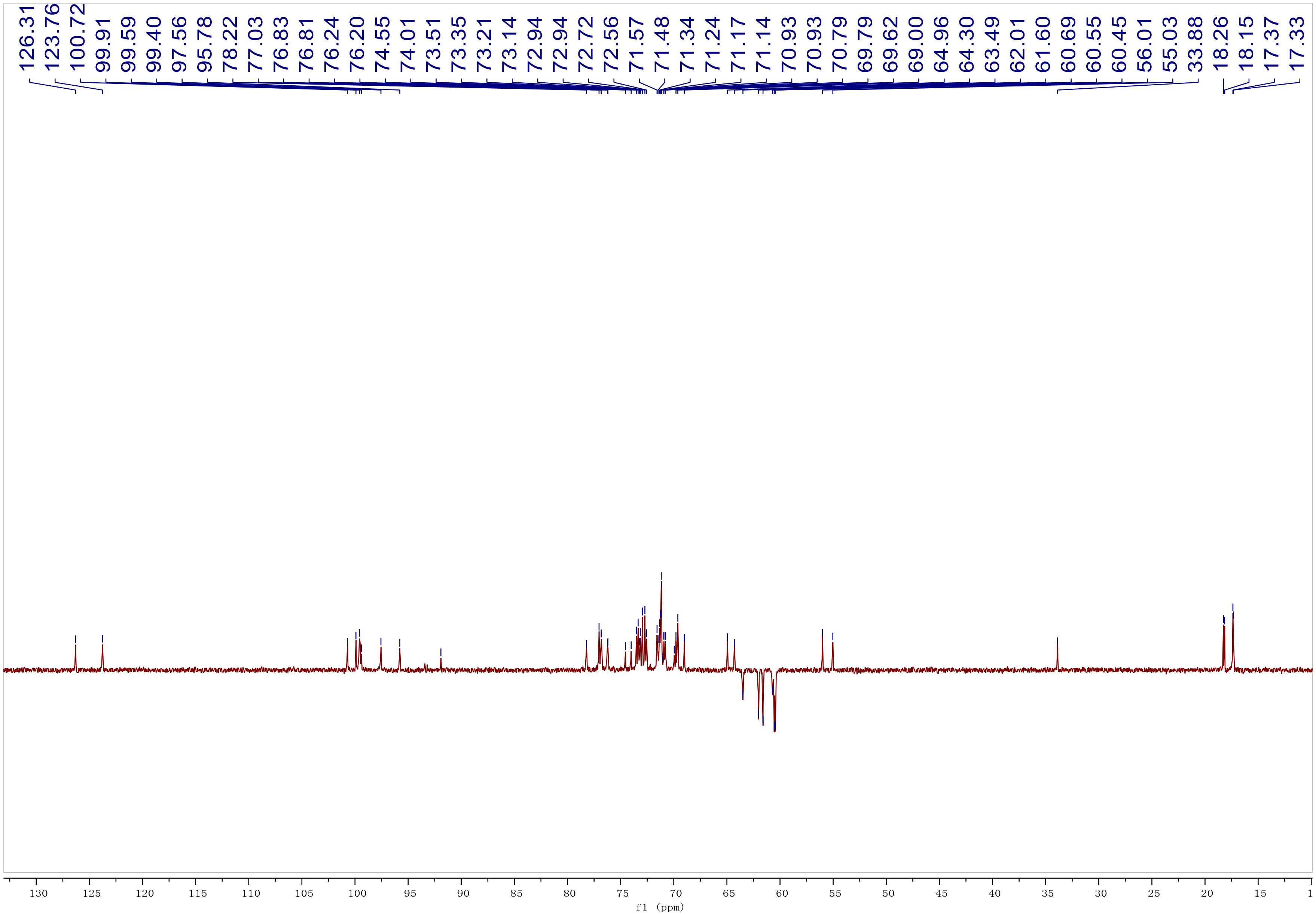

Figure S61. DEPT-135 spectrum of compound **12** (125 MHz, D_2O).

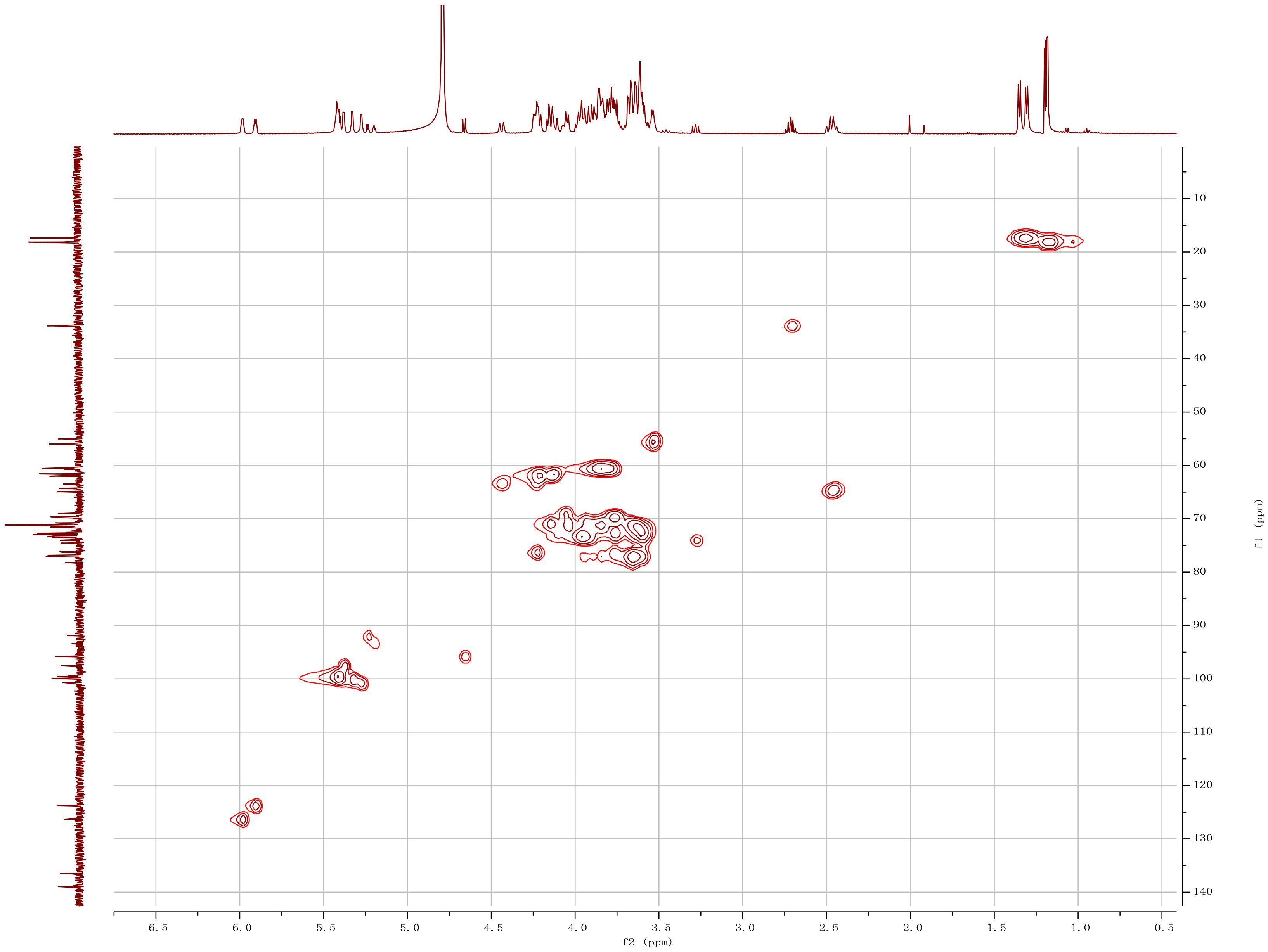

Figure S62. HSQC spectrum of compound **12** (500 MHz, D_2O).

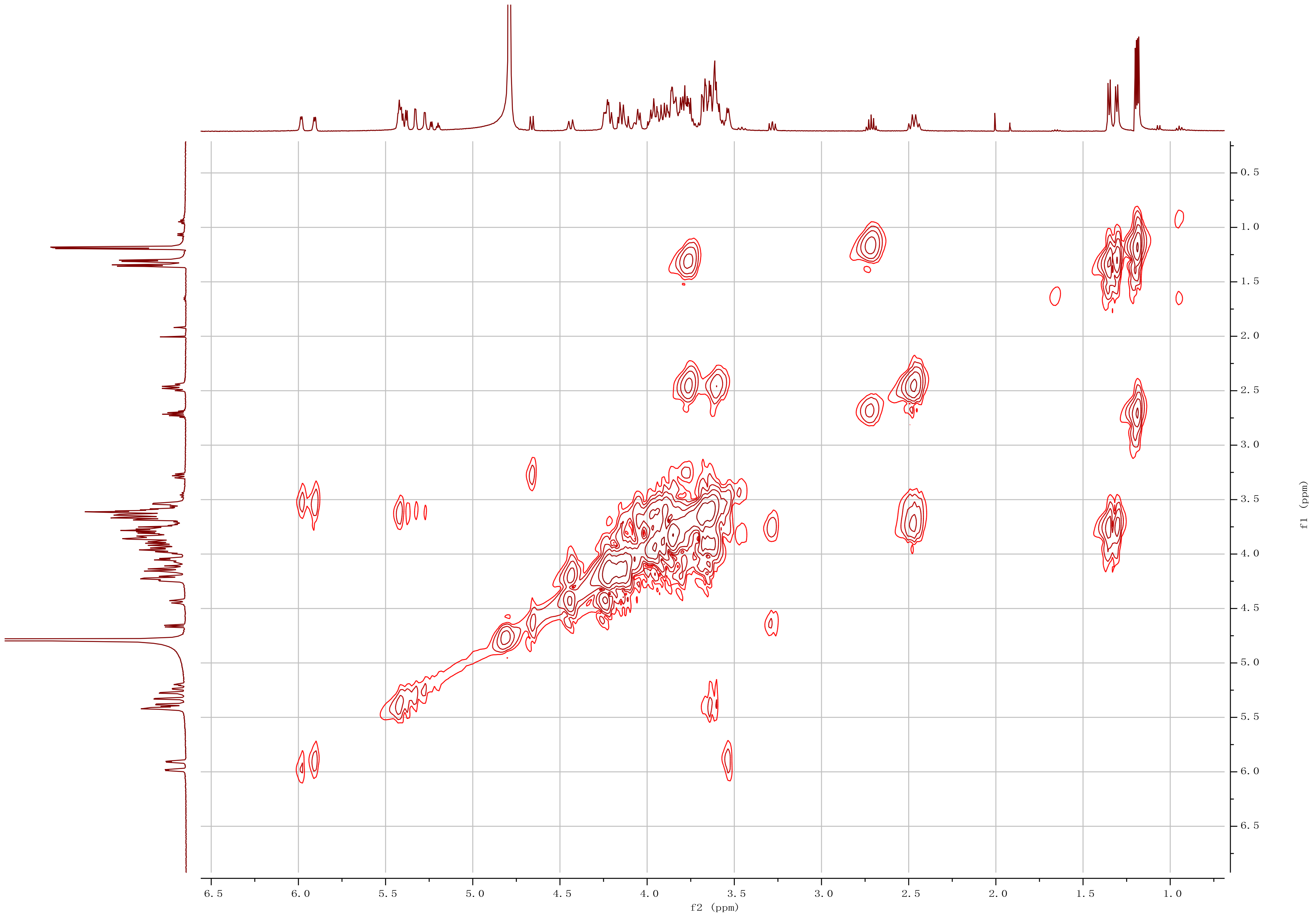

Figure S63. ${}^{1}H$-${}^{1}H$ COSY spectrum of compound **12** (500 MHz, D_2O).

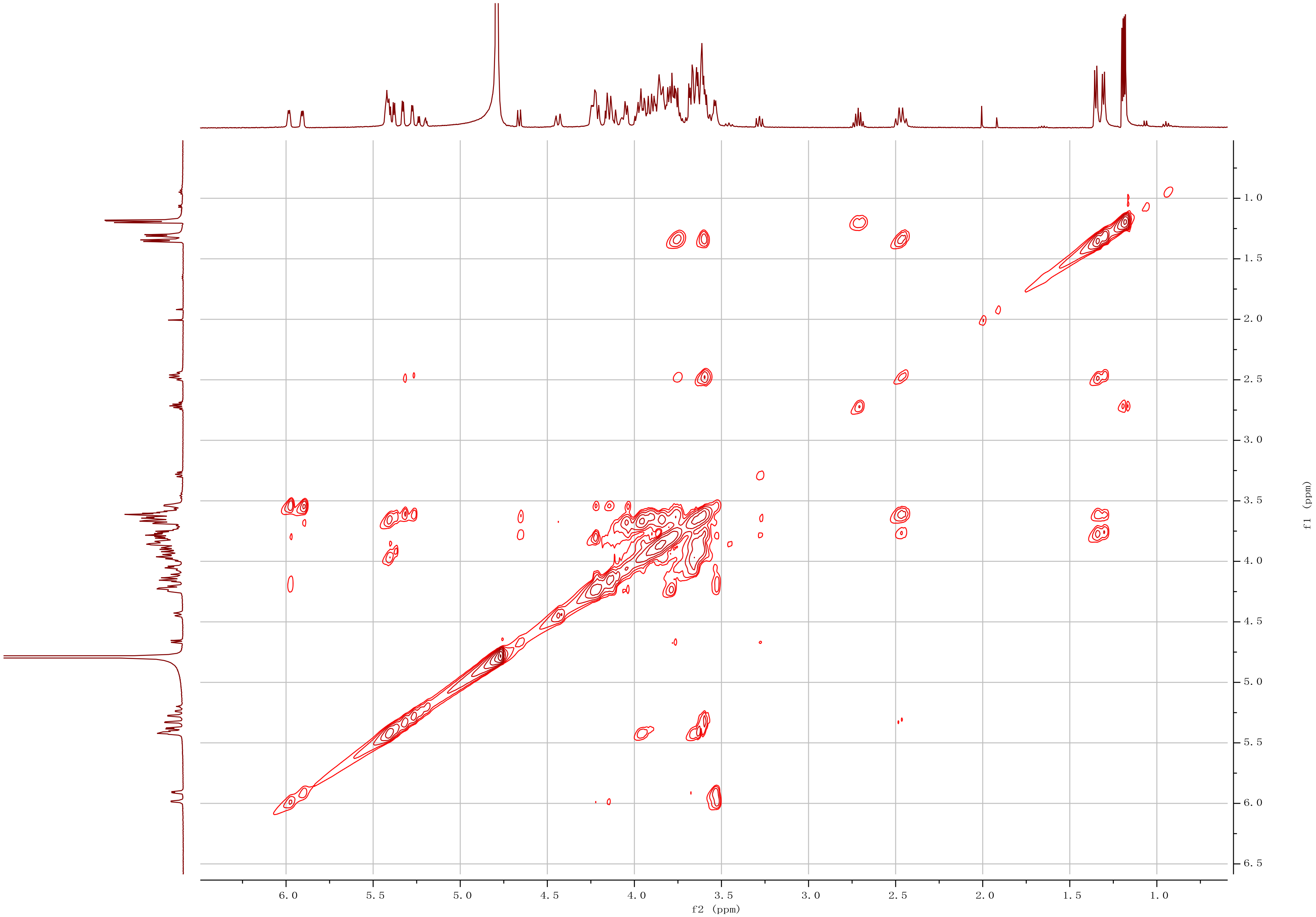

Figure S64. 2D-TOCSY spectrum of compound **12** (500 MHz, D_2O).

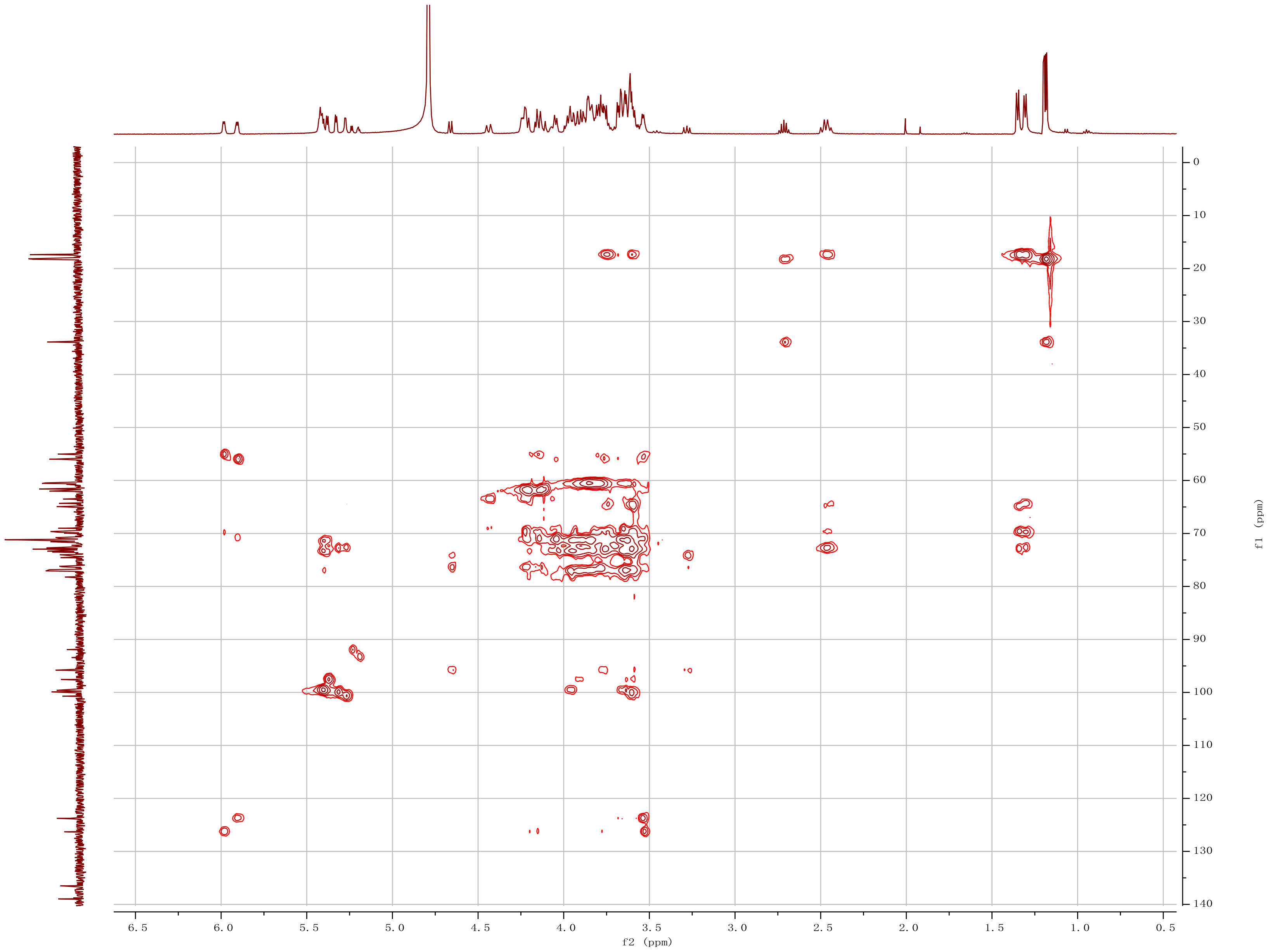

Figure S65. HSQC-TOCSY spectrum of compound **12** (500 MHz, D_2O).

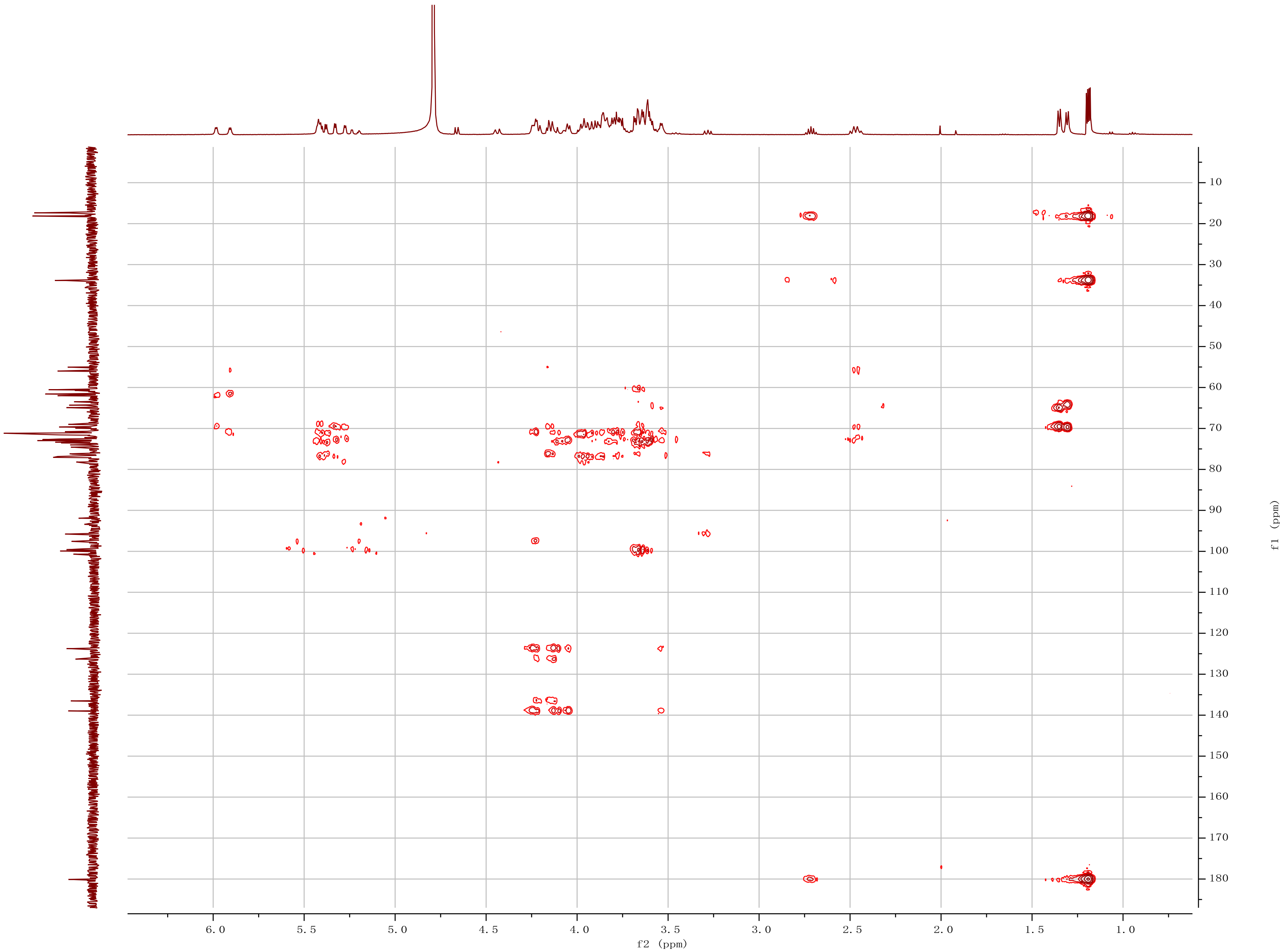

Figure S66. HMBC spectrum of compound **12** (500 MHz, D_2O).

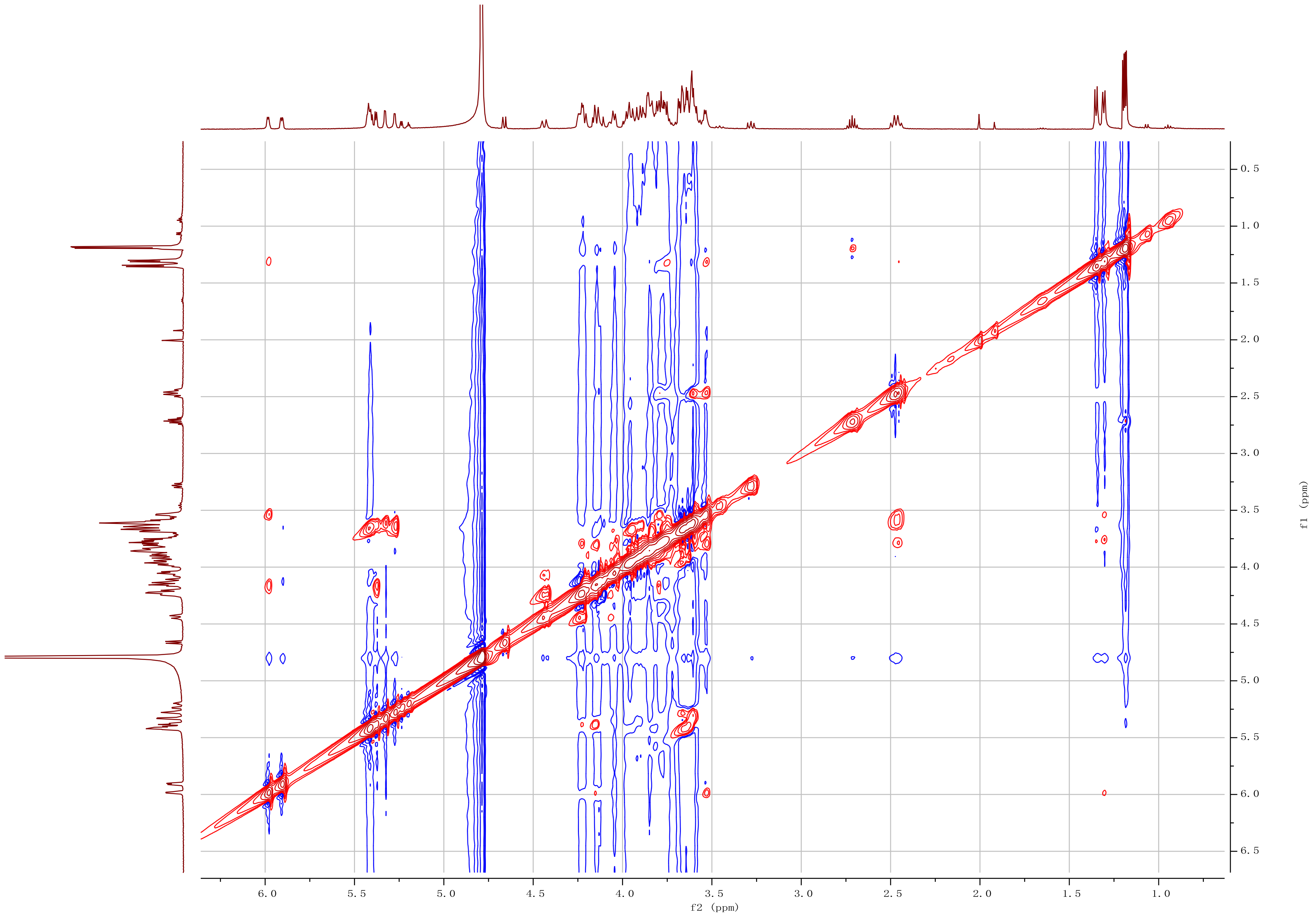

Figure S67. NOESY spectrum of compound **12** (500 MHz, D_2O).

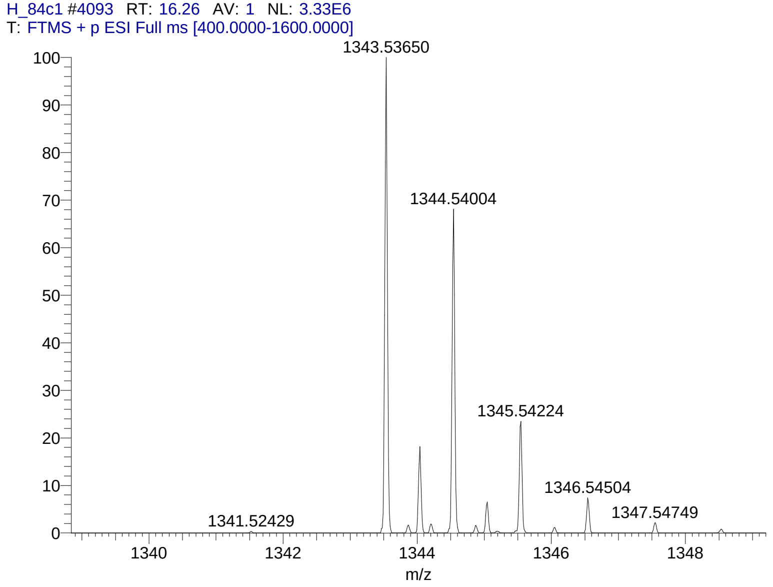

Figure S68. HRESIMS spectrum of compound **12**.

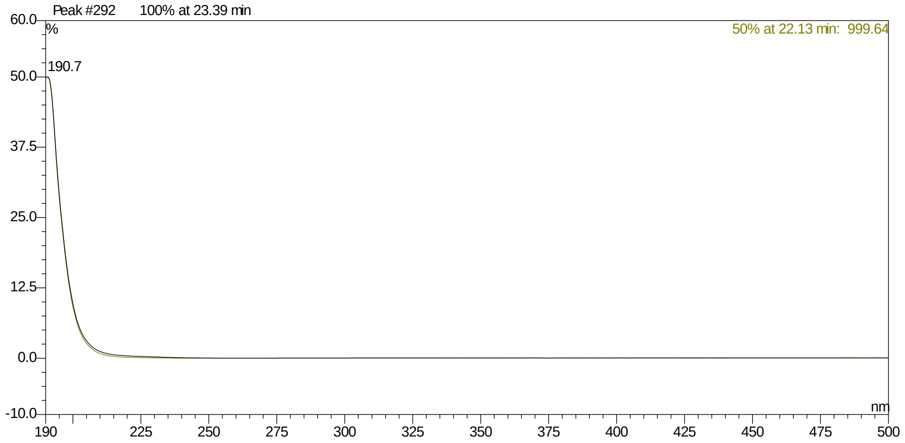

Figure S69. UV spectrum of compound **12**.

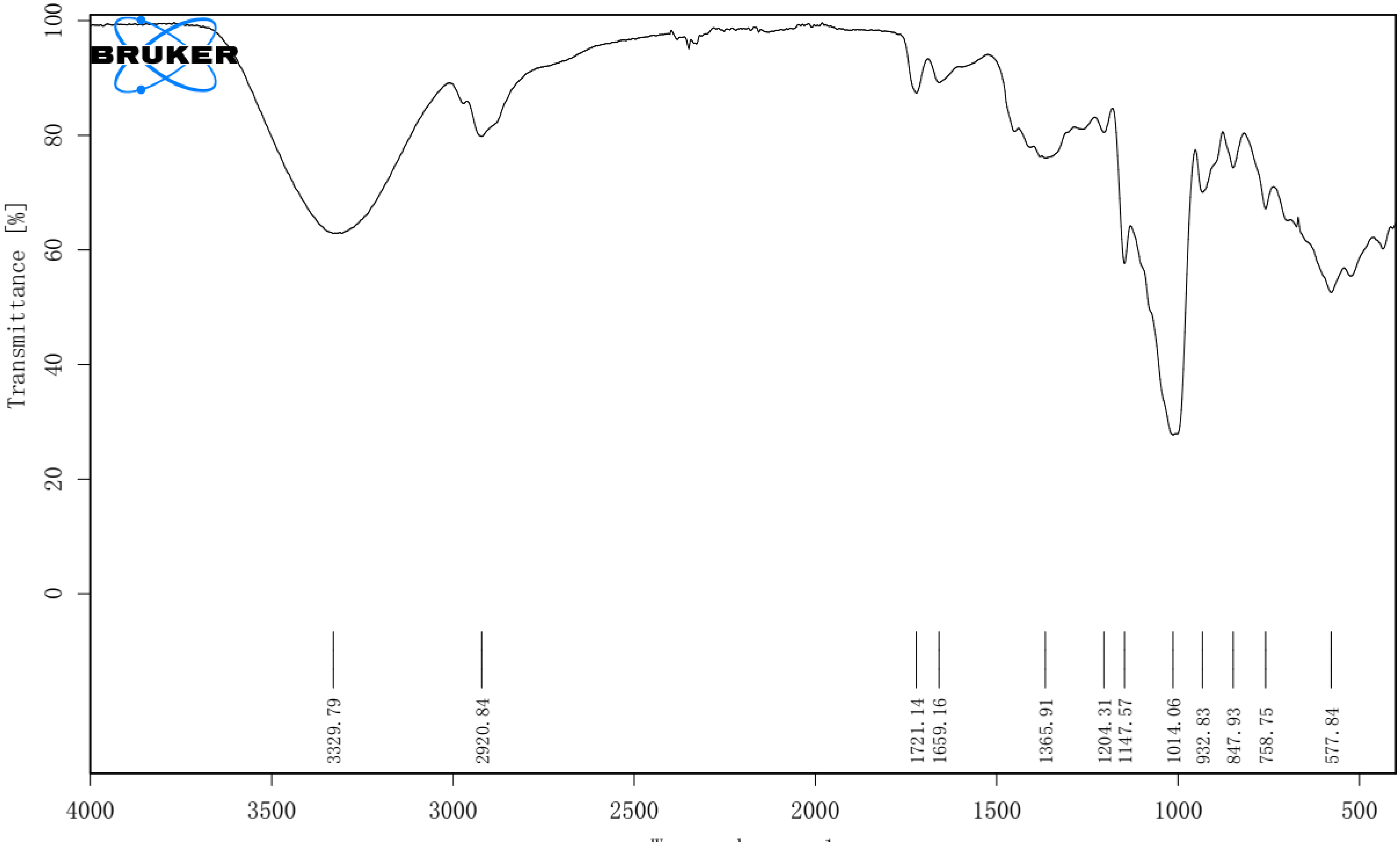

Figure S70. IR spectrum of compound **12**.

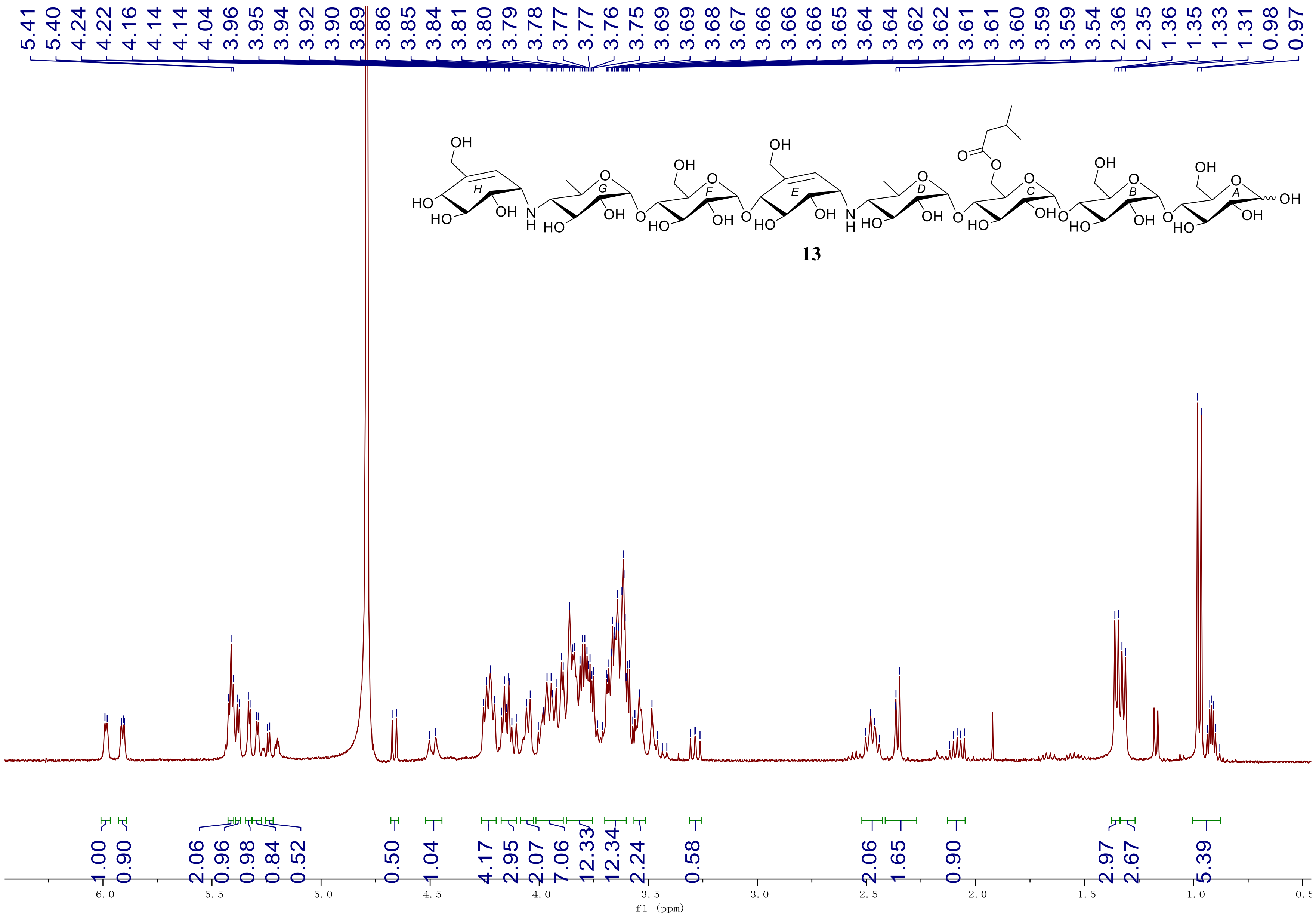

Figure S71. ^{1}H NMR spectrum of compound **13** (500 MHz, D_2O).

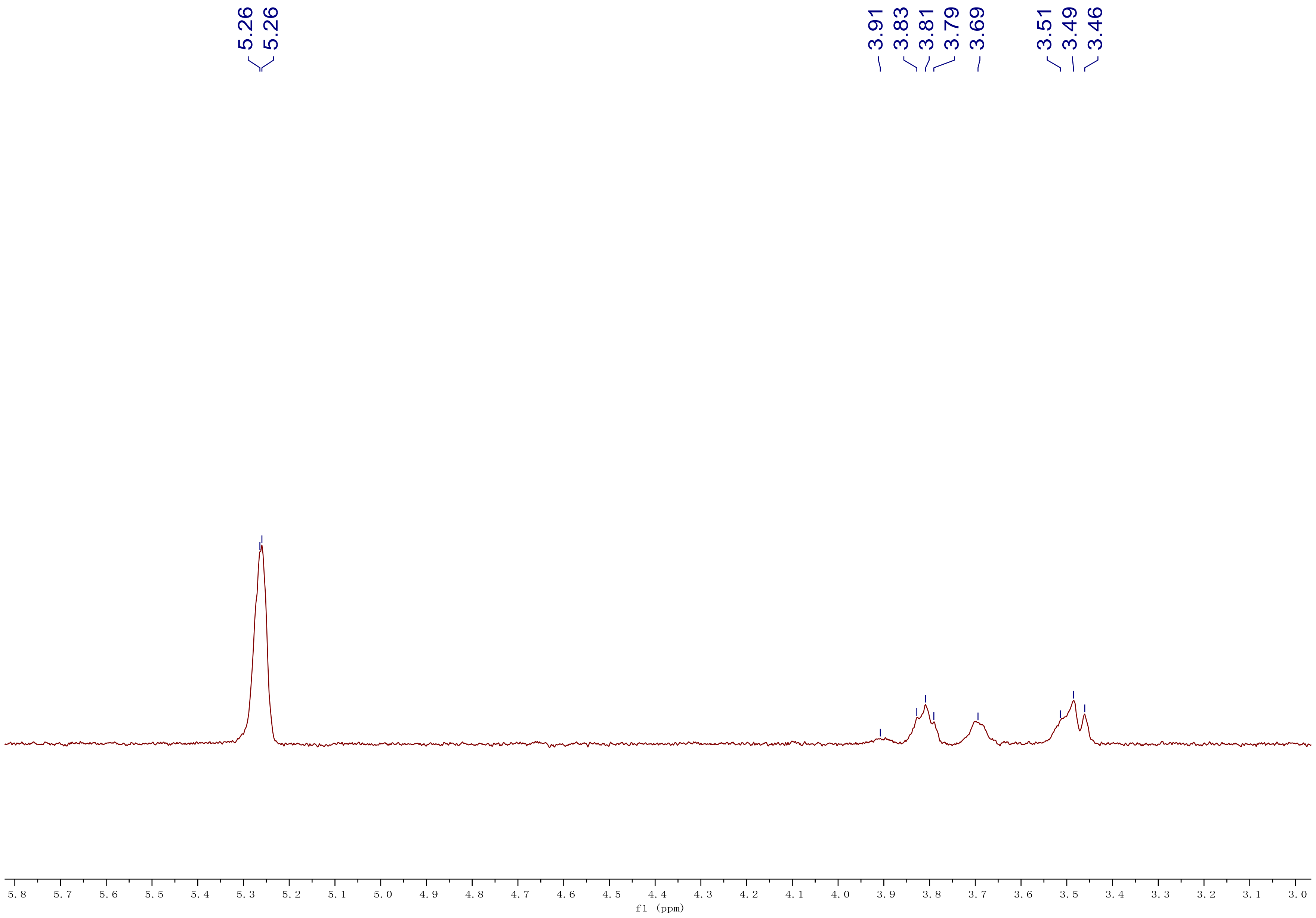

Figure S72. 1D-selective TOCSY spectrum of compound **13** (500 MHz, D_2O, excitation at δ 5.24, H-**A**1α).

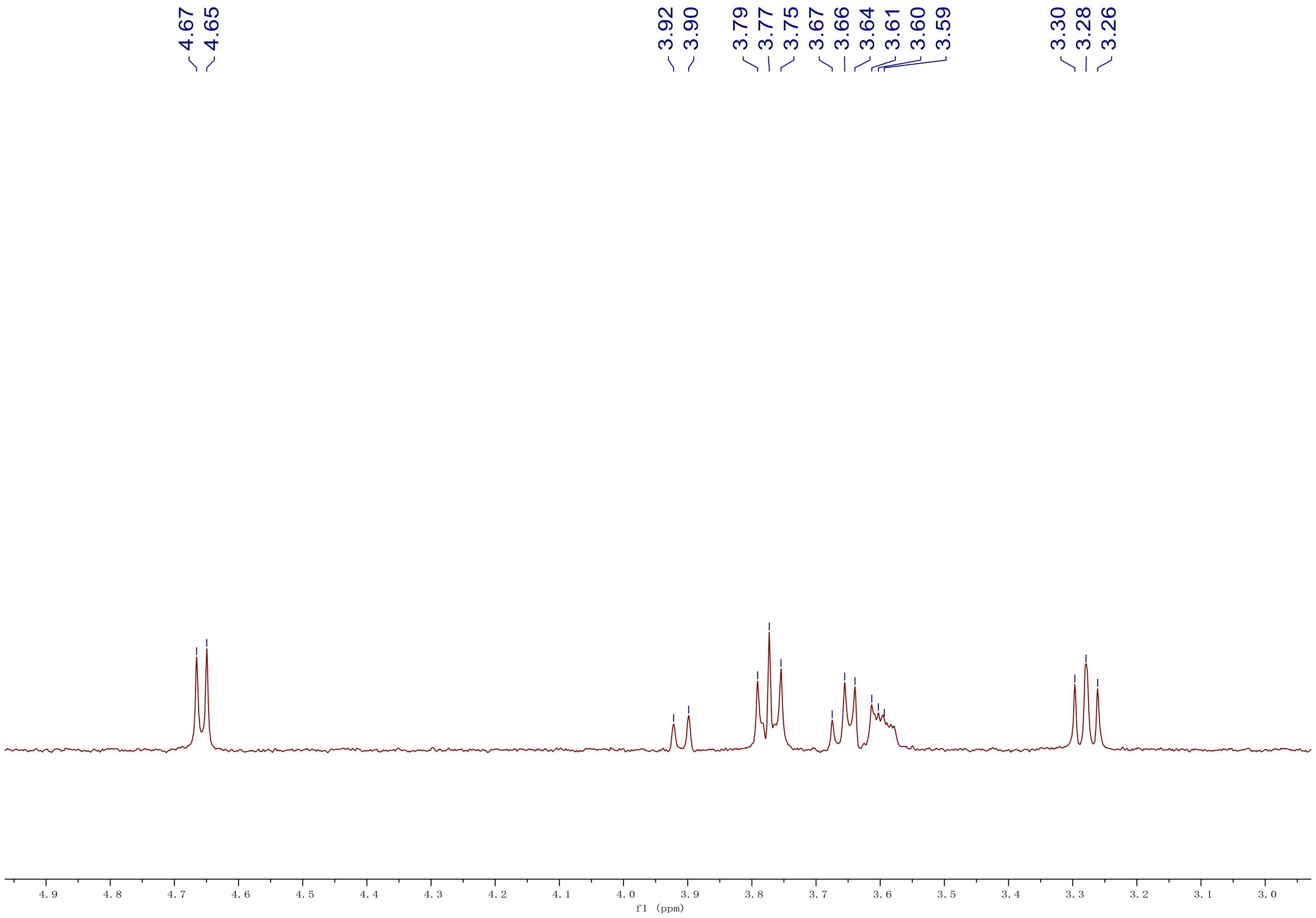

Figure S73. 1D-selective TOCSY spectrum of compound **13** (500 MHz, D_2O, excitation at δ 4.66, H-**A**1β).

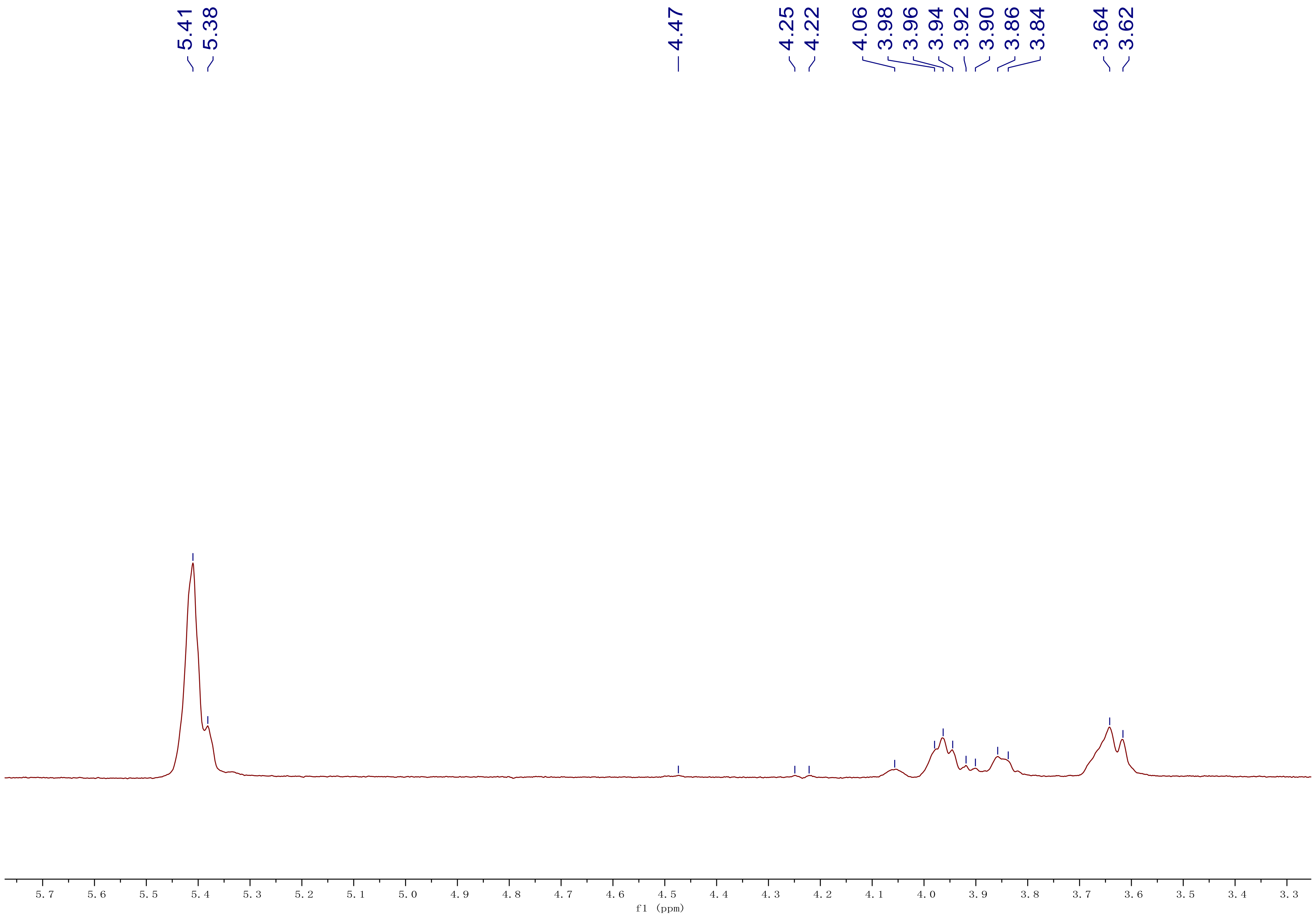

Figure S74. 1D-selective TOCSY spectrum of compound **13** (500 MHz, D_2O, excitation at δ 5.41, H-**B**1 and H-**C**1).

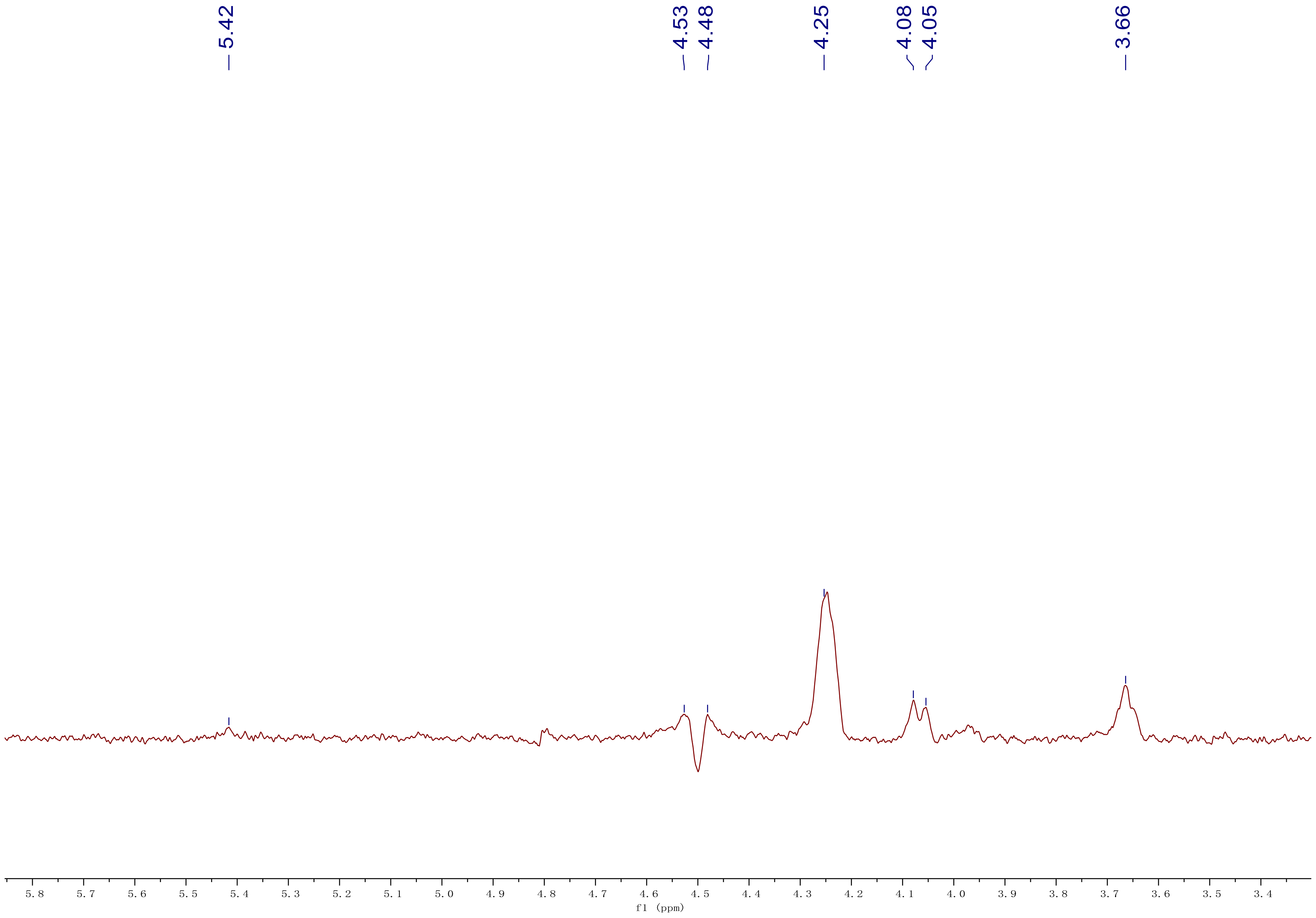

Figure S75. 1D-selective TOCSY spectrum of compound **13** (500 MHz, D_2O, excitation at δ 4.49, H-**C**6).

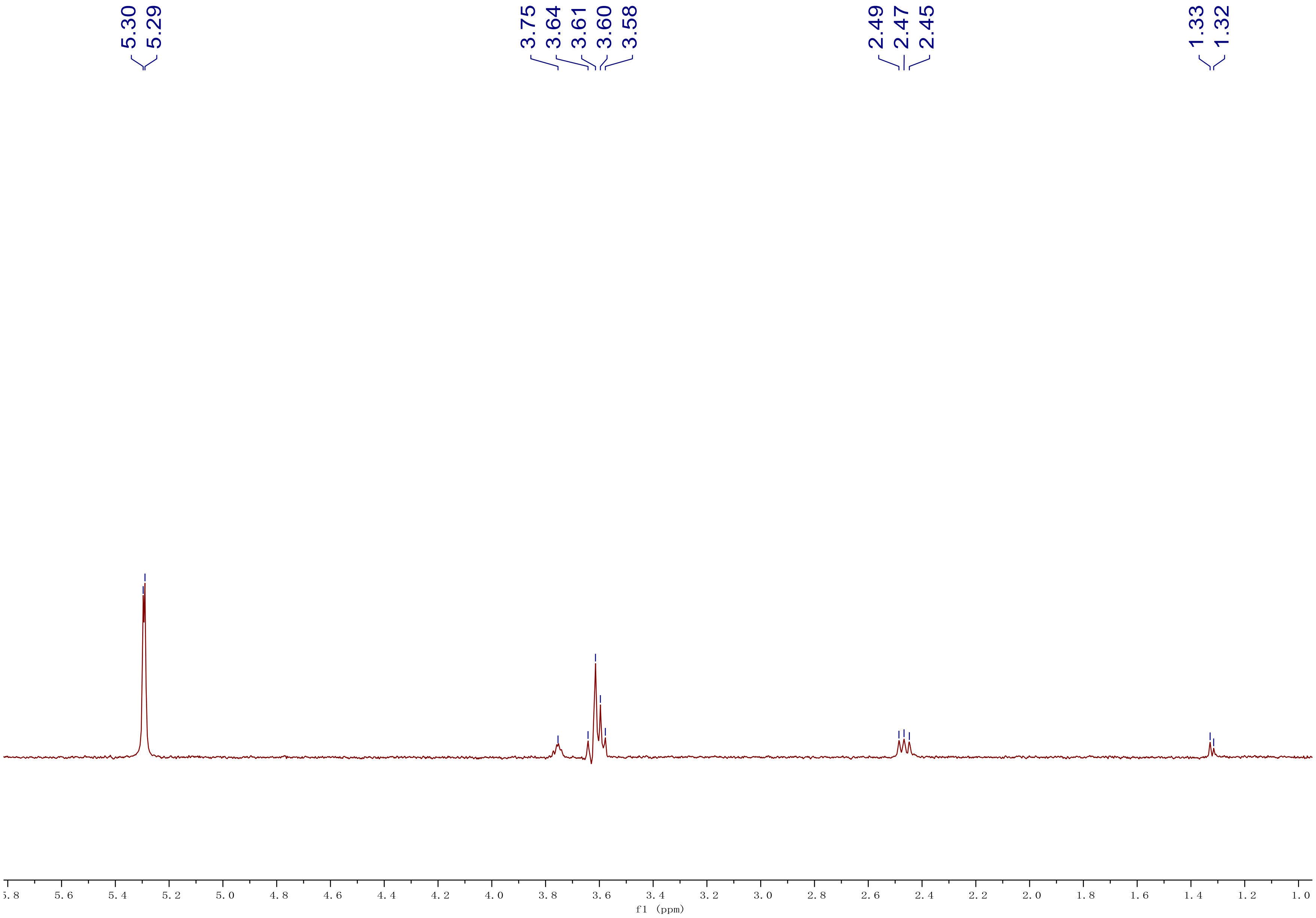

Figure S76. 1D-selective TOCSY spectrum of compound **13** (500 MHz, D_2O, excitation at δ 5.29, H-**D**1).

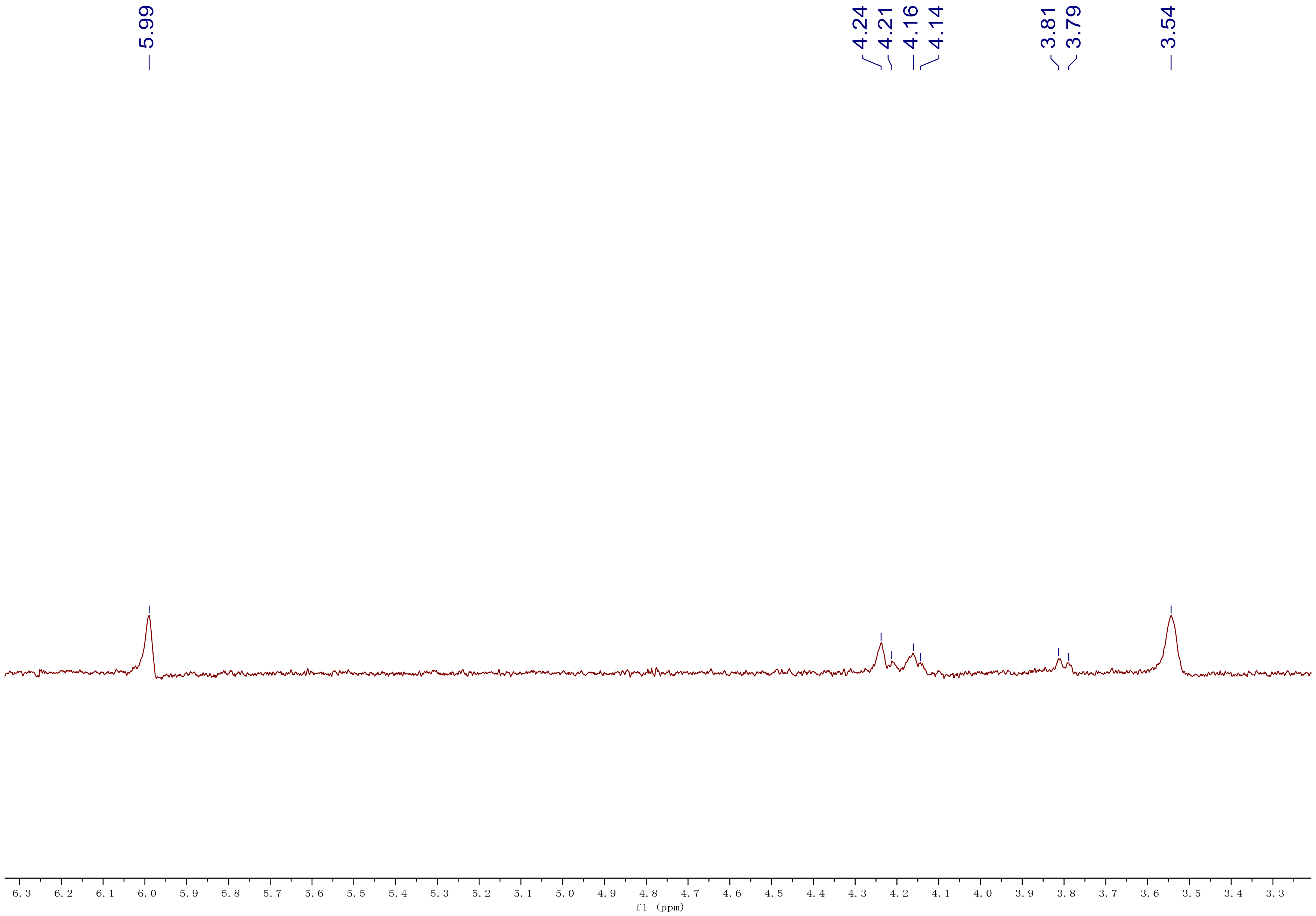

Figure S77. 1D-selective TOCSY spectrum of compound **13** (500 MHz, D_2O, excitation at δ 5.99, H-**E**7).

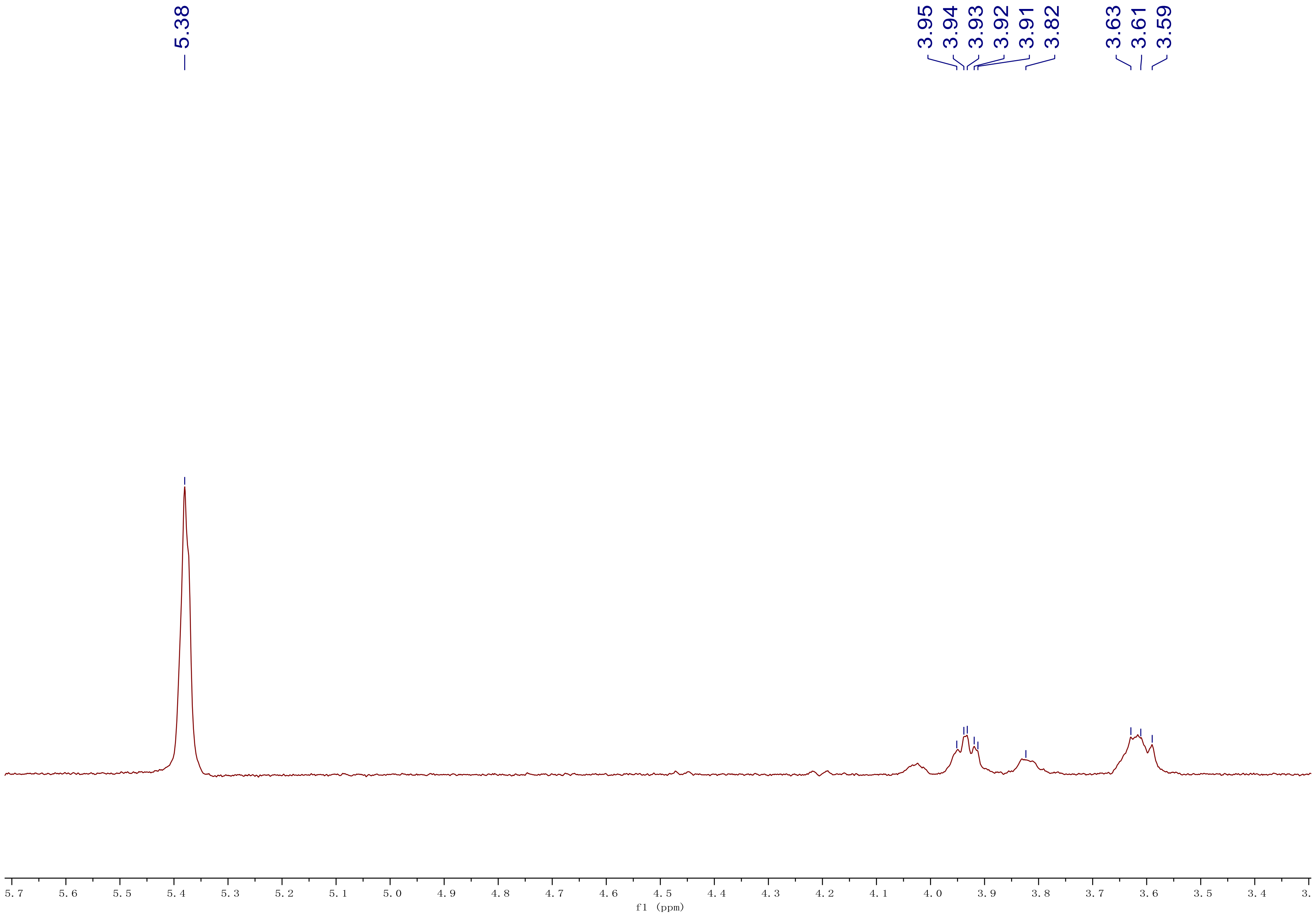

Figure S78. 1D-selective TOCSY spectrum of compound **13** (500 MHz, D_2O, excitation at δ 5.38, H-**F**1).

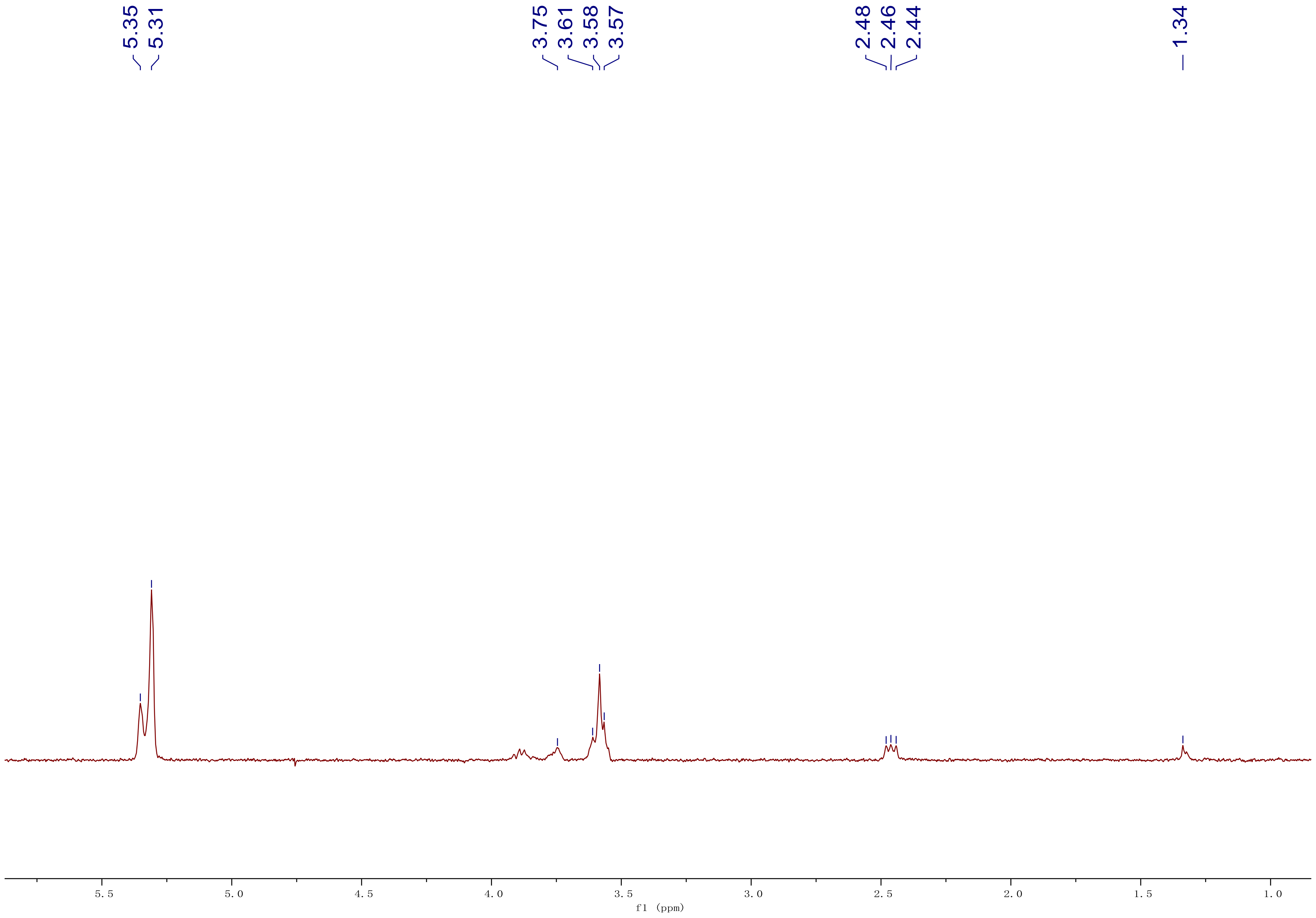

Figure S79. 1D-selective TOCSY spectrum of compound **13** (500 MHz, D_2O, excitation at δ 5.33, H-**G**1).

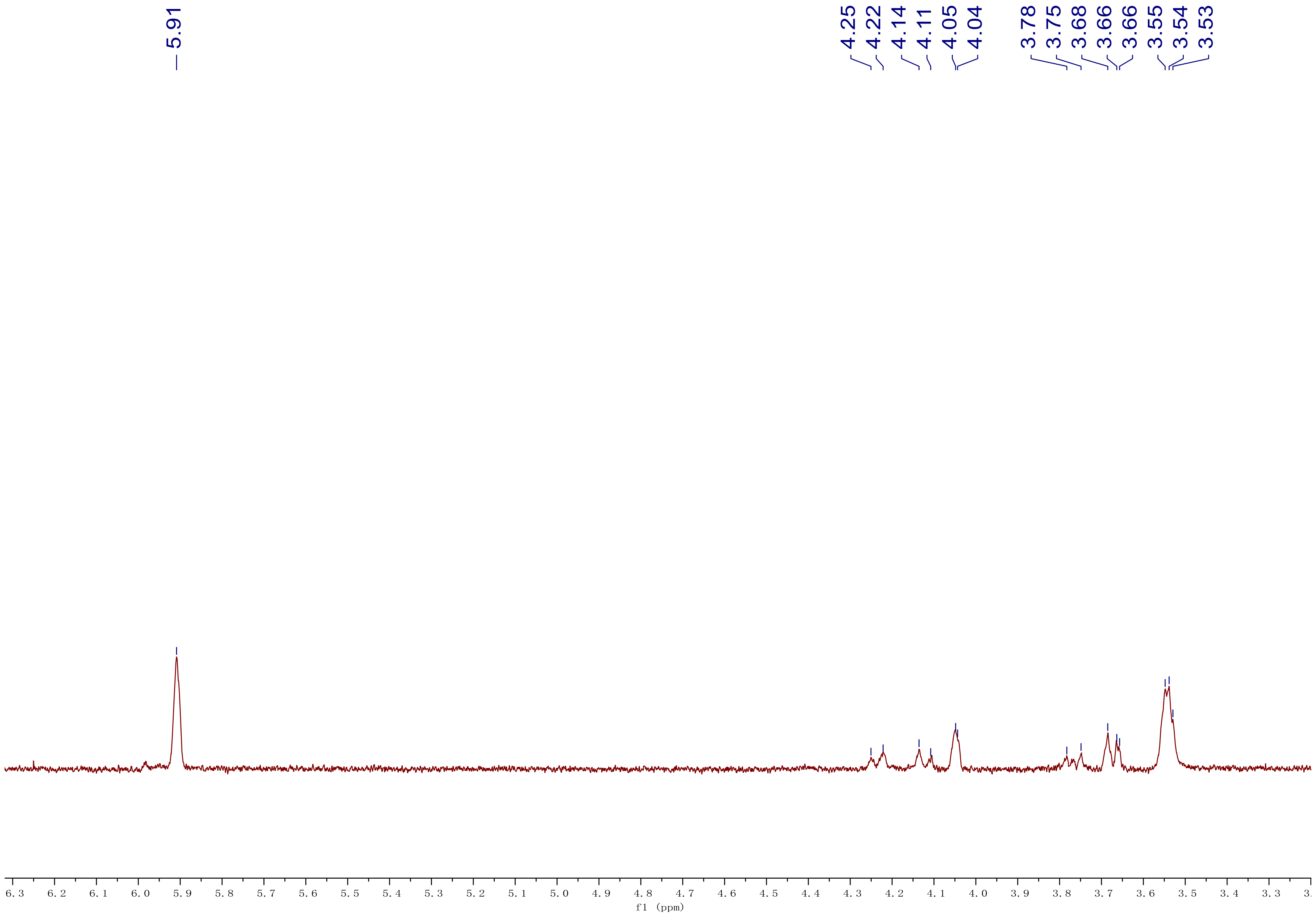

Figure S80. 1D-selective TOCSY spectrum of compound **13** (500 MHz, D_2O, excitation at δ 5.91, H-**H**7).

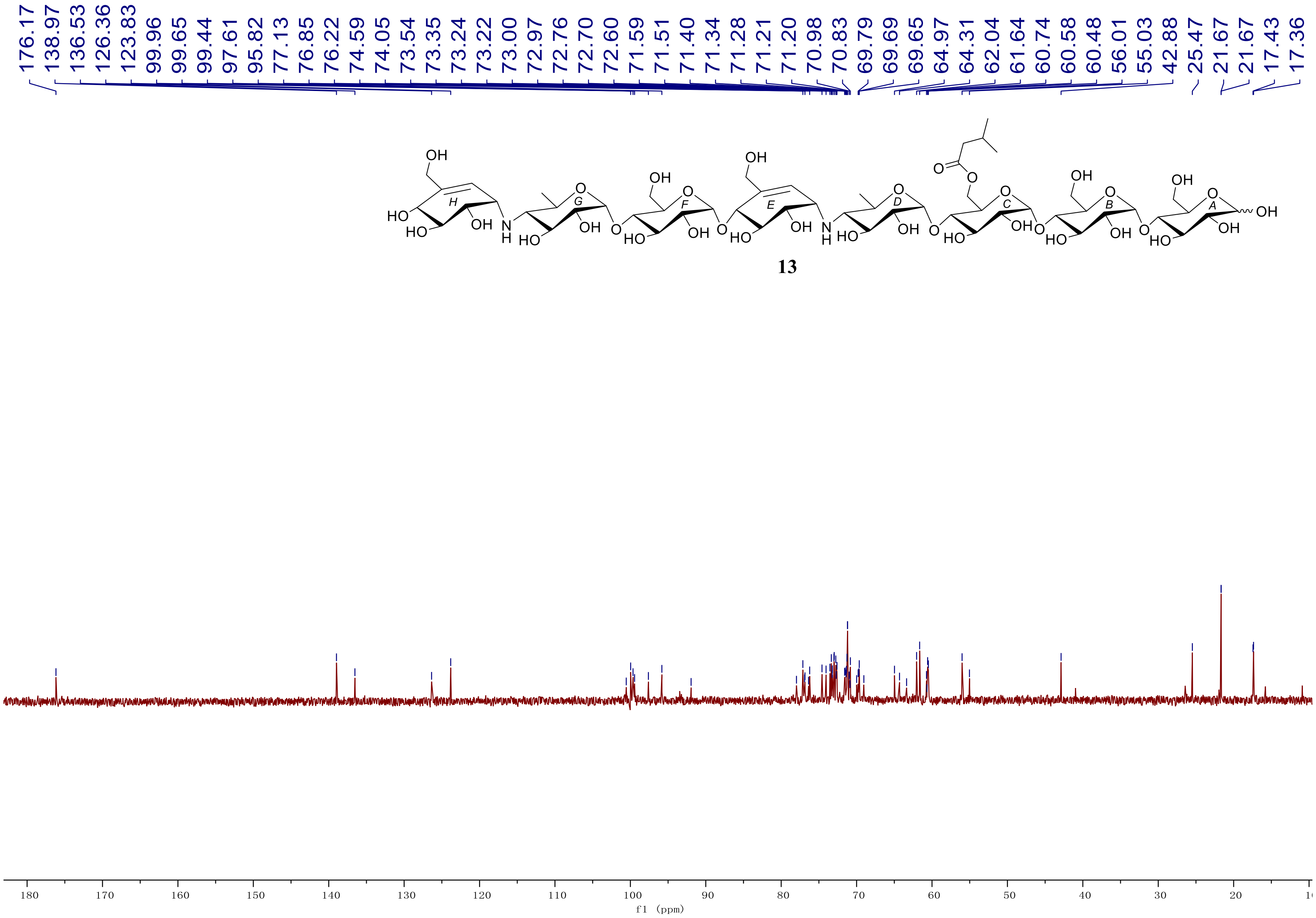

Figure S81. ^{13}C NMR spectrum of compound **13** (125 MHz, D_2O).

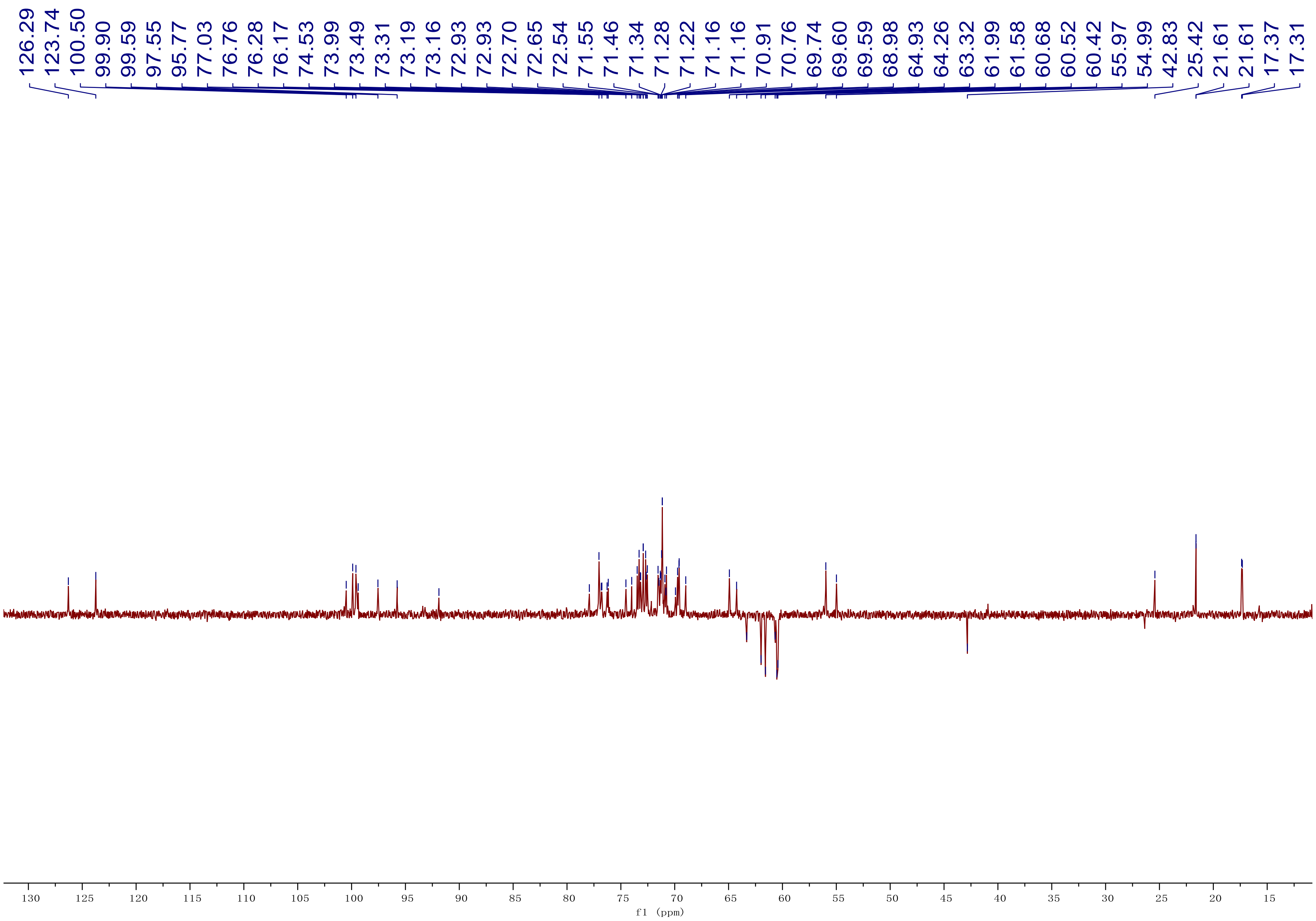

Figure S82. DEPT-135 spectrum of compound **13** (125 MHz, D_2O).

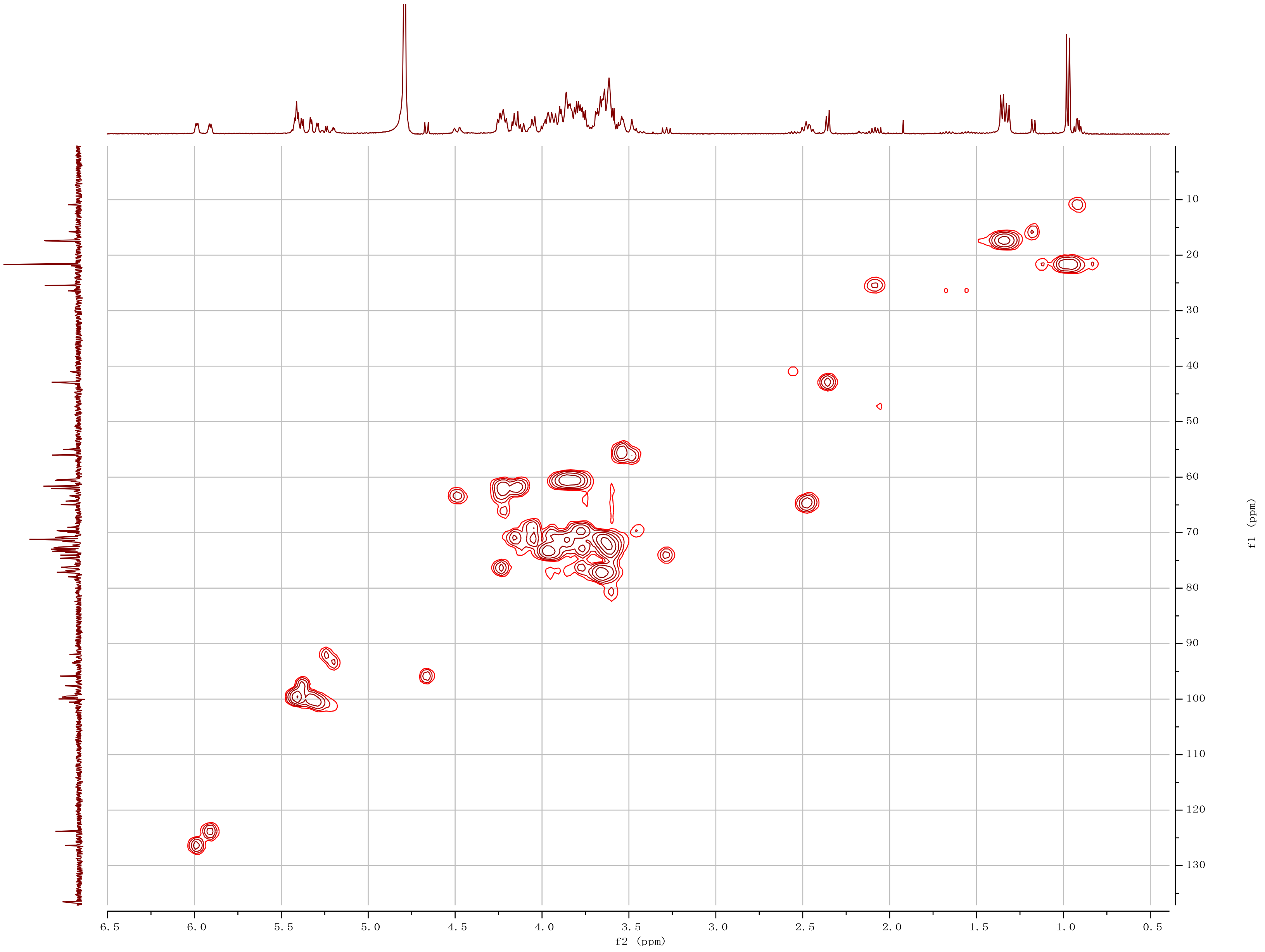

Figure S83. HSQC spectrum of compound **13** (500 MHz, D_2O).

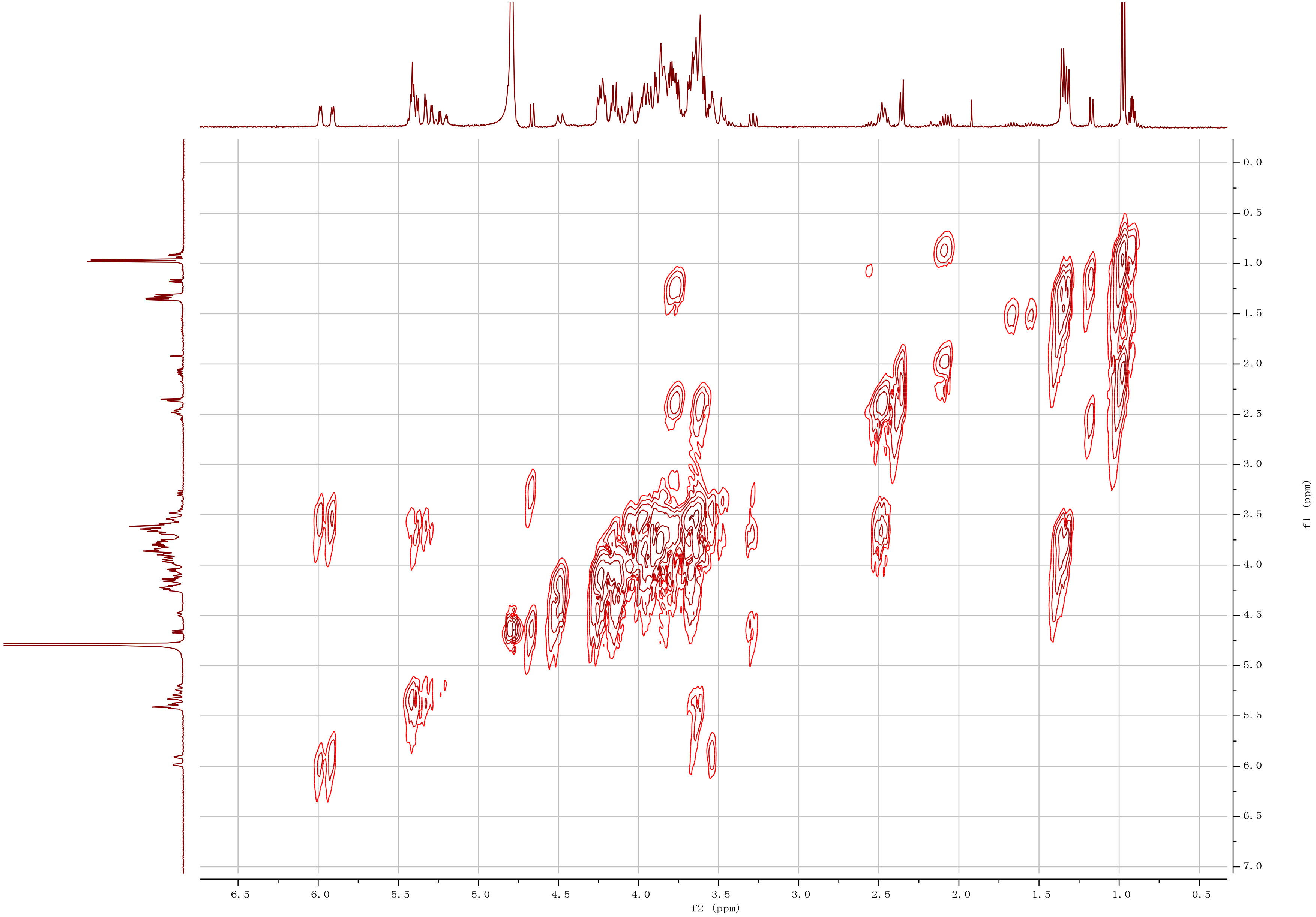

Figure S84. ^1H-^1H COSY spectrum of compound **13** (500 MHz, D_2O).

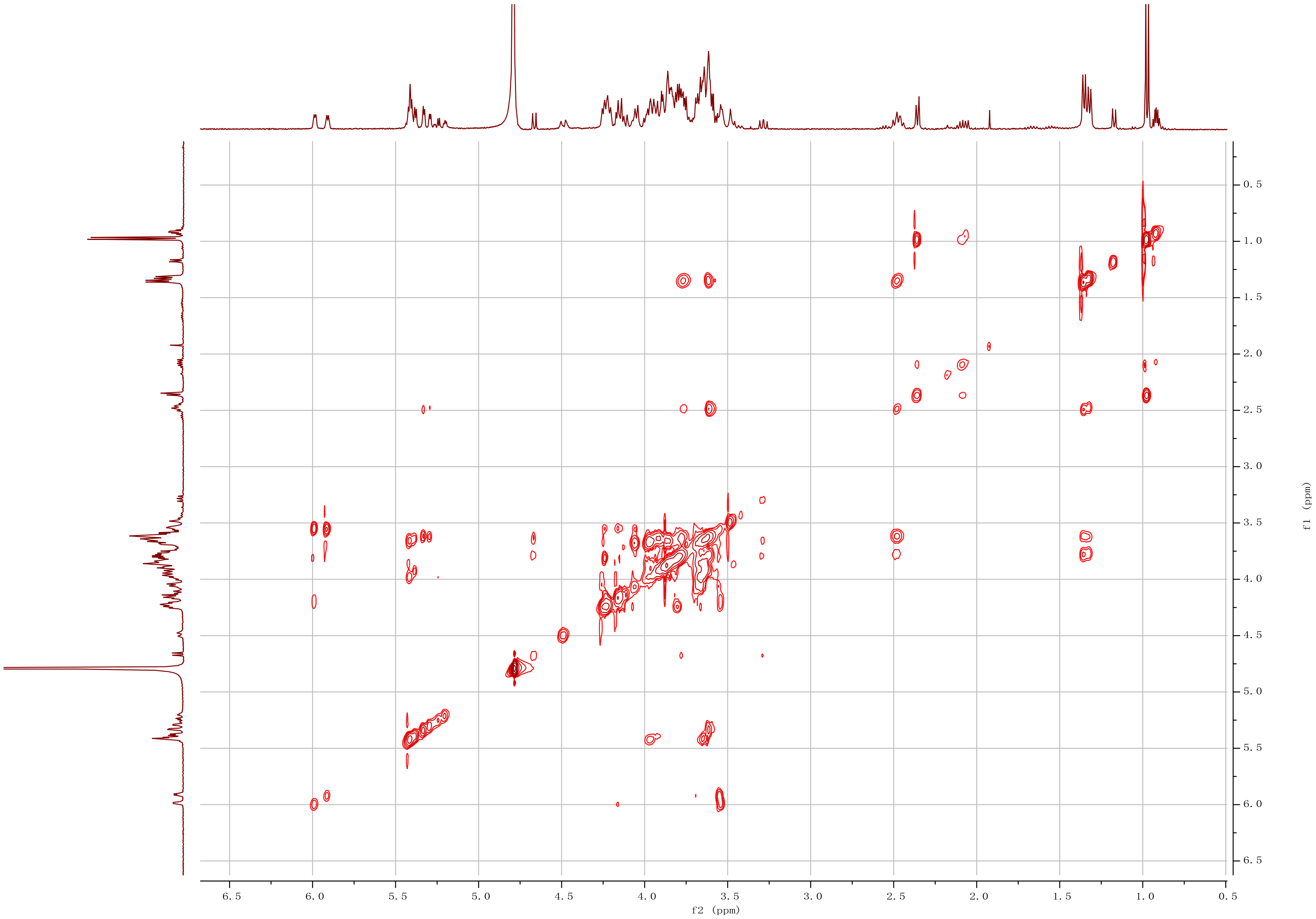

Figure S85. 2D-TOCSY spectrum of compound **13** (500 MHz, D_2O).

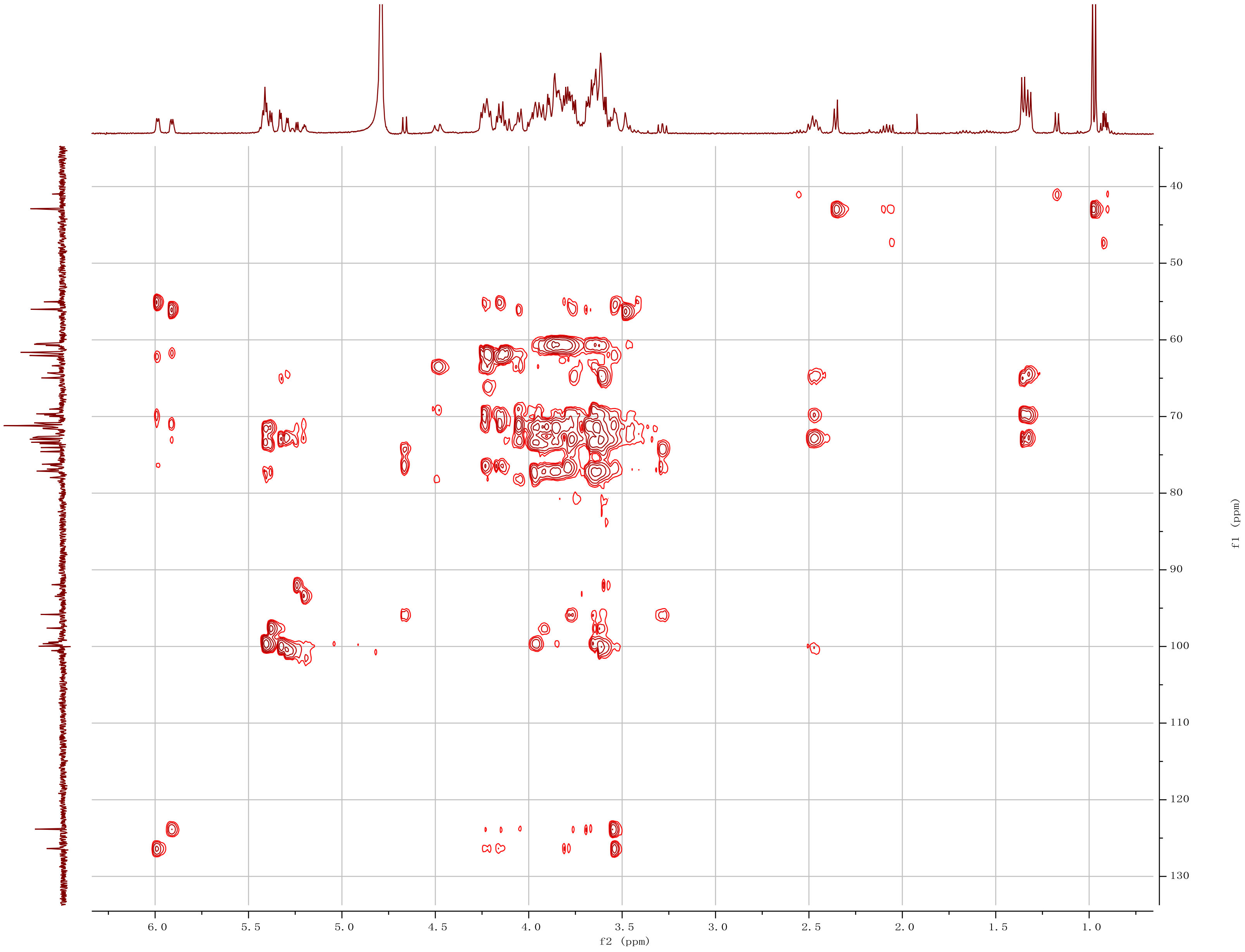

Figure S86. HSQC-TOCSY spectrum of compound **13** (500 MHz, D_2O).

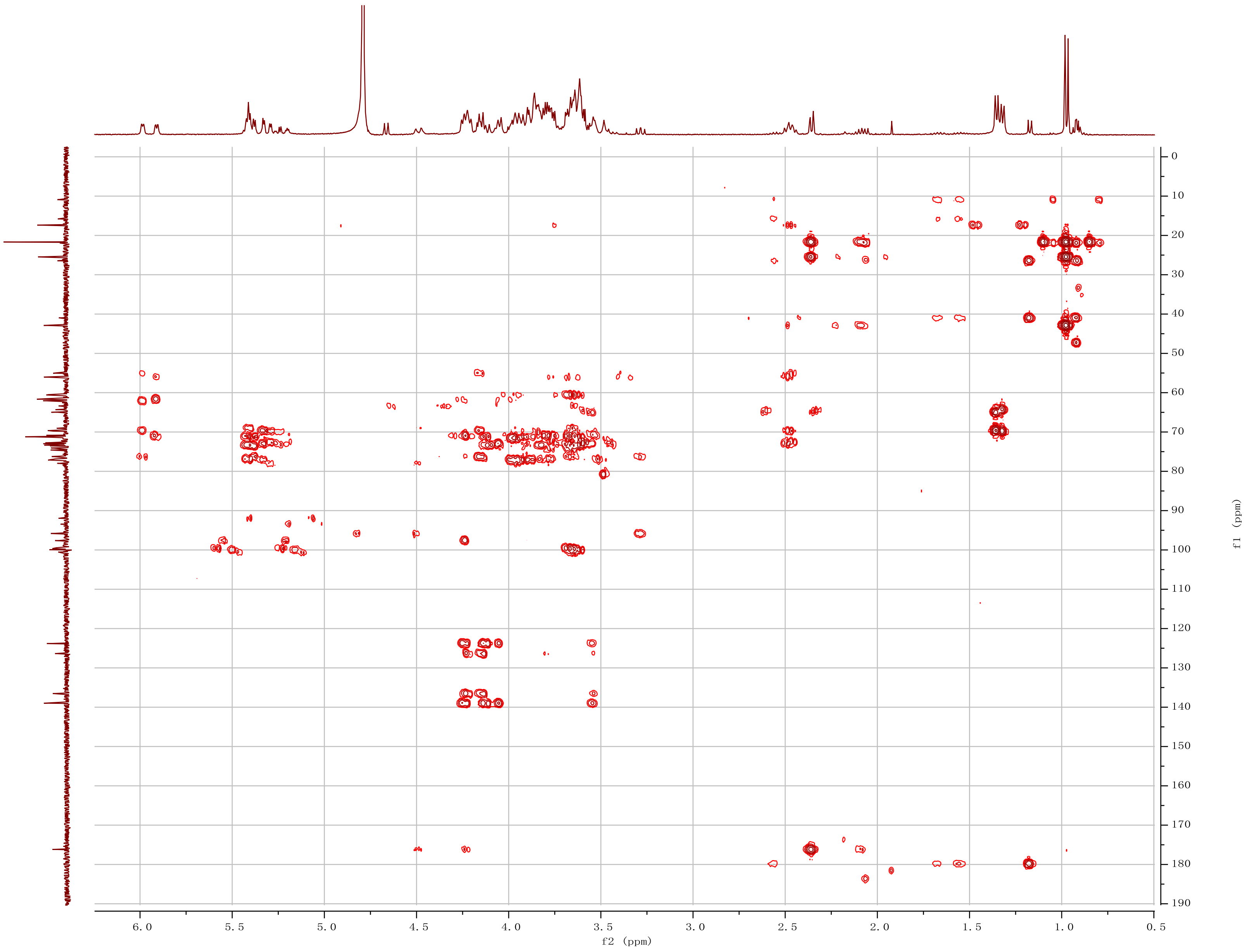

Figure S87. HMBC spectrum of compound **13** (500 MHz, D_2O).

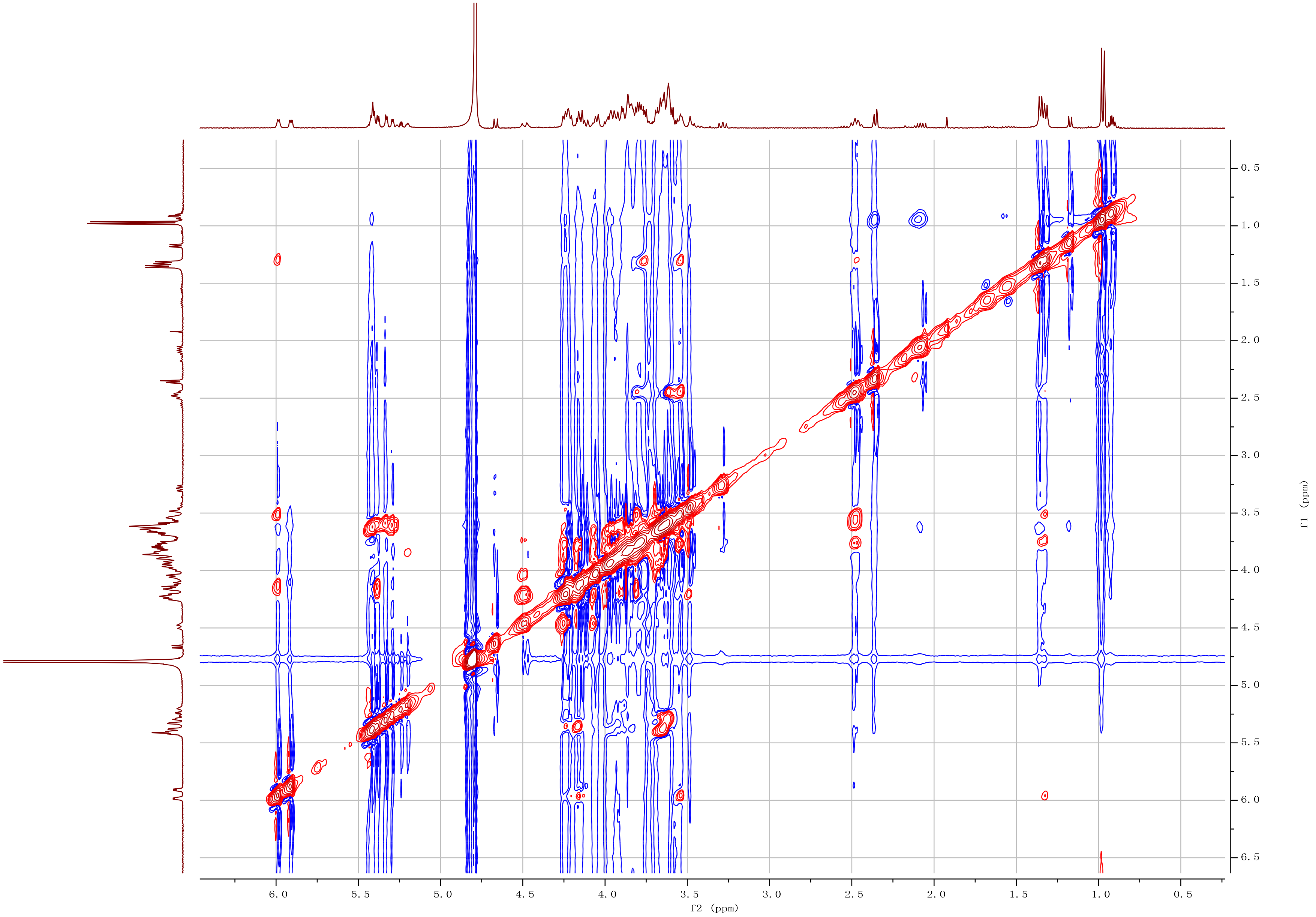

Figure S88. NOESY spectrum of compound **13** (500 MHz, D_2O).

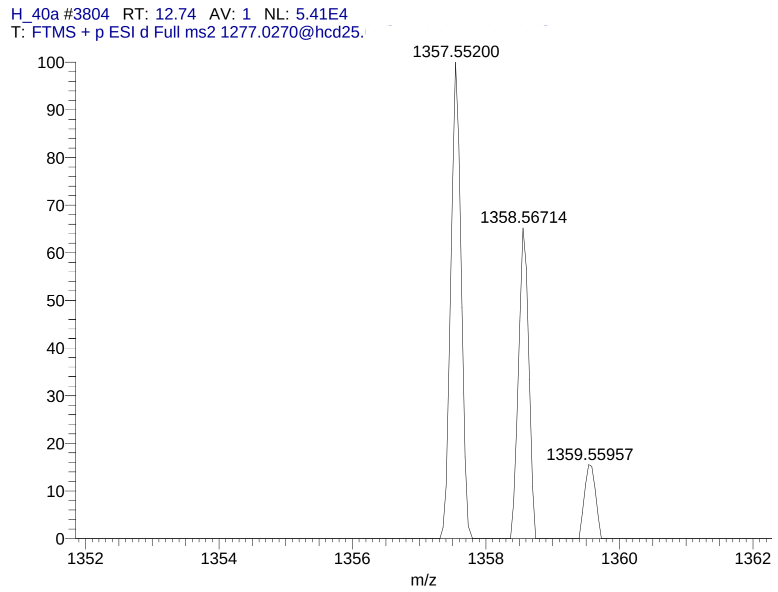

Figure S89. HRESIMS spectrum of compound **13**.

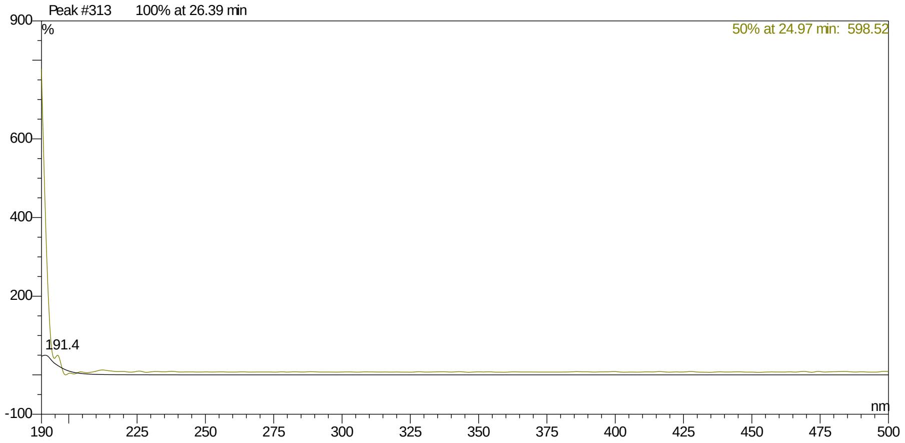

Figure S90. UV spectrum of compound **13**.

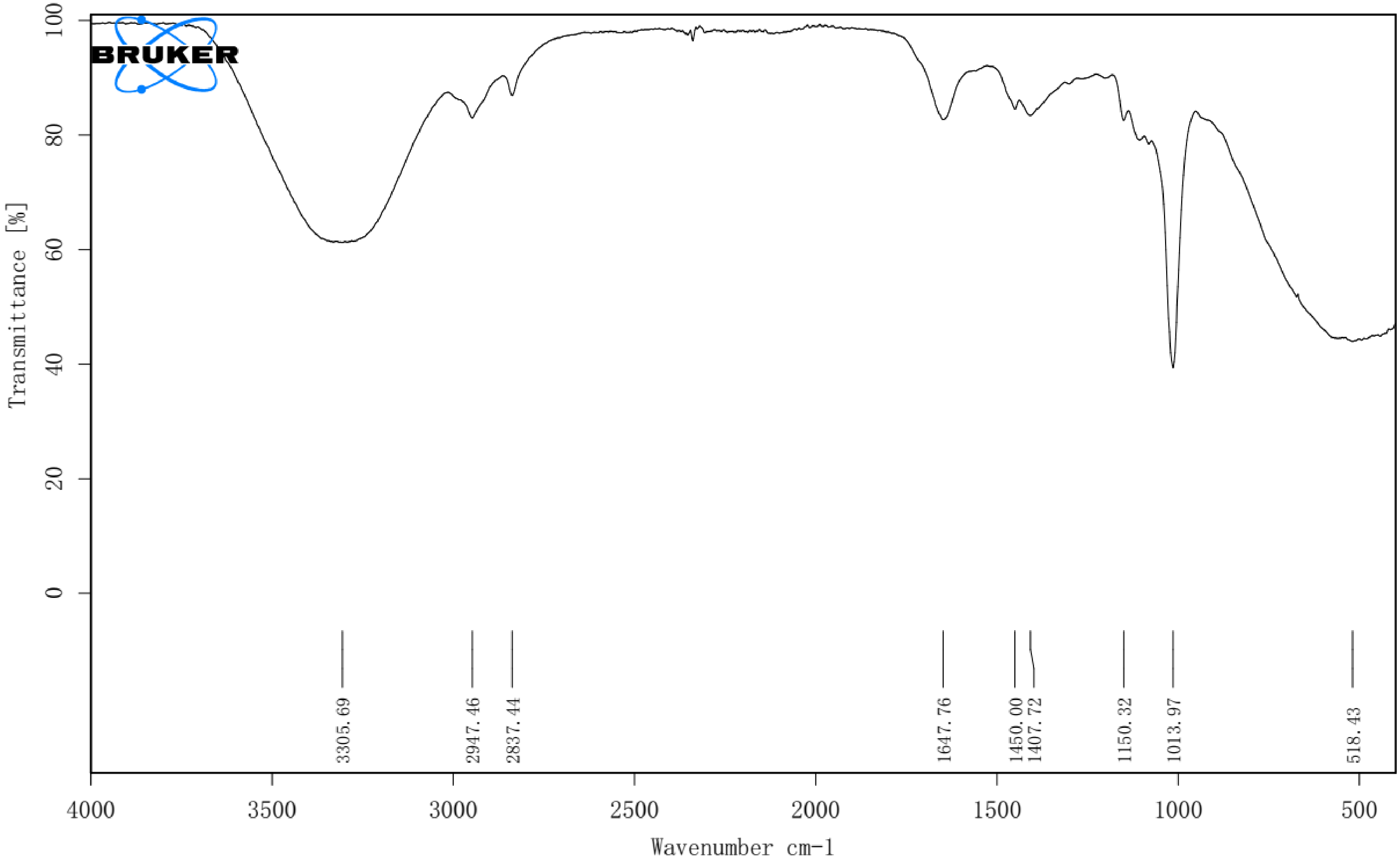

Figure S91. IR spectrum of compound **13**.

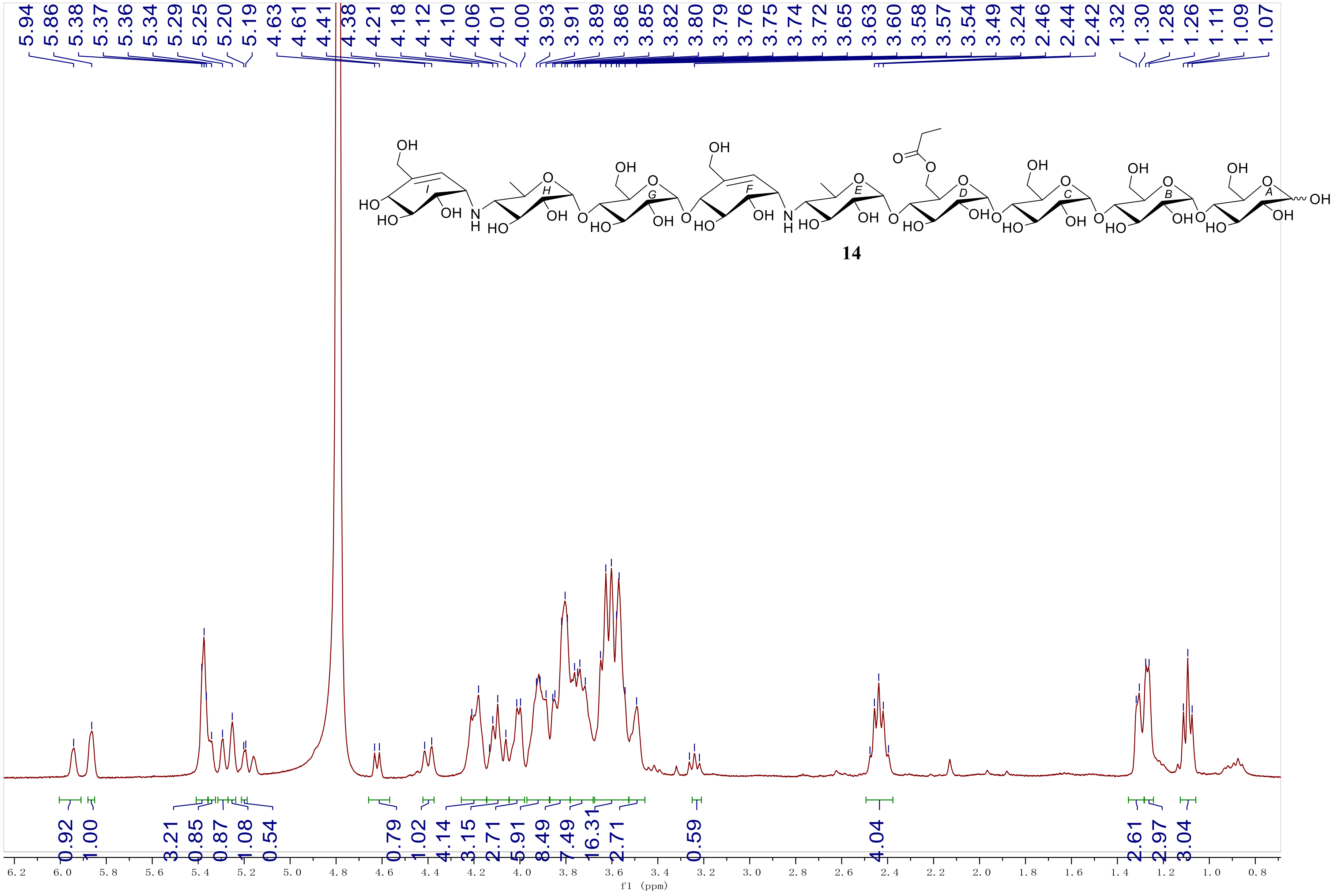

Figure S92. ^{1}H NMR spectrum of compound **14** (500 MHz, D_2O).

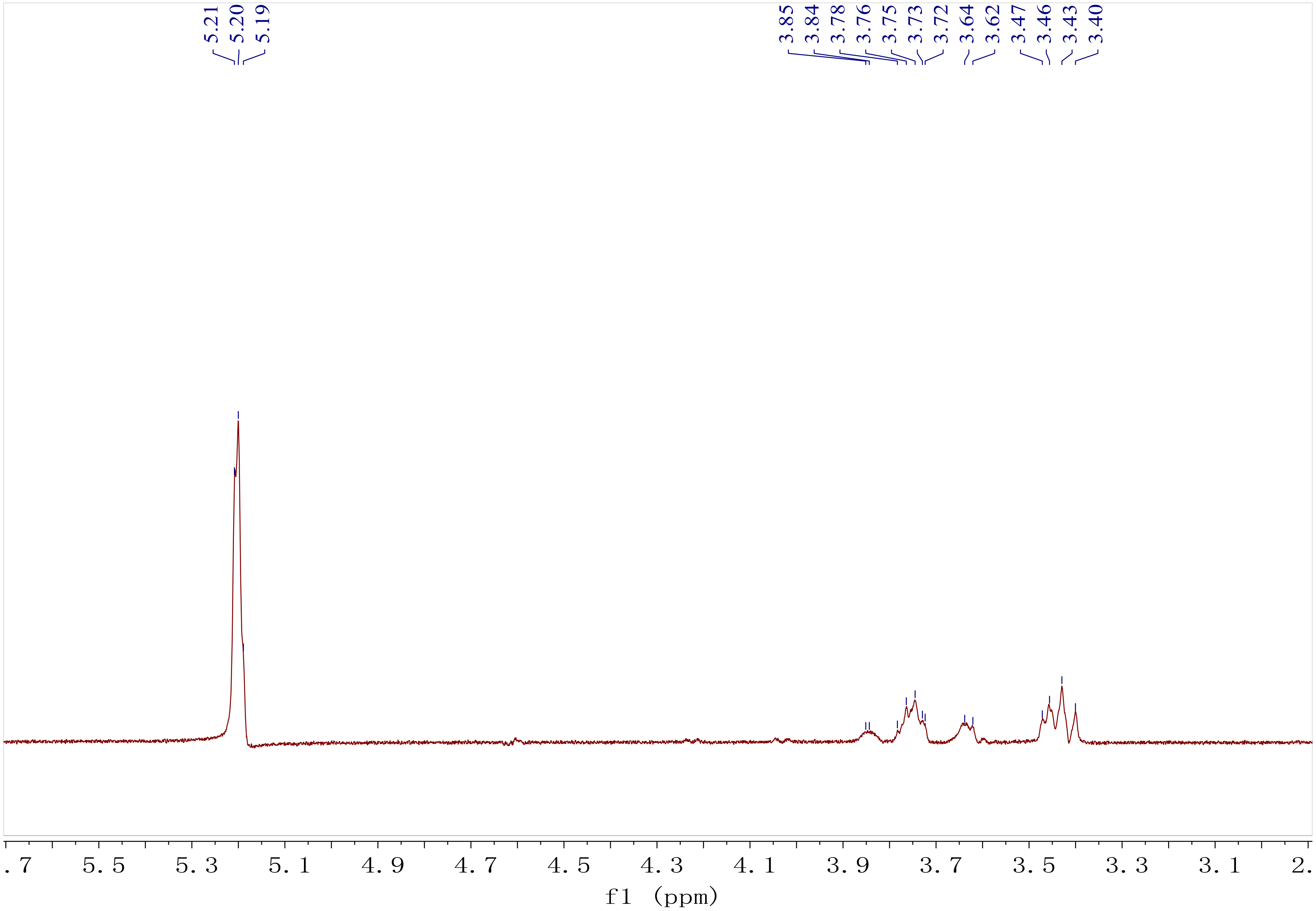

Figure S93. 1D-selective TOCSY spectrum of compound **14** (500 MHz, D_2O, excitation at δ 5.20, H-**A**1α).

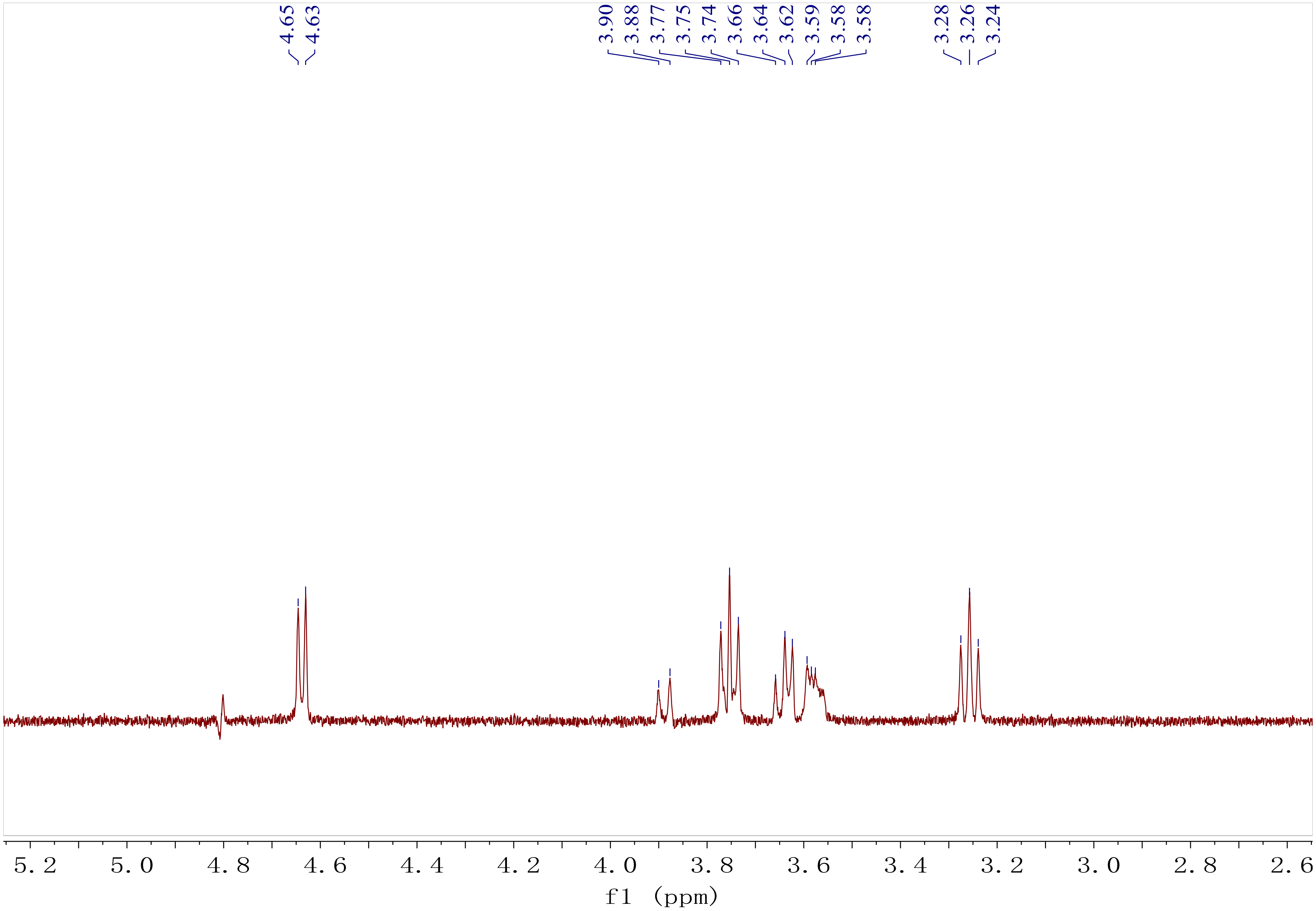

Figure S94. 1D-selective TOCSY spectrum of compound **14** (500 MHz, D_2O, excitation at δ 4.62, H-**A**1β).

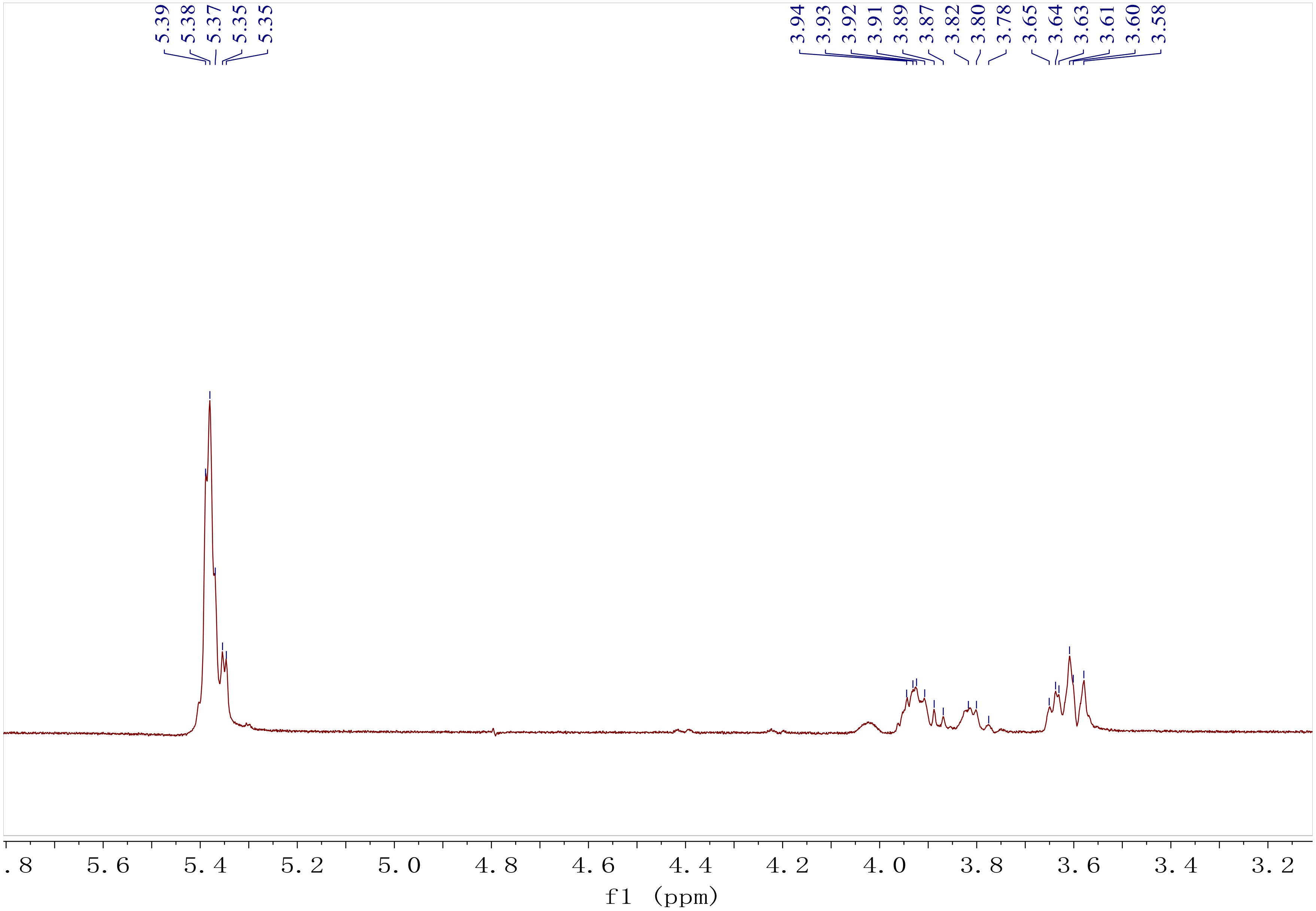

Figure S95. 1D-selective TOCSY spectrum of compound **14** (500 MHz, D_2O, excitation at δ 5.38, H-**B**1, H-**C**1, and H-**D**1).

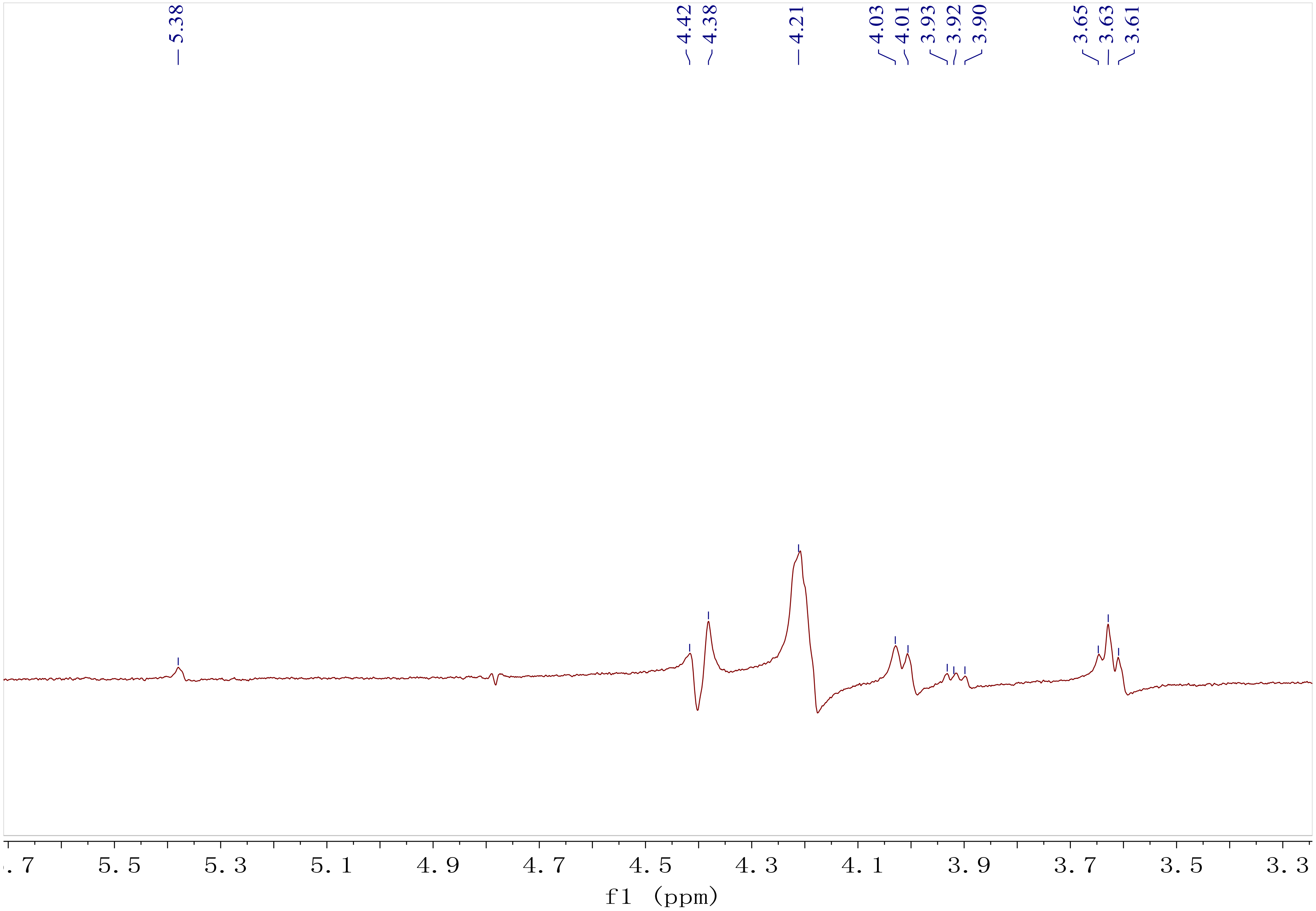

Figure S96. 1D-selective TOCSY spectrum of compound **14** (500 MHz, D_2O, excitation at δ 4.40, H-**D**6a).

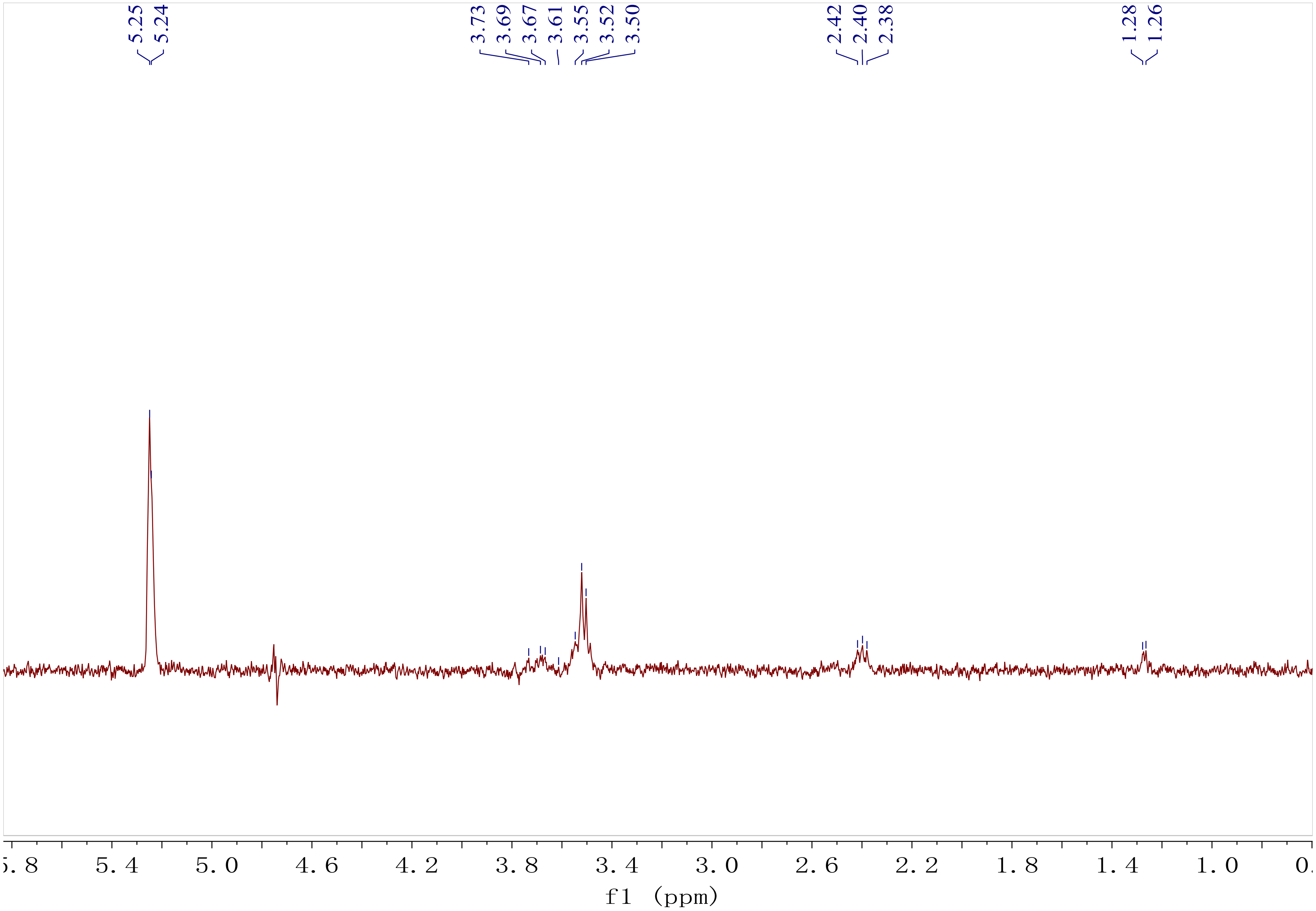

Figure S97. 1D-selective TOCSY spectrum of compound **14** (500 MHz, D_2O, excitation at δ 5.25, H-**E**1).

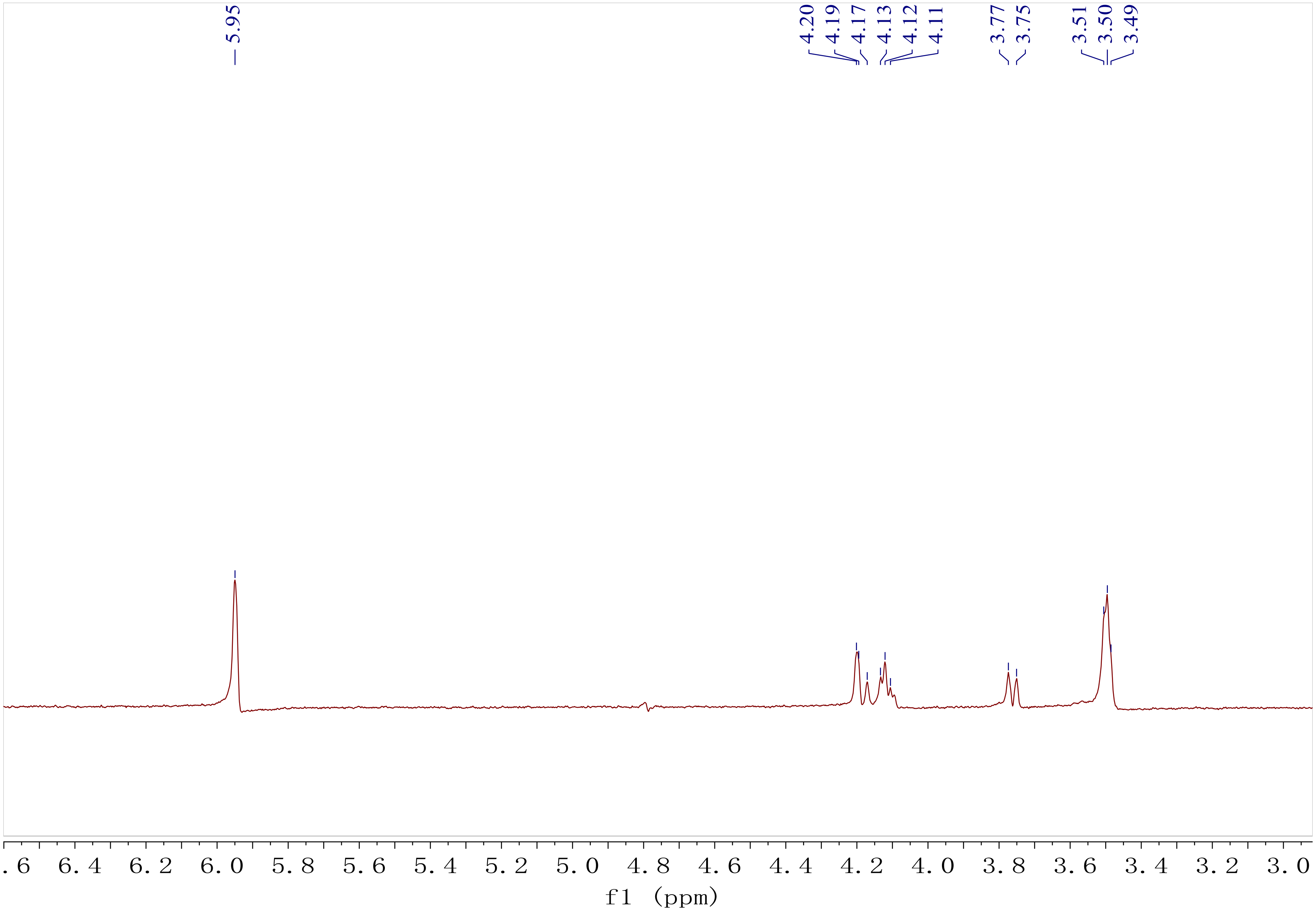

Figure S98. 1D-selective TOCSY spectrum of compound **14** (500 MHz, D_2O, excitation at δ 5.94, H-**F**7).

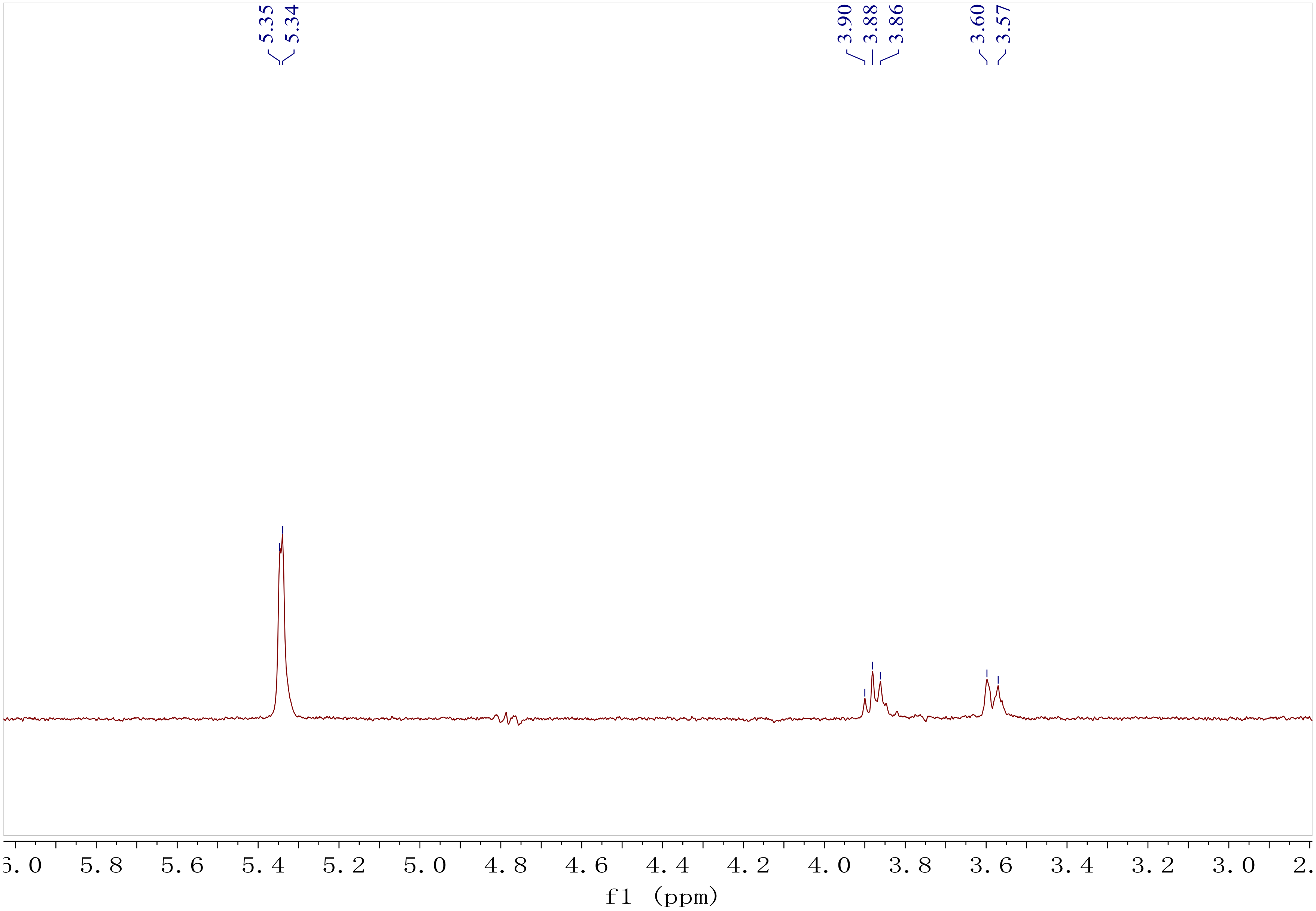

Figure S99. 1D-selective TOCSY spectrum of compound **14** (500 MHz, D_2O, excitation at δ 5.34, H-**G1**).

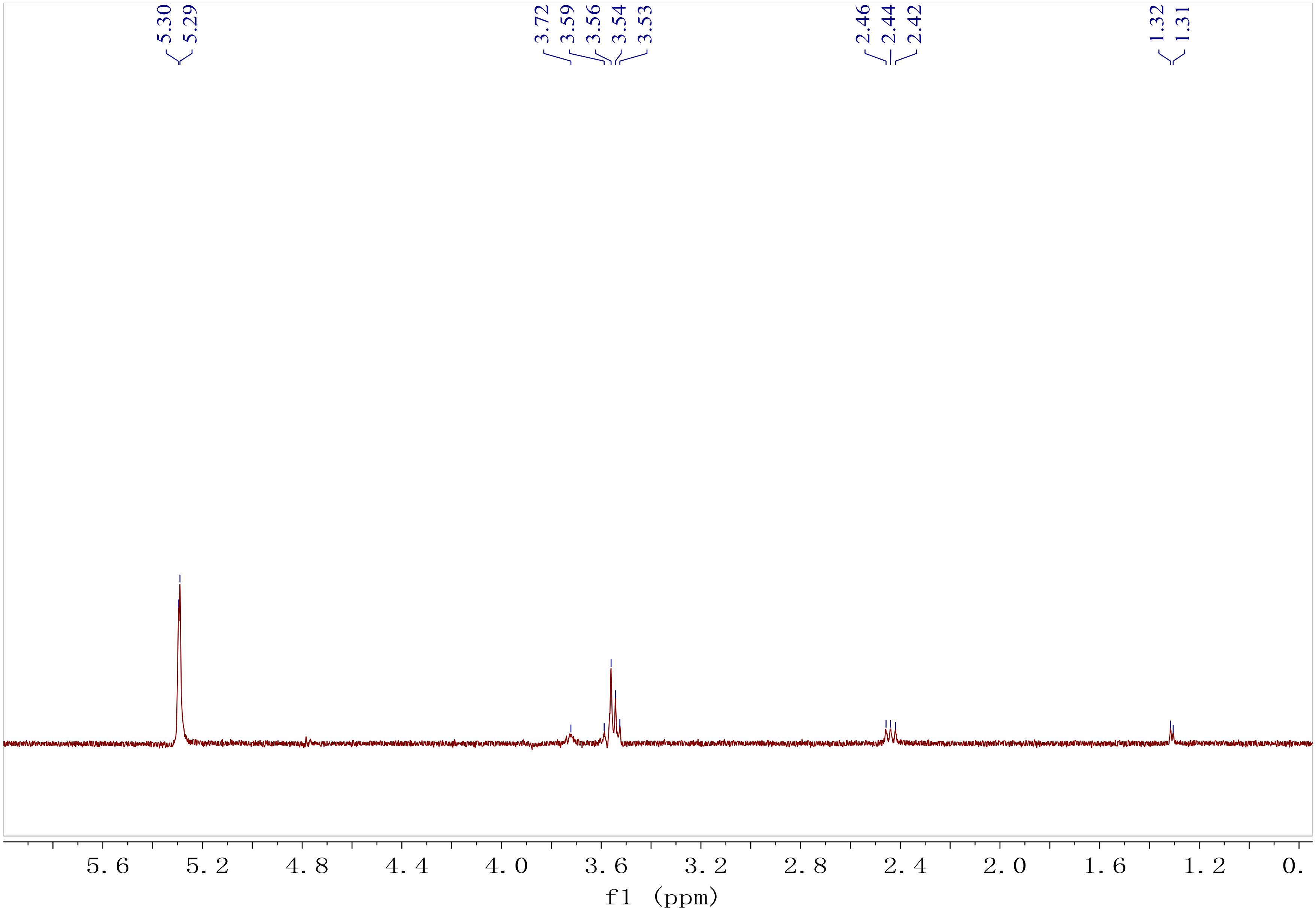

Figure S100. 1D-selective TOCSY spectrum of compound **14** (500 MHz, D_2O, excitation at δ 5.29, H-**H1**).

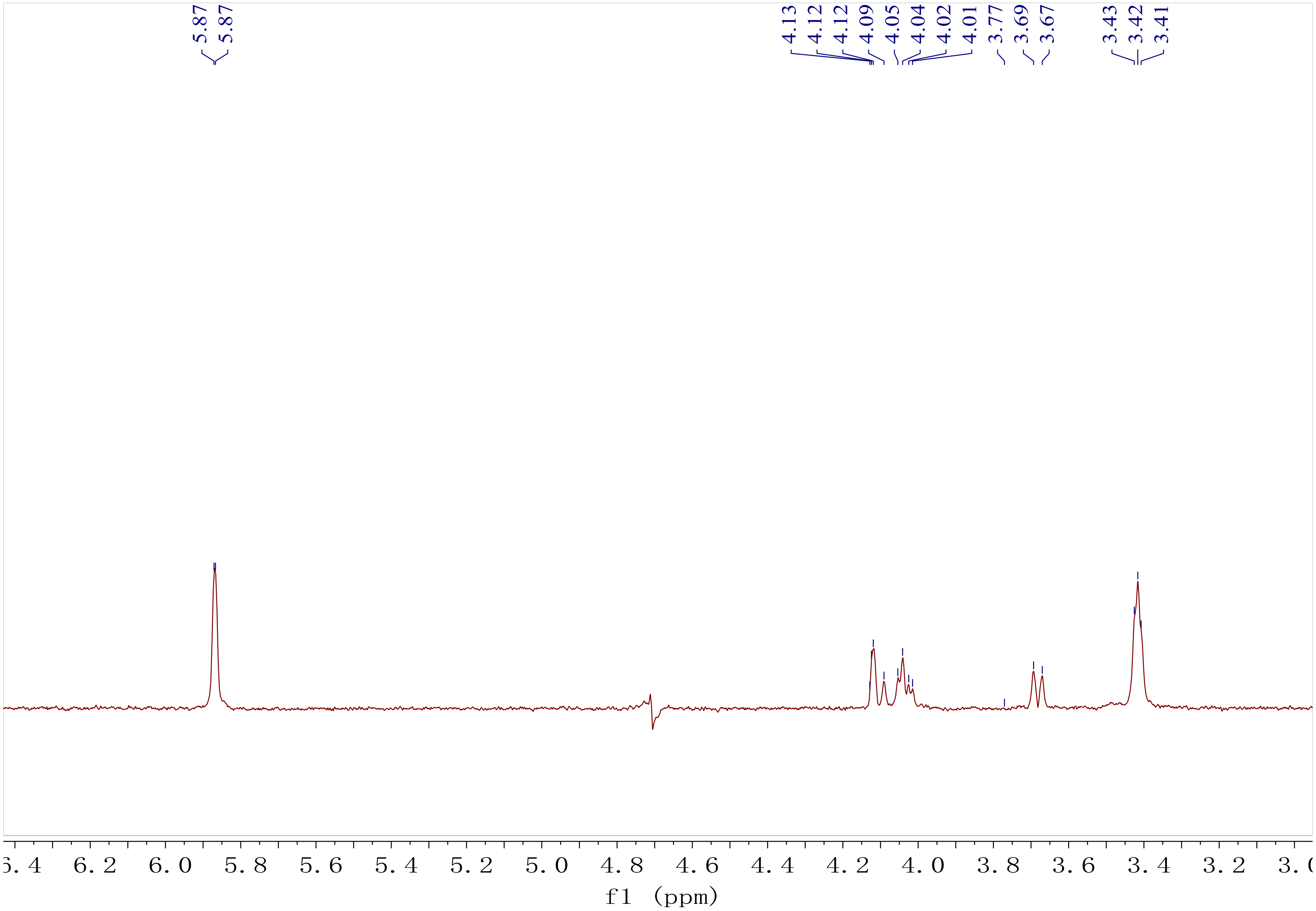

Figure S101. 1D-selective TOCSY spectrum of compound **14** (500 MHz, D_2O, excitation at δ 5.86, H-**I1**).

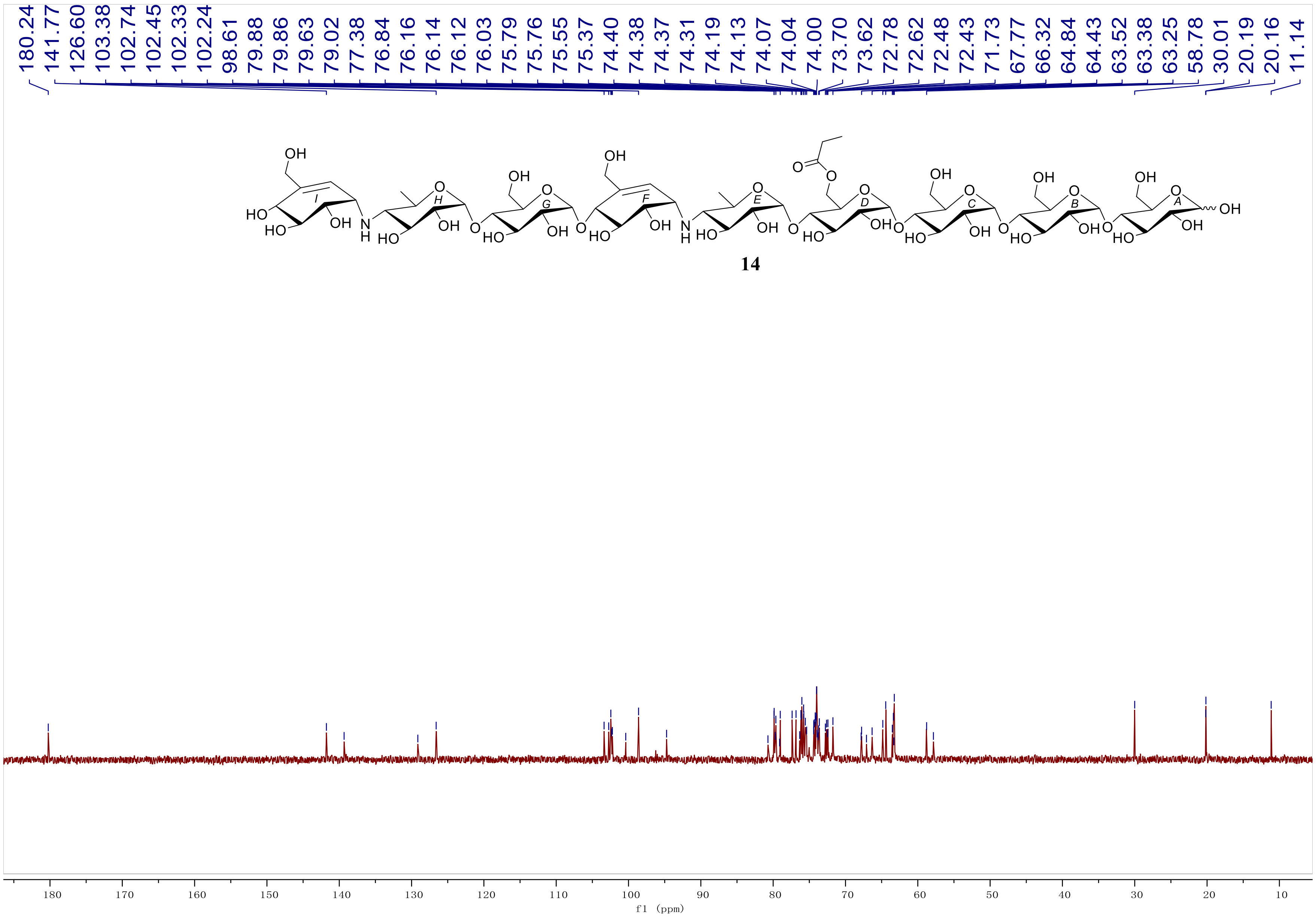

Figure S102. ^{13}C NMR spectrum of compound **14** (125 MHz, D_2O).

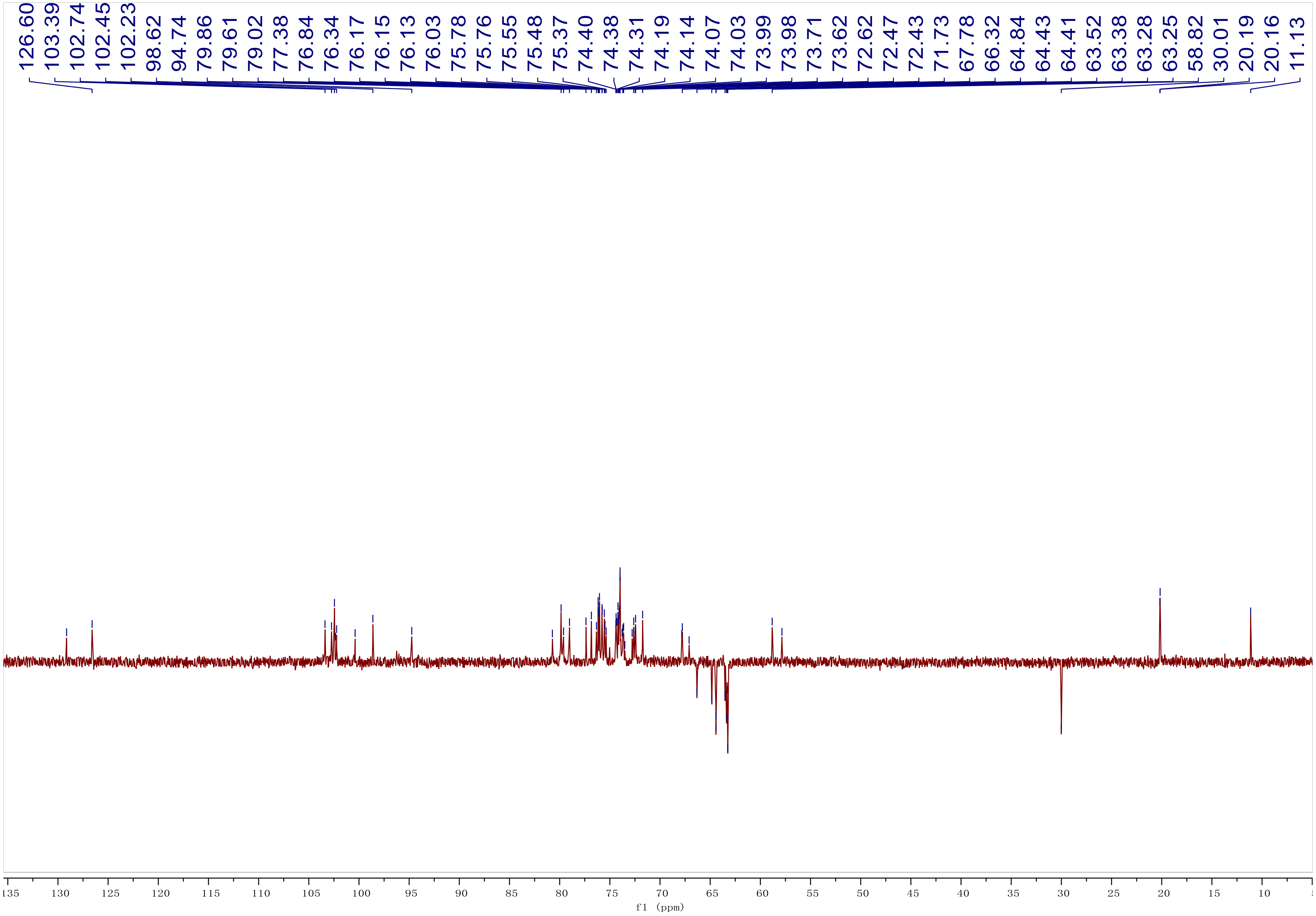

Figure S103. DEPT-135 spectrum of compound **14** (125 MHz, D_2O).

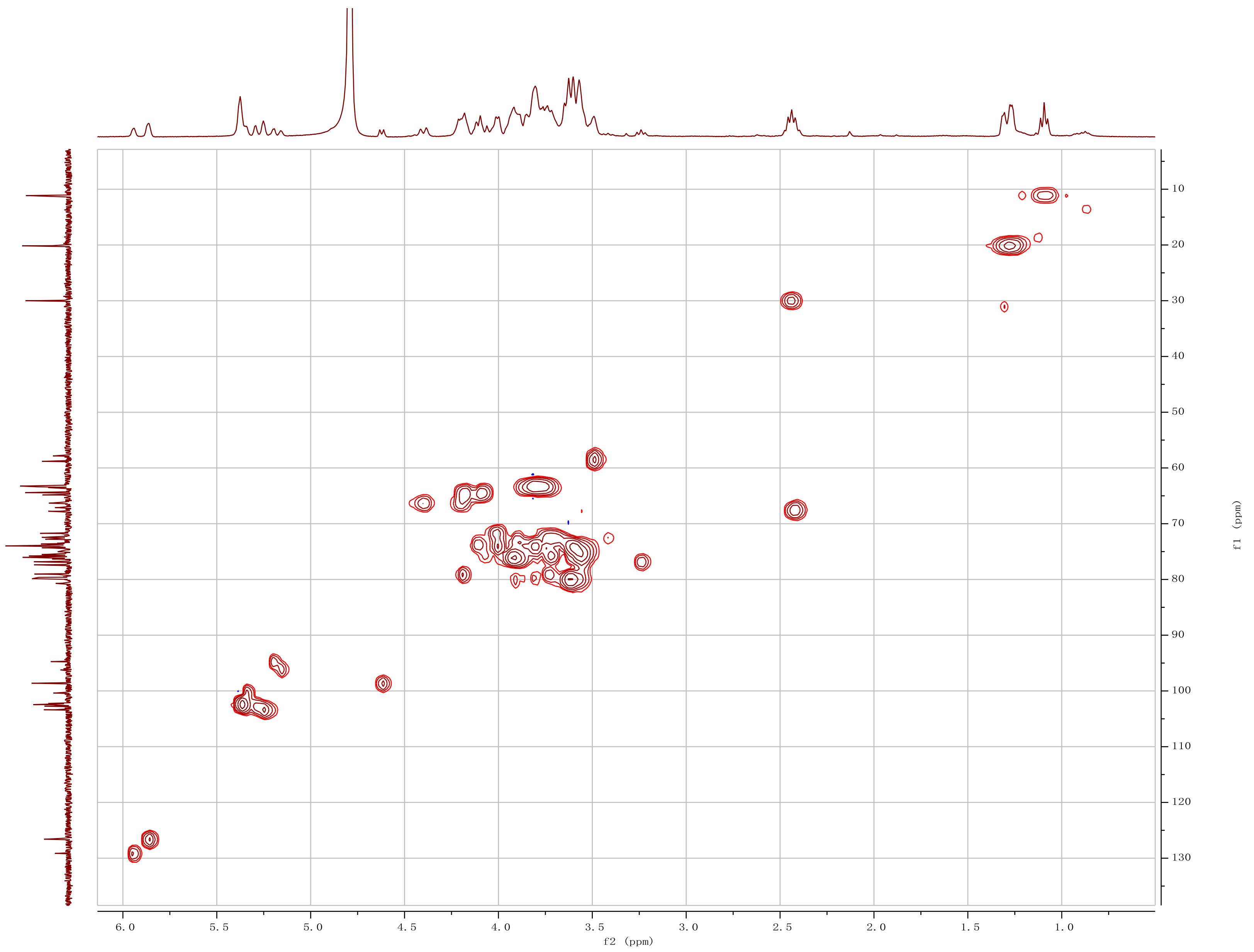

Figure S104. HSQC spectrum of compound **14** (500 MHz, D_2O).

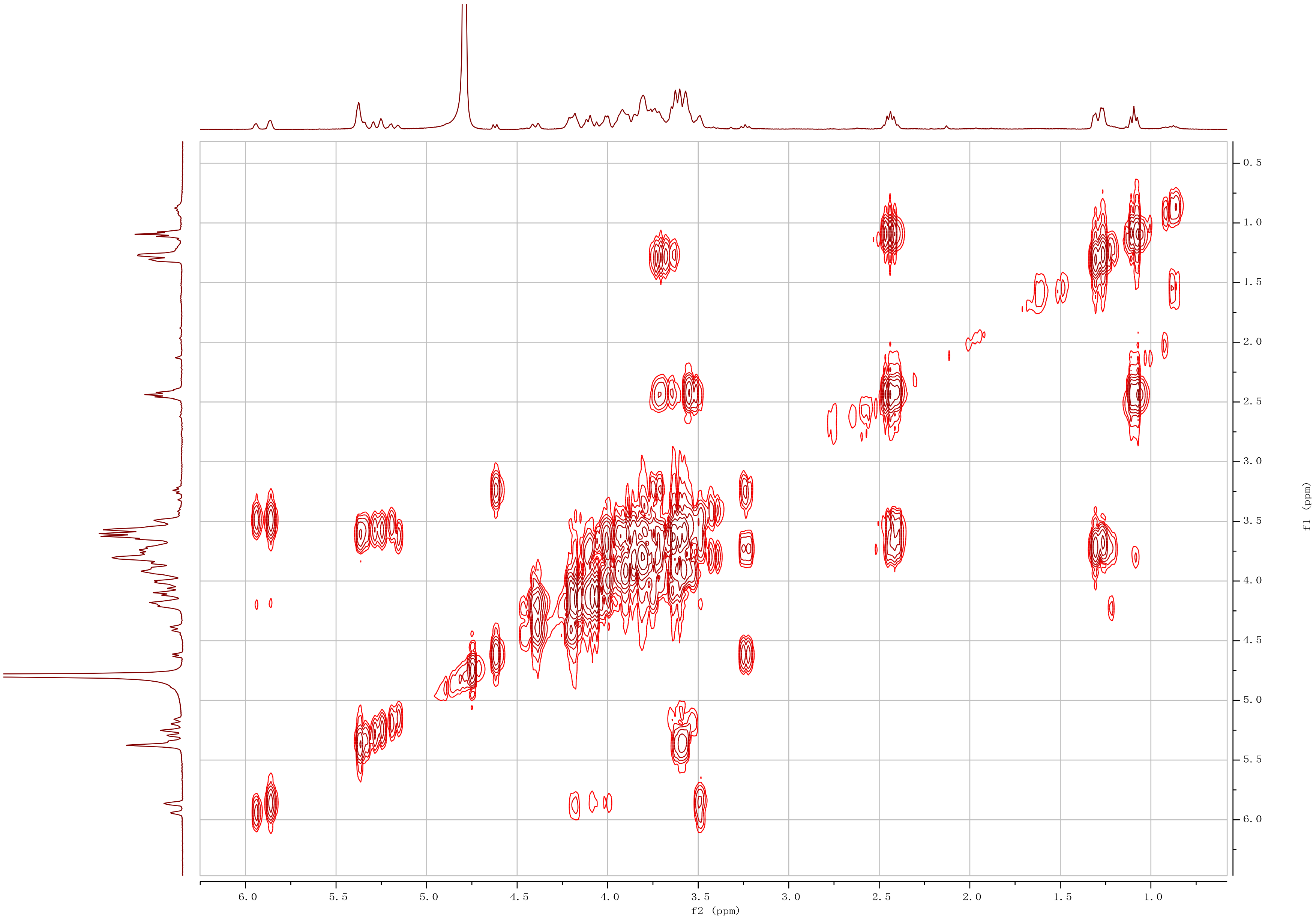

Figure S105. ^{1}H-^{1}H COSY spectrum of compound **14** (500 MHz, D_2O).

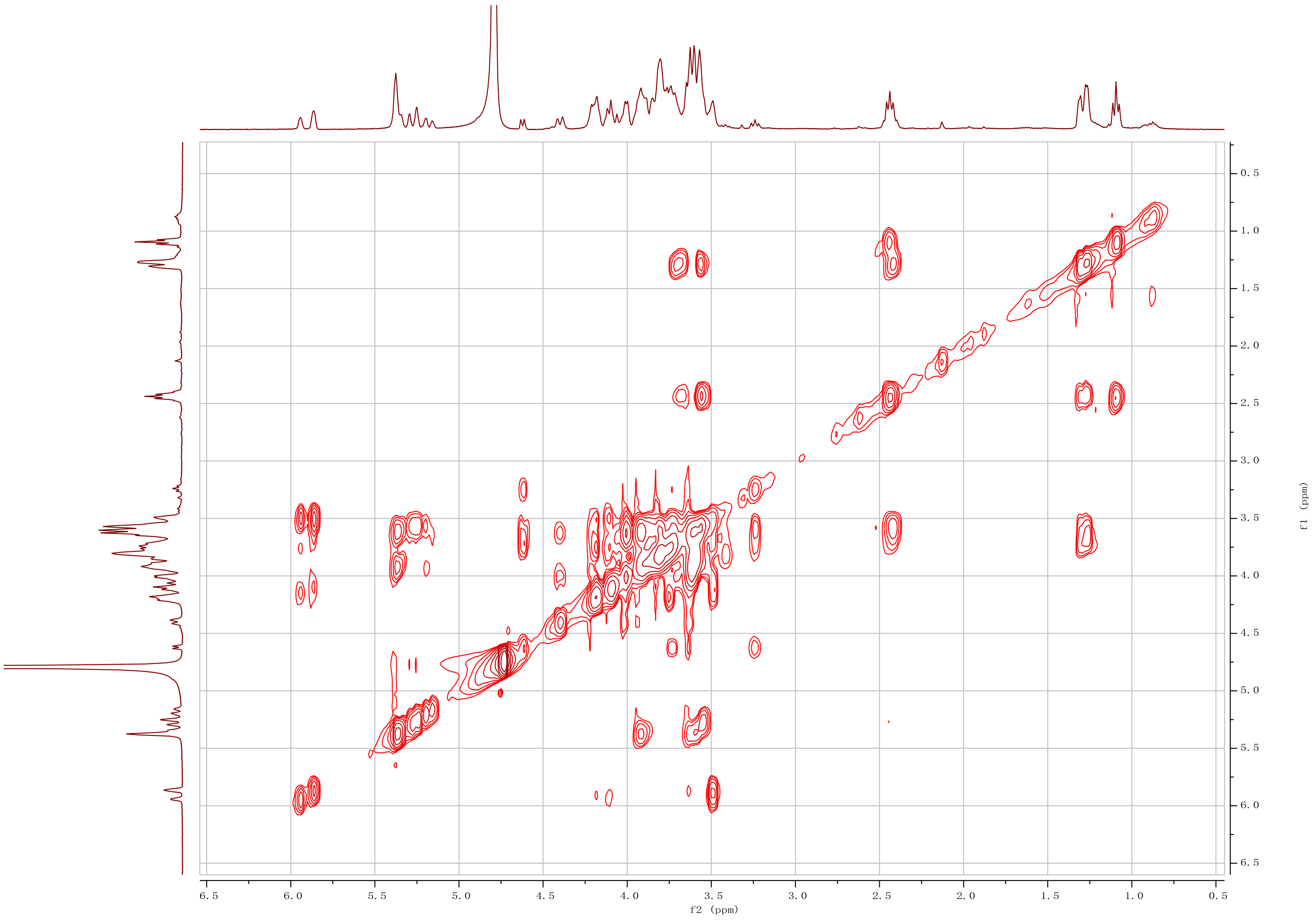

Figure S106. 2D-TOCSY spectrum of compound **14** (500 MHz, D_2O).

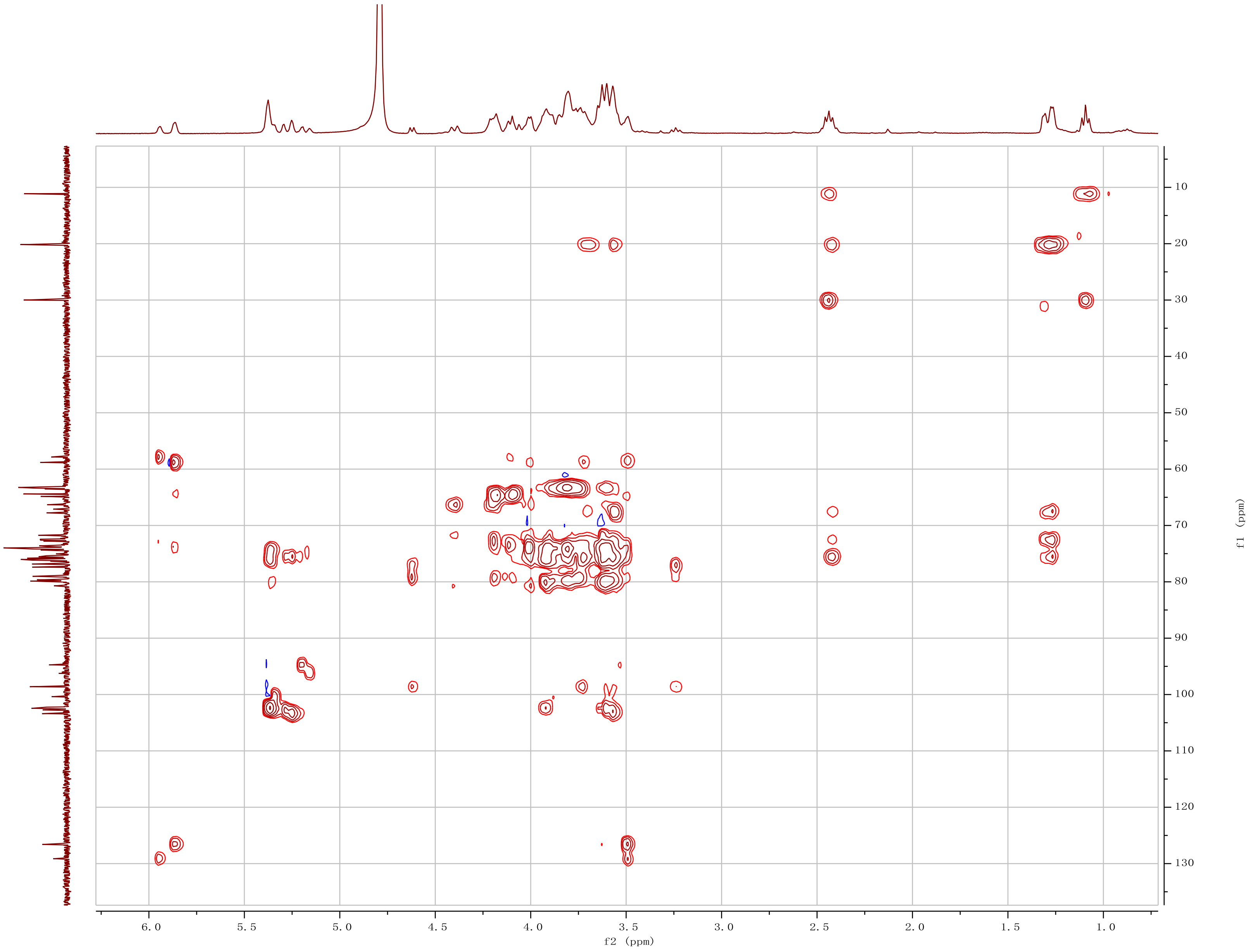

Figure S107. HSQC-TOCSY spectrum of compound **14** (500 MHz, D_2O).

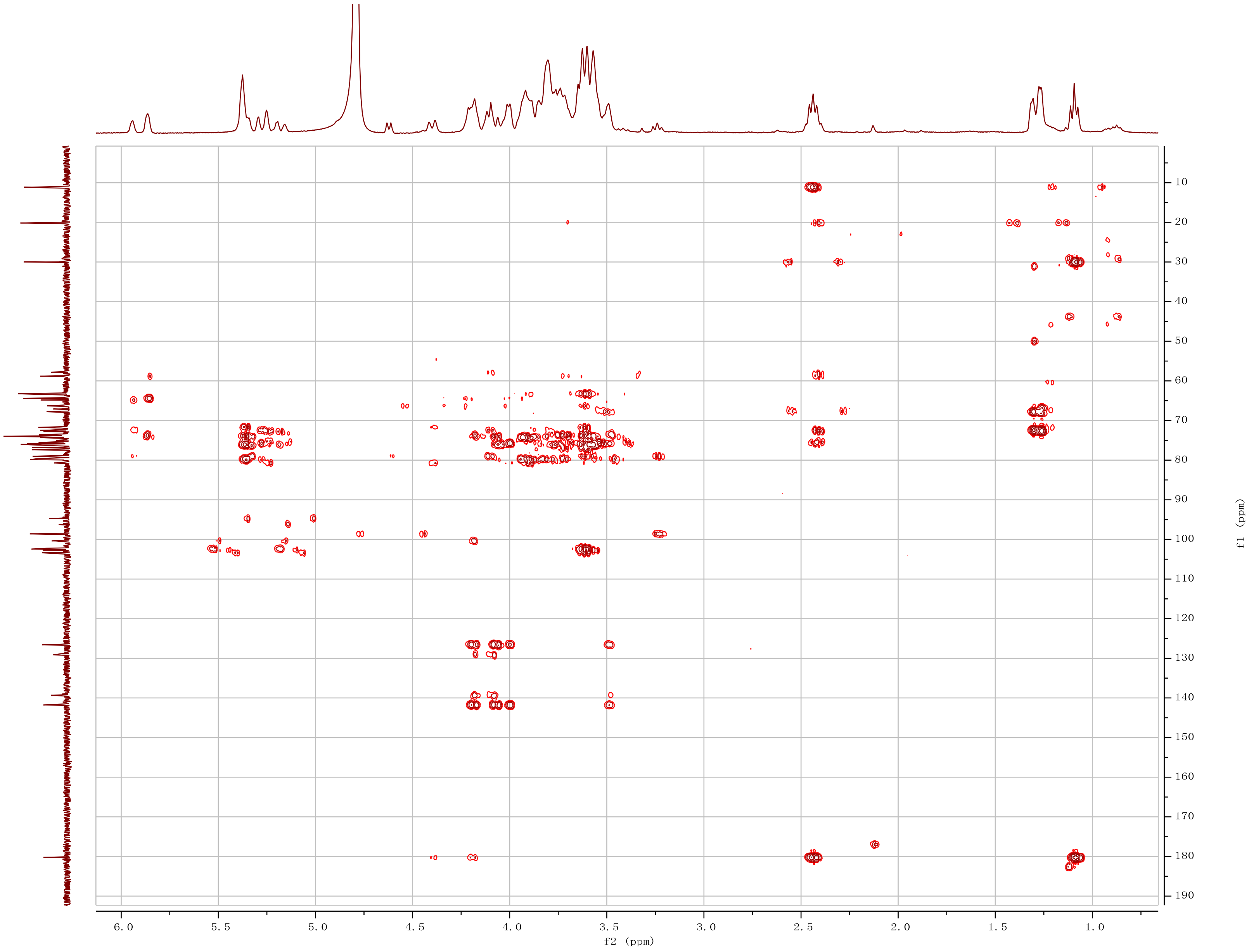

Figure S108. HMBC spectrum of compound **14** (500 MHz, D_2O).

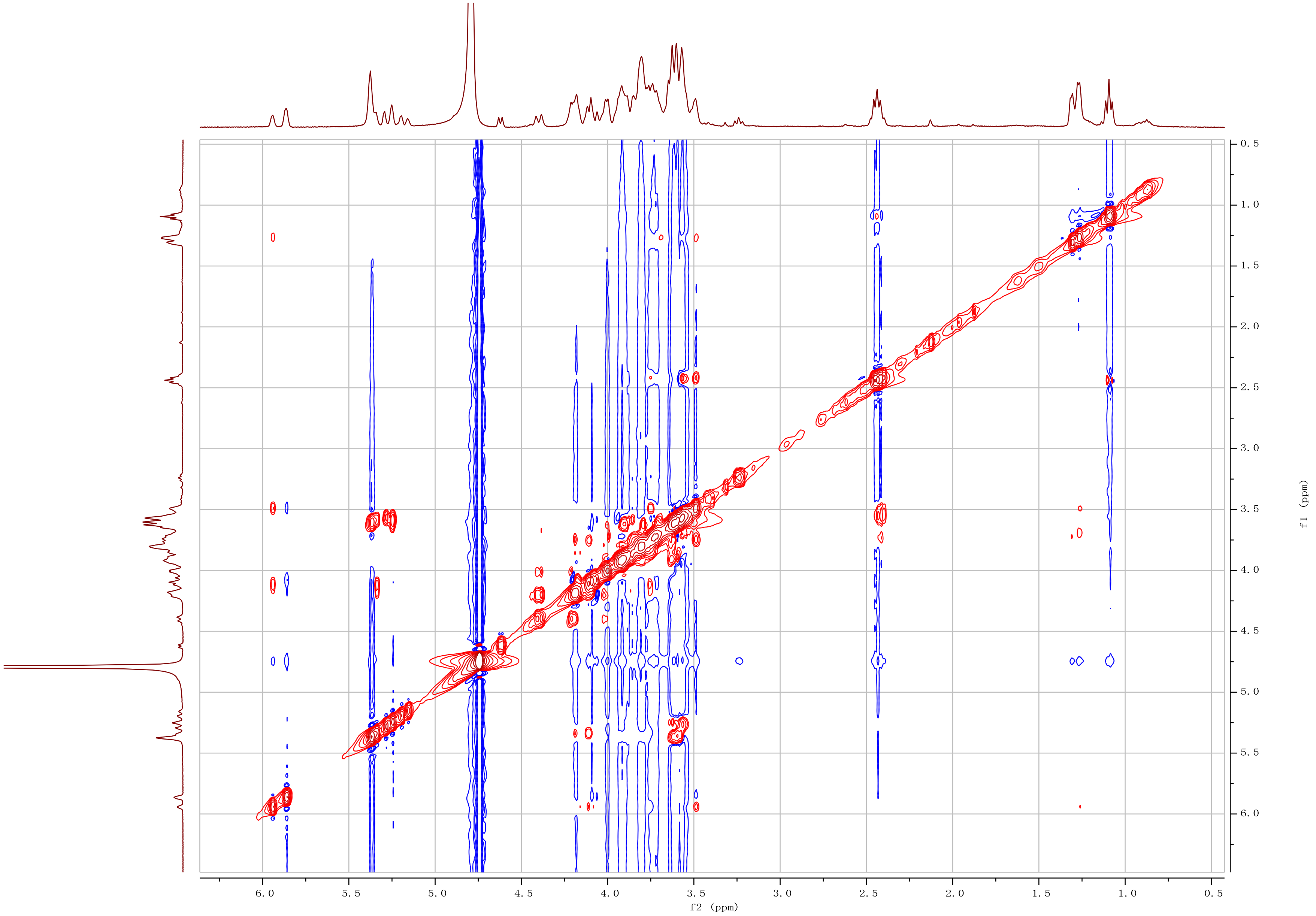

Figure S109. NOESY spectrum of compound **14** (500 MHz, D_2O).

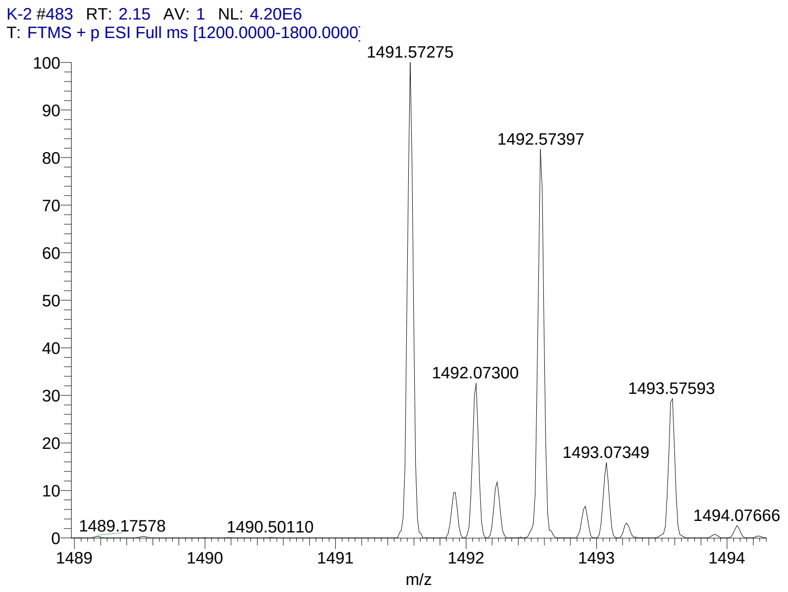

Figure S110. HRESIMS spectrum of compound **14**.

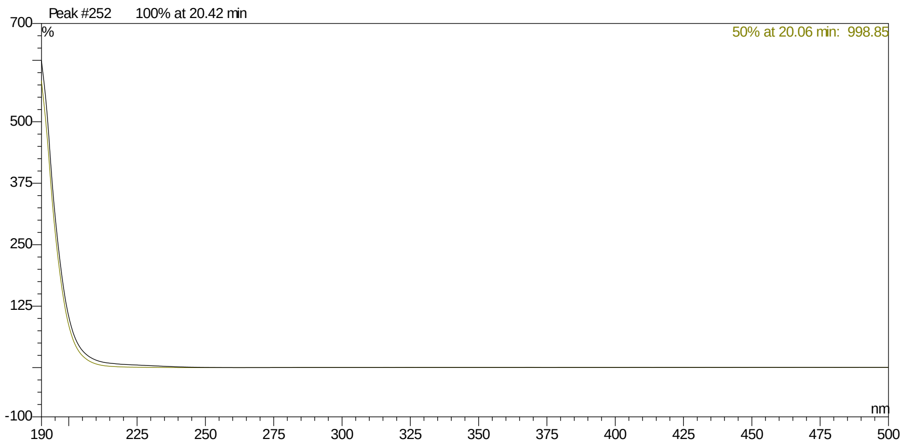

Figure S111. UV spectrum of compound **14**.

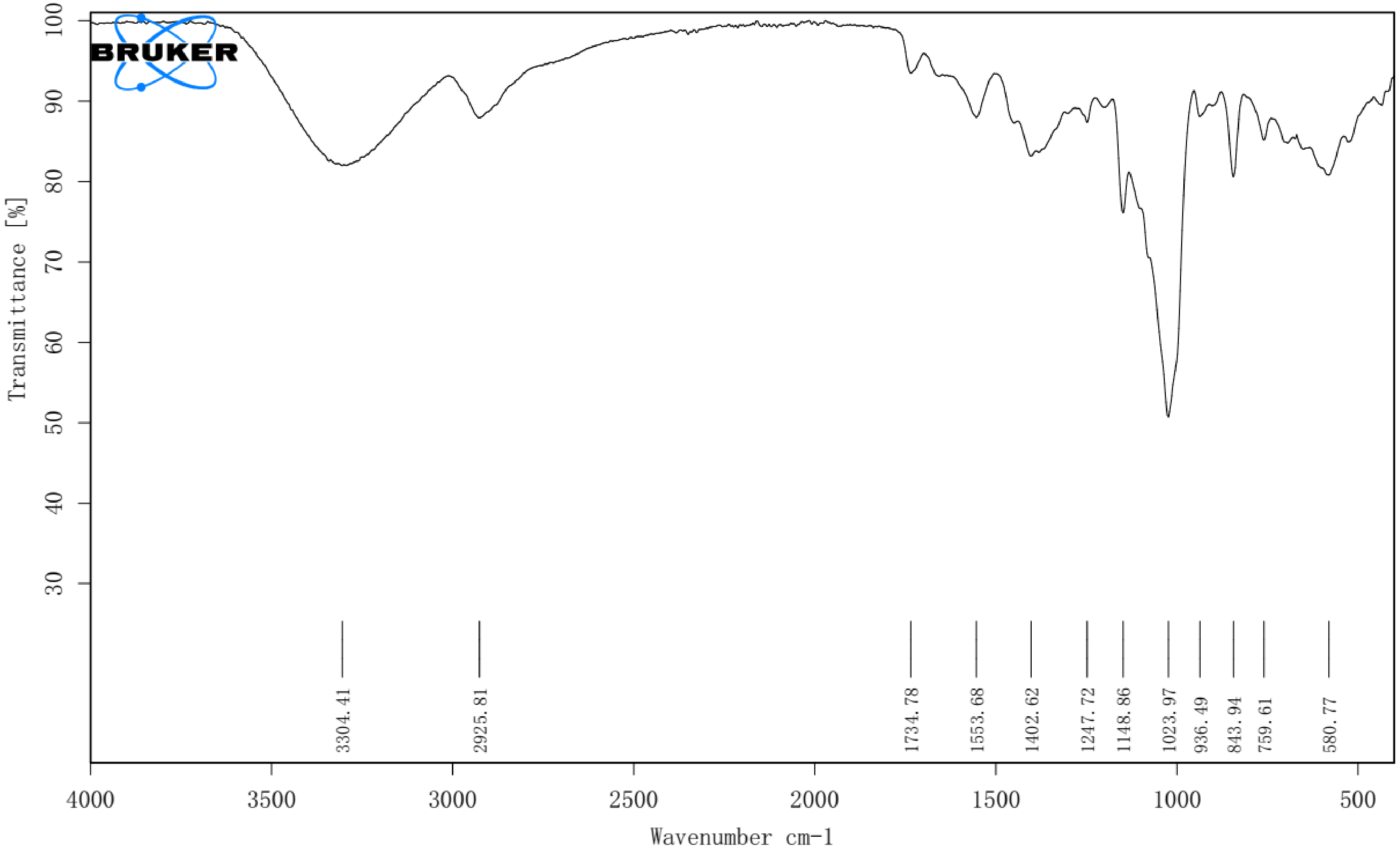

Figure S112. IR spectrum of compound **14**.

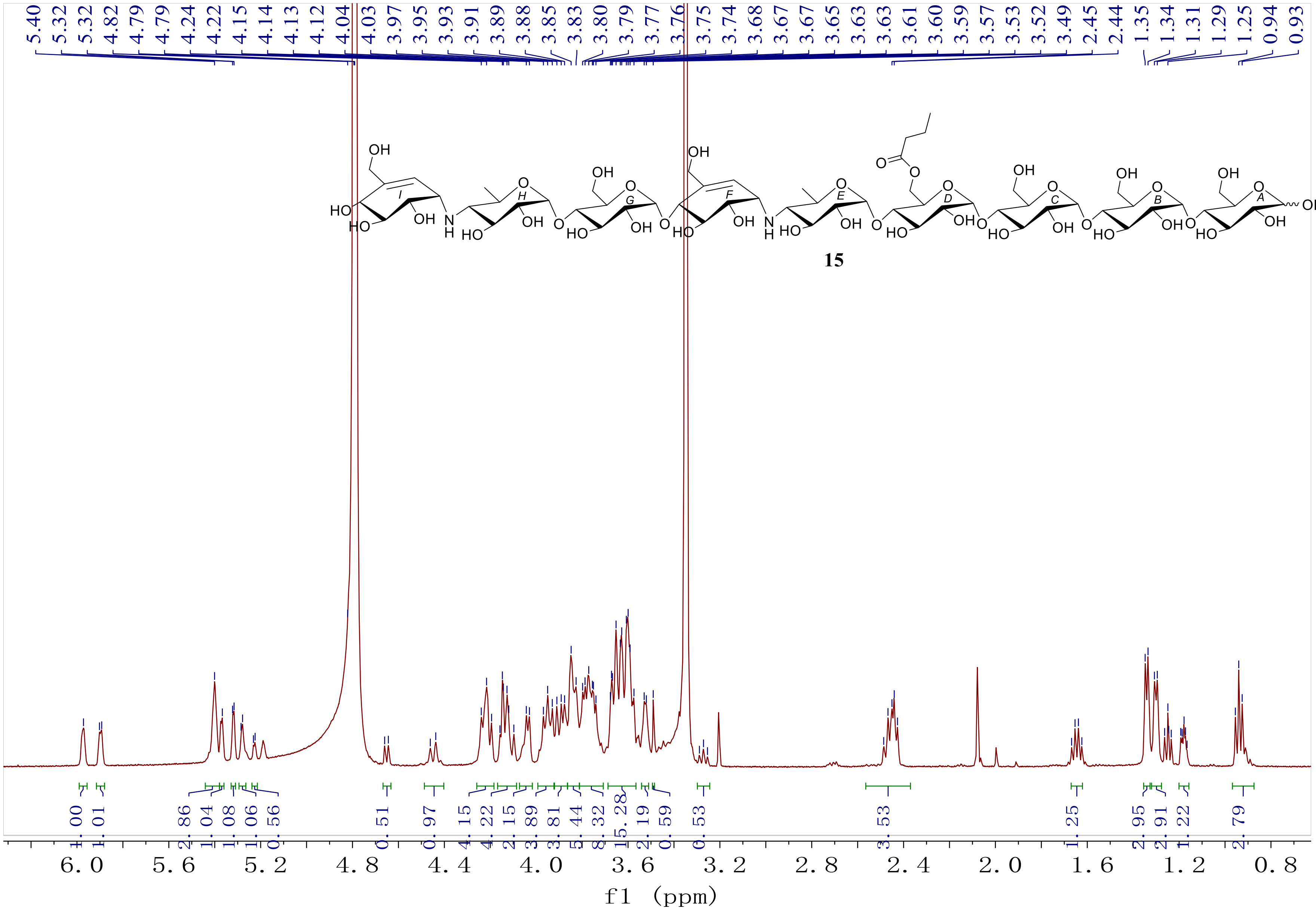

Figure S113. ^{1}H NMR spectrum of compound **15** (500 MHz, D_2O). 137

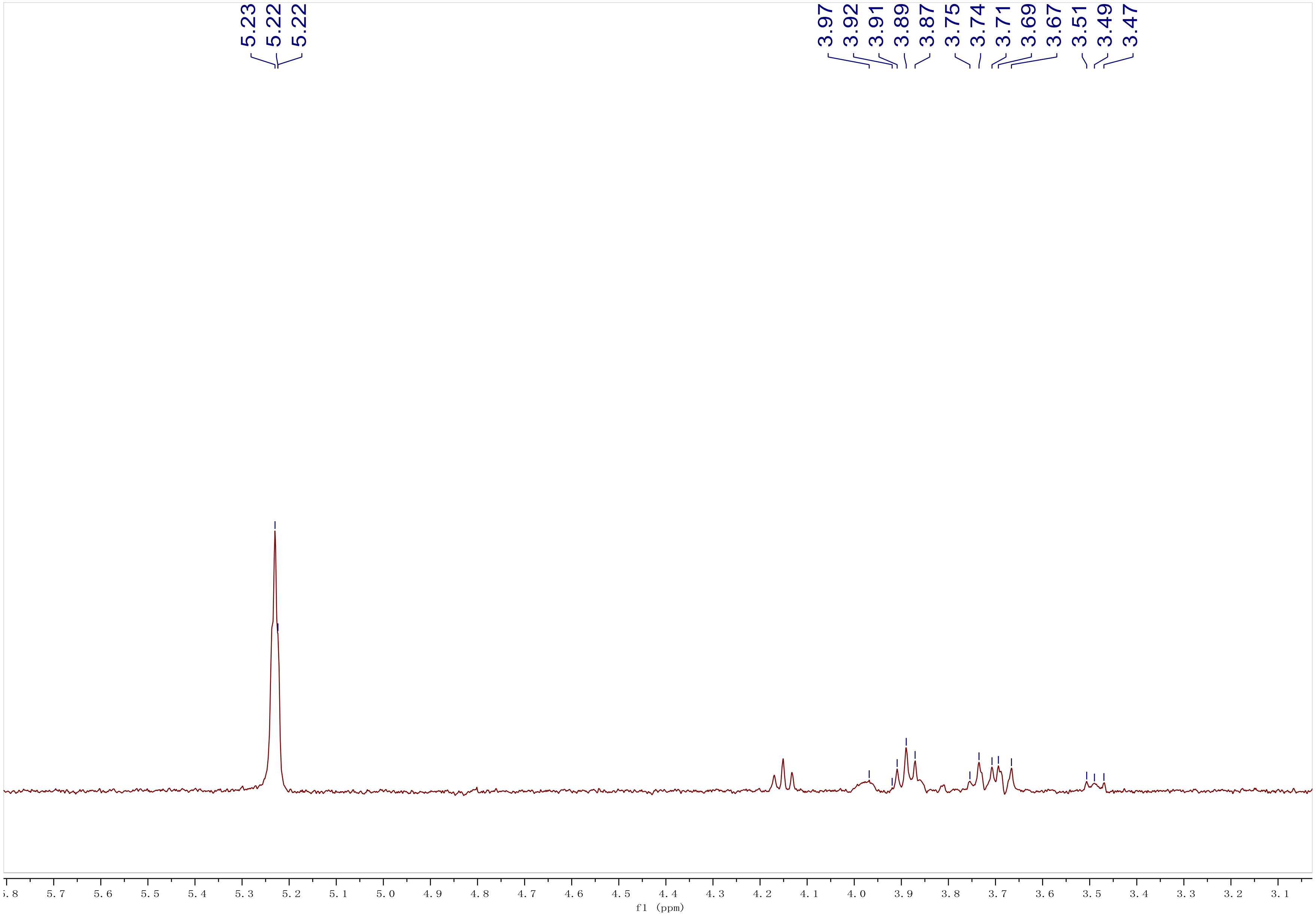

Figure S114. 1D-selective TOCSY spectrum of compound **15** (500 MHz, D_2O, excitation at δ 5.23, H-A1α).

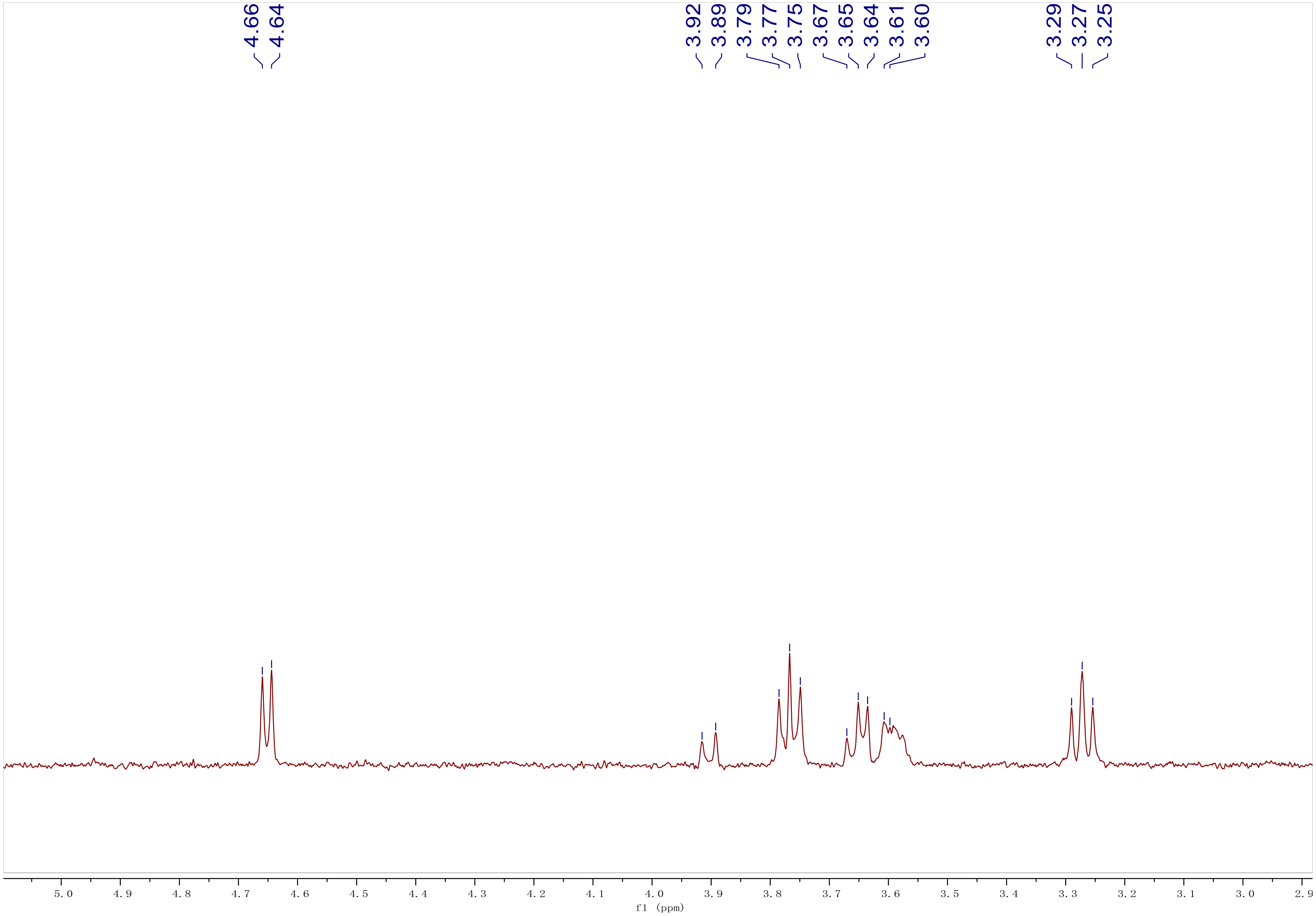

Figure S115. 1D-selective TOCSY spectrum of compound **15** (500 MHz, D_2O, excitation at δ 4.65, H-A1β).

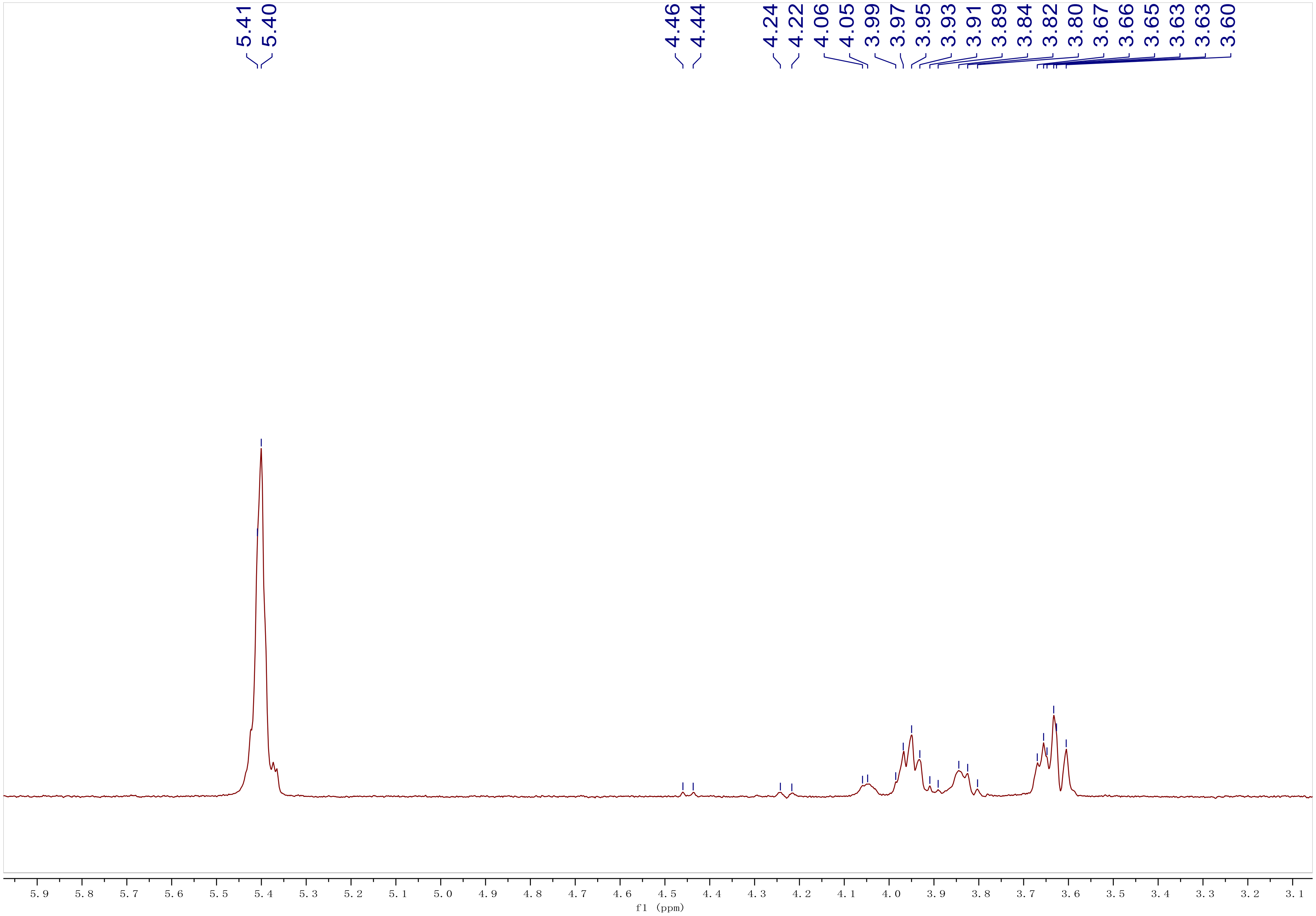

Figure S116. 1D-selective TOCSY spectrum of compound **15** (500 MHz, D_2O, excitation at δ 5.40, H-**B**1, **C**1, and **D**1).

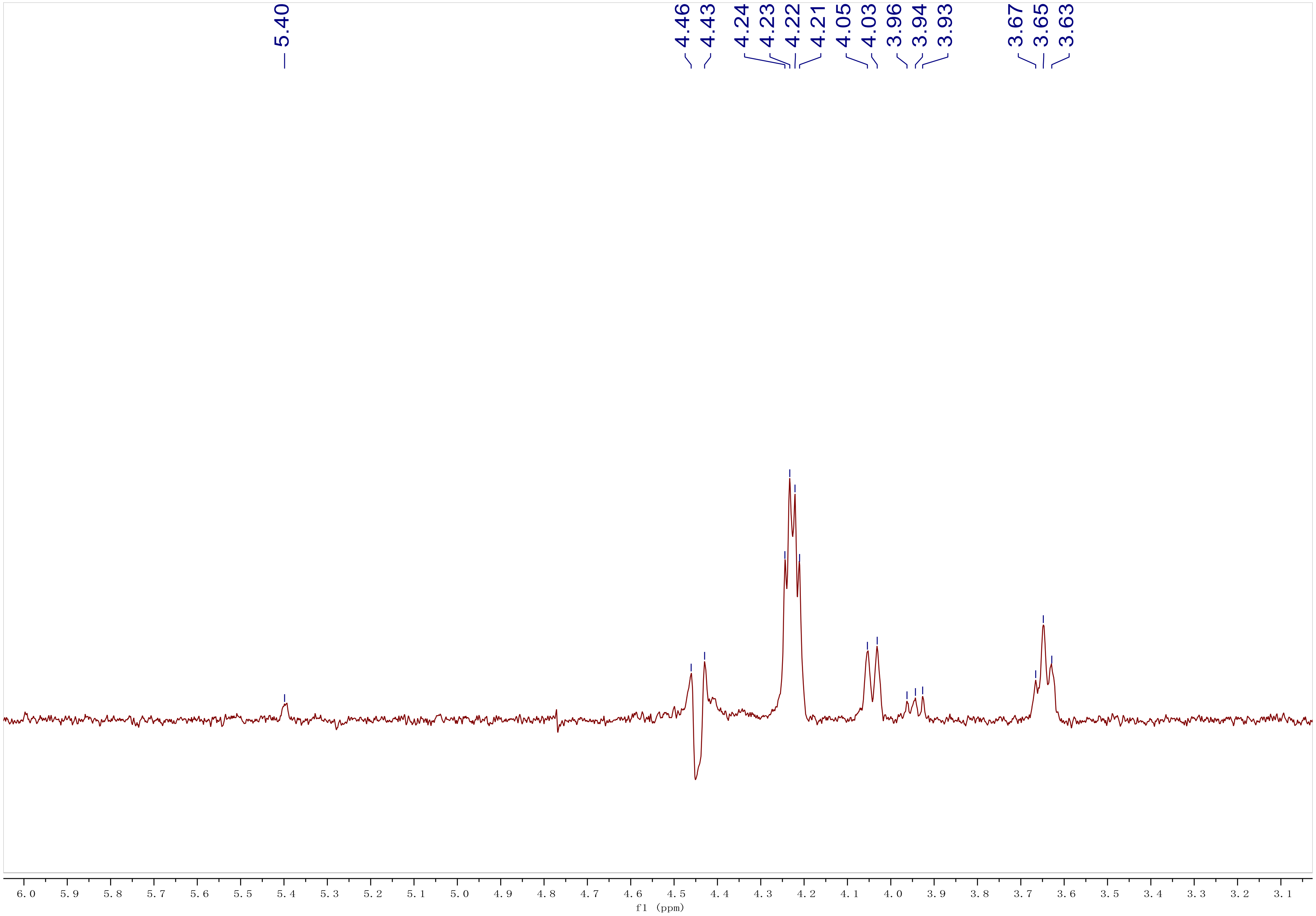

Figure S117. 1D-selective TOCSY spectrum of compound **15** (500 MHz, D_2O, excitation at δ 4.45, H-**D**6a).

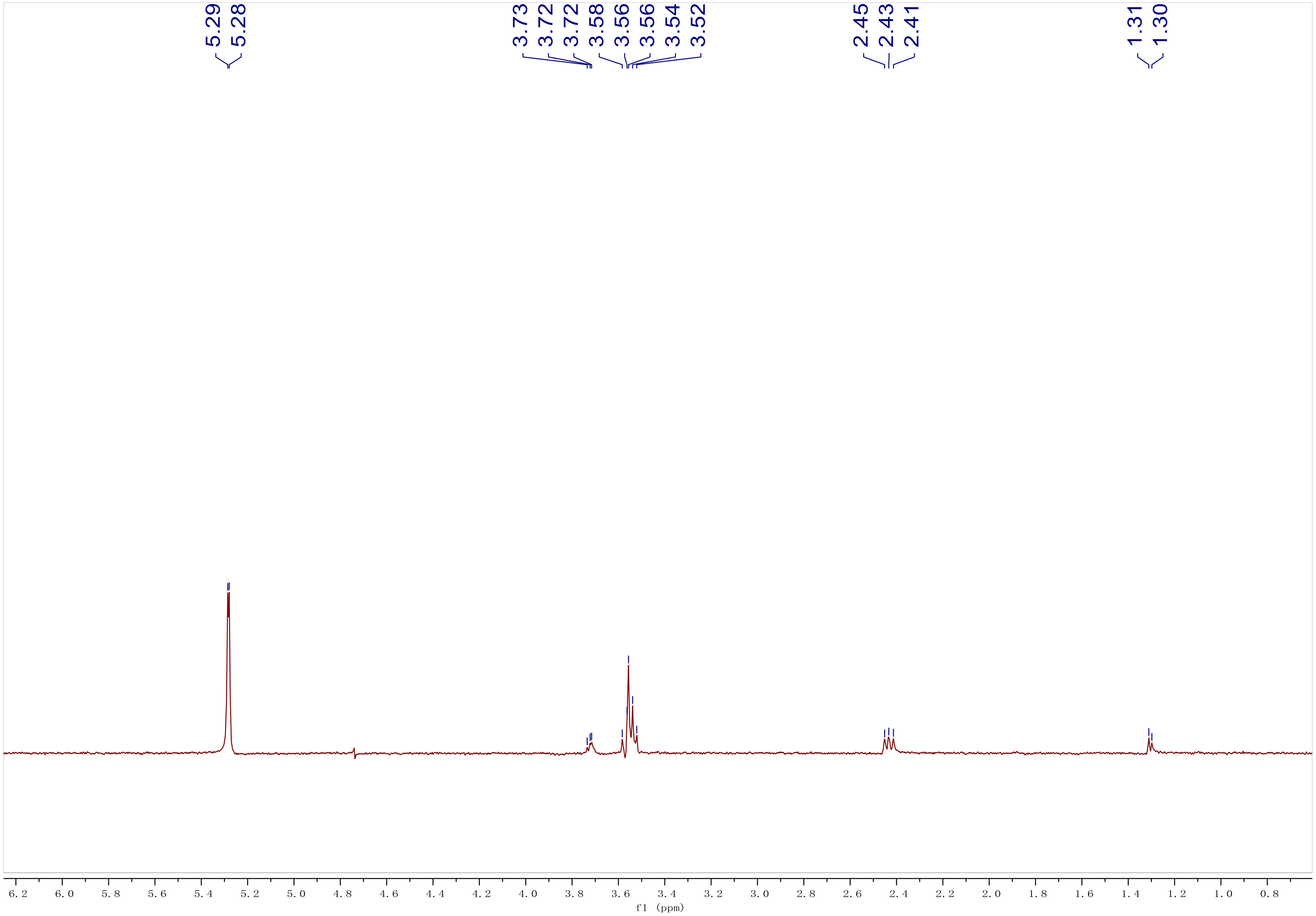

Figure S118. 1D-selective TOCSY spectrum of compound **15** (500 MHz, D_2O, excitation at δ 5.28, H-**E**1).

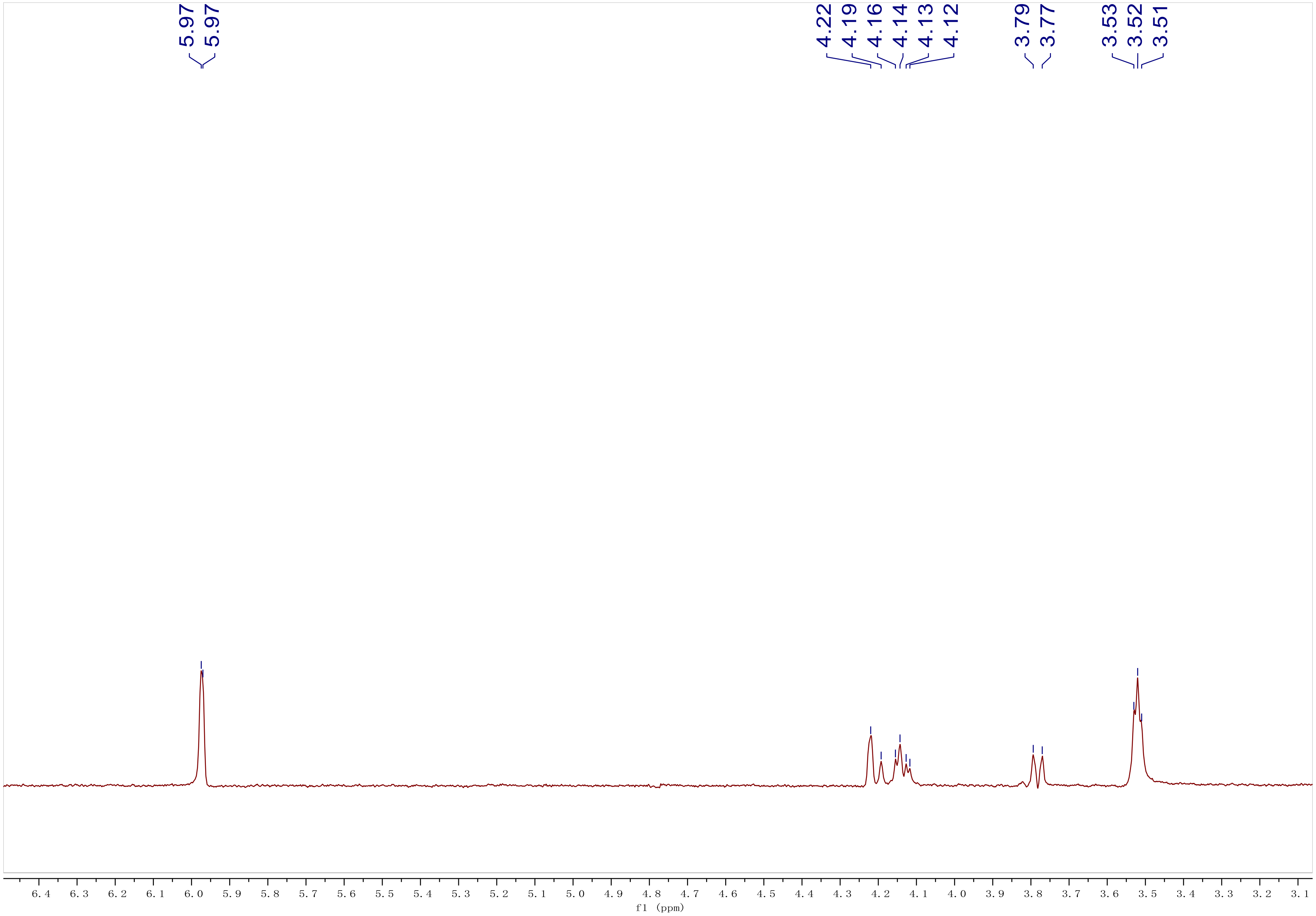

Figure S119. 1D-selective TOCSY spectrum of compound **15** (500 MHz, D_2O, excitation at δ 5.97, H-**F**1).

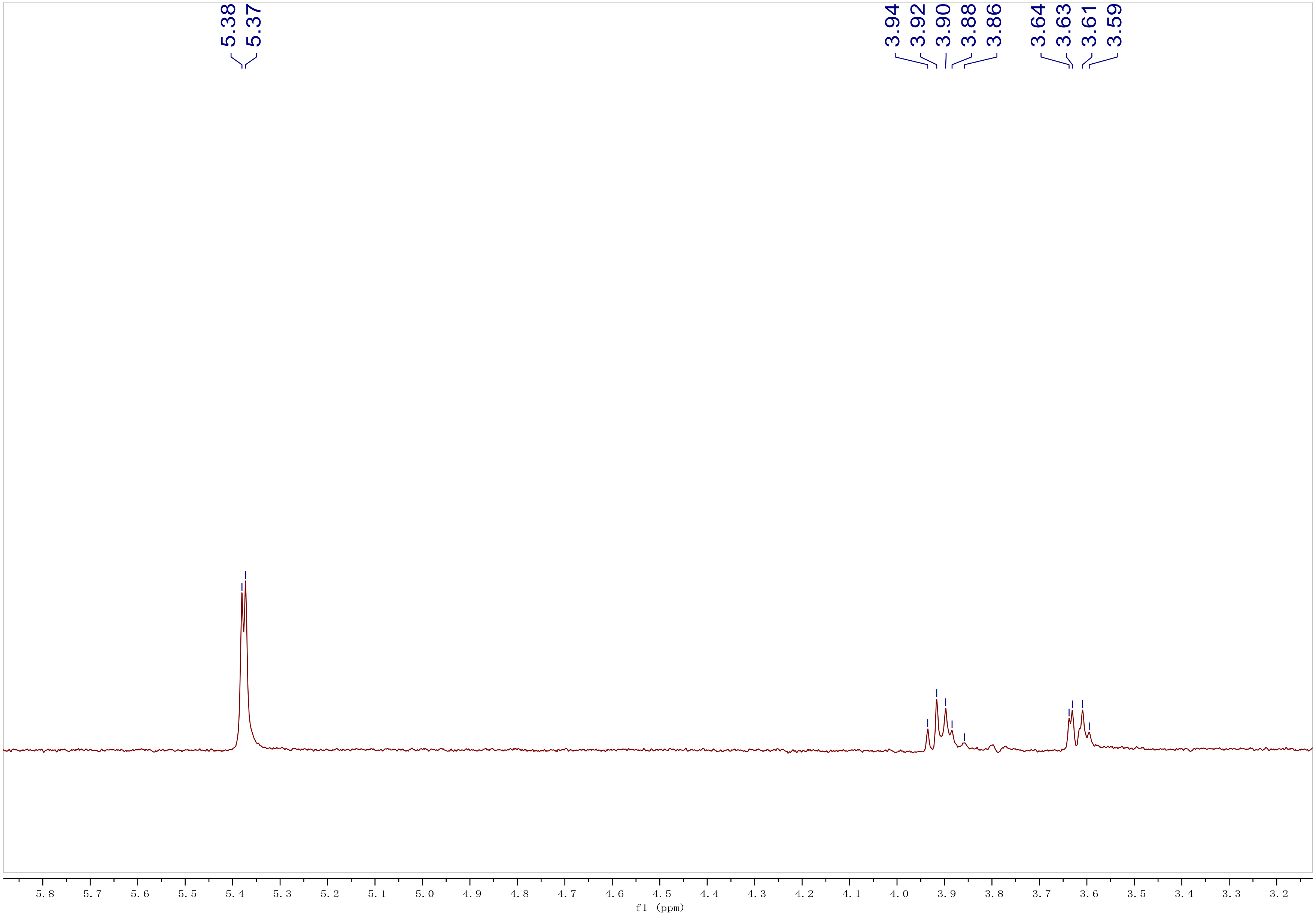

Figure S120. 1D-selective TOCSY spectrum of compound **15** (500 MHz, D_2O, excitation at δ 5.37, H-**G**1).

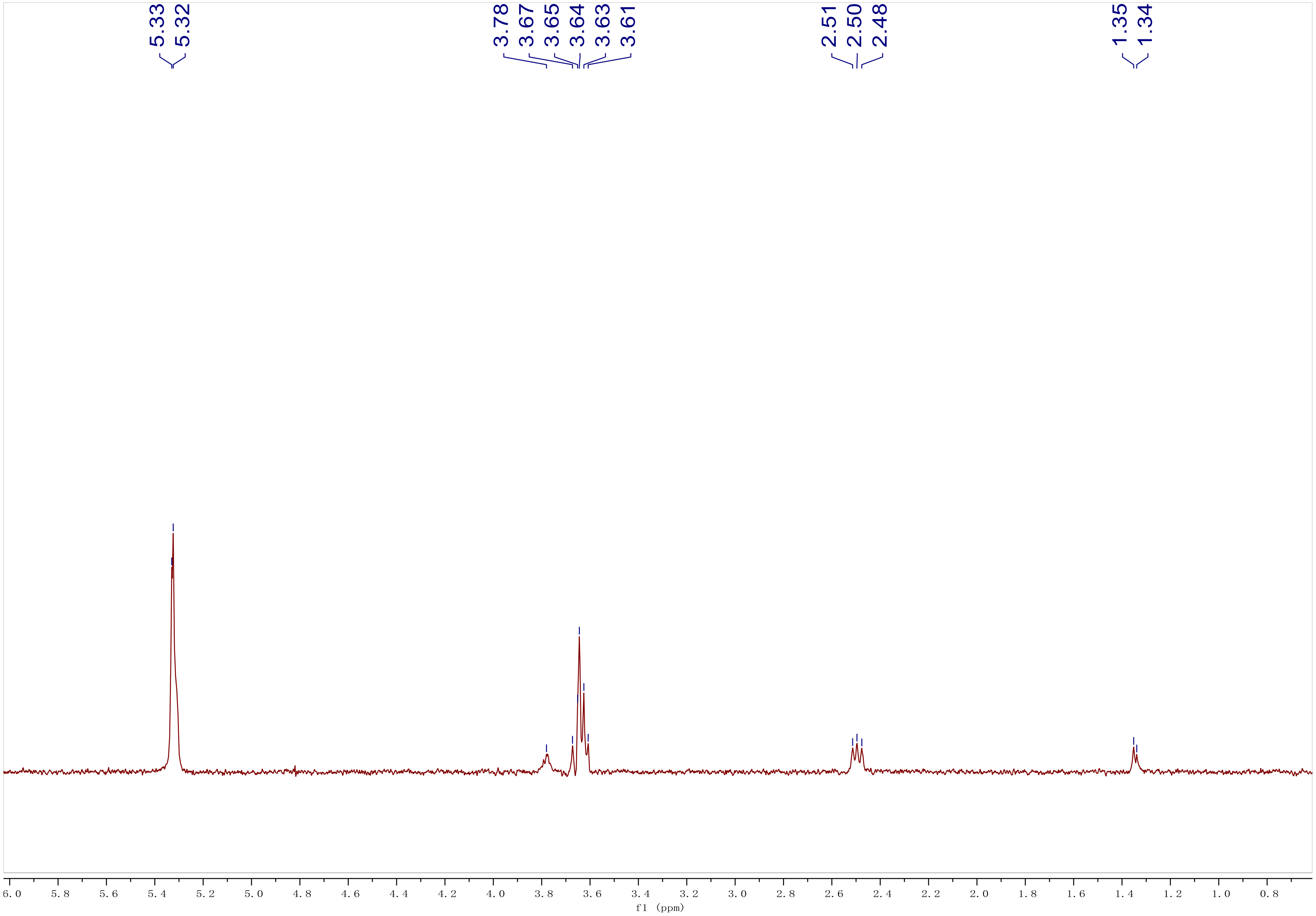

Figure S121. 1D-selective TOCSY spectrum of compound **15** (500 MHz, D_2O, excitation at δ 5.32, H-**H**1).

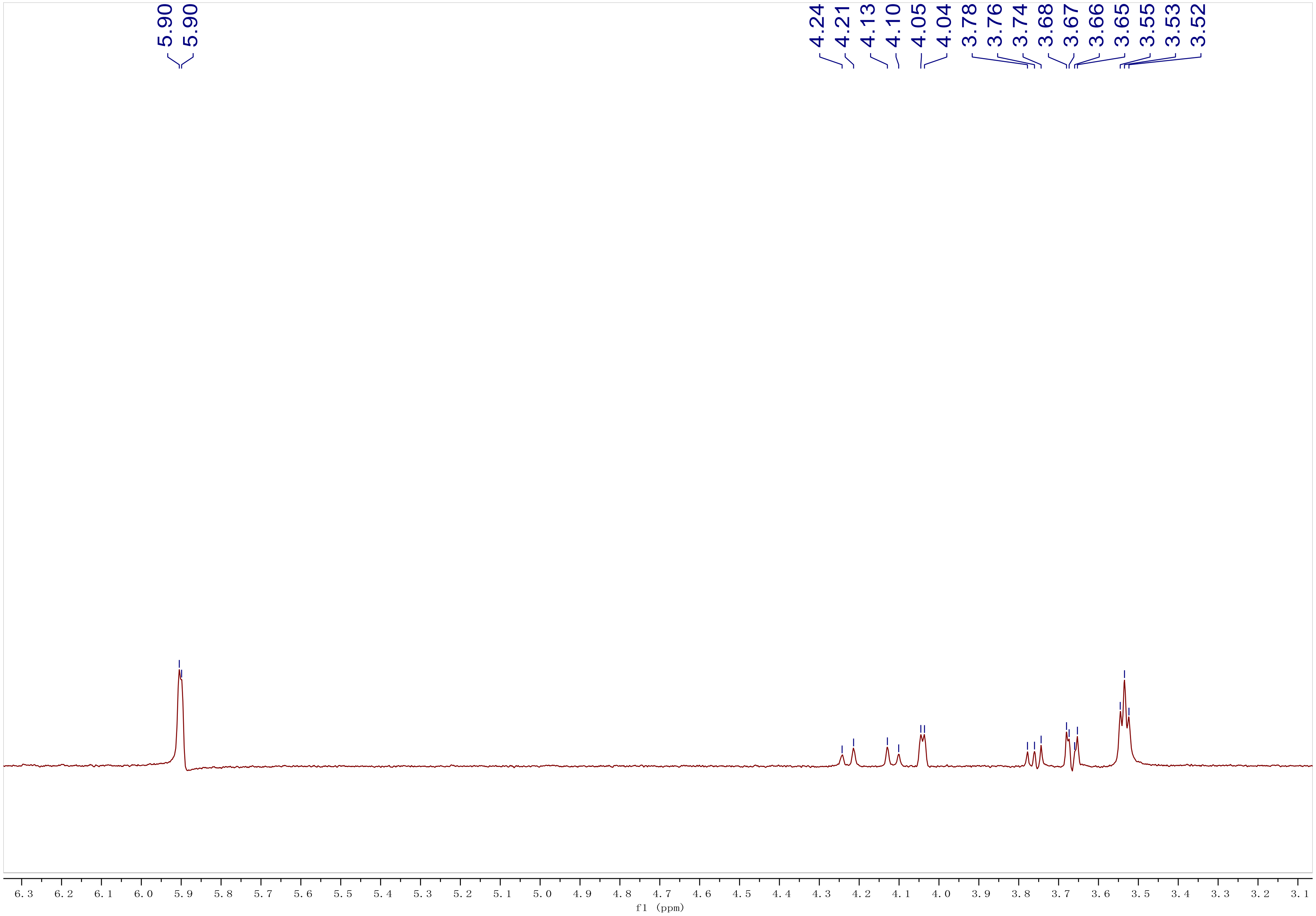

Figure S122. 1D-selective TOCSY spectrum of compound **15** (500 MHz, D_2O, excitation at δ 5.90, H-**I**1).

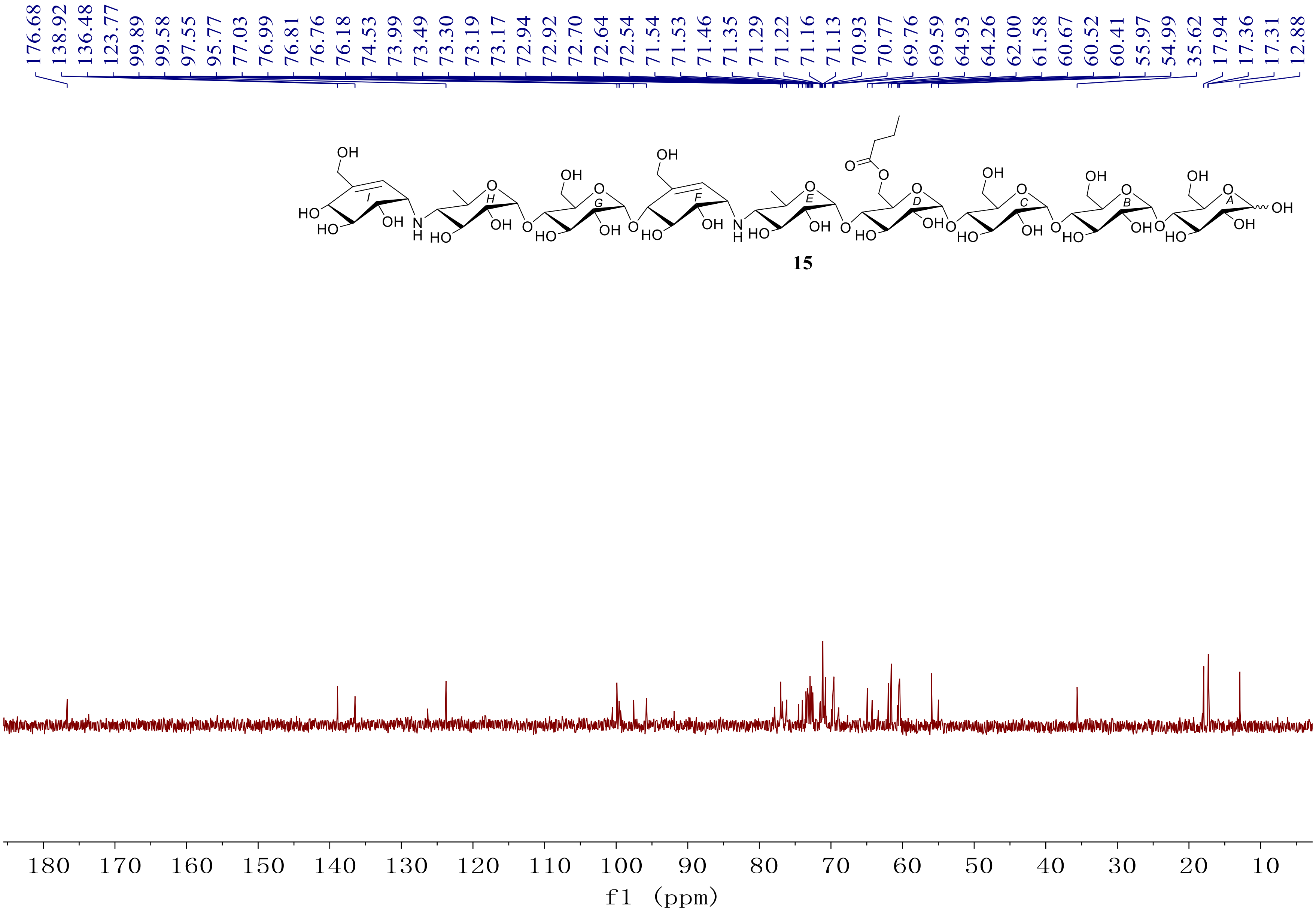

Figure S123. ^{13}C NMR spectrum of compound **15** (125 MHz, D_2O).

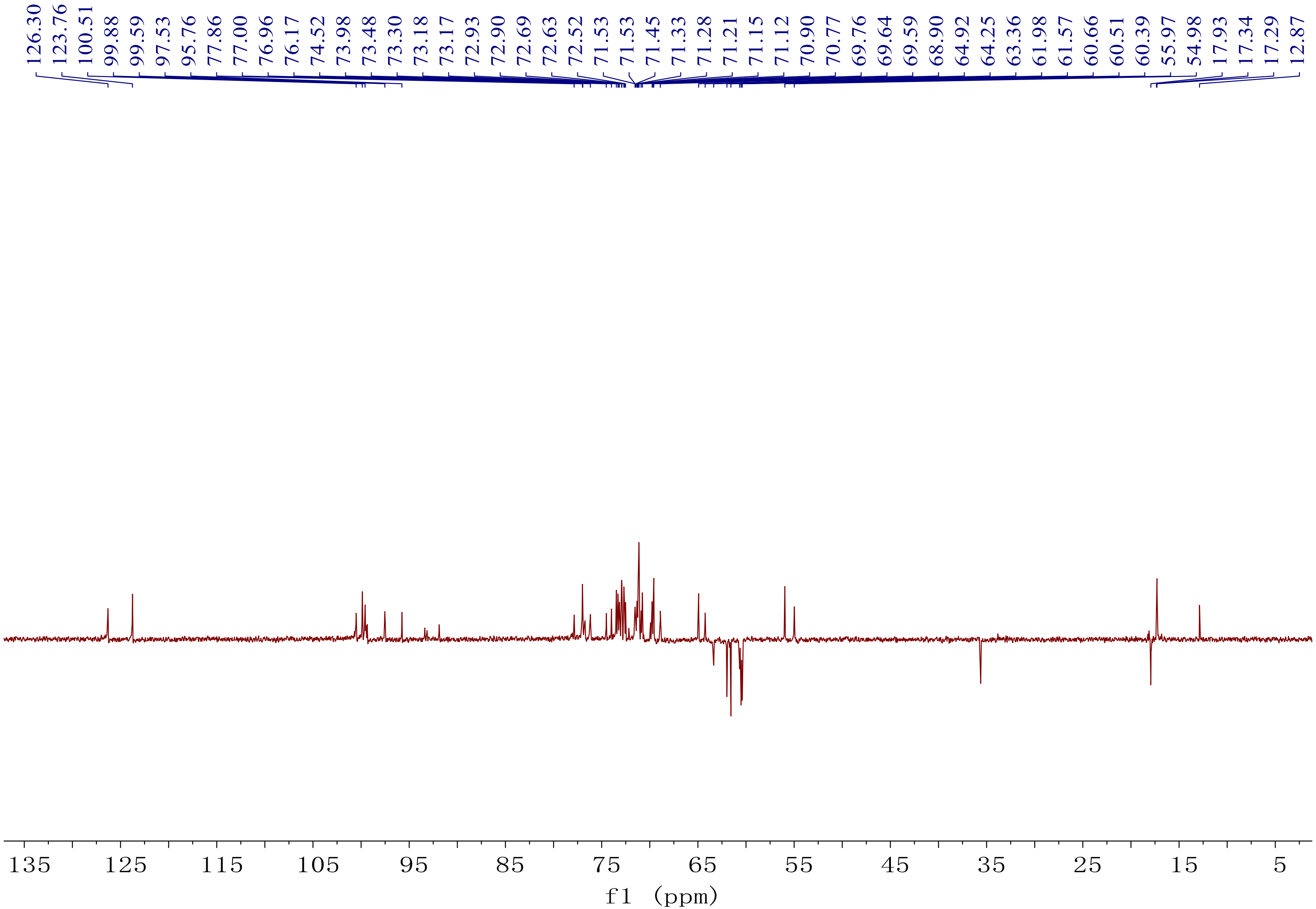

Figure S124. DEPT-135 spectrum of compound **15** (125 MHz, D_2O).

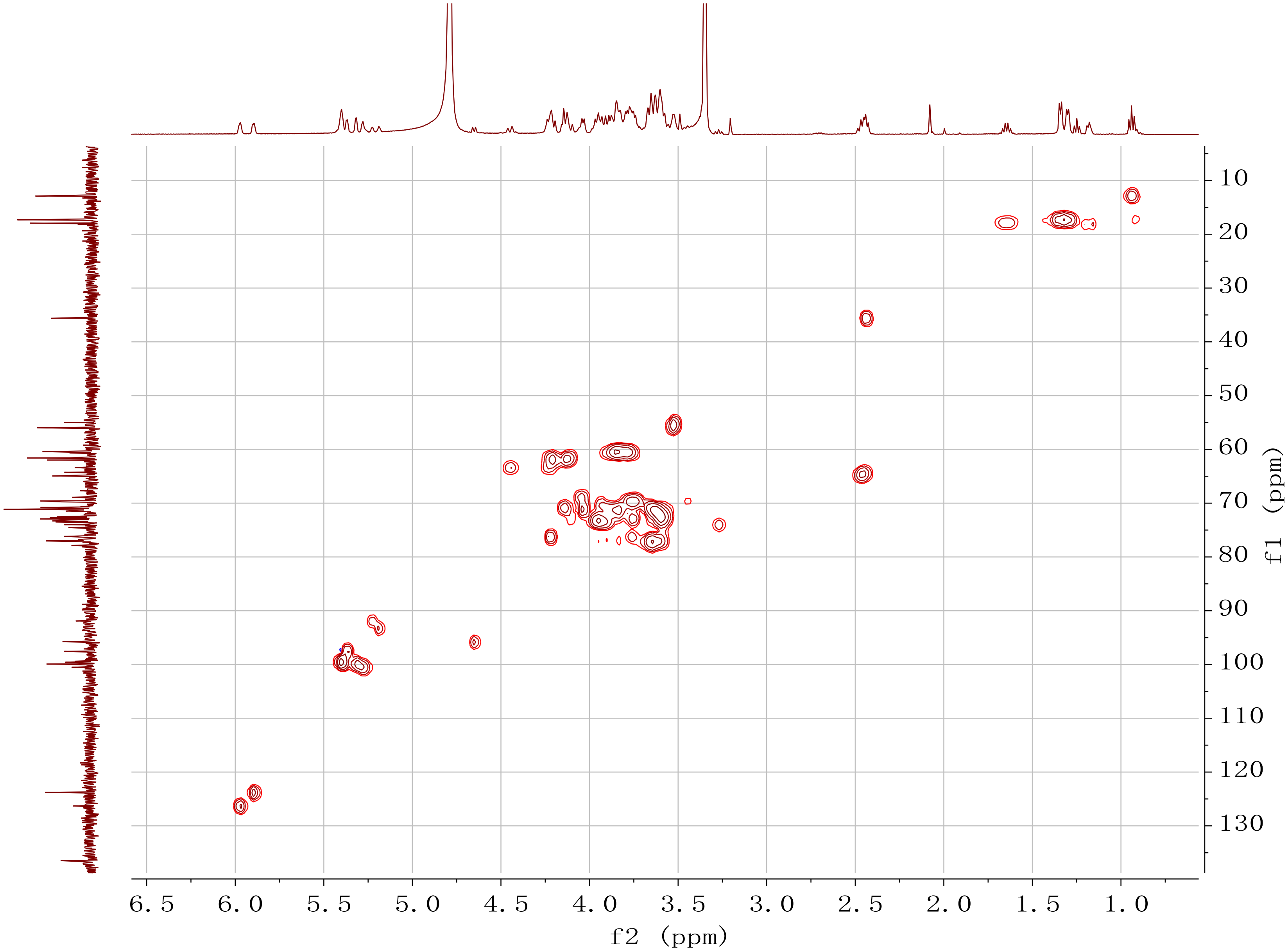

Figure S125. HSQC spectrum of compound **15** (500 MHz, D_2O).

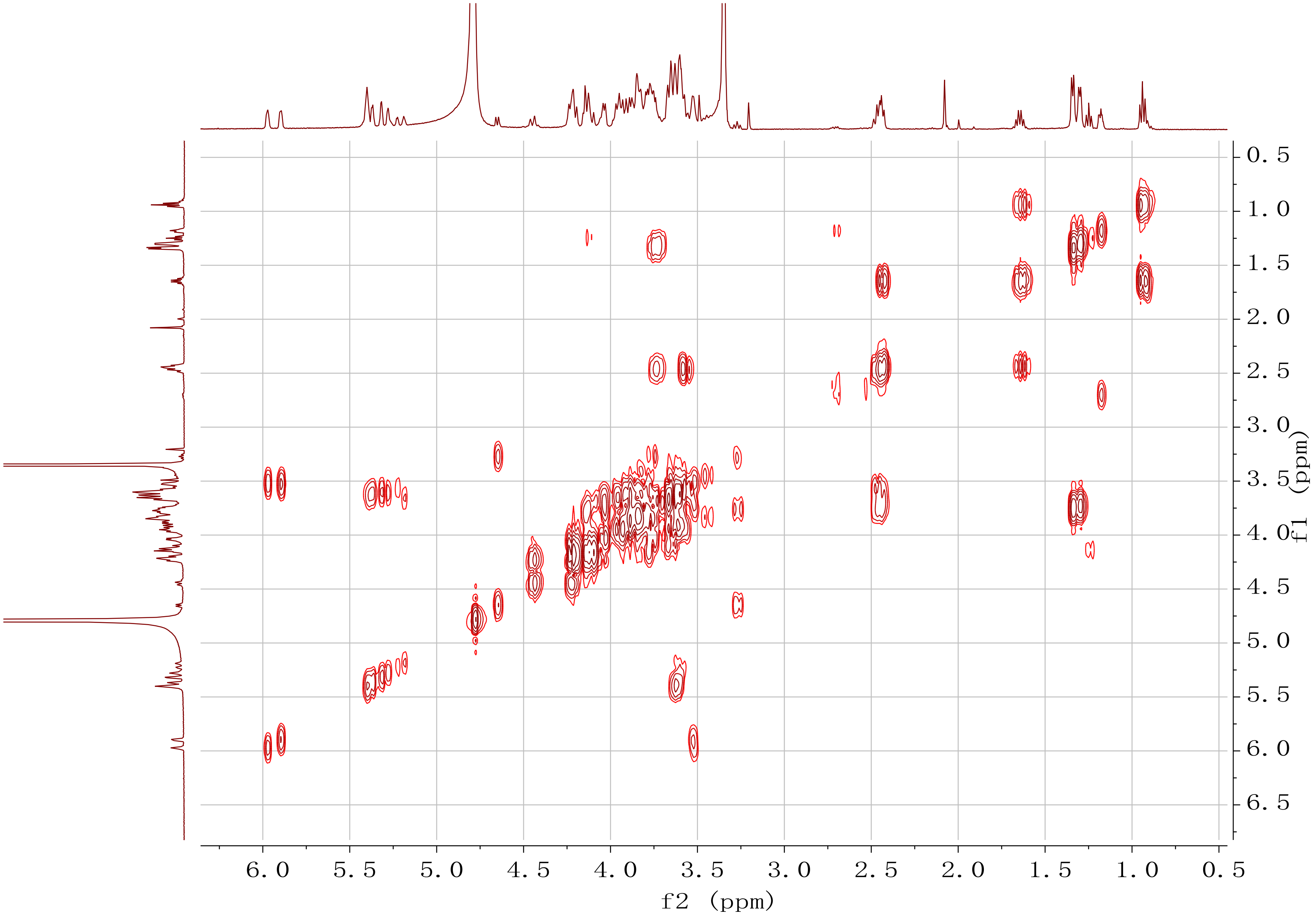

Figure S126. ^1H-^1H COSY spectrum of compound **15** (500 MHz, D_2O).

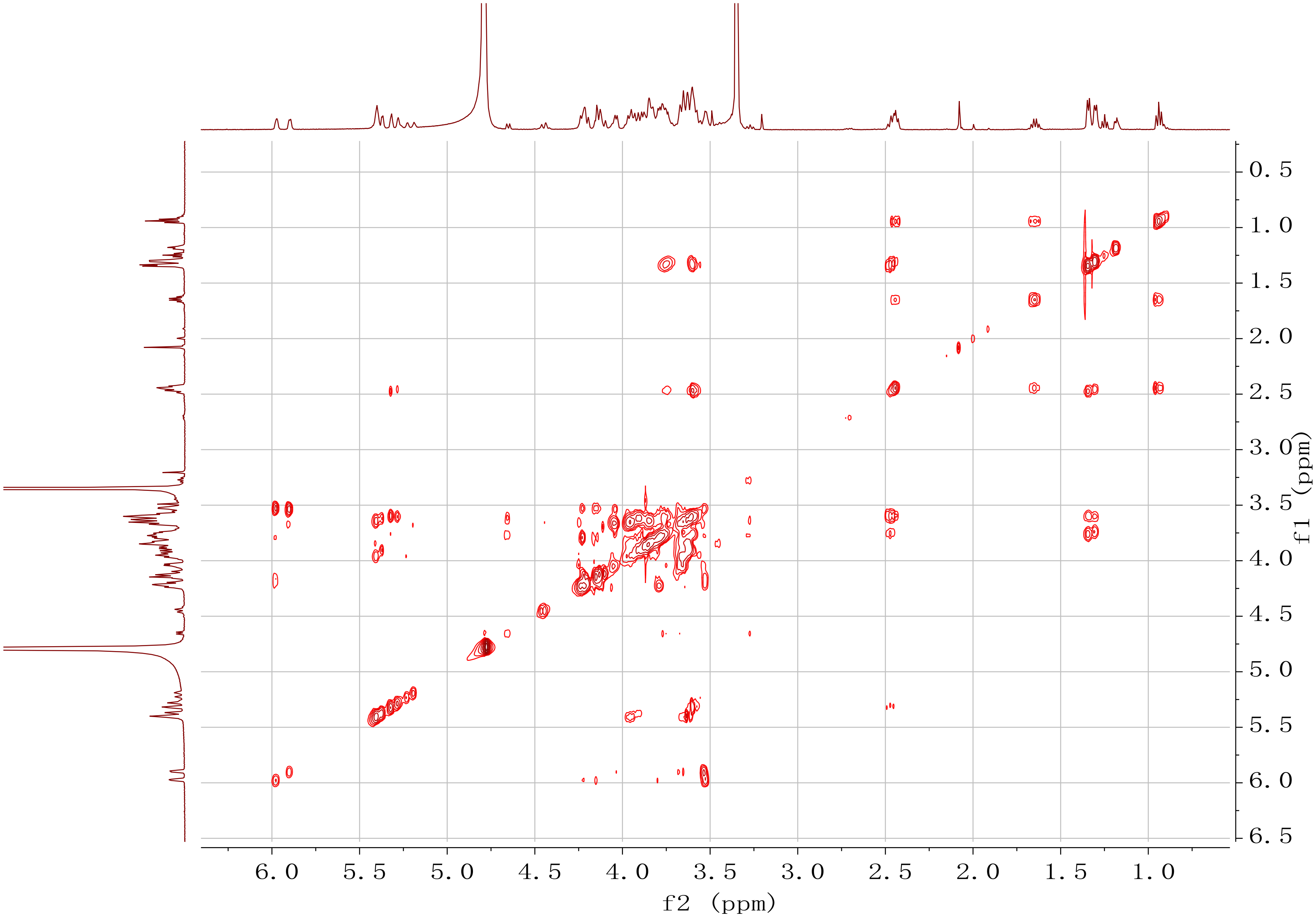

Figure S127. 2D-TOCSY spectrum of compound **15** (500 MHz, D_2O).

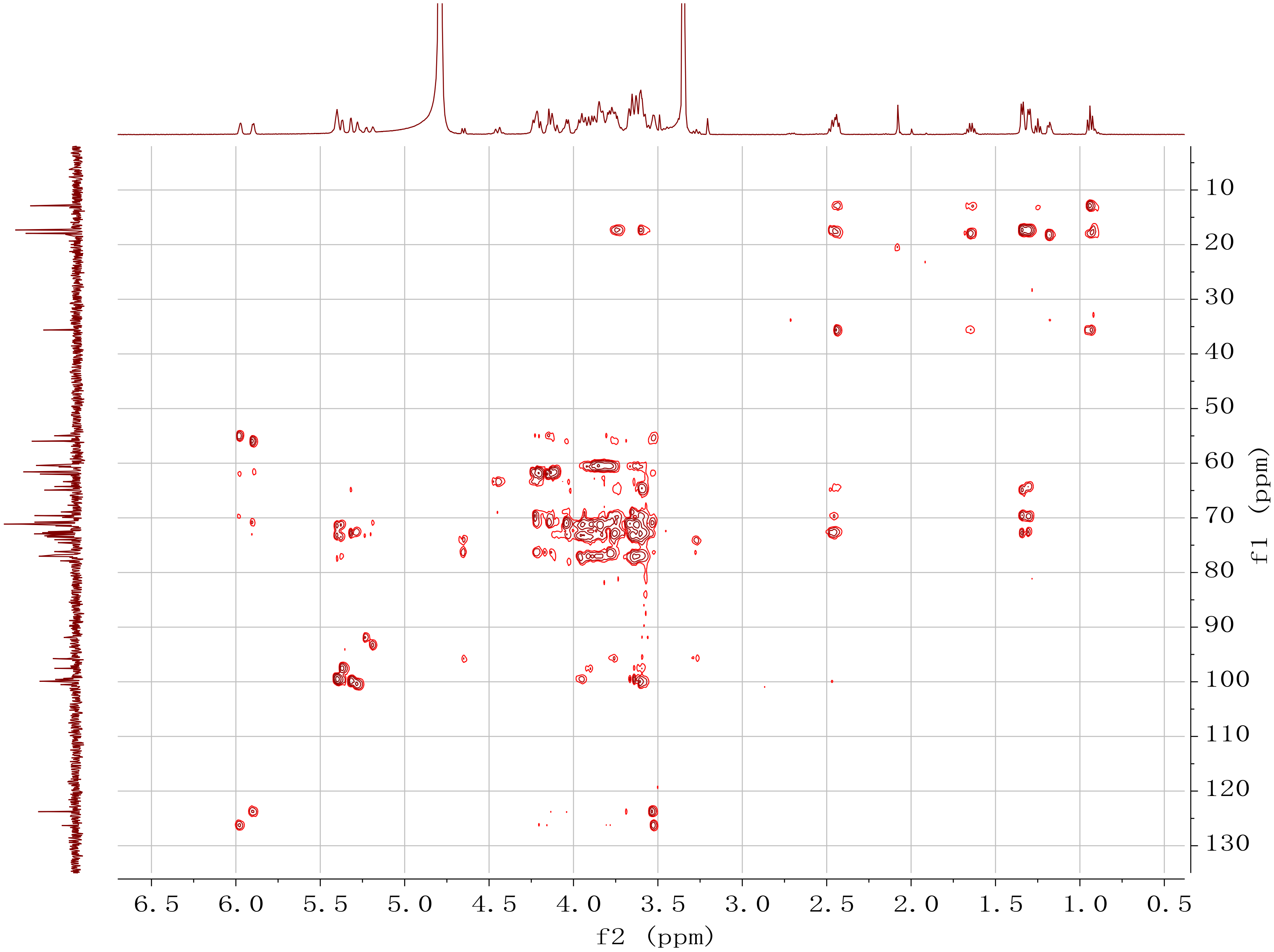

Figure S128. HSQC-TOCSY spectrum of compound **15** (500 MHz, D_2O).

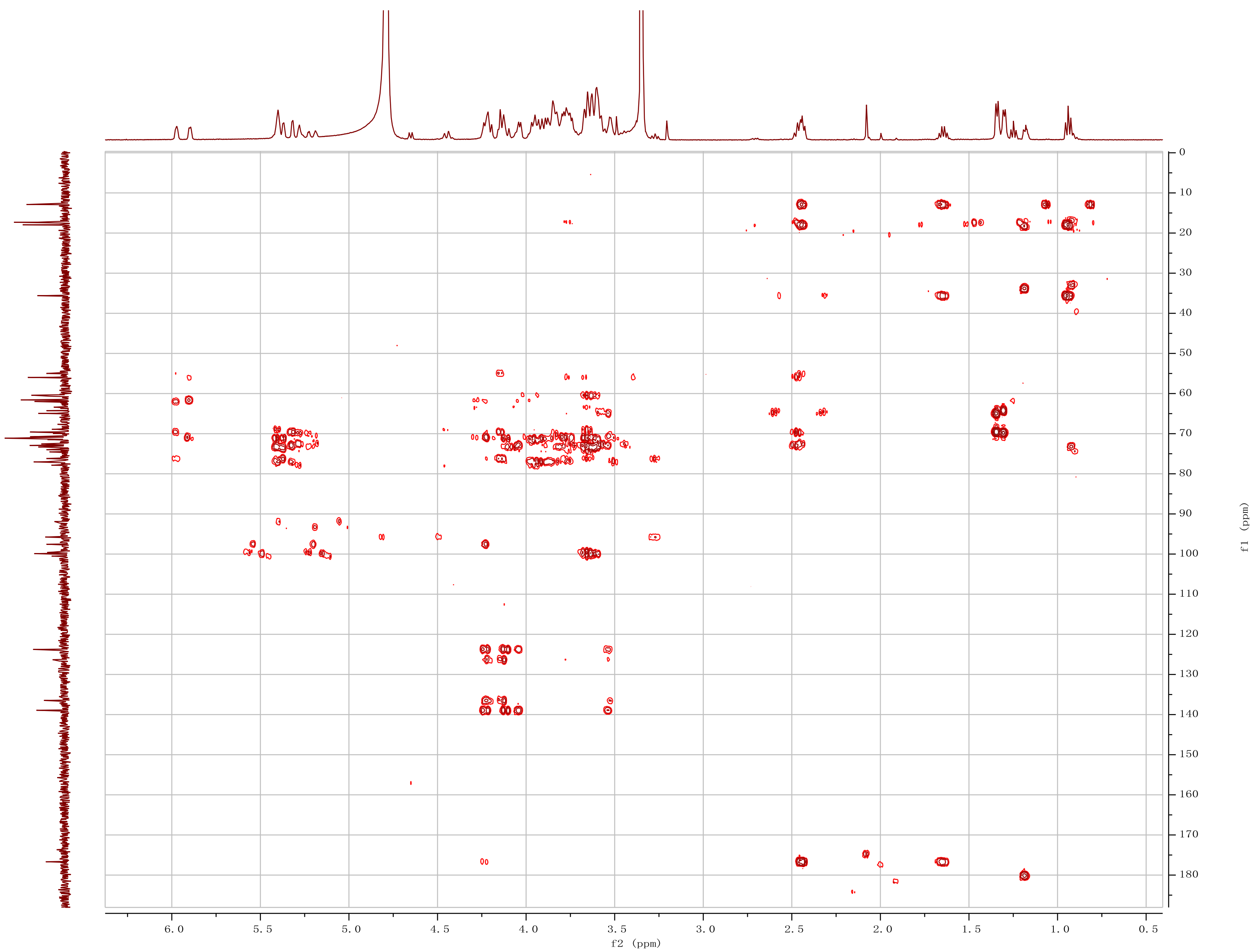

Figure S129. HMBC spectrum of compound **15** (500 MHz, D_2O).

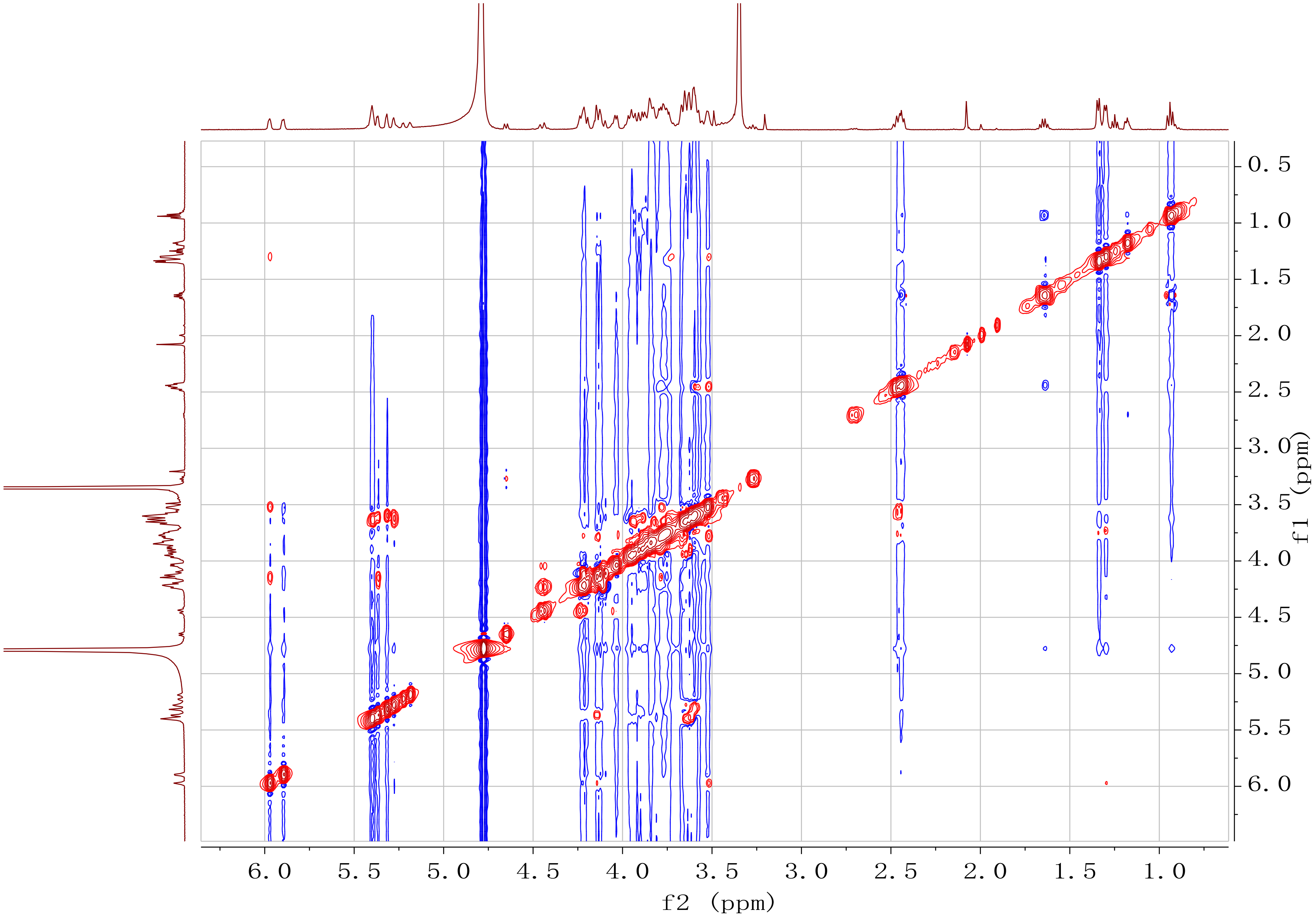

Figure S130. NOESY spectrum of compound **15** (500 MHz, D_2O).

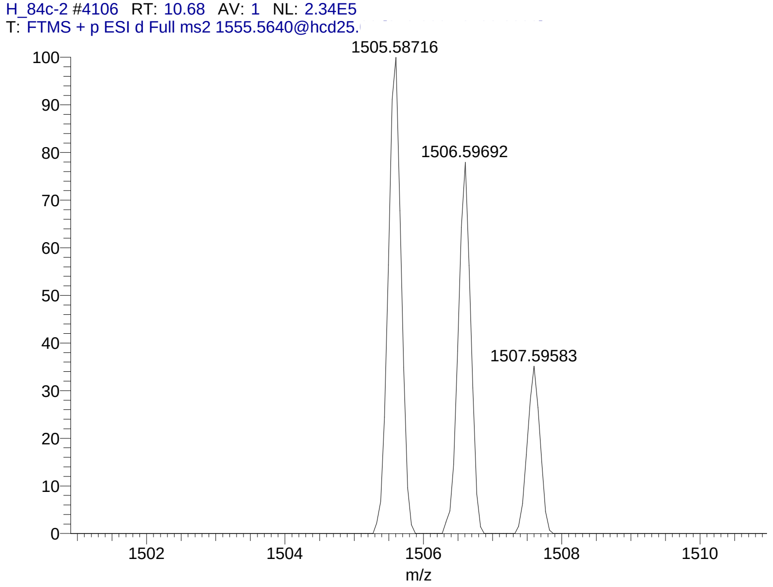

Figure S131. HRESIMS spectrum of compound **15**.

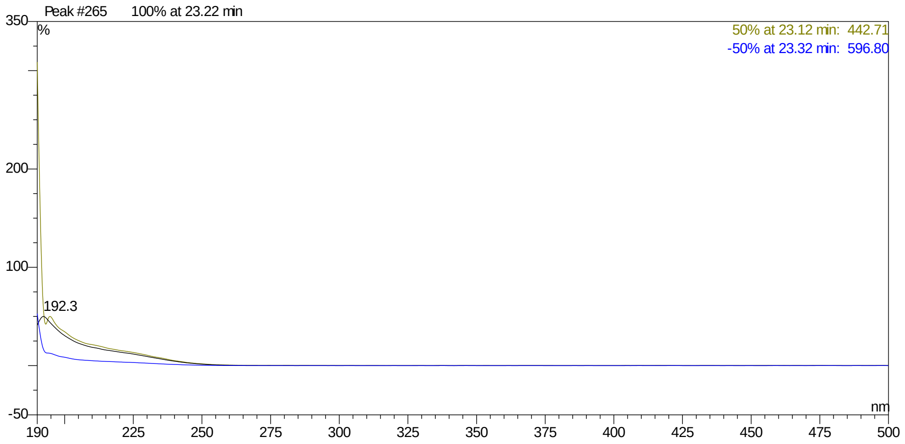

Figure S132. UV spectrum of compound **15**.

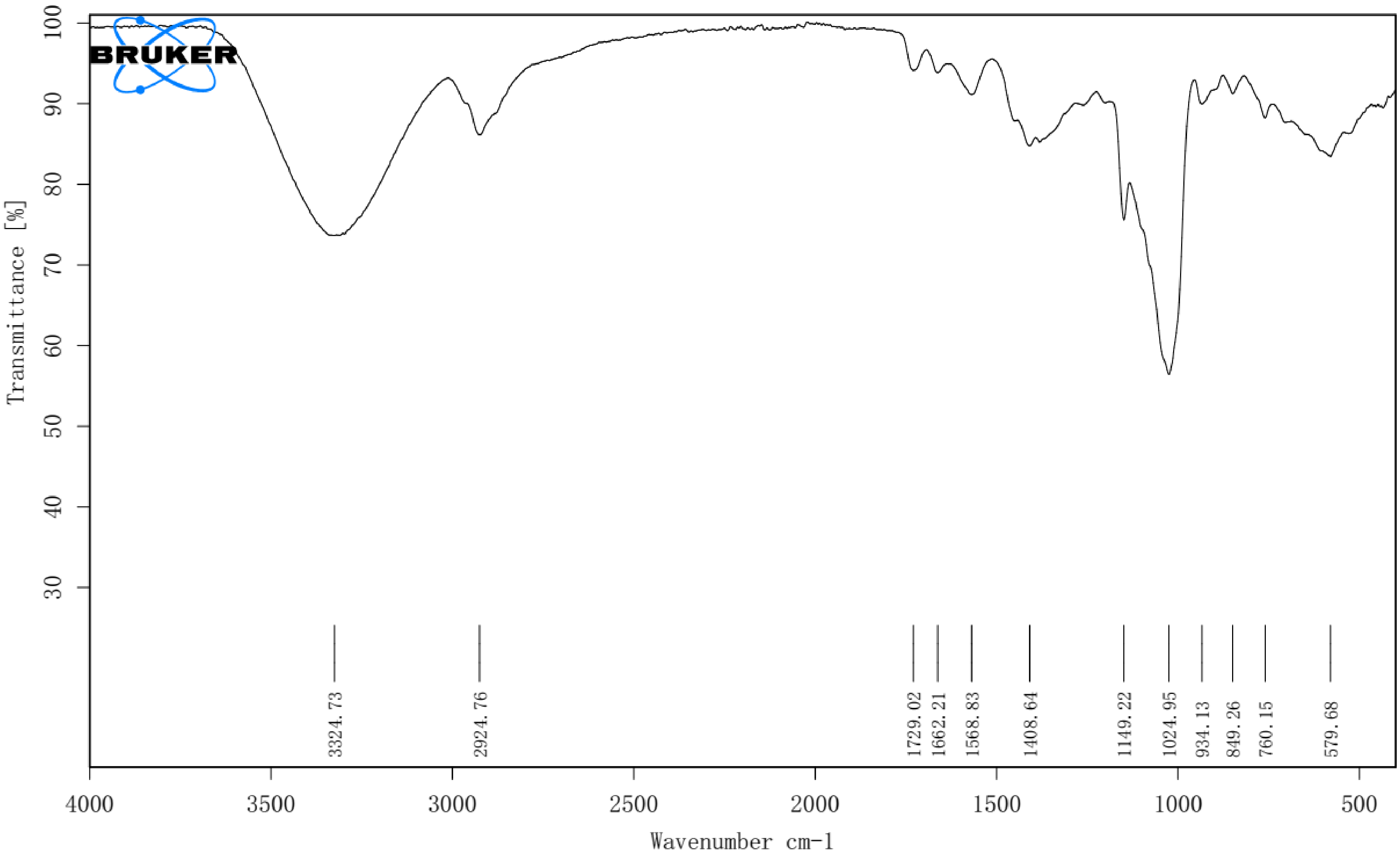

Figure S133. IR spectrum of compound **15**.

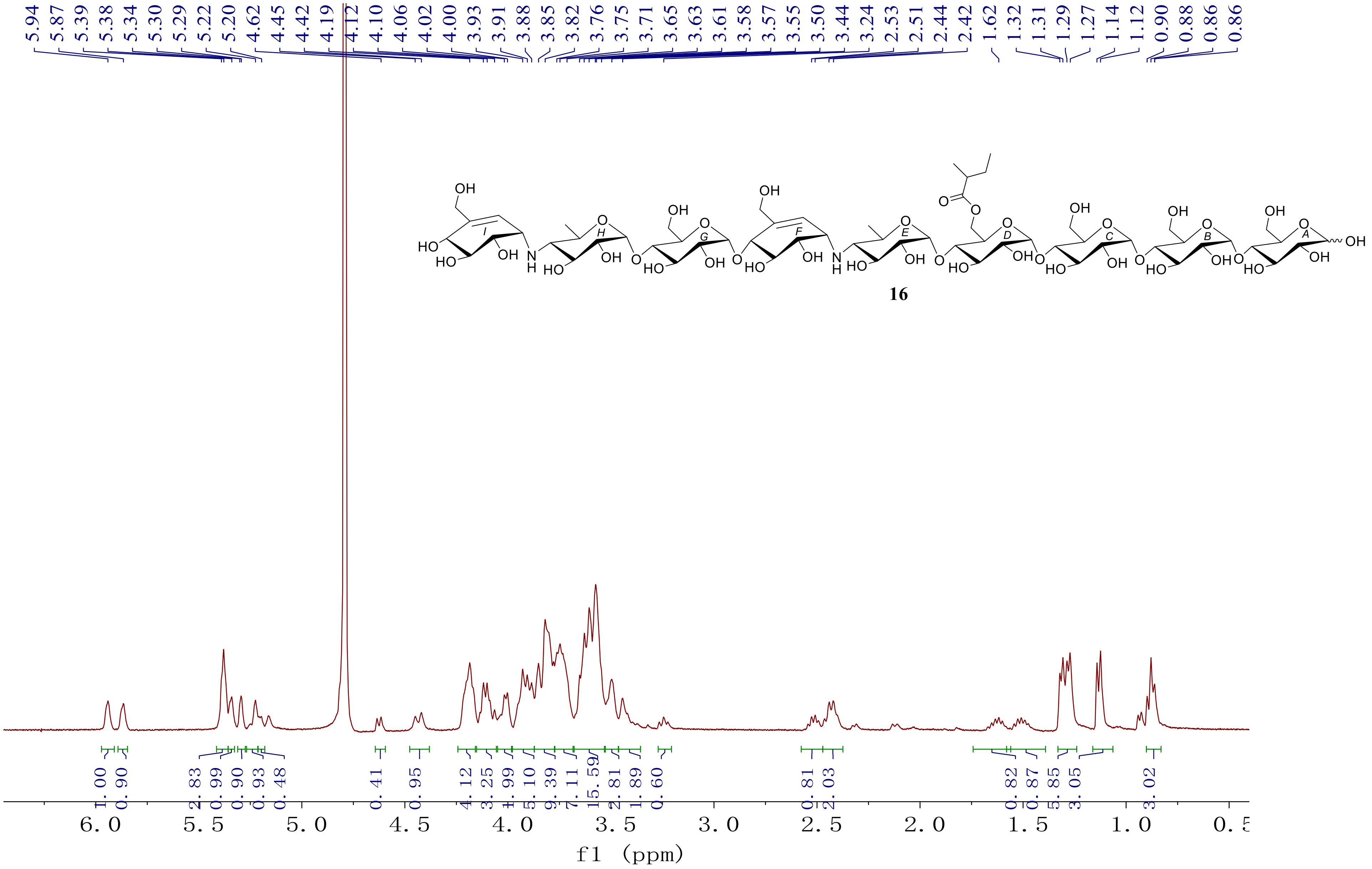

16

Figure S134. ^{1}H NMR spectrum of compound **16** (500 MHz, D_2O).

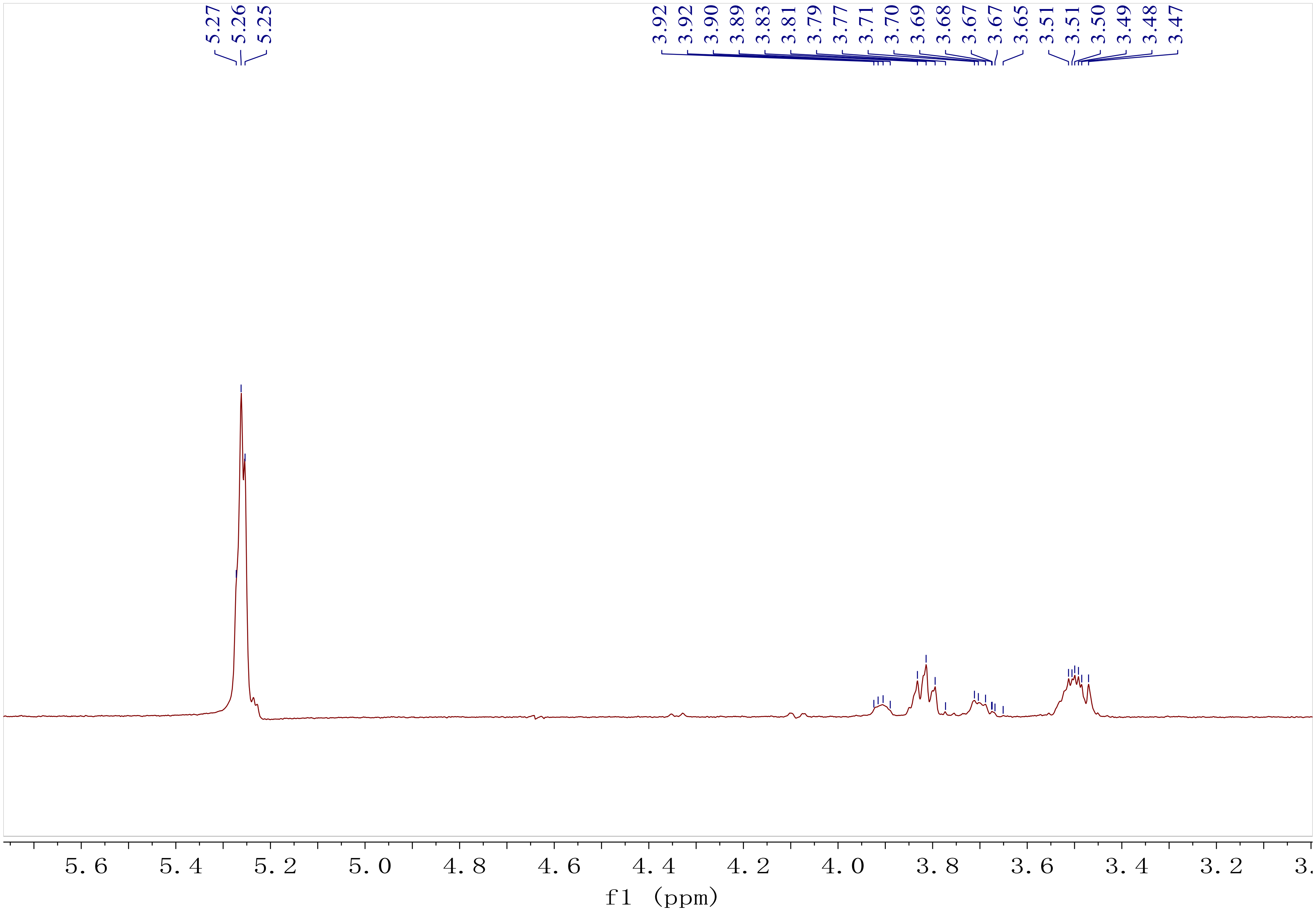

Figure S135. 1D-selective TOCSY spectrum of compound **16** (500 MHz, D_2O, excitation at δ 5.20, H-**A**1α).

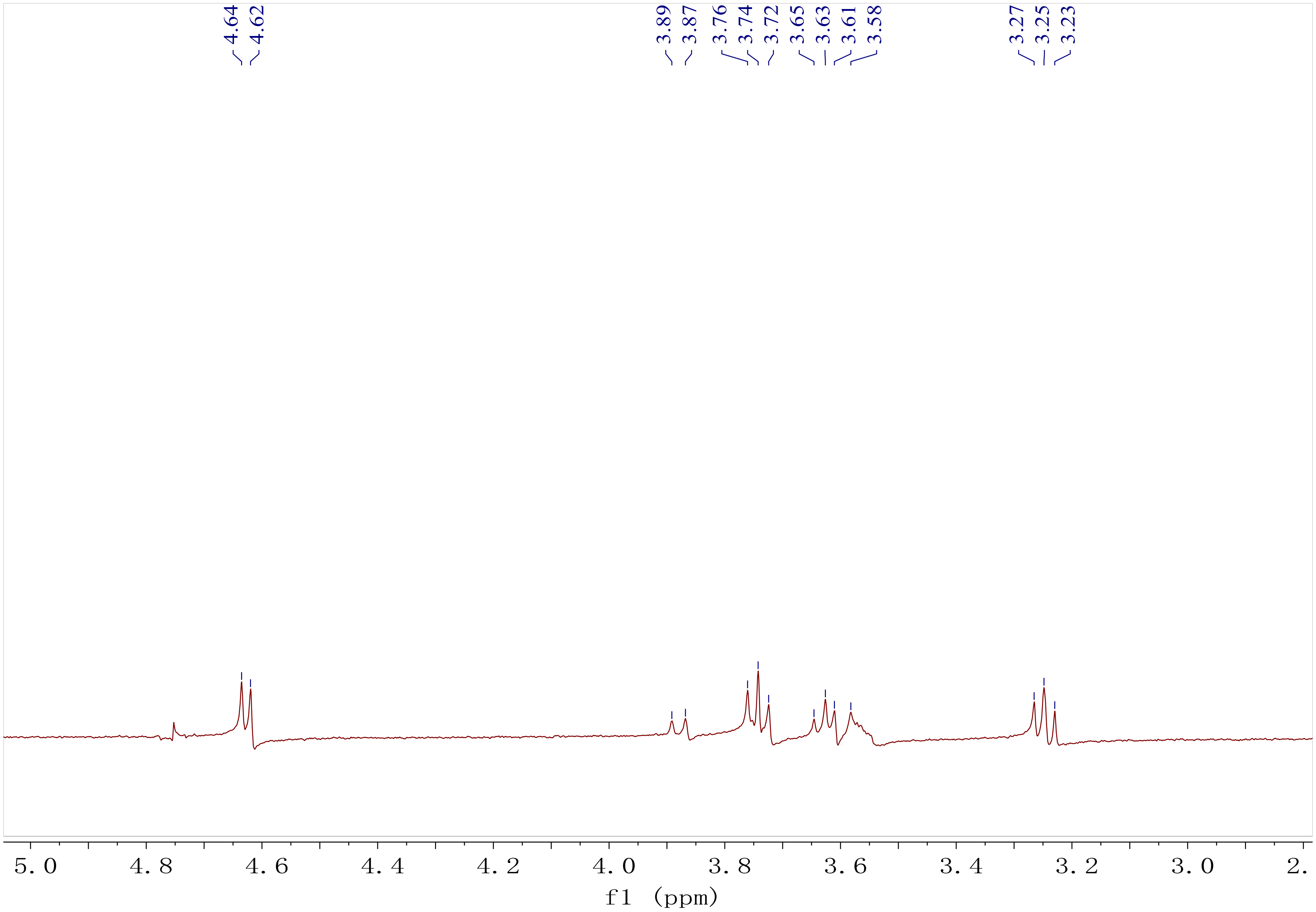

Figure S136. 1D-selective TOCSY spectrum of compound **16** (500 MHz, D_2O, excitation at δ 4.63, H-**A**1β).

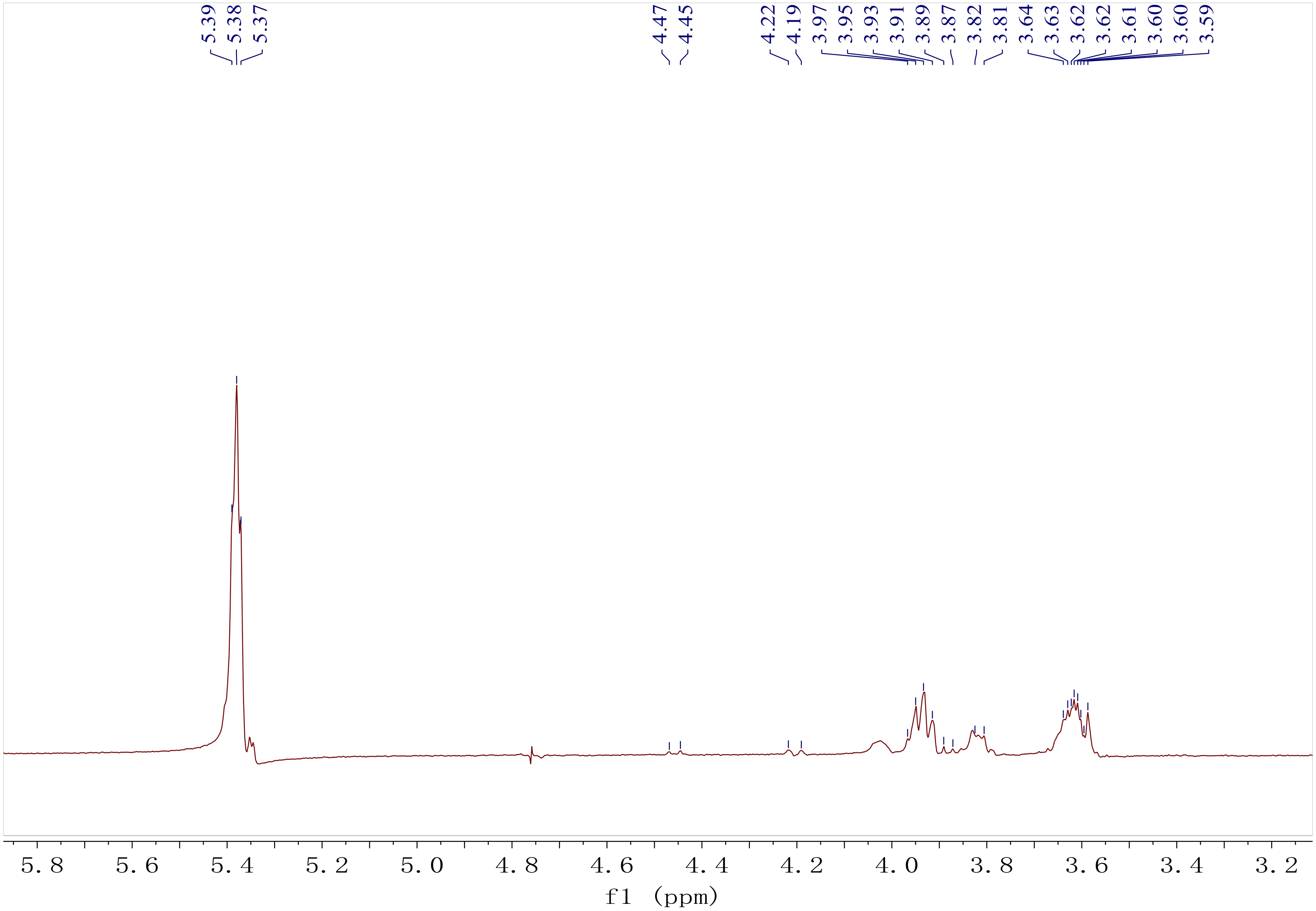

Figure S137. 1D-selective TOCSY spectrum of compound **16** (500 MHz, D_2O, excitation at δ 5.38, H-**B**1, H-**C**1, H-**D**1).

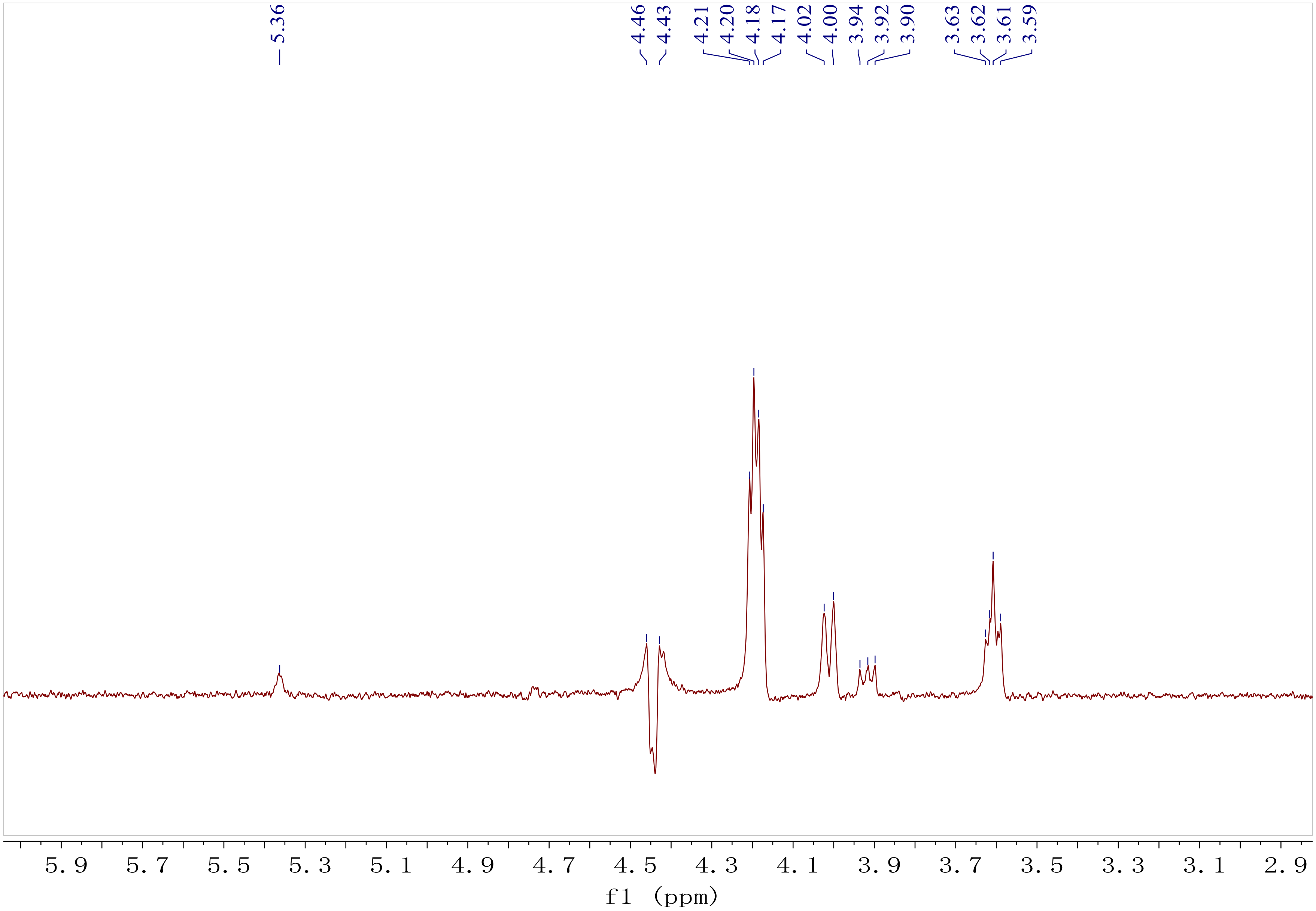

Figure S138. 1D-selective TOCSY spectrum of compound **16** (500 MHz, D_2O, excitation at δ 4.43, H-**D**6a).

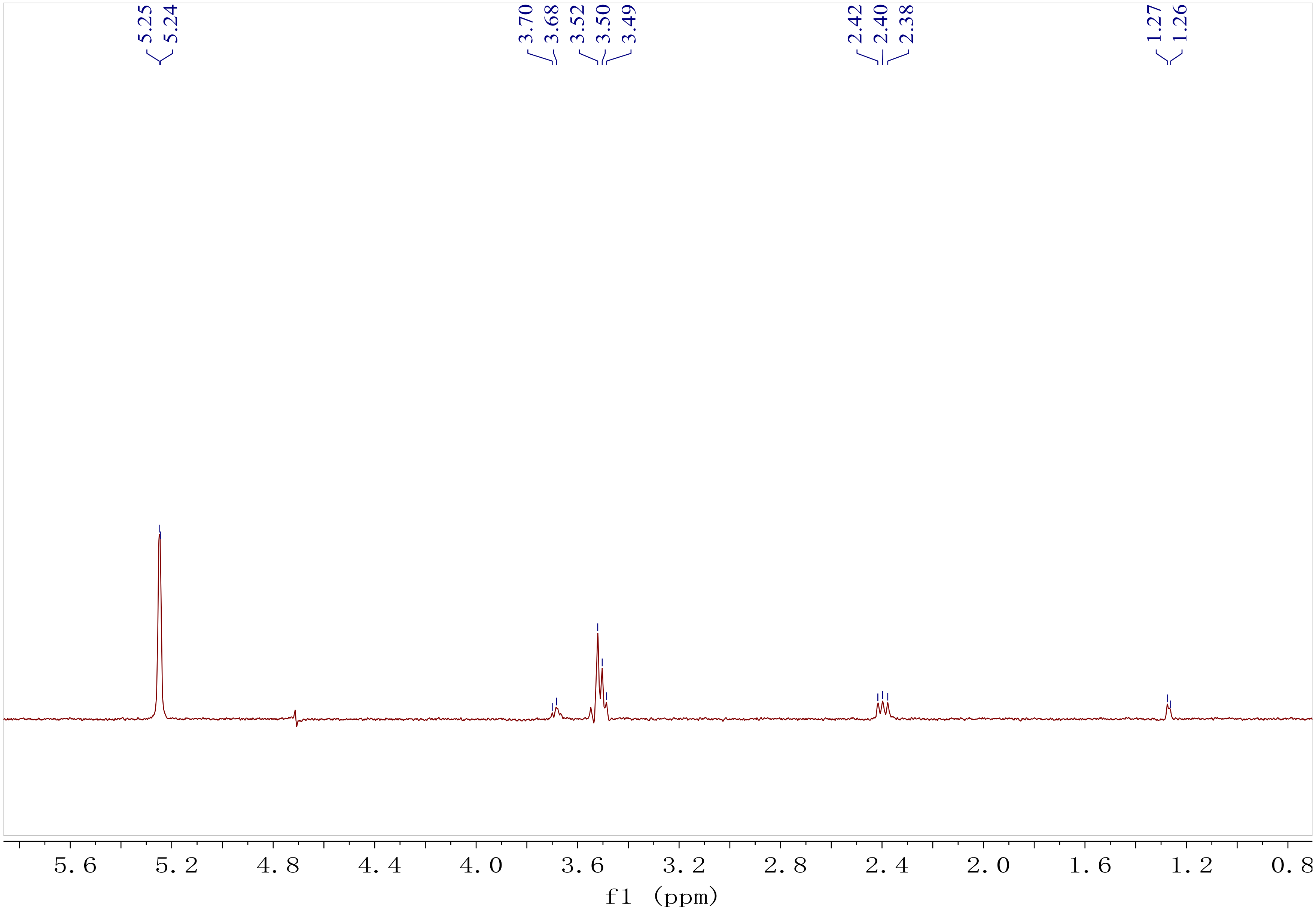

Figure S139. 1D-selective TOCSY spectrum of compound **16** (500 MHz, D_2O, excitation at δ 5.24, H-**E**1).

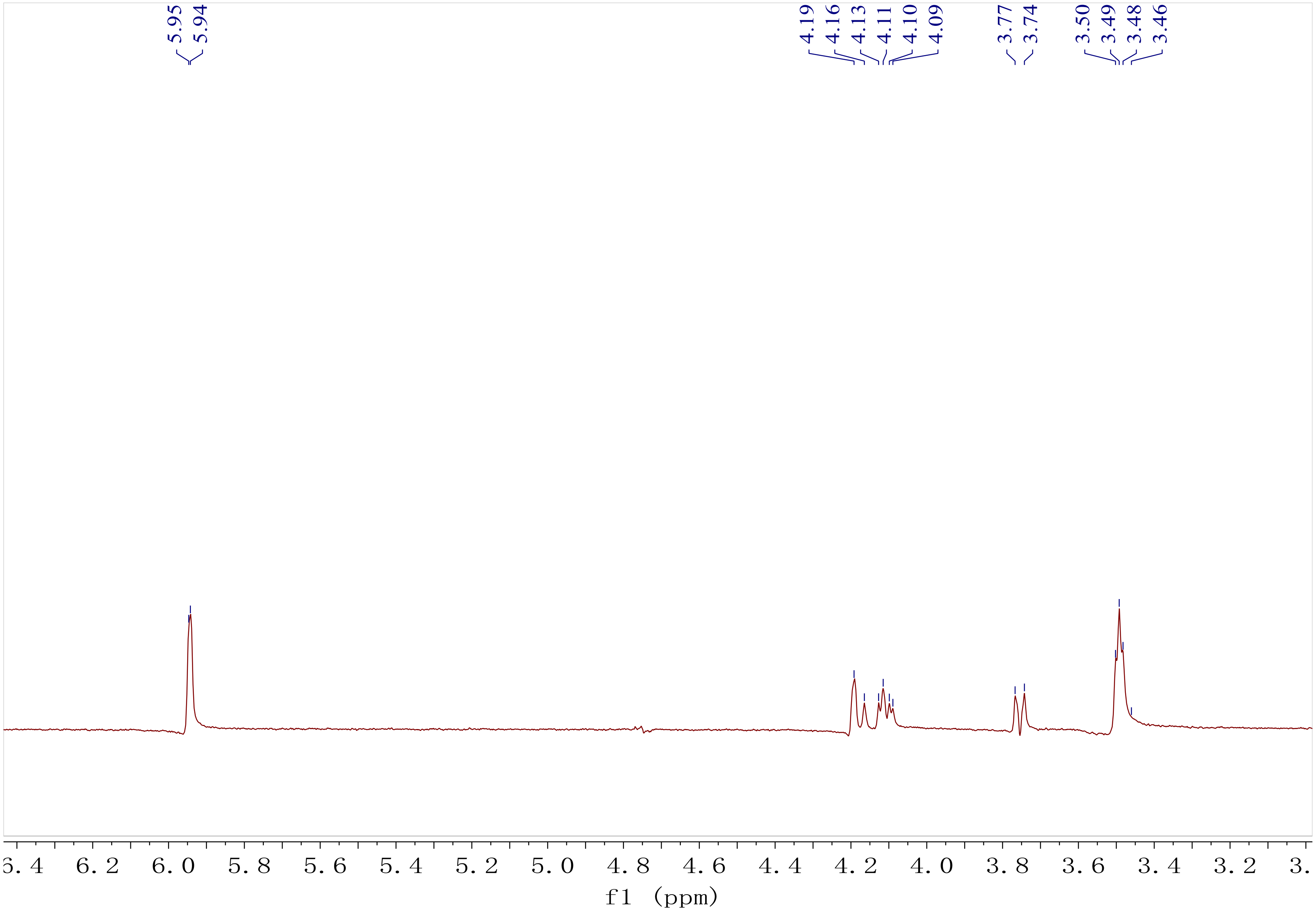

Figure S140. 1D-selective TOCSY spectrum of compound **16** (500 MHz, D_2O, excitation at δ 5.94, H-**F**7).

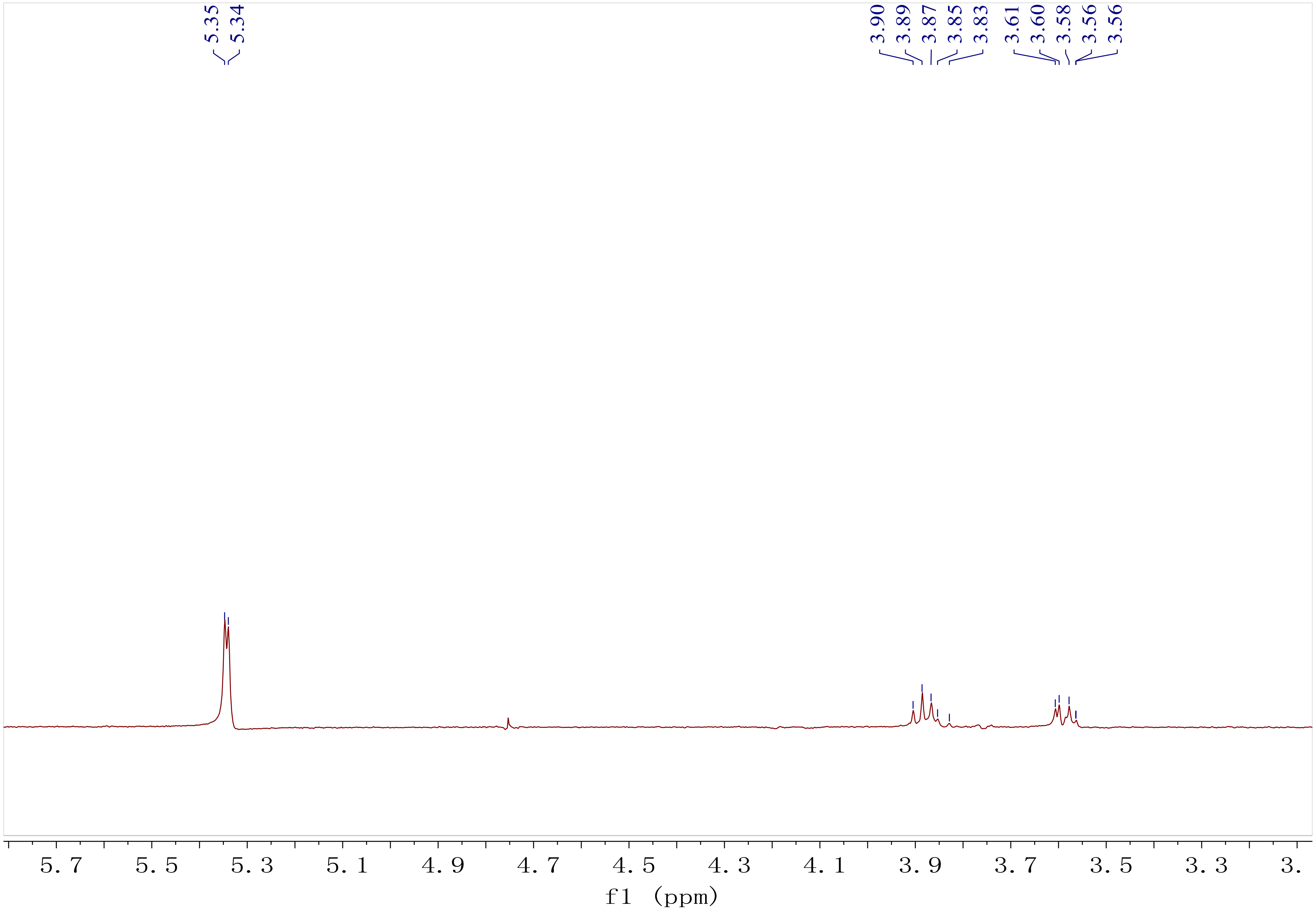

Figure S141. 1D-selective TOCSY spectrum of compound **16** (500 MHz, D_2O, excitation at δ 5.34, H-**G**1).

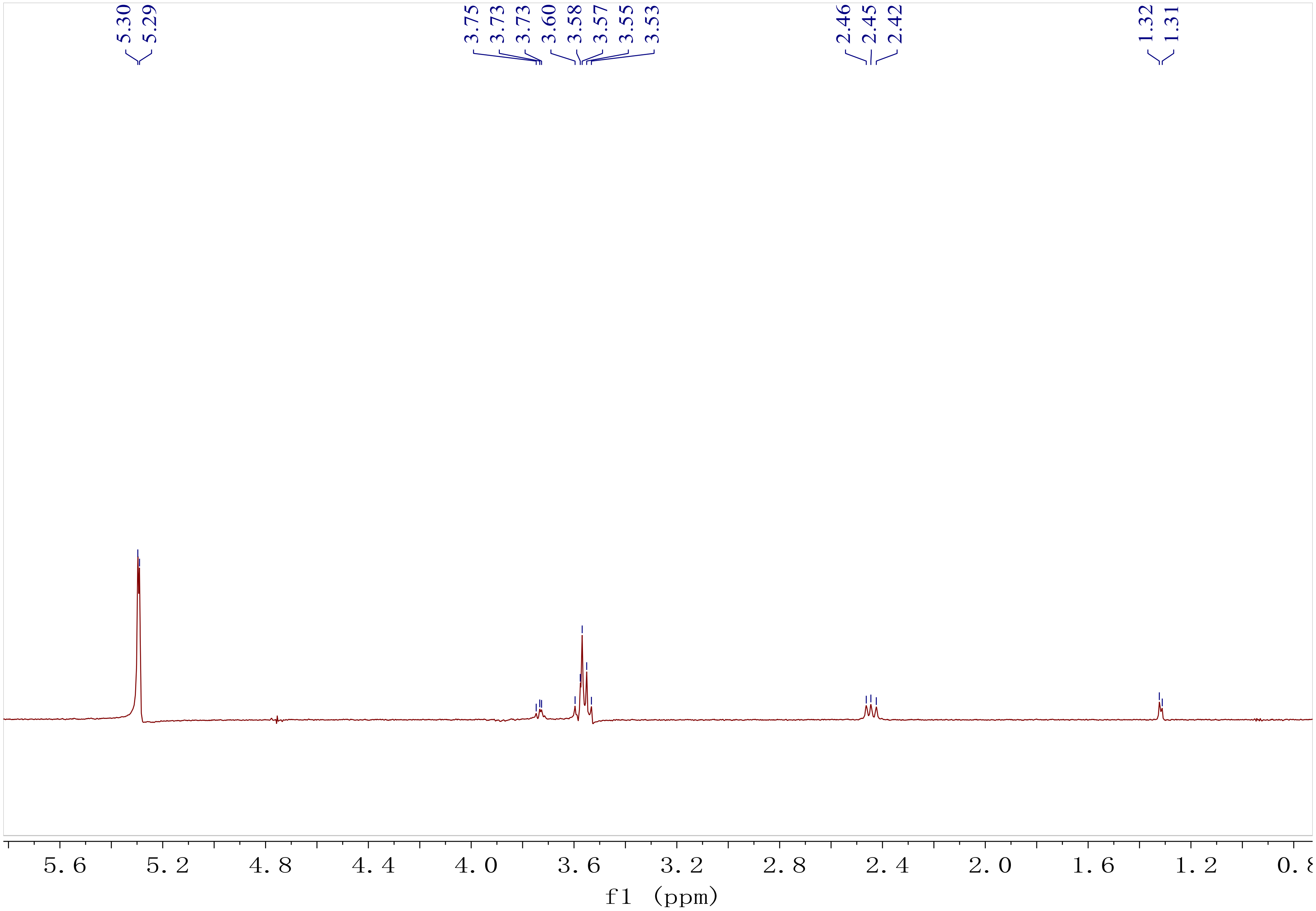

Figure S142. 1D-selective TOCSY spectrum of compound **16** (500 MHz, D_2O, excitation at δ 5.30, H-**H**1).

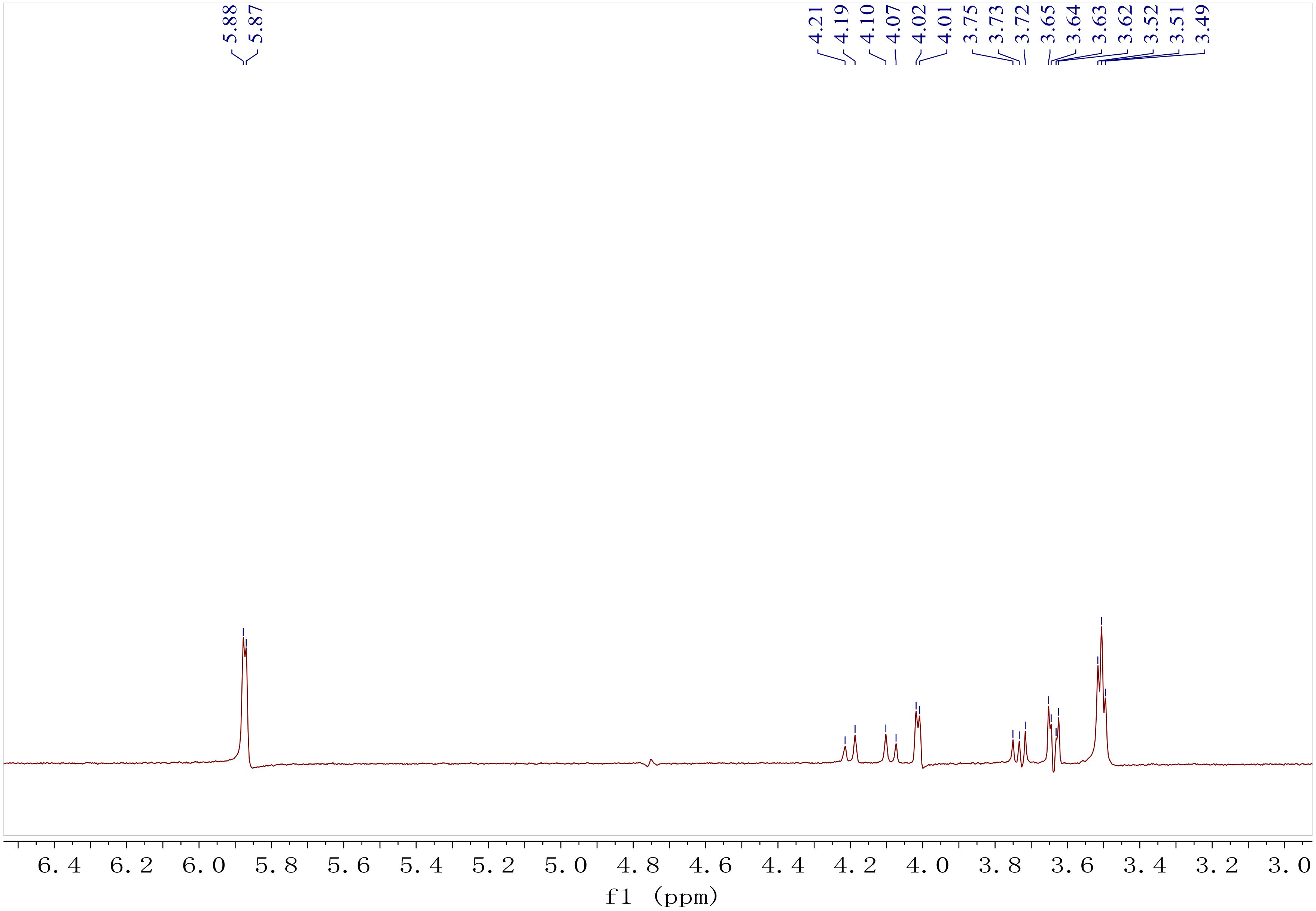

Figure S143. 1D-selective TOCSY spectrum of compound **16** (500 MHz, D_2O, excitation at δ 5.87, H-**I**7).

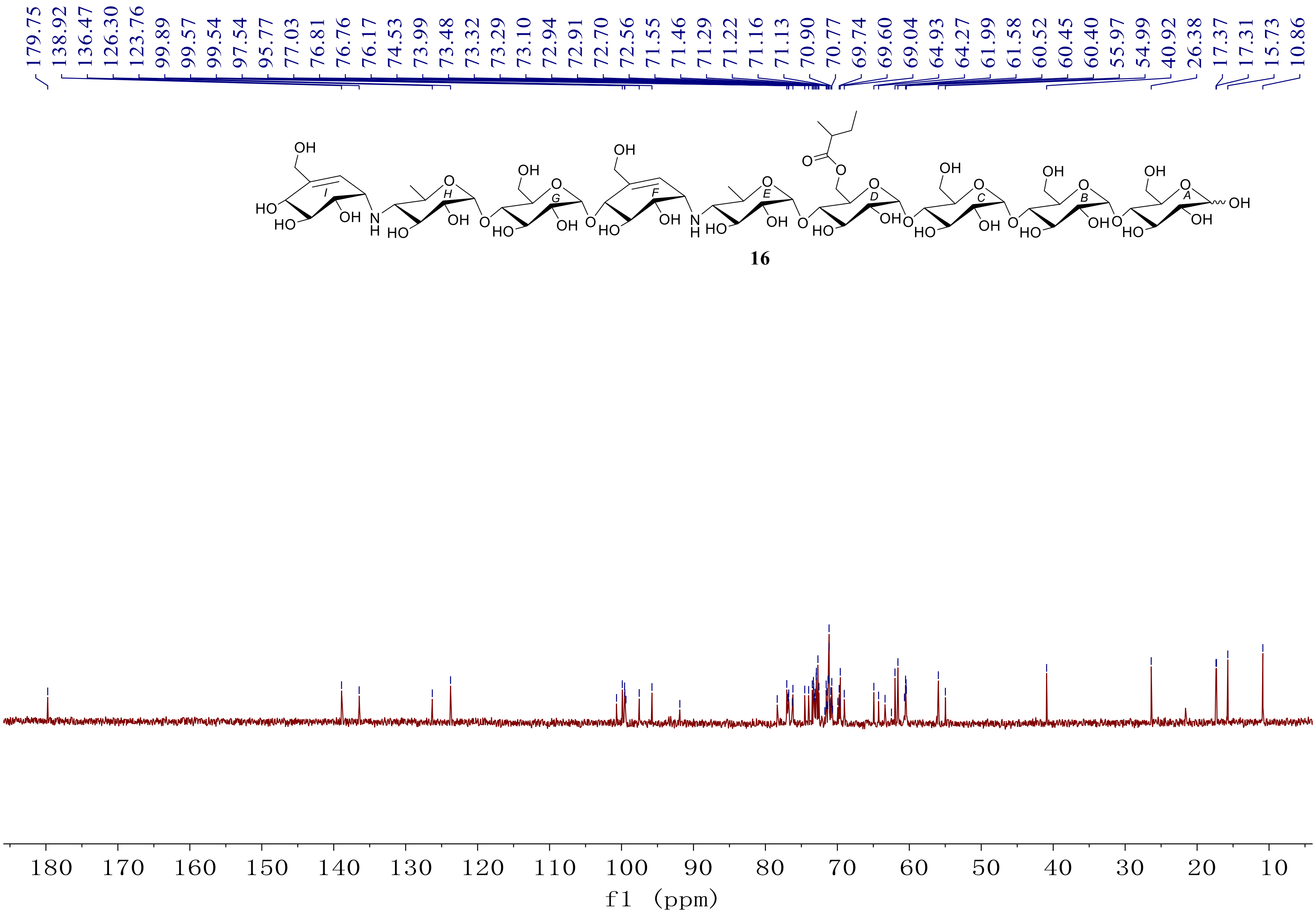

Figure S144. ^{13}C NMR spectrum of compound **16** (125 MHz, D_2O).

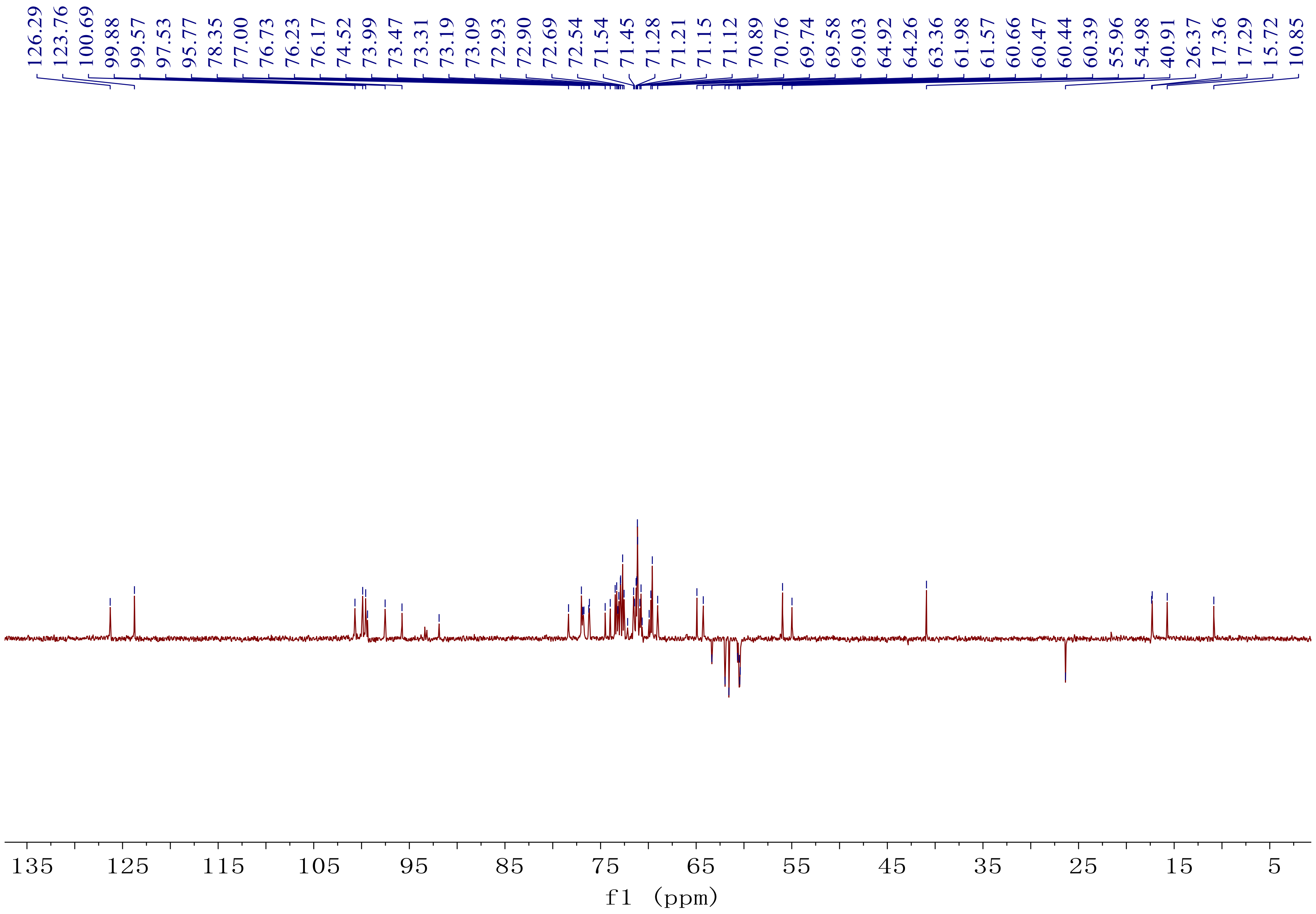

Figure S145. DEPT-135 spectrum of compound **16** (125 MHz, D_2O).

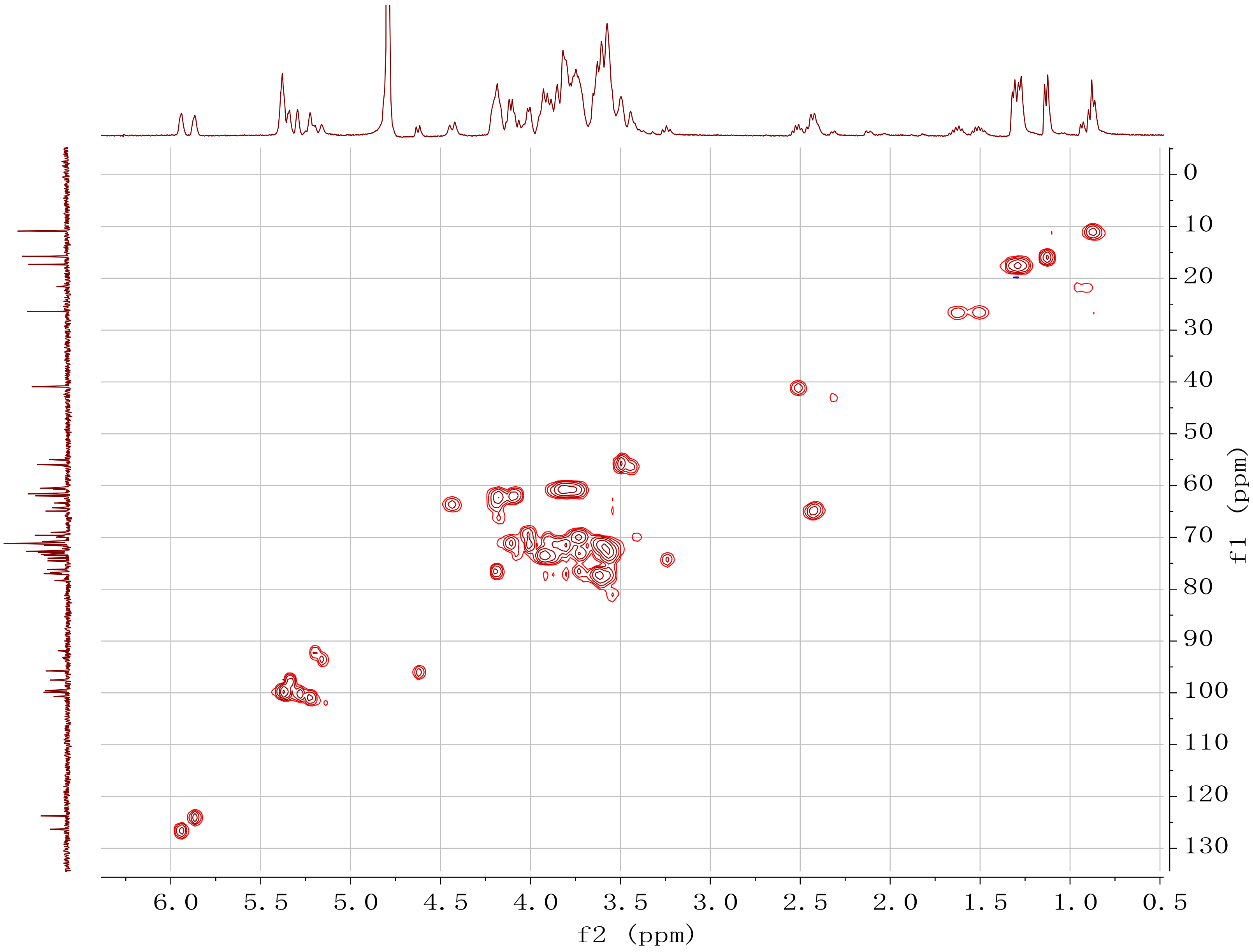

Figure S146. HSQC spectrum of compound **16** (500 MHz, D_2O).

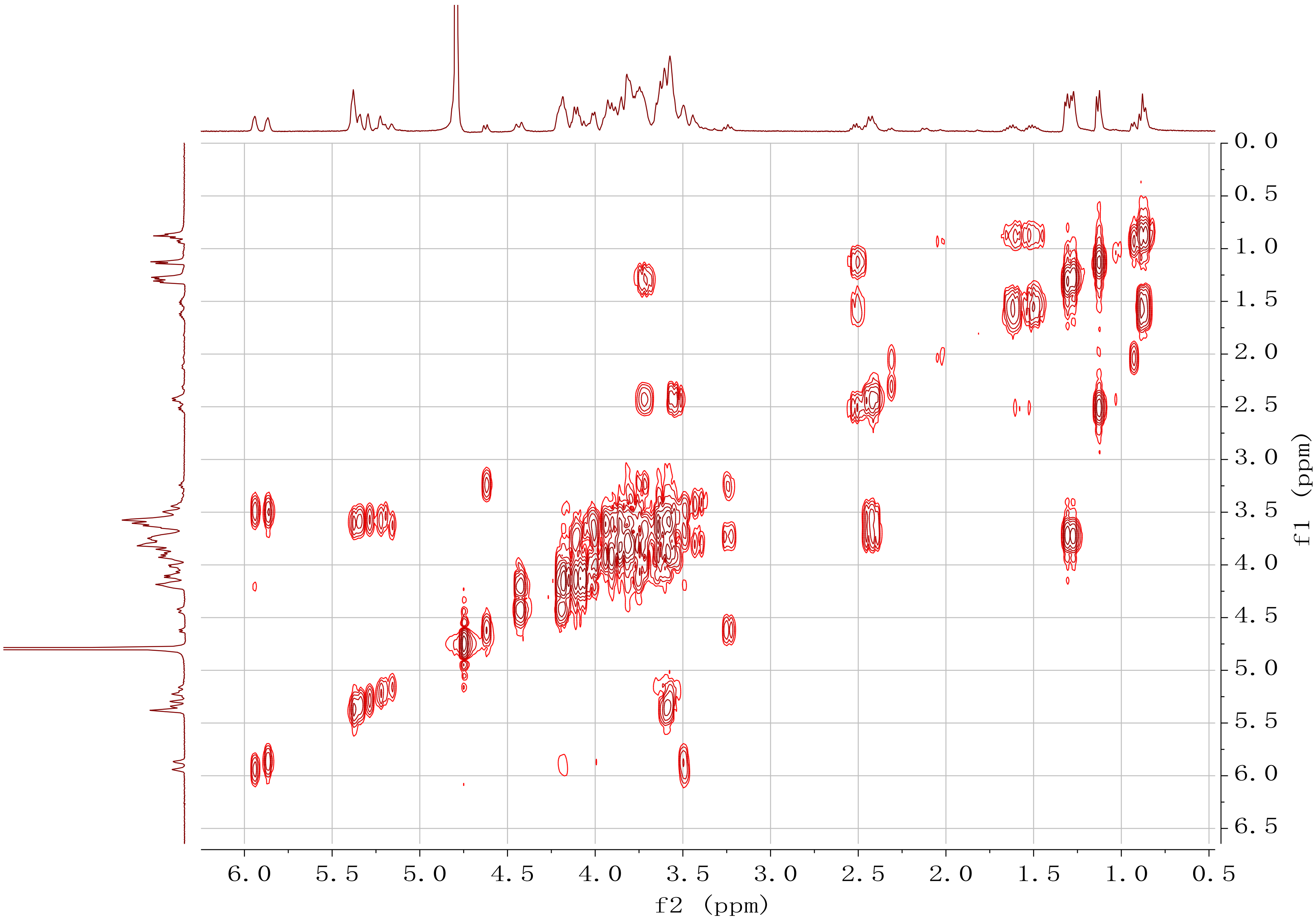

Figure S147. ^{1}H-^{1}H COSY spectrum of compound **16** (500 MHz, D_2O).

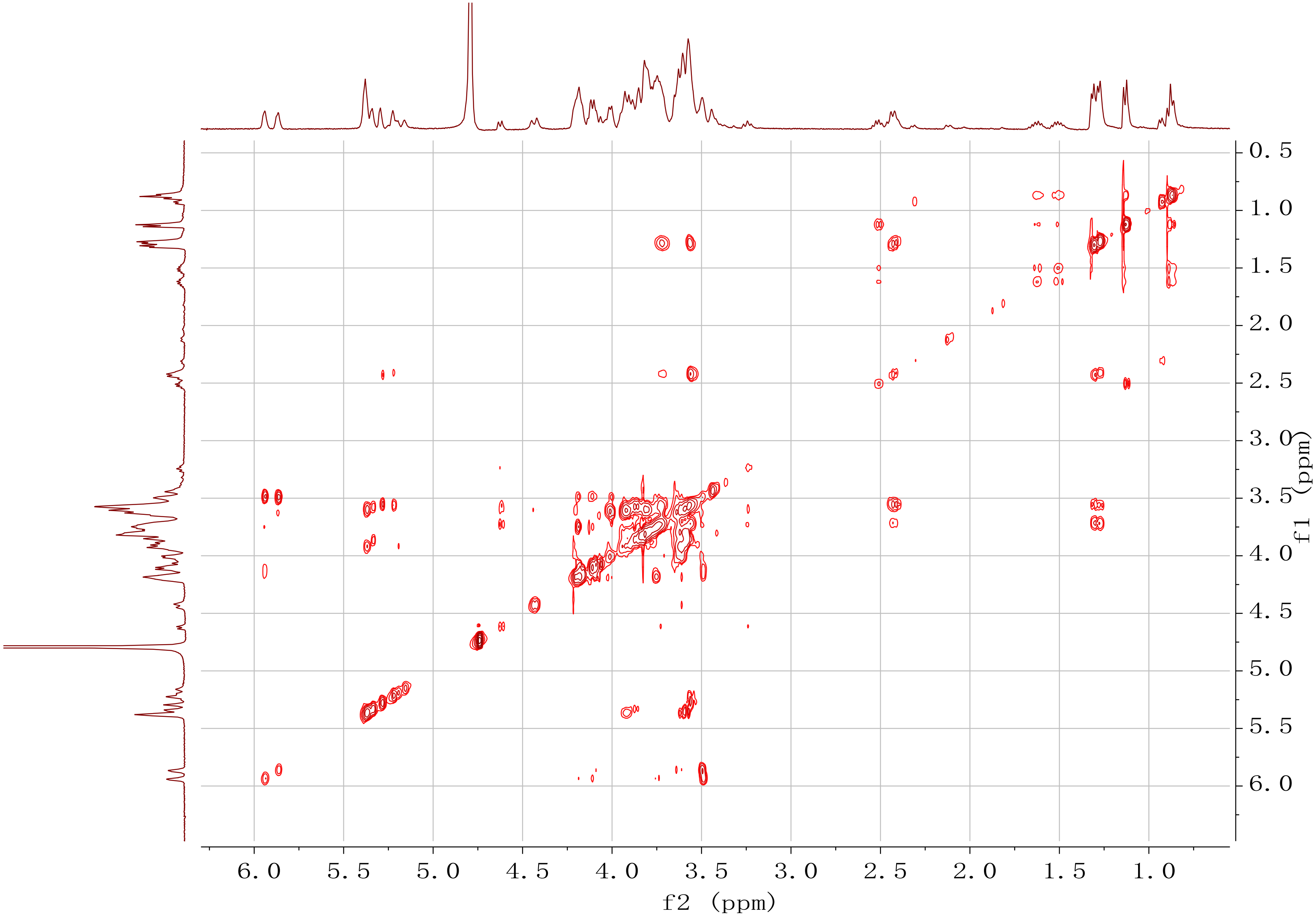

Figure S148. 2D-TOCSY spectrum of compound **16** (500 MHz, D_2O).

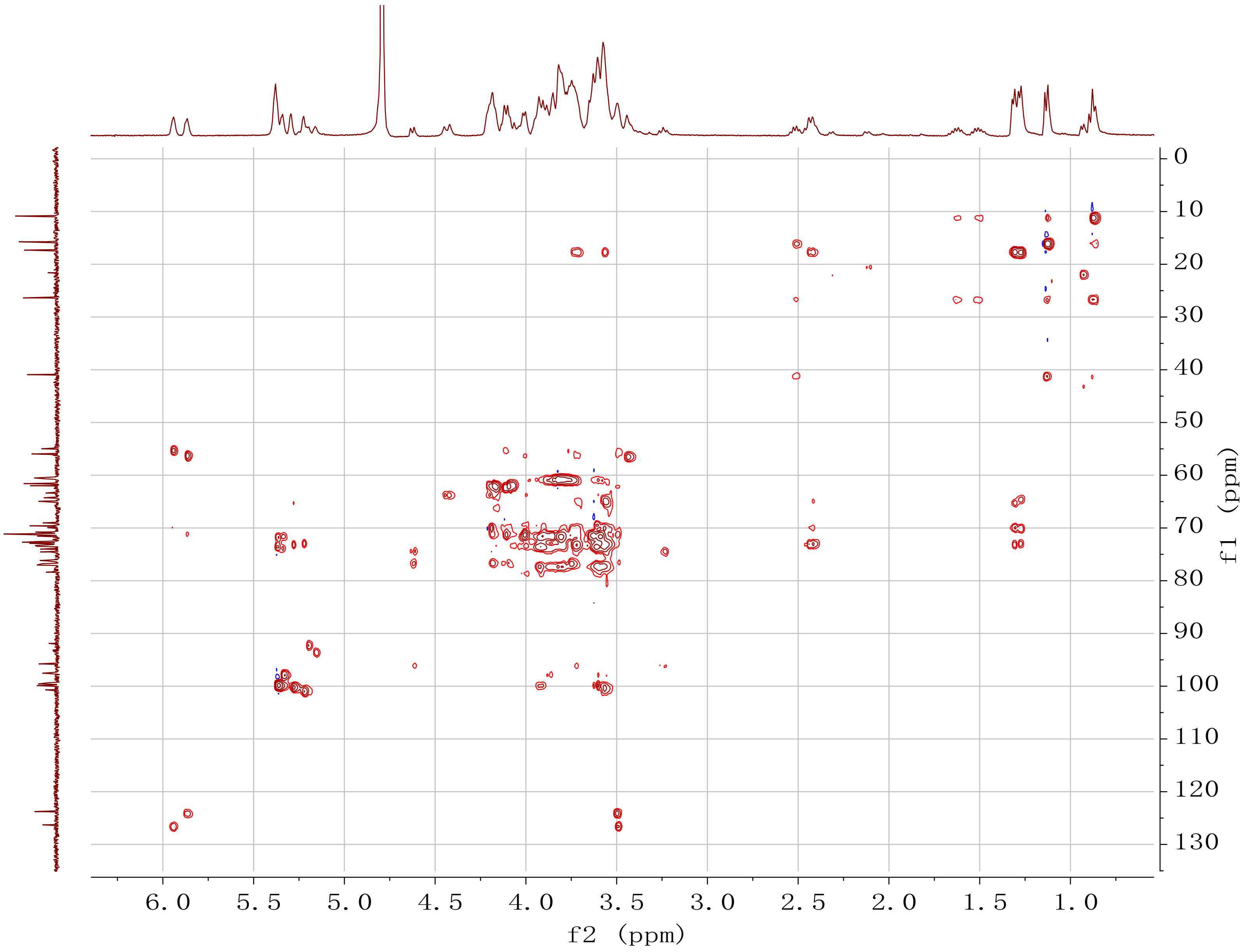

Figure S149. HSQC-TOCSY spectrum of compound **16** (500 MHz, D_2O).

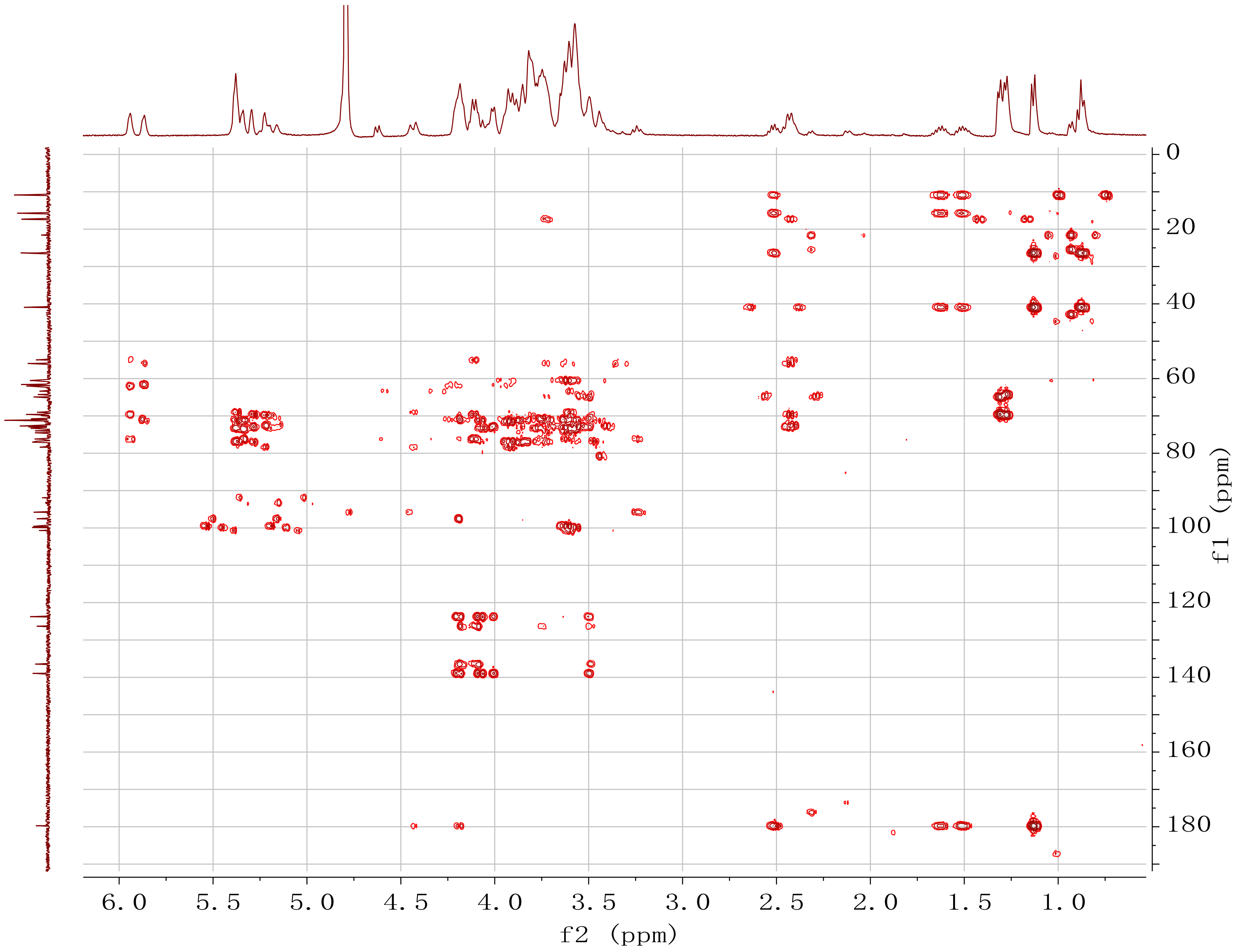

Figure S150. HMBC spectrum of compound **16** (500 MHz, D_2O).

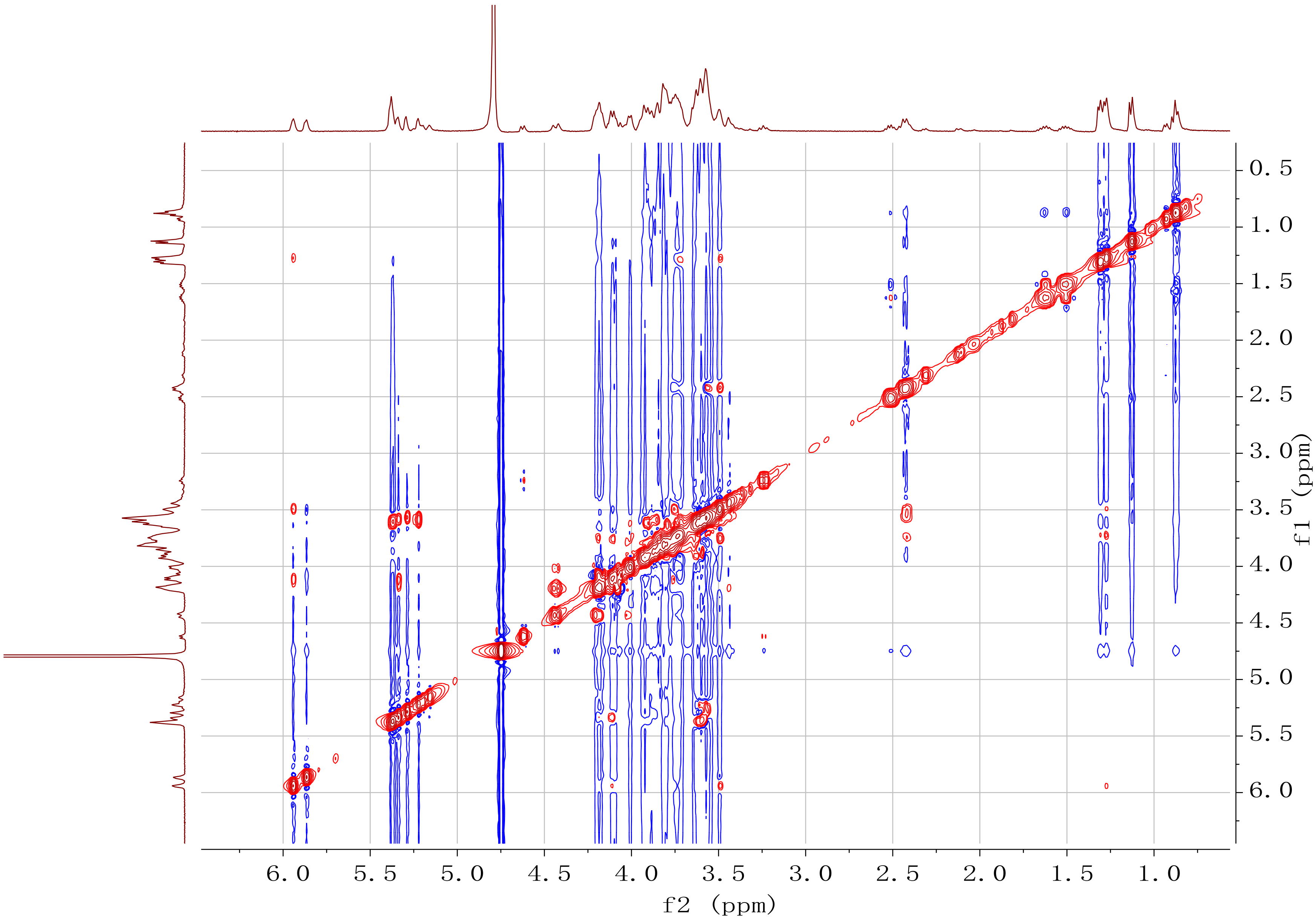

Figure S151. NOESY spectrum of compound **16** (500 MHz, D_2O).

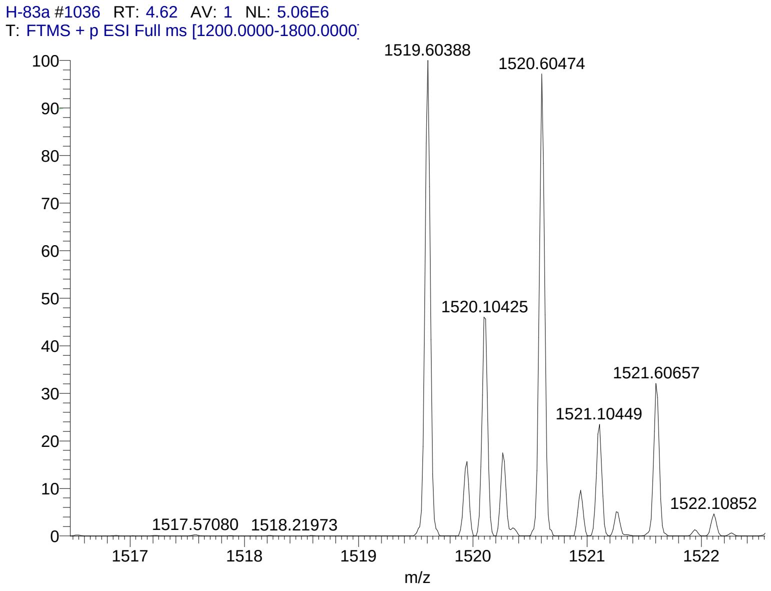

Figure S152. HRESIMS spectrum of compound **16**.

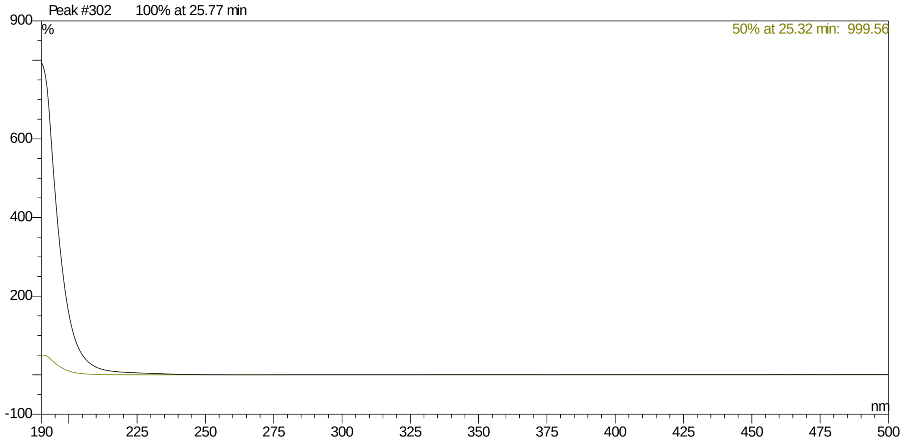

Figure S153. UV spectrum of compound **16**.

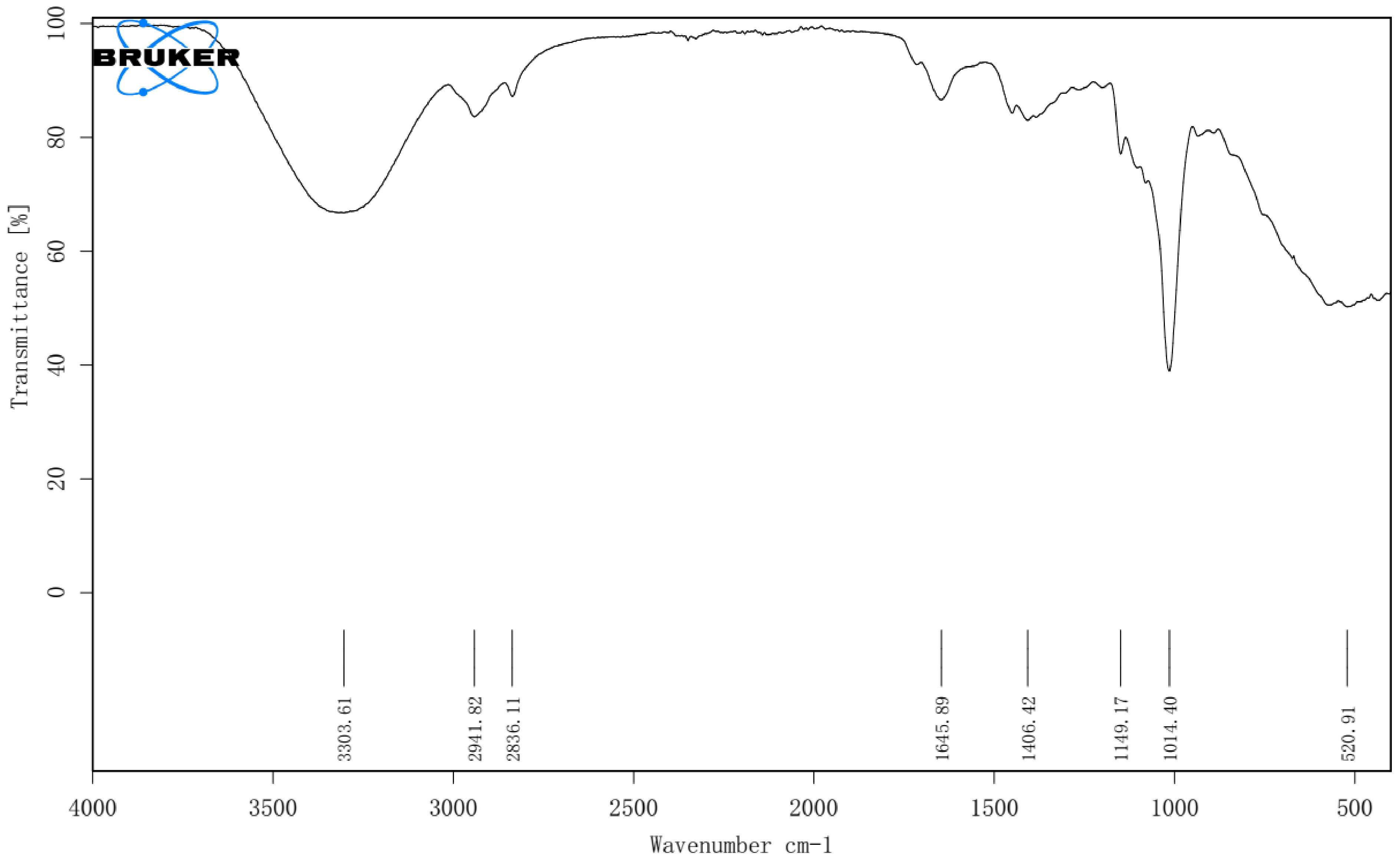

Figure S154. IR spectrum of compound **16**.
